# Simple Access to a Series of Higher Substituted Pentafluoroorthotellurate‐Based Silanes

**DOI:** 10.1002/chem.202503448

**Published:** 2026-01-27

**Authors:** Friederike Oesten, Lukas Fischer, Kurt F. Hoffmann, Niklas Limberg, Simon Steinhauer, Anja Wiesner, Julia Bader, Sebastian Riedel

**Affiliations:** ^1^ Fachbereich Biologie Chemie, Pharmazie Institut für Chemie und Biochemie – Anorganische Chemie Freie Universität Berlin Berlin Germany

**Keywords:** fluorine chemistry, Lewis acid, silicon, pentafluoroorthotellurate

## Abstract

The preparation and characterization of R_x_R′_y_SiOTeF_5_ (R = Me, Et, *^i^* Pr, Ph; x = 3, y = 0; R = Pr, *^t^* Bu, R′ = Me; x = 1, y = 2; 1a‐1f), R_2_Si(OTeF_5_)_2_ (R = Me, Et, *^i^* Pr, Ph; 2a‐2d) and RSi(OTeF_5_)_3_ (R = Me, Et, *^t^* Bu, Ph; 3a‐3d) by reaction of the corresponding chlorosilane and an OTeF_5_ transfer reagent is presented. Molecular structures in the solid state were obtained for Ph_3_SiOTeF_5_ (1f), Ph_2_Si(OTeF_5_)_2_ (2d) and *^t^* BuSi(OTeF_5_)_3_ (3c). Furthermore, the Lewis acidity was assessed by calculating the fluoride ion affinity (FIA), leading to the observation that a higher number of pentafluoroorthotellurate groups results in an increased FIA.

## Graphical abstract

The pentafluoroorthotellurate ligand was introduced to organo silanes. A variety of silanes with the formular R*_x_* Si(OTeF_5_)_4–_ *_x_* were synthesized and characterized via spectroscopic techniques and sc‐XRD. The influence of the number of OTeF_5_ groups on structural properties as well as the fluoride ion affinities (FIA) has been studied and compared to other silanes bearing electron‐withdrawing substituents.

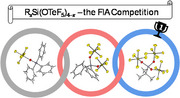

## 1 Introduction

In the last years, the interest in highly Lewis acidic silanes has risen since silicon is the second most abundant element in the earth's crust and possesses a low toxicity [1]. Silicon‐based precursors are produced in a multi‐ton scale, for example, in the Siemens process [2, 3] or the Müller–Rochow process [4]. These properties make a simple access and synthesis on a large scale of silicon‐based Lewis (super)acids desirable. The implementation of strong electron‐withdrawing substituents led to the synthesis of several neutral Lewis (super)acidic compounds such as the pentafluoroethyl silanes Si(C_2_F_5_)_2_Me_2_, Si(C_2_F_5_)_3_R (R = Me, Et, Ph) or Si(C_2_F_5_)_4_ [5, 6], the perhalogenated bis(catecholato)silane Si(cat^X^)_2_ (X = Cl, Br) [7, 8], perfluorocresolato silane Si(OTol^*F*^)_4_ [9] or the silicon trifluoromethanesulfonates Ph_x_Si(OTf)_4‐x_ (x = 1‐3) [10, 11, 12, 13, 14, 15, 16, 17] and Si(OTf)_4_ [18]. However, among the known silicon‐based Lewis acids only a few pentafluoroorthotellurate‐based (OTeF_5_, teflate) compounds are reported in the literature, namely Me_3_SiOTeF_5_ [19, 20], Et_3_SiOTeF_5_ [21], Ph_3_SiOTeF_5_ [22], and F_3_SiOTeF_5_ [23]. Me_3_SiOTeF_5_ was reported already in 1973 [20] but was finally fully characterized 45 years later [19]. Si(OTeF_5_)_4_ was only briefly mentioned and its characterization was limited to the melting point of 37°C [20]. Compounds of the formula R_2_Si(OTeF_5_)_2_ or RSi(OTeF_5_)_3_ are not reported.

Lewis acids are of general interest and find application in many fields of chemistry, whether in homogenous or heterogenous catalysis [24], in weakly coordinating anions (WCAs) [25] or in frustrated Lewis pairs (FLP) [26, 27]. The acidity of a Lewis acid depends on its substituents at the central atom. The replacement of monoatomic ligands like the fluorido ligand with bulkier groups with similar electron‐withdrawing properties, leads to an increased Lewis acidity, paving the way to a diverse reactivity resulting in elusive cations [25] or the activation of small molecules [26, 28, 29]. A common method to assess the Lewis acidity theoretically is the fluoride ion affinity (FIA), which corresponds to the negative binding enthalpy Δ*H* of the fluoride ion with a Lewis acid [30]. Experimentally, the Lewis acidity can be determined *via* the ^31^P NMR chemical shift of free triethylphosphine oxide, OPEt_3_, compared to the shift of its Lewis acid complex (Gutmann‐Beckett method [31, 32]) or the blue‐shifted *ν*(CN) stretching mode of CD_3_CN after binding to a Lewis acid [33, 34]. Krossing et al. coined the term “Lewis superacid” for molecular Lewis acids that are stronger than monomeric SbF_5_ in the gas phase [30]. Those Lewis superacids often consist of a central atom bound to sterically demanding moieties such as fluorinated/halogenated aryl derivatives, alkoxy ligands, the trifluoromethanesulfonate group, or the pentafluoroorthotellurate ligand [35]. We recently reported on the properties and reactivity of the Lewis superacid Al(OTeF_5_)_3_. Its FIA of 591 kJ mol^−1^ (calculated on BP86/SV(P)) [36] is significantly higher than that of the benchmark compound SbF_5_ (calc. FIA: 493 kJ mol^−1^ on BP86/SV(P)) [37]. This high FIA value results from the sterically demanding teflate group which has electron‐withdrawing properties that can be compared to that of fluorine [38]. Compared to other oxygen‐based ligands, the binding oxygen atom of the teflate group exhibits reduced π‐back‐donation due to electron withdrawal by the peripheral fluorine atoms. Thus, the teflate ligand can serve as a fluorine substitute leading to a higher Lewis acidity in the corresponding compounds [39, 40]. Further advantages of the teflate group are the robustness against electrophiles and oxidation due to the high charge delocalization [40, 41].

Herein we present a series of silicon teflates with the formula R_x_R′_y_SiOTeF_5_ (R = Me, Et, *^i^* Pr, Ph; x = 3, y = 0; R = Pr, *^t^* Bu, R′ = Me; x = 1, y = 2; 1a‐1f), R_2_Si(OTeF_5_)_2_ (R = Me, Et, *^i^* Pr, Ph; 2a‐2d) as well as RSi(OTeF_5_)_3_ (R = Me, Et, *^t^* Bu, Ph; 3a‐3d). The spectroscopic data and the FIA values show a strong relation to the number of teflate groups bound to the silicon centre and are exemplarily discussed with Ph_3_SiOTeF_5_ (1f), Ph_2_Si(OTeF_5_)_2_ (2d) as well as *^t^* BuSi(OTeF_5_)_3_ (3c). Finally, the molecular structures of 1f, 2d, and 3c were determined *via* single‐crystal X‐ray diffraction.

## 2 Results and Discussion

**SCHEME 1:**
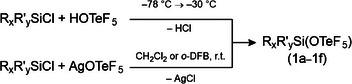
Synthesis of R_x_R′_y_SiOTeF_5_ with R = Me, Et, *^i^* Pr, Ph; x = 3, y = 0; R = Pr, *^t^* Bu, R′ = Me; x = 1, y = 2; 1a‐1f).

Ph_3_SiOTeF_5_ is literature‐known and was so far only characterized by ^19^F and ^13^C NMR spectroscopy. It was synthesized by the reaction of Ph_3_SiCl with TlOTeF_5_ [22]. Following the reported procedure for Me_3_SiOTeF_5_ [19], we prepared Ph_3_SiOTeF_5_ (1f) by the treatment of neat Ph_3_SiCl with HOTeF_5_ as a teflate transfer reagent. Additionally, compound 1f can be accessed by the reaction of Ph_3_SiCl with AgOTeF_5_ in *o*‐DFB (Scheme 1). Furthermore, various alkyl chlorosilanes were used and either HOTeF_5_ or AgOTeF_5_ was employed as the teflate transfer reagent to form Et_3_SiOTeF_5_ (1b), Me_2_PrSiOTeF_5_ (1c), *^i^* Pr_3_SiOTeF_5_ (1d), and *^t^* BuMe_2_SiOTeF_5_ (1e).

**FIGURE 1:**
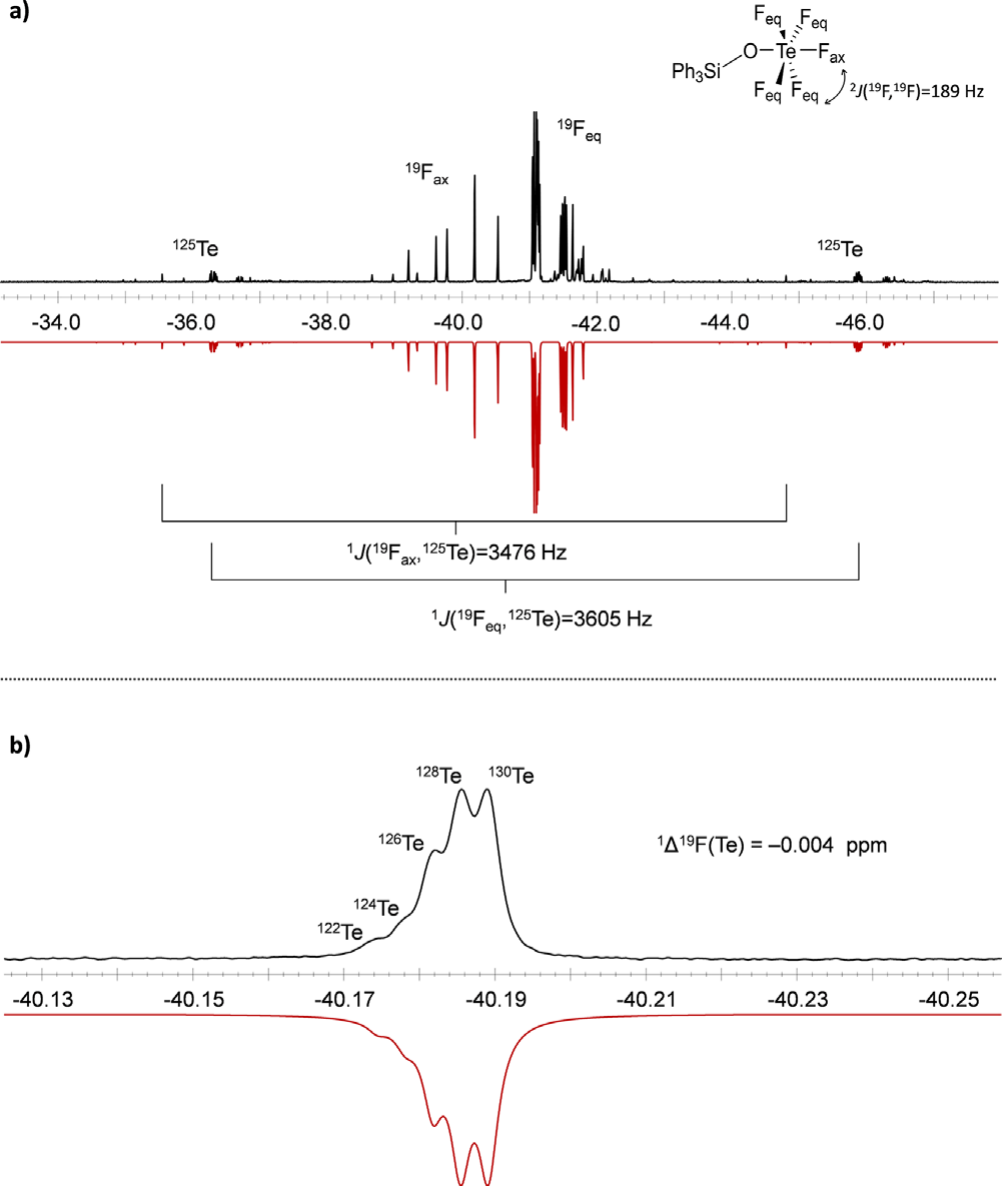
(a) Experimental (top) and simulated (bottom) ^19^F NMR spectrum (377 MHz, *o*‐DFB, r.t.) of Ph_3_SiOTeF_5_ (1f). (b) Section of the experimental (top) and simulated (bottom) pseudo‐quintett of the ^19^F NMR spectrum of Ph_3_SiOTeF_5_ (1f).

**FIGURE 2:**
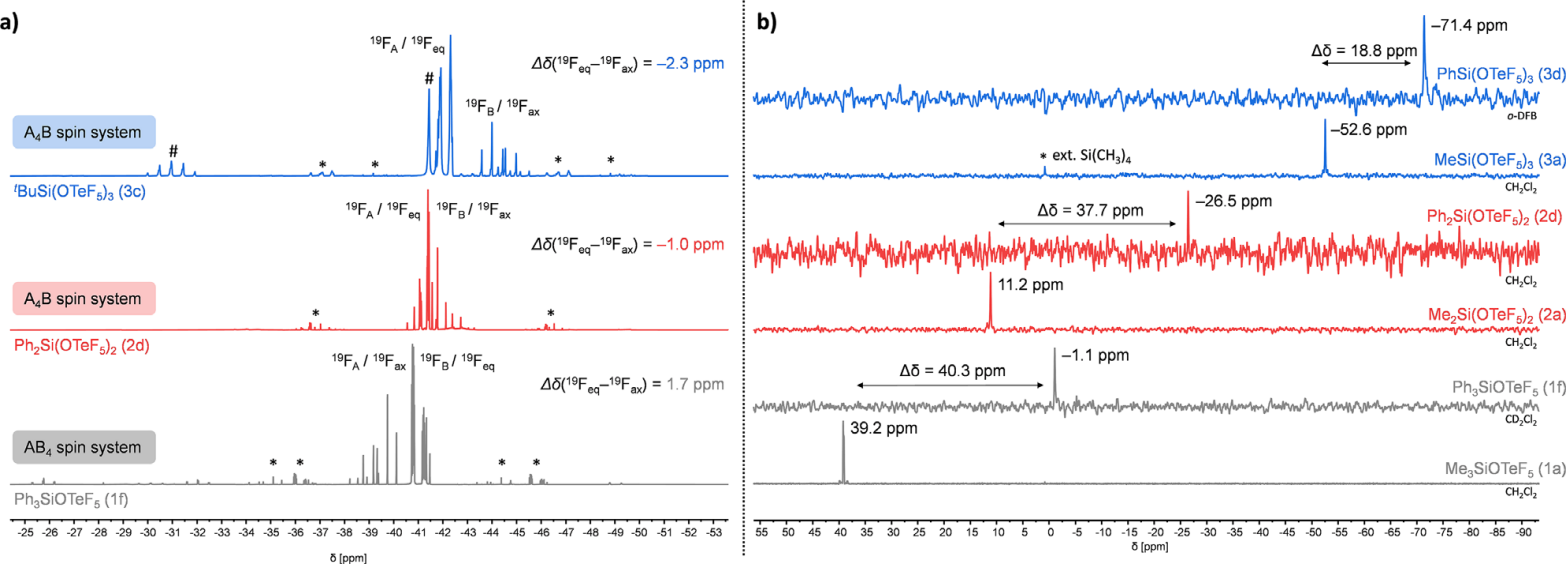
^19^F NMR spectra (left, 377 MHz, r.t., CH_2_Cl_2_) for Ph_3_SiOTeF_5_ (1f), Ph_2_Si(OTeF_5_)_2_ (2d) and *^t^* BuSi(OTeF_5_)_3_ (3c) (from bottom to top). The ^125^Te satellites are marked with an asterisk (*). AgOTeF_5_ is marked with a hash (#). Comparison of the ^29^Si DEPT NMR spectra (right, 80 MHz, r.t.) for Me_3_SiOTeF_5_ (CH_2_Cl_2_; 1a), Ph_3_SiOTeF_5_ (CD_2_Cl_2_; 1f), Me_2_Si(OTeF_5_)_2_ (CH_2_Cl_2_; 2a), Ph_2_Si(OTeF_5_)_2_ (CH_2_Cl_2_; 2d), MeSi(OTeF_5_)_3_ (CH_2_Cl_2_; 3a), and PhSi(OTeF_5_)_3_ (*o*‐DFB; 3d, from bottom to top).

The ^19^F NMR spectrum of 1f (Figure 1a) reveals the distinctive AB_4_X spin system for the teflate group with signals at *δ*(^19^F_ax_) = −39.3 ppm and *δ*(^19^F_eq_) = −41.0 ppm and the corresponding ^2^ *J*(^19^F_ax_,^19^F_eq_) coupling constant of 189 Hz. The ^1^ *J*(^19^F_ax_,^125^Te) and ^1^ *J*(^19^F_eq_,^125^Te) coupling constants are found to be 3476 and 3612 Hz. These findings are in good agreement with reported values in the literature [22] and were obtained by the simulation of the ^19^F NMR spectrum [42]. Interestingly, the isotopic shift pattern of the spinless tellurium isotopes Ph_3_SiO^130^TeF_5_, Ph_3_SiO^128^TeF_5_, Ph_3_SiO^126^TeF_5_, Ph_3_SiO^124^TeF_5_, and Ph_3_SiO^122^TeF_5_ are resolved. The secondary isotopic shift of ^1^Δ^19^F(^130,128^Te) = −0.004 ppm results to a splitting of 1.3 Hz at a measuring frequency of 377 MHz (Figure 1b). This value is in the same range as for the [TeF_7_]^−^ anion (^1^Δ^19^F(^130,128^Te) = −0.0042 ppm) [43]. While all monosubstituted teflate species 1a‐1f possess ^19^F NMR chemical shift values in a similar range, alkylated and arylated species can be differentiated in the ^29^Si DEPT NMR spectra. Here, the chemical shift occurs between 35.8−40.6 ppm for all alkylated compounds. However, Ph_3_SiOTeF_5_ (1f) shows an upfield‐shifted chemical shift of *δ*(^29^Si) = −1.1 ppm (Table 1 and Figure 2). This trend is also observed for the analogous triflate compounds Me_3_SiOTf and Ph_3_SiOTf (*δ*(^29^Si, Me_3_SiOTf) = 43.1 ppm [44], *δ*(^29^Si, Ph_3_SiOTf)  = 1.7 ppm [45]).

**TABLE 1:** ^19^F NMR parameter for R_x_Si(OTeF_5_)_y_ (x = 3, 2, 1; y = 1, 2, 3).

	Chemical shift (δ)^d^, ^e^ [ppm]				Coupling constant (*J*)^d^ [Hz]	
Species	^19^F_ax_	^19^F_eq_	^29^Si	*Δδ*(^19^F_eq_–^19^F_ax_) [ppm]	^1^ *J*(^19^F_ax_,^125^Te) / ^1^ *J*(^19^F_eq_,^125^Te)	^2^ *J*(^19^F_ax_,^19^F_eq_)
Me_3_SiOTeF_5_ (1a)^a^	−39.8	−43.8	39.2	4.0	3420/3556	186
Et_3_SiOTeF_5_ (1b)^a^	−39.2	−43.9	40.6	4.7	3424/3581	188
Me_2_PrSiOTeF_5_ (1c)^a^	−39.0	−43.2	39.4	4.2	3423/3577	187
*^i^* Pr_3_SiOTeF_5_ (1d)^a^	−38.7	−43.8	35.8	5.1	3442/3586	190
*^t^* BuMe_2_SiOTeF_5_ (1e)^a^	−39.1	−43.6	39.7	4.5	3428/3583	187
Ph_3_SiOTeF_5_ (1f)^a^, ^b^	−39.3	−41.0	−1.1	1.7	3476/3612	189
Me_2_Si(OTeF_5_)_2_ (2a)^a^	−41.9	−42.4	11.2	0.5	3544/3586	188
Et_2_Si(OTeF_5_)_2_ (2b)^a^	−42.2	−43.0	8.9	0.8	3538/3592	188
*^i^* Pr_2_Si(OTeF_5_)_2_ (2c)^a^	−42.3	−43.4	3.6	1.1	3533/3580	188
Ph_2_Si(OTeF_5_)_2_ (2d)^a^	−43.0	−42.0	−26.5	−1.0	3560/3611	188
MeSi(OTeF_5_)_3_ (3a)^a^	−44.4	−41.7	−52.6	−2.7	3643/3610	187
EtSi(OTeF_5_)_3_ (3b)^a^	−44.5	−42.0	−55.3	−2.5	3639/3610	187
*^t^* BuSi(OTeF_5_)_3_ (3c)^a^	−44.6	−42.3	−62.4	−2.3	3634/3625	187
PhSi(OTeF_5_)_3_ (3d)^c^	−45.8	−42.3	−71.4	−3.5	3641/3629	187

^a^ NMR spectra were recorded in CH_2_Cl_2_ at r.t. with acetone‐d6 as external standard.

^b^ NMR spectra were recorded in CD_2_Cl_2_ at r.t.

^c^ NMR spectra were recorded in *o*‐DFB at r.t. with acetone‐d6 as external standard.

^d^ Chemical shifts and coupling constants of ^19^F NMR spectra are given as simulated by gNMR [42].

^e^ Within the AB_4_X or A_4_BX spin system of the –OTeF_5_ group axial and equatorial fluorine atoms are described as F_ax_ and F_eq_.

**SCHEME 2:**
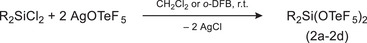
Synthesis of R_2_Si(OTeF_5_)_2_ with R = Me, Et, *^i^* Pr and Ph (2a–2d).

Next, we investigated the substitution of two chlorido ligands in R_2_SiCl_2_ (R = alkyl, aryl) by OTeF_5_ groups (Scheme 2).

The application of HOTeF_5_ as a teflate transfer reagent was unsuccessful. However, by employing AgOTeF_5_ in CH_2_Cl_2_ or *o*‐DFB the compounds Me_2_Si(OTeF_5_)_2_ (2a), Et_2_Si(OTeF_5_)_2_ (2b), *^i^* Pr_2_Si(OTeF_5_)_2_ (2c), and Ph_2_Si(OTeF_5_)_2_ (2d) were formed.

While the chemical shift difference of the axial and equatorial fluorine nuclei in the ^19^F NMR spectrum (*Δδ*(^19^F_eq_–^19^F_ax_)) is between 4.0 and 5.1 ppm for the monosubstituted silicon teflates and 1.7 ppm for the aryl‐substituted species Ph_3_SiOTeF_5_ (1f), it becomes smaller for 2a–2d (Table 1) and results in a partial overlap of the two signals. In the case of R = Me, Et, and *^i^* Pr the AB_4_X spin system is still present with chemical shift difference *Δδ*(^19^F_eq_–^19^F_ax_) between 0.5 and 1.1 ppm. Only in the case of Ph_2_Si(OTeF_5_)_2_ (2d) the two signals are inversed, resulting in an A_4_BX spin system with a shift difference of −1.0 ppm. The ^2^ *J*(^19^F_ax_,^19^F_eq_) coupling constant for 2a–2d of 188 Hz is in a similar range as for the monosubstituted compounds.

In the ^29^Si DEPT NMR spectra we observe the same behaviour as for the monosubstituted silanes — the signal of the arylated teflate‐based silane is upfield‐shifted compared to the alkylated silanes. The chemical shift of Ph_2_Si(OTeF_5_)_2_ (2d) is *δ*(^29^Si) = −26.5 ppm and therefore in the same range as the corresponding triflate compound Ph_2_Si(OTf)_2_ with *δ*(^29^Si) = −24.6 ppm [45]. The chemical shifts of 2a–2c range between 3.6 and 11.2 ppm, which is in good agreement of the literature‐known value of Me_2_Si(OTf)_2_ (*δ*(^29^Si) = 15.3 ppm) [46].

**SCHEME 3:**
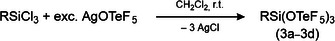
Synthesis of RSi(OTeF_5_)_3_ with R = Me, Et, *^t^* Bu and Ph (3a–3d).

Further, we introduced a third teflate group to the silicon centre. Interestingly, the reaction of RSiCl_3_ and three equivalents of AgOTeF_5_ led to an incomplete substitution, but a slight excess of AgOTeF_5_ resulted in the formation of the trisubstituted teflate compounds MeSi(OTeF_5_)_3_ (3a), EtSi(OTeF_5_)_3_ (3b), *^t^* BuSi(OTeF_5_)_3_ (3c), and PhSi(OTeF_5_)_3_ (3d; Scheme 3).

The excess of AgOTeF_5_ can be partially crystallized from CH_2_Cl_2_ but traces of AgOTeF_5_ always remain in solution. With *Δδ*(^19^F_eq_–^19^F_ax_) ranging from −2.3 to −3.5 ppm the signals in the ^19^F NMR spectra appear as pseudo‐doublet and pseudo‐quintet, resulting in an A_4_BX spin system. The ^19^F chemical shifts as well as the coupling constants are similar for 3a–3d (Table 1 and Figure 2). The chemical shifts of 3a–3d in the ^29^Si DEPT NMR spectra vary between *δ*(^29^Si) = −52.6 ppm (MeSi(OTeF_5_)_3_; 3a) and *δ*(^29^Si) = −71.4 ppm (PhSi(OTeF_5_)_3_: 3d; Table 1). The reported chemical shift for MeSi(OTf)_3_ of *δ*(^29^Si) = −57.3 ppm [10] is comparable to that of the teflate compound. Again, the phenyl‐substituted compound possesses the highest upfield chemical shift with *δ*(^29^Si)  = −71.4 ppm. Compared to the mono‐ and bisubstituted silicon teflates the ^29^Si NMR chemical shift is for all RSi(OTeF_5_)_3_ upfield shifted. Overall, it is noticeable that the difference in the ^29^Si NMR chemical shift between the alkylated and the arylated substances becomes smaller within the substitution stages. Furthermore, for some species a ^2^ *J*(^125^Te,^29^Si) coupling constant (Me_2_PrSiOTeF_5_: 123 Hz; 1c) or a ^2^ *J*(^19^F,^29^Si) coupling constant (*^i^* Pr_3_SiOTeF_5_: 4 Hz; 1d) is detected. For Me_2_PrSiOTeF_5_ (1c) also a ^2^ *J*(^125^Te,^13^C) coupling constant of 26 Hz is resolved. For all R_x_Si(OTeF_5_)_4‐x_ (x = 3, 2, 1) ^125^Te NMR spectra were recorded. All of them show a doublet of quintet with ^1^ *J*(^19^F,^125^Te) coupling constants between 3420 Hz and 3644 Hz which increase the more teflate groups are introduced in the molecule.

**FIGURE 3:**
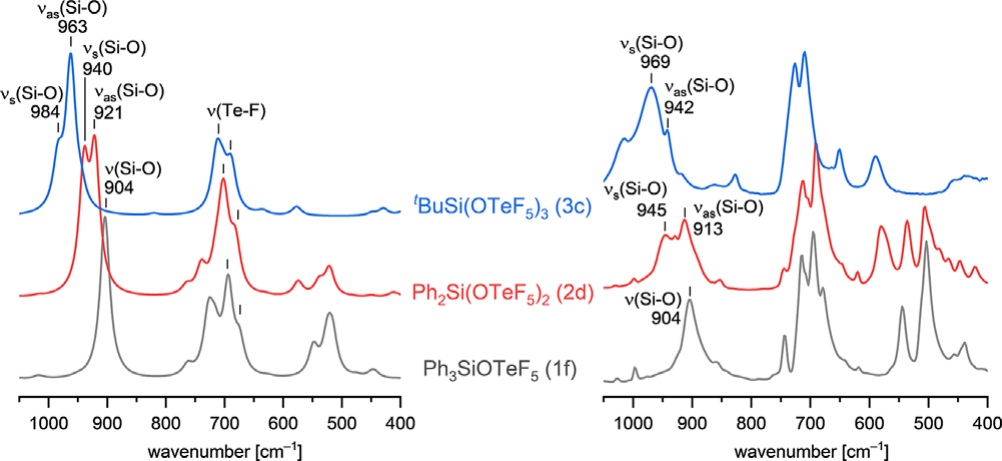
Calculated (left) and experimental (right) spectra of Ph_3_SiOTeF_5_ (1f), Ph_2_Si(OTeF_5_)_2_ (2d), and *^t^* BuSi(OTeF_5_)_3_ (3c, from bottom to top).

The compounds 1f, 2d, and 3c were analyzed by IR spectroscopy (Figure 3) showing a relation between the number of teflate substituents and the absorption band of the Si–O vibrational modes. With an increasing teflate content the Si–O stretching vibrations are blue‐shifted from 904 cm^−^^1^ (1f) to 969 cm^−^^1^ (3c). Compared to literature‐known Me_3_SiOTeF_5_ (931 cm^−^^1^) [19] the Si–O stretching frequency of the phenyl derivative 1f is red‐shifted (904 cm^−^^1^). The calculated stretching frequencies are in good agreement with the experimental stretching vibrations (Table 2).

**TABLE 2:** Comparison of the experimental and calculated stretching frequencies (in cm^−1^) at B3LYP/def2‐TZVPP level of theory of Ph_3_SiOTeF_5_ (1f), Ph_2_Si(OTeF_5_)_2_ (2d) and *^t^* BuSi(OTeF_5_)_3_ (3c). The calculated stretching frequencies are in brackets.

Species	*ν*(Si–O)	*ν*_as_(Si–O)	*ν*_s_(Si–O)
Ph_3_SiOTeF_5_ (1f)	904 (904)	−	−
Ph_2_Si(OTeF_5_)_2_ (2d)	−	913 (921)	945 (940)
*^t^* BuSi(OTeF_5_)_3_ (3c)	−	942 (961, 963)	969 (984)

**FIGURE 4:**
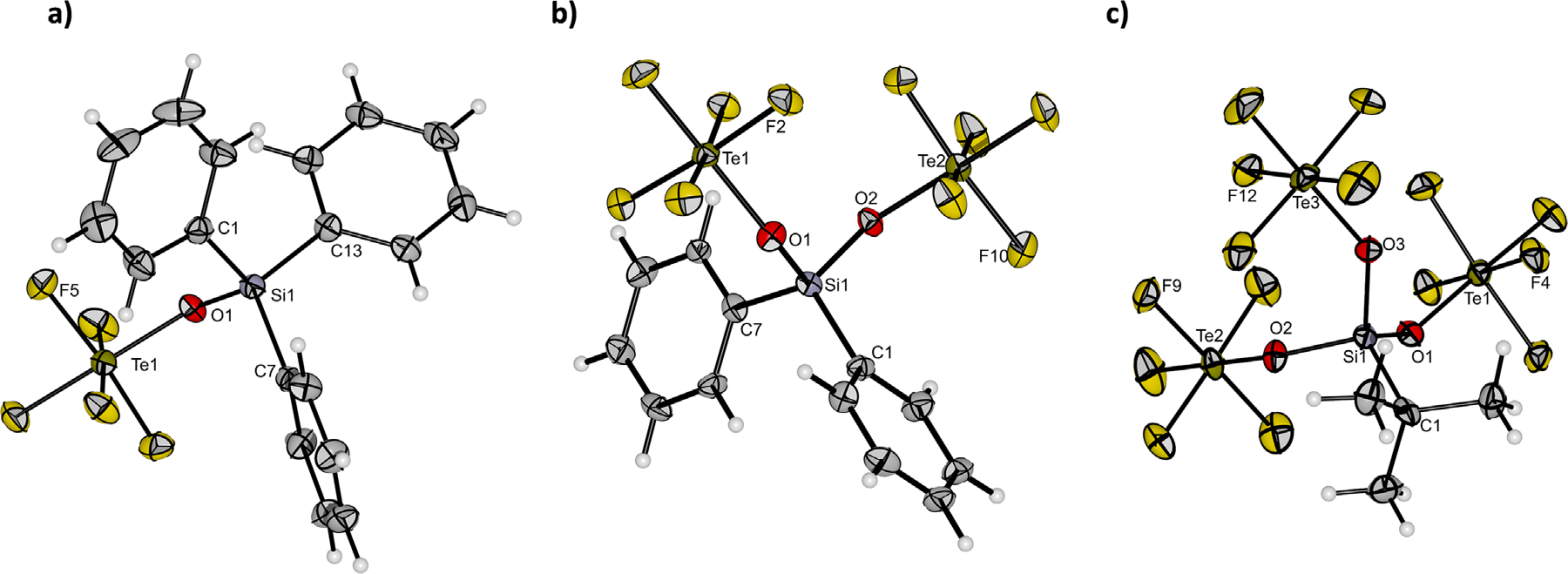
Molecular structure of (a) Ph_3_SiOTeF_5_ (1f), (b) Ph_2_Si(OTeF_5_)_2_ (2d), and (c) *^t^* BuSi(OTeF_5_)_3_ (3c) in the solid state. Thermal ellipsoids are set at 50% probability. Selected bond length [pm] and angles [°] for a): Si1–O1 170.8(2), O1–Te1 182.8(8), Si1–C1 185.9(4), Si1–C7 185.2(4), Si1–C13 185.7(4), O1–Si1–C1 109.91(14), O1–Si1–C13 101.86(14), O1–Si1–C7 107.08(14), C13‐Si1–C1 111.73(16), C1‐Si1–C7 112.05(16). Selected bond length [pm] and angles [°] for (b): Si1–O1 166.8(4), O1–Te1 183.3(4), O2–Te2 184.2(3), Si1–O2 167.3(4), Si1–C1 183.0(5), Si1‐C7 184.3(5), O1–Si1–O2 106.49(19), O1–Si1–C1 105.2(2), O1–Si1–C7 112.7(2), O2–Si1–C1 111.7(2), O2–Si1–C7 105.1(2), C1–Si1–C7 115.5(2). Selected bond length [pm] and angles [°] for c): Si1–O1 164.2(3), O1–Te1 185.0(3), O2–Te2 184.7(3), O3–Te3 184.8(3), Si1–O2 164.4(3), Si1–O3 164.0(3), Si1–C1 190.6(5), O1–Si1–O2 106.22(16), O3–Si1–O1 106.84(16), O3–Si1–O2 107.97(16), O1–Si1–C1 112.25(17), O2–Si1–C1 110.71(17), O3–Si1–C1 112.53(17).

Crystals suitable for single‐crystal X‐ray diffraction could be obtained for Ph_3_SiOTeF_5_ (1f), Ph_2_Si(OTeF_5_)_2_ (2d) and *^t^* BuSi(OTeF_5_)_3_ (3c) (Figure 4). As observed in the blue‐shift of the Si–O stretching modes with increasing amount of teflate ligands at the silicon centre, the Si–O bond length decreases in the molecular structures due to the increasing Lewis acidity at the Si atom (1f: d(Si1–O1) =  1.708(2) Å, 2d: d(Si1–O1) = 1.668(4) Å, d(Si1–O2) = 1.673(4) Å, 3c: d(Si1–O1) =  1.642(3) Å, d(Si1–O2) = 1.644(3) Å, d(Si1–O3) = 1.640(3) Å. The O–Te bond length slightly increases (O1–Te1: 1f = 1.828(8) Å, 2d = 1.833(4) Å, 3c = 1.850(3) Å, O2–Te2: 2d = 1.842(3) Å, 3c = 1.847(3) Å, O3–Te3: 3c = 1.848(3) Å). Furthermore, the spatial representation at the silicon centre described by the geometry index *τ*_4_ is discussed, whereby *τ*_4_ can range from *τ*_4_ = 0.0 for a perfect square planar geometry to *τ*_4_ = 1.0 for a perfect tetrahedron [47]. Compounds 1f and 3c show a slightly distorted tetrahedral geometry with *τ*_4_ = 0.95 for 1f and *τ*_4_ = 0.96 for 3c. The distortion in compound 2d is slightly more pronounced with *τ*_4_ = 0.93.

All relevant bond length and binding parameters of Ph_3_SiOTeF_5_ (1f) are similar to Ph_3_SiOTf [11].

Furthermore, the gas phase Fluoride Ion Affinities (FIA) of all compounds have been determined on the BP86/def‐SV(P) as well as on the B3LYP/def2‐TZVPP level of theory to estimate their Lewis acidity. Therefore, the isodesmic reactions with fluorotrimethylsilane as the anchor point were used [37]. The benchmark compound and threshold for Lewis superacidity is SbF_5_ with an FIA value of 493 kJ mol^−^^1^, calculated on BP86/SV(P) [37].

All substitution pattern for the teflate compounds R_3_SiOTeF_5_ (1a–1f), R_2_Si(OTeF_5_)_2_ (2a–2d), and RSi(OTeF_5_)_3_ (3a–3d) and their fluoride adducts have been computed. To compare these results, the FIA of the silanes with further electron‐withdrawing groups like the pentafluoroethyl, triflate and bis(catecholato) group were calculated as well.

For the monosubstituted silicon teflates we faced the problem that the fluoride adducts [R_x_R′_y_SiF(OTeF_5_)]^‒^ (R = Me, Et, *^i^* Pr_3_, Ph; x = 3, y = 0; R = Pr, *^t^* Bu, R′ = Me; x = 1, y = 2; 1a‐1f) dissociated into R_x_R′_y_SiF and an [OTeF_5_]^−^ anion during structure optimization. The structures were also deemed unstable when implicit solvent model (COSMO, ε = 100) or a more sophisticated functional and basis set (B3LYP/def2‐TZVPP) were applied. Therefore, the calculation of the FIA has not been successful. Nevertheless, the fluoride adducts of the higher homologues [R_x_SiF(OTeF_5_)_4‐x_]^−^ (x = 2, 1, 0) could be calculated and the following trends are observed (Table 3). With a higher amount of coordinated teflate groups, the FIA increases and MeSi(OTeF_5_)_3_ (3a; 501 kJ mol^−^^1^), EtSi(OTeF_5_)_3_ (3b; 500 kJ mol^−^^1^), *^t^* BuSi(OTeF_5_)_3_ (3c; 489 kJ mol^−^^1^) as well as PhSi(OTeF_5_)_3_ (3d; 501 kJ mol^−^^1^) have a comparable FIA as SbF_5_. The increased Lewis acidity of the Si centre with increasing teflate content results also in shortened Si–O bonds as observed in the molecular structure in the solid state.

**TABLE 3:** Calculated fluoride ion affinities (FIAs).

		FIA [kJ mol^−1^]	
Species		BP86/def‐SV(P)	B3LYP/def2‐TZVPP
SbF_5_ [18]		493	505
*Bisubstituted silanes*			
Teflate group	Me_2_Si(OTeF_5_)_2_ (2a)	448	410
	Et_2_Si(OTeF_5_)_2_ (2b)	444	405
	*^i^* Pr_2_Si(OTeF_5_)_2_ (2c)	450	410
	Ph_2_Si(OTeF_5_)_2_ (2d)	441	410
C_2_F_5_ group	Me_2_Si(C_2_F_5_)_2_	316	342
	Ph_2_Si(C_2_F_5_)_2_ ^a^	323 / 323	349 / 332
Triflate group	Me_2_Si(OTf)_2_	420	436
	Ph_2_Si(OTf)_2_	427	433
Bis(catecholato) group	Ph_2_Si(cat^Cl^)	407	381
*Trisubstituted silanes*			
Teflate group	MeSi(OTeF_5_)_3_ (3a)	501	450
	EtSi(OTeF_5_)_3_ (3b)	500	447
	*^t^* BuSi(OTeF_5_)_3_ (3c)	489	430
	PhSi(OTeF_5_)_3_ (3d)	501	450
C_2_F_5_ group	MeSi(C_2_F_5_)_3_ ^a^	358 / 357	382 / 383
	PhSi(C_2_F_5_)_3_	370	382
Triflate group	MeSi(OTf)_3_	474	481
	PhSi(OTf)_3_	459	484
*Homoleptic silanes*			
Si(OTeF_5_)_4_		559	495
Si(C_2_F_5_)_4_		409	436
Si(OTf)_4_		496	519
Si(cat^Cl^)_2_		527	499

^a^ Two conformers of the fluoride adduct gave almost the same energy and therefore the same FIA values (see Figure S153, S154, S156 and S157).

**FIGURE 5:**
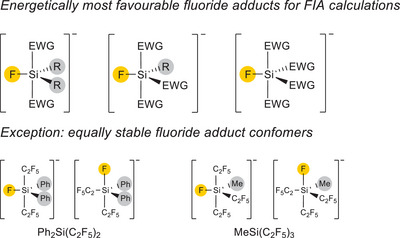
Energetical favourable conformers of the FIA adducts of [R_x_SiF(EWG)_4‐x_]^−^ (x = 2, 1, 0; EWG = teflate, C_2_F_5_, triflate, bis(catecholato)).

The calculated fluoride adducts of R_2_Si(OTeF_5_)_2_ (2a‐2d) and RSi(OTeF_5_)_3_ (3a‐3d) can exist in several conformers and our evaluation revealed that the fluoride ligand and the organic residues always occupy the equatorial position in the trigonal bipyramid (Figure 5, top).

Changing the basis set and the functional to B3LYP/def2‐TZVPP all investigated silicon‐based teflates can no longer described as Lewis superacids. This surprising behaviour is probably due to the change in the Si–F bond length. As the fluorido ligand is stronger bound to the silicon centre on the B3LYP/def2‐TZVPP level the FIA is reduced for the calculated structures.

Comparing the FIAs of the teflate compounds with those of silanes with electron‐withdrawing groups like C_2_F_5_, triflate or bis(catecholato) showed that the teflate‐based silanes [R_x_Si(OTeF_5_)_4‐x_]^−^ (x = 2, 1, 0) give the highest values (Table 3), for example PhSi(OTeF_5_)_3_ (3d; 501 kJ mol^−^^1^) vs. PhSi(C_2_F_5_)_3_ (370 kJ mol^−^^1^) vs. PhSi(OTf)_3_ (459 kJ mol^−^^1^). Again, the different conformers for the fluoride adducts have been evaluated. The fluoride ligand and the organic moieties in the triflate‐based fluorido silanes [R_x_SiF(OTf)_4‐x_]– (x = 2, 1, 0) is found to be in the equatorial position of the trigonal bipyramid. This is also true for the pentafluoroethyl‐based fluorido silanes. Only for [Ph_2_SiF(C_2_F_5_)_2_]^−^ and [MeSiF(C_2_F_5_)_3_]^−^ a second conformer equal in energy was found (see Figure 5, bottom). In those cases the fluorido ligand is in the apical position.

Natural charges obtained by NPA underline the findings of the FIA analysis. With increased Lewis acidity from R_3_SiOTeF_5_ to RSi(OTeF_5_)_3_ an increased positive charge at the Si centre (1.943 in 1f vs. 2.155 in 2d vs. 2.383 in 3c) is observed.

Unfortunately, the experimental evaluation of the Lewis acidity *via* the Gutmann–Beckett method led to several species in the ^31^P NMR spectrum and therefore was found to be inconclusive.

## 3 Conclusion

This work introduces an alternative synthetic route for the synthesis of teflate‐based silane of the formula R_3_SiOTeF_5_ (1b–1f) starting from the corresponding chlorosilanes and HOTeF_5_ or AgOTeF_5_ as teflate transfer reagents. A new synthetic procedure for the higher teflate‐substituted substances R_x_Si(OTeF_5_)_4‐x_ (x = 2, 1; 2a–2d and 3a–3d) was developed as well. The synthesized compounds were analysed *via* multinuclear NMR and IR spectroscopy. For each substitution class one representative was isolated and structurally analyzed using X‐ray diffraction. Finally, the Lewis acidity of these silanes was evaluated by their FIA and compared to those of other silanes with electron‐withdrawing groups. For all studied strong electron‐withdrawing groups (teflate, C_2_F_5_, triflate, bis(catecholato)) a higher apicophilicity is found than for fluoride.

## Conflicts of Interest

The authors declare no conflict of interest.

## Supporting information



The authors have cited additional references within the Supporting Information [48, 49, 50, 51, 52, 53, 54, 55, 56, 57, 58, 59, 60, 61, 62]. Deposition Number(s) 2407211 (Ph_3_SiOTeF_5_), 2407212 (Ph_2_SiO(TeF_5_)_2_), 2499079 (^*t*^BuSiO(TeF_5_)_3_) contain(s) the supplementary crystallographic data for this paper. These data are provided free of charge by the joint Cambridge Crystallographic Data Centre and Fachinformationszentrum Karlsruhe Access Structures service.
**Supporting File 1**: chem70610‐sup‐0001‐SuppMat.docx.

## Data Availability

The data that support the findings of this study are available from the corresponding author upon reasonable request.

## References

[1] A. M. Gressner , T. Arndt , eds., Lexikon der Medizinischen Laboratoriumsdiagnostik (Berlin, Heidelberg: Springer‐Verlag Berlin Heidelberg, 2019): 2170.

[2] Siemens & Halske, DT 1140549, 1954.

[3] Siemens & Halske, DT 1102117, 1954.

[4] E. G. Rochow , “The Direct Synthesis of Organosilicon Compounds,” Journal of the American Chemical Society 67 (1945): 963–965.

[5] S. Steinhauer , J. Bader , H.‐G. Stammler , N. Ignat'ev , and B. Hoge , “Synthesis of Tris‐ and Tetrakis(pentafluoroethyl)silanes,” Angewandte Chemie International Edition 53 (2014): 5206–5209. 24692241 10.1002/anie.201400291

[6] B. Hoge , S. Steinhauer , N. Ignatyev , and M. Schulte , DE 102012006896 A1 (2012).

[7] D. Hartmann , M. Schädler , and L. Greb , “Bis(catecholato)silanes: Assessing, Rationalizing and Increasing Silicon's Lewis Superacidity,” Chemical Science 10 (2019): 7379–7388. 31489160 10.1039/c9sc02167a PMC6713871

[8] R. Maskey , M. Schädler , C. Legler , and L. Greb , “Bis(perchlorocatecholato)silane—A Neutral Silicon Lewis Super Acid,” Angewandte Chemie International Edition 57 (2018): 1717–1720. 29240282 10.1002/anie.201712155

[9] F. S. Tschernuth , L. Bichlmaier , S. Stigler , and S. Inoue , “Tuning the Lewis Acidity of Neutral Silanes Using Perfluorinated Aryl‐ and Alkoxy Substituents”, European Journal of Inorganic Chemistry 26 (2023): e202300388.

[10] W. Uhlig , “Darstellung Neuartiger Monomerer, Oligomerer und Polymerer Silyltriflate,” Chemische Berichte 125 (1992): 47–53.

[11] A. Asadi , A. G. Avent , C. Eaborn , et al., “Reactions of a Highly Crowded Triorganotin Iodide with Silver Salts. Migration of a Methyl Group From Silicon to Tin within a Cation,” Organometallics 21 (2002): 2183–2188.

[12] W. He , X. Zheng , Y. Xiong , and Z. Zhang , CN 111217850 A, 2020.

[13] M. Schmeißer , P. Sartori , and B. Lippsmeier , “Zur Chemie der Perfluoralkansulfonsäuren,” Chemische Berichte 103 (1970): 868–879.

[14] W. Uhlig , “Zur Synthese neuartiger Triflate der 14. Gruppe,” Journal of Organometallic Chemistry 409 (1991): 377–383.

[15] S. J. Angus‐Dunne , L. E. P. Lee Chin , R. C. Burns , and G. A. Lawrance , “Metallocene and Organo‐Main Group Trifluoromethanesulfonates,” Transition Metal Chemistry 31 (2006): 268–275.

[16] S. Kuppuswamy , M. W. Holtcamp , D. M. Fiscua , M. Bedoya , L. G. McCullough , and X. Ye , WO 2018/151904 A1, 2018.

[17] A. S. Silva , D. Li , C.‐T. Lue , et al., WO 2019/246069 A1, 2019.

[18] A. Hermannsdorfer and M. Driess , “Silicon Tetrakis(trifluoromethanesulfonate): A Simple Neutral Silane Acting as a Soft and Hard Lewis Superacid,” Angewandte Chemie International Edition 60 (2021): 13656–13660. 33826216 10.1002/anie.202103414 PMC8252640

[19] M. A. Ellwanger , C. von Randow , S. Steinhauer , et al., “Tuning the Lewis Acidity of Difluorido gold(III) Complexes: The Synthesis of [AuClF_2_(SIMes)] and [AuF_2_(OTeF_5_)(SIMes)],” Chemical Communications 54 (2018): 9301–9304. 30070660 10.1039/c8cc05233f

[20] F. Sladky and H. Kropshofer , “Pentafluoro‐orthotellurates of Silicon, Germanium, and Tin,” Journal of the Chemical Society, Chemical Communications (1973): 600–601.

[21] K. F. Hoffmann , A. Wiesner , S. Steinhauer , and S. Riedel , “Insights on the Lewis Superacid Al(OTeF_5_)_3_: Solvent Adducts, Characterization and Properties,” Chemistry – A European Journal 28 (2022): e202201958. 35901430 10.1002/chem.202201958 PMC9804164

[22] D. M. Van Seggen , P. K. Hurlburt , M. D. Noirot , O. P. Anderson , and S. H. Strauss , “Tetrakis(pentafluorooxotellurato)borate(1‐): Coordinating Ability and Reactivity of a Very Large Weakly Coordinating Anion,” Inorganic Chemistry 31 (1992): 1423–1430.

[23] K. Seppelt , “Kovalente Uranverbindungen des Typs F_X_U(OTeF_5_)_6−X_ ,” Chemische Berichte 109 (1976): 1046–1052.

[24] A. Corma and H. García , “Lewis Acids: From Conventional Homogeneous to Green Homogeneous and Heterogeneous Catalysis,” Chemical Reviews 103 (2003): 4307–4365. 14611265 10.1021/cr030680z

[25] I. M. Riddlestone , A. Kraft , J. Schaefer , and I. Krossing , “Taming the Cationic Beast: Novel Developments in the Synthesis and Application of Weakly Coordinating Anions,” Angewandte Chemie International Edition 57 (2018): 13982; 29266644 10.1002/anie.201710782

[26] D. W. Stephan , “Frustrated Lewis Pairs,” Journal of the American Chemical Society 137 (2015): 10018–10032. 26214241 10.1021/jacs.5b06794

[27] L. Wickemeyer , P. C. Trapp , N. Aders , B. Neumann , H.‐G. Stammler , and N. W. Mitzel , “Reactivity of Oxygen‐Bridged Geminal Al/P and Si/P Frustrated Lewis Pairs towards Heterocumulenes,” Chemistry – A European Journal 29 (2023): e202203685. 36734185 10.1002/chem.202203685

[28] D. W. Stephan , “The Broadening Reach of Frustrated Lewis Pair Chemistry,” Science 354 (2016): aaf7229–aaf7229‐8. 27940818 10.1126/science.aaf7229

[29] G. C. Welch , R. R. San Juan , J. D. Masuda , and D. W. Stephan , “Reversible, Metal‐Free Hydrogen Activation,” Science 314 (2006): 1124–1126. 17110572 10.1126/science.1134230

[30] L. O. Müller , D. Himmel , J. Stauffer , et al., “Simple Access to the Non‐Oxidizing Lewis Superacid PhF→Al(OR^F^)_3_ (R^F^═C(CF_3_)_3_),” Angewandte Chemie International Edition 47 (2008): 7659–7663; 18767085 10.1002/anie.200800783

[31] M. A. Beckett , G. C. Strickland , J. R. Holland , and K. S. Varma , “A Convenient n.m.r. Method for the Measurement of Lewis Acidity at Boron Centres: Correlation of Reaction Rates of Lewis Acid Initiated Epoxide Polymerizations with Lewis Acidity,” Polymer 37 (1996): 4629–4631.

[32] U. Mayer , V. Gutmann , and W. Gerger , “The Acceptor Number — A Quantitative Empirical Parameter for the Electrophilic Properties of Solvents,” Monatshefte für Chemie 106 (1975): 1235–1257.

[33] B. Swanson and D. F. Shriver , “Vibrational Spectra, Vibrational Analysis, and Bonding in Acetonitrile‐Boron Trifluoride,” Inorganic Chemistry 9 (1970): 1406–1416.

[34] I. R. Beattie and T. Gilson , “430. A Normal Co‐ordinate Analysis of MeCN, BX_3_, and its Relevance to the Thermodynamic Stability of Co‐ordination Compounds,” Journal of the Chemical Society (1964): 2292–2295.

[35] L. Greb , “Lewis Superacids: Classifications, Candidates, and Applications,” Chemistry – A European Journal 24 (2018): 17881–17896. 29943864 10.1002/chem.201802698

[36] A. Wiesner , T. W. Gries , S. Steinhauer , H. Beckers , and S. Riedel , “Superacids Based on Pentafluoroorthotellurate Derivatives of Aluminium,” Angewandte Chemie International Edition 129 (2017): 8263–8266; 10.1002/anie.201702807 28558157

[37] H. Böhrer , N. Trapp , D. Himmel , M. Schleep , and I. Krossing , “From Unsuccessful H_2_‐Activation with FLPs Containing B(Ohfip)_3_ to a Systematic Evaluation of the Lewis Acidity of 33 Lewis Acids Based on Fluorine, Chloride, Hydride and Methyl Ion Affinities,” Dalton Transactions 44 (2015): 7489–7499. 25803574 10.1039/c4dt02822h

[38] R. Damerius , P. Huppmann , D. Lentz , and K. Seppelt , “Ligand Properties of the –OSeF_5_ and –OTeF_5_ Groups in Pseudo‐trigonal‐bipyramidal Molecules,” Journal of the Chemical Society, Dalton Transactions (1984): 2821–2826.

[39] J. Bader , L. Fischer , K. F. Hoffmann , et al., “On Pentafluoroorthotellurates and Related Compounds,” Chemical Reviews 125 (2025): 9140–9186. 40957838 10.1021/acs.chemrev.5c00075 PMC12512107

[40] K. Seppelt , “Stabilization of Unusual Oxidation and Coordination States by the Ligands OSF_5_, OSeF_5_, and OTeF_5_ ,” Angewandte Chemie International Edition 21 (1982): 877–888;

[41] M. Gerken , H. P. A. Mercier , and G. J. Schrobilgen in Advanced Inorganic Fluorides; Synthesis, Characterization and Applications, Eds.: T. Nakajima , B. Žemva , A. Tressaud (Lausanne: Elsevier Science S.A., 2000): 117–174.

[42] Adept Scientific, gNMR V 5.0, 2005.

[43] K. O. Christe , D. A. Dixon , J. C. P. Sanders , G. J. Schrobilgen , and W. W. Wilson , “Heptacoordination: Pentagonal Bipyramidal XeF_7_ ^+^ and TeF_7_ ^−^ Ions,” Journal of the American Chemical Society 115 (1993): 9461–9467.

[44] A. Schulz , J. Thomas , and A. Villinger , “Preparation and Characterization of [CF_3_SO_3_(SiMe_3_)_2_]^+^[B(C_6_F_5_)_4_]^−^ ,” Chemical Communications 46 (2010): 3696–3698. 20393643 10.1039/c0cc00013b

[45] S. A. Weicker and D. W. Stephan , “Activation of Carbon Dioxide by Silyl Triflate‐Based Frustrated Lewis Pairs,” Chemistry – A European Journal 21 (2015): 13027–13034. 26223404 10.1002/chem.201501904

[46] H. Yamashita , M. Hatori , M. Igarashi , and K. Sato , JP2022055604, 2020.

[47] L. Yang , D. R. Powell , and R. P. Houser , “Structural Variation in Copper(I) Complexes with Pyridylmethylamide Ligands: Structural Analysis with a New Four‐Coordinate Geometry Index, τ_4_ ,” Dalton Transactions (2007): 955–964. 17308676 10.1039/b617136b

[48] A. Engelbrecht and F. Sladky , “Pentafluoro‐orthotelluric Acid, HOTeF_5_ ,” Angewandte Chemie International Edition 3 (1964): 383–383.

[49] E. Mayer and F. Sladky , “Infrared and Raman Spectra of the TeF_5_O^−^ Anion and Evidence for Contact‐Ion‐Pair Formation in the TeF_5_OAg─CH_3_CN System. Normal‐Coordinate Analysis of the TeF_5_O^−^ and SeF_5_O^−^ Ions,” Inorganic Chemistry 14 (1975): 589–592.

[50] F. Sladky , H. Kropshofer , O. Leitzke , and P. Peringer , “Synthesis and Characterization of Compounds Containing the F_5_TeO^−^ Anion,” Journal of Inorganic and Nuclear Chemistry 28 (1976): 69–71.

[51] R. K. Harris , E. D. Becker , S. M. Cabral de Menezes , P. Granger , R. E. Hoffman , and K. W. Zilm , “Further Conventions for NMR Shielding and Chemical Shifts (IUPAC Recommendations 2008),” Magnetic Resonance in Chemistry 46 (2008): 582–598. 18407566 10.1002/mrc.2225

[52] Adept Scientific, gNMR V 5.0, 2005.

[53] G. M. Sheldrick , “A Short History of *SHELX* ,” Acta Crystallographica Section A 64 (2008): 112–122. 10.1107/S0108767307043930 18156677

[54] G. M. Sheldrick , “Crystal Structure Refinement with *SHELXL* ,” Acta Crystallographica Section C 71 (2015): 3–8. 10.1107/S2053229614024218 PMC4294323 25567568

[55] O. V. Dolomanov , L. J. Bourhis , R. J. Gildea , J. A. K. Howard , and H. Puschmann , “ *OLEX2*: A Complete Structure Solution, Refinement and Analysis Program,” Journal of Applied Crystallography 42 (2009): 339–341.

[56] TURBOMOLE GmbH, *TURBOMOLE V7.6. a development of University of Karlsruhe and Forschungszentrum Karlsruhe GmbH* , 2021.

[57] A. D. Becke , “Density‐Functional Exchange‐Energy Approximation with Correct Asymptotic Behavior,” Physical Review A 38 (1988): 3098–3100. 10.1103/physreva.38.3098 9900728

[58] C. Lee , W. Yang , and R. G. Parr , “Development of the Colle‐Salvetti Correlation‐Energy Formula into a Functional of the Electron Density,” Physical Review B 37 (1988): 785–789. 10.1103/physrevb.37.785 9944570

[59] S. H. Vosko , L. Wilk , and M. Nusair , “Accurate Spin‐Dependent Electron Liquid Correlation Energies for Local Spin Density Calculations: A Critical Analysis,” Canadian Journal of Physics 58 (1980): 1200–1211.

[60] M. Sierka , A. Hogekamp , and R. Ahlrichs , “Fast Evaluation of the Coulomb Potential for Electron Densities Using Multipole Accelerated Resolution of Identity Approximation,” The Journal of Chemical Physics 118 (2003): 9136–9148.

[61] F. Weigend and R. Ahlrichs , “Balanced Basis Sets of Split Valence, Triple Zeta Valence and Quadruple Zeta Valence Quality for H to Rn: Design and Assessment of Accuracy,” Physical Chemistry Chemical Physics 7 (2005): 3297–3305. 16240044 10.1039/b508541a

[62] P. Deglmann , F. Furche , and R. Ahlrichs , “An Efficient Implementation of Second Analytical Derivatives for Density Functional Methods,” Chemical Physics Letters 362 (2002): 511–518.

