# Supplementary material for: Simple Access to a Series of Higher Substituted Pentafluoroorthotellurate‐Based Silanes
Source: Chemistry. 2026 Jan 27;32(14):e03448. doi: 10.1002/chem.202503448 (PMC13088018; doi:10.1002/chem.202503448)
Supplement: Supplementary file 1 — The authors have cited additional references within the Supporting Information [48, 49, 50, 51, 52, 53, 54, 55, 56, 57, 58, 59, 60, 61, 62]. Deposition Number(s) 2407211 (Ph3SiOTeF5), 2407212 (Ph2SiO(TeF5)2), 2499079 ( t BuSiO(TeF5)3) contain(s) the supplementary crystallographic data for this paper. These data are provided free of charge by the joint Cambridge Crystallographic Data Centre and Fachinformationszentrum Karlsruhe Access Structures service. Supporting File 1: chem70610‐sup‐0001‐SuppMat.docx. [file CHEM-32-e03448-s001.docx]

Table of Content

[1 Experimental section 3](#_Toc215824999)

[_1.1_ General procedures and materials 3](#_Toc215825000)

[_1.2_ Synthesis of Me_3_SiOTeF_5_ with HOTeF_5_ (1a) 4](#_Toc215825001)

[_1.3_ Synthesis of Et_3_SiOTeF_5_ with HOTeF_5_ (1b) 4](#_Toc215825002)

[_1.4_ Formation of Et_3_SiOTeF_5_ with AgOTeF_5_ (1b) 4](#_Toc215825003)

[_1.5_ Synthesis of Me_2_PrSiOTeF_5_ with HOTeF_5_ (1c) 4](#_Toc215825004)

[_1.6_ Formation of Me_2_PrSiOTeF_5_ with AgOTeF_5_ (1c) 5](#_Toc215825005)

[_1.7_ Synthesis of *^i^*Pr_3_SiOTeF_5_ with HOTeF_5_ (1d) 5](#_Toc215825006)

[_1.8_ Formation of *^i^*Pr_3_SiOTeF_5_ with AgOTeF_5_ (1d) 5](#_Toc215825007)

[_1.9_ Synthesis of *^t^*BuMe_2_SiOTeF_5_ with HOTeF_5_ (1e) 6](#_Toc215825008)

[_1.10_ Formation of *^t^*BuMe_2_SiOTeF_5_ with AgOTeF_5_ (1e) 6](#_Toc215825009)

[_1.11_ Synthesis of Ph_3_SiOTeF_5_ with HOTeF_5_ (1f) 6](#_Toc215825010)

[_1.12_ Synthesis of Ph_3_SiOTeF_5_ with AgOTeF_5_ (1f) 7](#_Toc215825011)

[_1.13_ Formation of Me_2_Si(OTeF_5_)_2_ (2a) 7](#_Toc215825012)

[_1.14_ Formation of Et_2_Si(OTeF_5_)_2_ (2b) 7](#_Toc215825013)

[_1.15_ Formation of *^i^*Pr_2_Si(OTeF_5_)_2_ (2c) 8](#_Toc215825014)

[_1.16_ Synthesis of Ph_2_Si(OTeF_5_)_2_ (2d) 8](#_Toc215825015)

[_1.17_ Formation of MeSi(OTeF_5_)_3_ (3a) 8](#_Toc215825016)

[_1.18_ Formation of EtSi(OTeF_5_)_3_ (3b) 9](#_Toc215825017)

[_1.19_ Synthesis of *^t^*BuSi(OTeF_5_)_3_ (3c) 9](#_Toc215825018)

[_1.20_ Formation of PhSi(OTeF_5_)_3_ (3d) 9](#_Toc215825019)

[2 NMR Spectra 10](#_Toc215825020)

[_2.1_ NMR spectra of Me_3_SiOTeF_5_ with HOTeF_5_ (1a) 10](#_Toc215825021)

[_2.2_ NMR spectra of Et_3_SiOTeF_5_ with HOTeF_5_ (1b) 13](#_Toc215825022)

[_2.3_ NMR spectra of Et_3_SiOTeF_5_ with AgOTeF_5_ (1b) 15](#_Toc215825023)

[_2.4_ NMR spectra of Me_2_PrSiOTeF_5_ with HOTeF_5_ (1c) 18](#_Toc215825024)

[_2.5_ NMR spectra of Me_2_PrSiOTeF_5_ with AgOTeF_5_ (1c) 21](#_Toc215825025)

[_2.6_ NMR spectra of *^i^*Pr_3_SiOTeF_5_ with HOTeF_5_ (1d) 24](#_Toc215825026)

[_2.7_ NMR spectra of *^i^*Pr_3_SiOTeF_5_ with AgOTeF_5_ (1d) 27](#_Toc215825027)

[_2.8_ NMR spectra of *^t^*BuMe_2_SiOTeF_5_ with HOTeF_5_ (1e) 29](#_Toc215825028)

[_2.9_ NMR spectra of *^t^*BuMe_2_SiOTeF_5_ with AgOTeF_5_ (1e) 32](#_Toc215825029)

[_2.10_ NMR spectra of Ph_3_SiOTeF_5_ with HOTeF_5_ (1f) 34](#_Toc215825030)

[_2.11_ NMR spectra of Ph_3_SiOTeF_5_ with AgOTeF_5_ (1f) 37](#_Toc215825031)

[_2.12_ NMR spectra of Me_2_Si(OTeF_5_)_2_ (2a) 40](#_Toc215825032)

[_2.13_ NMR spectra of Et_2_Si(OTeF_5_)_2_ (2b) 42](#_Toc215825033)

[_2.14_ NMR spectra of *^i^*Pr_2_Si(OTeF_5_)_2_ (2c) 45](#_Toc215825034)

[_2.15_ NMR spectra of Ph_2_Si(OTeF_5_)_2_ (2d) 48](#_Toc215825035)

[_2.16_ NMR spectra of MeSi(OTeF_5_)_3_ (3a) 50](#_Toc215825036)

[_2.17_ NMR spectra of EtSi(OTeF_5_)_3_ (3b) 53](#_Toc215825037)

[_2.18_ NMR spectra of *^t^*BuSi(OTeF_5_)_3_ (3c) 56](#_Toc215825038)

[_2.19_ NMR spectra of PhSi(OTeF_5_)_3_ (3d) 58](#_Toc215825039)

[3 Vibrational Spectra 60](#_Toc215825040)

[_3.1_ Infrared spectrum of Ph_3_SiOTeF_5_ with AgOTeF_5_ (1f) 60](#_Toc215825041)

[_3.2_ Infrared spectrum of Ph_2_Si(OTeF_5_)_2_ (2d) 60](#_Toc215825042)

[_3.3_ Infrared spectrum of *^t^*BuSi(OTeF_5_)_3_ (3c) 61](#_Toc215825043)

[_3.4_ Raman spectrum of Ph_3_SiOTeF_5_ (1f) 61](#_Toc215825044)

[_3.5_ Raman spectrum of Ph_2_Si(OTeF_5_)_2_ (2d) 62](#_Toc215825045)

[_3.6_ Raman spectrum of *^t^*BuSi(OTeF_5_)_3_ in CH_2_Cl_2_ (3c) 62](#_Toc215825046)

[4 Crystal data 63](#_Toc215825047)

[5 Quantum-chemical calculations 65](#_Toc215825048)

[_5.1_ Me_3_SiOTeF_5_ 65](#_Toc215825049)

[_5.2_ Et_3_SiOTeF_5_ 66](#_Toc215825050)

[_5.3_ Me_2_PrSiOTeF_5_ 67](#_Toc215825051)

[_5.4_ *^i^*Pr_3_SiOTeF_5_ 68](#_Toc215825052)

[_5.5_ *^t^*BuMe_2_SiOTeF_5_ 69](#_Toc215825053)

[_5.6_ Ph_3_SiOTeF_5_ 70](#_Toc215825054)

[_5.7_ Me_2_Si(OTeF_5_)_2_ 71](#_Toc215825055)

[_5.8_ [Me_2_Si(OTeF_5_)_2_F]^–^ 71](#_Toc215825056)

[_5.9_ Et_2_Si(OTeF_5_)_2_ 72](#_Toc215825057)

[_5.10_ [Et_2_Si(OTeF_5_)_2_F]^–^ 73](#_Toc215825058)

[_5.11_ *^i^*Pr_2_Si(OTeF_5_)_2_ 74](#_Toc215825059)

[_5.12_ [*^i^*Pr_2_Si(OTeF_5_)_2_F]^–^ 75](#_Toc215825060)

[_5.13_ Ph_2_Si(OTeF_5_)_2_ 76](#_Toc215825061)

[_5.14_ [Ph_2_Si(OTeF_5_)_2_F]^–^ 77](#_Toc215825062)

[_5.15_ MeSi(OTeF_5_)_3_ 78](#_Toc215825063)

[_5.16_ [MeSi(OTeF_5_)_3_F]^–^ 78](#_Toc215825064)

[_5.17_ EtSi(OTeF_5_)_3_ 79](#_Toc215825065)

[_5.18_ [EtSi(OTeF_5_)_3_F]^–^ 80](#_Toc215825066)

[_5.19_ *^t^*BuSi(OTeF_5_)_3_ 81](#_Toc215825067)

[_5.20_ [*^t^*BuSi(OTeF_5_)_3_F]^–^ 82](#_Toc215825068)

[_5.21_ PhSi(OTeF_5_)_3_ 83](#_Toc215825069)

[_5.22_ [PhSi(OTeF_5_)_3_F]^–^ 84](#_Toc215825070)

[_5.23_ Si(OTeF_5_)_4_ 85](#_Toc215825071)

[_5.24_ [Si(OTeF_5_)_4_F]^–^ 86](#_Toc215825072)

[_5.25_ Me_2_Si(SO_3_CF_3_)_2_ 87](#_Toc215825073)

[_5.26_ [Me_2_Si(SO_3_CF_3_)_2_F]^–^ 87](#_Toc215825074)

[_5.27_ Ph_2_Si(SO_3_CF_3_)_2_ 88](#_Toc215825075)

[_5.28_ [Ph_2_Si(SO_3_CF_3_)_2_F]^–^ 89](#_Toc215825076)

[_5.29_ MeSi(SO_3_CF_3_)_3_ 90](#_Toc215825077)

[_5.30_ [MeSi(SO_3_CF_3_)_3_F]^–^ 91](#_Toc215825078)

[_5.31_ PhSi(SO_3_CF_3_)_3_ 92](#_Toc215825079)

[_5.32_ [PhSi(SO_3_CF_3_)_3_F]^–^ 93](#_Toc215825080)

[_5.33_ Si(SO_3_CF_3_)_4_ 94](#_Toc215825081)

[_5.34_ [Si(SO_3_CF_3_)_4_F]^–^ 95](#_Toc215825082)

[_5.35_ Me_2_Si(C_2_F_5_)_2_ 96](#_Toc215825083)

[_5.36_ [Me_2_Si(C_2_F_5_)_2_F]^–^ 96](#_Toc215825084)

[_5.37_ Ph_2_Si(C_2_F_5_)_2_ 97](#_Toc215825085)

[_5.38_ [Ph_2_Si(C_2_F_5_)_2_F]^–^ 98](#_Toc215825086)

[_5.39_ MeSi(C_2_F_5_)_3_ 100](#_Toc215825087)

[_5.40_ [MeSi(C_2_F_5_)_3_F]^–^ 100](#_Toc215825088)

[_5.41_ PhSi(C_2_F_5_)_3_ 102](#_Toc215825089)

[_5.42_ [PhSi(C_2_F_5_)_3_F]^–^ 103](#_Toc215825090)

[_5.43_ Si(C_2_F_5_)_4_ 104](#_Toc215825091)

[_5.44_ [Si(C_2_F_5_)_4_F]^–^ 105](#_Toc215825092)

[_5.45_ Ph_2_Si(cat^Cl^) 106](#_Toc215825093)

[_5.46_ [Ph_2_Si(cat^Cl^)F]^–^ 107](#_Toc215825094)

[_5.47_ Si(cat^Cl^)_2_ 108](#_Toc215825095)

[_5.48_ [Si(cat^Cl^)_2_F]^–^ 109](#_Toc215825096)

[_5.49_ Me_3_Si^+^ 109](#_Toc215825097)

[_5.50_ Me_3_SiF 109](#_Toc215825098)

[6 References 111](#_Toc215825099)

# Experimental section

## General procedures and materials

All experiments were carried out under exclusion of moisture and oxygen using standard Schlenk techniques. Triboflon III was used to grease the glassware. Before use solvents were dried with CaH_2_ (*o*-DFB, CD_2_Cl_2_) or using a MBraun SPS-800 solvent system (CH_2_Cl_2_) and stored on 3 and 4 Å molecular sieves. Solids were handled inside a glovebox with an atmosphere of dry argon (O_2_<0.5 ppm, H_2_O<0.5 ppm). The synthesis of the pentafluoroorthotelluric acid HOTeF_5_^[1]^ and AgOTeF_5_^[2]^ were performed as described elsewhere. All other reagents were purchased commercially and used as received. NMR spectra were measured on a JEOL 400 MHz ECS or ECZ spectrometer. All reported chemical shifts (*δ* in ppm) were referenced to Ξ values given in IUPAC recommendations of 2008 using the ^2^H signal of the deuterated solvent as internal reference.^[3]^ For external locking acetone-d6 was flame sealed in a glass capillary and the lock oscillator frequency was adjusted to give *δ*(1H) = 7.26 ppm for a CHCl_3_ sample locked on the capillary. Chemical shifts and coupling constants of ^19^F NMR spectra are given as simulated by *gNMR 5.0*.^[4]^ Multiplicity is designated as followed: d = dublet, dquin = dublet from quintets, s = singulet, sept = septet, m = multiplet. IR spectra were recorded on a Bruker ALPHA FTIR spectrometer equipped with a diamond ATR at room temperature inside the glovebox under argon atmosphere (range 4000-550 cm^–1^, resolution 4 cm^–1^, 64 scans). The Bruker OPUS 7.5 software package was used to process the collected data. Raman spectra were measured on a Bruker MultiRAM II with a low-temperature Ge detector (1064 nm, 50-100 mW, resolution 4 cm^–1^). Single crystal X-ray diffraction was performed on a Bruker D8 Venture diffractometer with a CMOS area detector employing MoKα radiation. Single crystals were selected at –40 °C in perfluoroether oil under nitrogen atmosphere and mounted on a 0.15 nm MiTeGen MicroMount. The crystal structures were solved with the *ShelXT*^[5]^ structure solution programme and refined with *ShelXL*^[6]^ refinement package by full-matrix least-squares methods against F2 by OLEX2.^[7]^ Deposition Number(s) contain(s) the supplementary crystallographic data for this paper which are provided free of charge by the joint Cambridge Crystallographic Data Centre and Fachinformationszentrum Karlsruhe Access Structures service. In Table S1 crystal data and other details of the structure analyses are summarized. The Diamond V4.6.4 software was used for visualization. Quantum-chemical calculations were performed using the *Turbomole V7.6.1* software.^[8]^ Geometry optimizations for all investigated molecular structures were done in redundant internal coordinates using the DFT functionals BP86 or B3LYP^[9]^ with the resolution-of-identity (RI)^[10]^ approximation together with the basis set def-TZVP or def2-TZVPP.^[11]^ All optimized structures were checked for minima and imaginary frequencies with the integrated EIGER and AOFORCE^[12]^ modules. With the FREEH module thermal and entropic contributions to the reaction energies were calculated for standard conditions (298.15 K, 0.1 MPa) at the RI-BP86(D3BJ)/def-TZVP and RI-B3LYP(D3BJ)/def2-TZVPP level. IR and Raman spectra were simulated without scaling factor and a FWHM of 10 cm^–1^.

## Synthesis of Me_3_SiOTeF_5_ with HOTeF_5_ (1a)

Me_3_SiOTeF_5_ was synthesized according to a literature known procedure.^[13]^

^1^H NMR (401 MHz, CH_2_Cl_2_, external lock acetone-d6, 20 °C): *δ*=0.47 (s, 9H, CH_3_) ppm.

^13^C NMR (100 MHz, CH_2_Cl_2_, external lock acetone-d6, 20 °C): *δ*=0.3 (Si-CH_3_) ppm.

^19^F NMR spectrum (377 MHz, CH_2_Cl_2_, external lock acetone-d6, 20 °C) *δ*=–39.8 (**A**B_4_X, 1F, ^1^*J*(^19^F,^125^Te)=3420 Hz, ^2^*J*(^19^F,^19^F)=186 Hz), –43.8 (A**B**_4_X, 4F, ^1^*J*(^19^F,^125^Te)=3556 Hz, ^2^*J*(^19^F,^19^F)=186 Hz) ppm.

^29^Si DEPT NMR (79 MHz, CH_2_Cl_2_, external lock acetone-d6, 20 °C): *δ*=39.2 (s) ppm.

^125^Te NMR (126 MHz, CH_2_Cl_2_, external lock acetone-d6, 20 °C): *δ*=561 (dquin, ^1^*J*(^19^F,^125^Te)=3420 Hz, ^1^*J*(^19^F,^125^Te)=3556 Hz) ppm.

## Synthesis of Et_3_SiOTeF_5_ with HOTeF_5_ (1b)

At –78 °C Et_3_SiCl (201 mg, 1.33 mmol) was added to HOTeF_5_ (324 mg, 1.35 mmol). The reaction mixture was warmed up to –30 °C and stirred for 4 days. The removal of HCl under reduced pressure at –50 °C was tracked by the IR spectrum of the gas phase. A colourless liquid was obtained and characterized as Et_3_SiOTeF_5_ (463 mg, 1.31 mmol, 97%).

^1^H NMR (401 MHz, neat, external lock acetone-d6, 17 °C): *δ*=1.10 (A_3_B_2_, m, 3H, CH_3_, ^3^*J*(^1^H,^1^H)≈8.2 Hz), 0.89 (A_3_B_2_, m, 2H, Si-CH_2_, ^3^*J*(^1^H,^1^H)≈8.2 Hz) ppm.

^13^C NMR (100 MHz, neat, external lock acetone-d6, 20 °C): *δ*=5.5 (Si-CH_2_), 4.8 (CH_3_) ppm.

^19^F NMR (377 MHz, neat, external lock acetone-d6, 17 °C): *δ*=–40.4 (**A**B_4_X, 1F, ^1^*J*(^19^F,^125^Te)=3428 Hz, ^2^*J*(^19^F,^19^F)=187 Hz), –44.6 (A**B**_4_X, 4F, ^1^*J*(^19^F,^125^Te)=3571 Hz, ^2^*J*(^19^F,^19^F)=187 Hz) ppm.

^29^Si DEPT NMR (79 MHz, neat, external lock acetone-d6, 20 °C): *δ*=38.3 (s, Et_3_SiCl), 39.5 (s, ^2^*J*(^125^Te,^29^Si)=138 Hz, ^2^*J*(^1^H,^29^Si)=5.3 Hz) ppm.

## Formation of Et_3_SiOTeF_5_ with AgOTeF_5_ (1b)

A solution of Et_3_SiCl (95 mg, 0.63 mmol) in 0.3 mL of CH_2_Cl_2_ was added at r.t. to a suspension of AgOTeF_5_ (219 mg, 0.63 mmol) in 0.7 mL of CH_2_Cl_2_. The mixture was stirred for 24 hours at r.t. A white solid precipitated. The supernatant was isolated as colourless liquid with a PFA transfer canula and characterized as Et_3_SiOTeF_5_ (99% NMR yield).

^1^H NMR (401 MHz, CH_2_Cl_2_, external lock acetone-d6, 18 °C): *δ*=1.03 (m, 3H, CH_3_), 0.85 (m, 2H, Si-CH_2_) ppm.

^13^C NMR (101 MHz, CH_2_Cl_2_, external lock acetone-d6, 18 °C): *δ*=5.8 (Si-CH_2_, ^2^*J*(^125^Te,^13^C)=18 Hz), 5.5 (CH_3_) ppm.

^19^F NMR (377 MHz, CH_2_Cl_2_, external lock acetone-d6, 18 °C): *δ*=–39.2 (**A**B_4_X, 1F, ^1^*J*(^19^F,^125^Te)=3424 Hz, ^2^*J*(^19^F,^19^F)=188 Hz), –43.9 (A**B**_4_X, 4F, ^1^*J*(^19^F,^125^Te)=3581 Hz, ^2^*J*(^19^F,^19^F)=188 Hz) ppm.

^29^Si DEPT NMR (80 MHz, CH_2_Cl_2_, external lock acetone-d6, 17 °C): *δ*=40.6 ppm.

^125^Te NMR (126 MHz, CH_2_Cl_2_, external lock acetone-d6, 19 °C): *δ*=558 (dquin, ^1^*J*(^19^F,^125^Te)=3424 Hz, ^1^*J*(^19^F,^125^Te)=3581 Hz) ppm.

## Synthesis of Me_2_PrSiOTeF_5_ with HOTeF_5_ (1c)

At –78 °C Me_2_PrSiCl (350 mg, 2.56 mmol) was added to HOTeF_5_ (636 mg, 2.65 mmol). The reaction mixture was warmed up to –30 °C and stirred for 2 hours. The removal of HCl under reduced pressure at –50 °C was tracked by the IR spectrum of the gas phase. A colourless liquid was obtained and characterized as Me_2_PrSiOTeF_5_ (830 mg, 2.44 mmol, 92%).

^1^H NMR (401 MHz, neat, external lock acetone-d6, 17 °C): *δ*=1.53 (m, 2H, CH_2_**CH_2_**CH_3_), 1.07 (m, 3H, CH_2_CH_2_**CH_3_**), 0.87 (m, 2H, Si–CH_2_), 0.41 (s, 6H, Si–CH_3_) ppm.

^13^C NMR (101 MHz, neat, external lock acetone-d6, 17 °C): *δ*=19.5 (Si-CH_2_, ^1^*J*(^29^Si,^13^C)=61 Hz, ^2^*J*(^125^Te,^13^C)=26 Hz), 16.9 (CH_2_**CH_2_**CH_3_, ^2^*J*(^29^Si,^13^C)=8 Hz), 15.7 (CH_2_CH_2_**CH_3_**), –1.6 (Si‒CH_3_, ^1^*J*(^29^Si,^13^C)=58 Hz, ^2^*J*(^125^Te,^13^C)=13 Hz) ppm.

^19^F NMR (377 MHz, neat, external lock acetone-d6, 17 °C): *δ*=–40.2 (**A**B_4_X, 1F, ^1^*J*(^19^F,^125^Te)=3427 Hz, ^2^*J*(^19^F,^19^F)=186 Hz), –44.1 (A**B_4_**X, 4F, ^1^*J*(^19^F,^125^Te)=3568 Hz, ^2^*J*(^19^F, ^19^F)=186 Hz) ppm.

^29^Si DEPT NMR (80 MHz, neat, external lock acetone-d6, 17 °C): *δ*=38.1 (s, ^2^*J*(^125^Te,^29^Si)=123 Hz, ^1^*J*(^13^C, ^29^Si)=59 Hz) ppm.

^125^Te NMR (126 MHz, neat, external lock acetone-d6, 17 °C): *δ*=560 (dquin, ^1^*J*(^125^Te,^19^F_ax_)=3427 Hz, ^1^*J*(^125^Te,^19^F_eq_=3568 Hz) ppm.

## Formation of Me_2_PrSiOTeF_5_ with AgOTeF_5_ (1c)

A solution of Me_2_PrSiCl (98 mg, 0.72 mmol) in 0.3 mL of CH_2_Cl_2_ was added at r.t. to a suspension of AgOTeF_5_ (250 mg, 0.72 mmol) in 0.7 mL of CH_2_Cl_2_. The mixture was stirred for 20 hours at r.t. A white solid precipitated. All volatiles were separated from the residue by condensation as colourless liquid (99% NMR yield).

^1^H NMR (401 MHz, CH_2_Cl_2_, external lock acetone-d6, 18 °C): *δ*=1.43 (m, 2H, CH_2_**CH_2_**CH_3_), 1.00 (m, 3H, CH_2_CH_2_**CH_3_**), 0.82 (m 2H, Si–CH_2_), 0.37 (s, 6H, Si–CH_3_) ppm.

^13^C NMR (101 MHz, CH_2_Cl_2_, external lock acetone-d6, 17 °C): *δ*=19.7 (Si-CH_2_, ^2^*J*(^125^Te,^13^C) =26 Hz), 17.4 (CH_2_**CH_2_**CH_3_), 15.9 (CH_2_CH_2_**CH_3_**), –0.6 (Si‒CH_3_, ^1^*J*(^29^Si,^13^C)=58 Hz).

^19^F NMR (377 MHz, CH_2_Cl_2_, external lock acetone-d6, 18 °C): *δ*=–39.0 (**A**B_4_X, 1F, ^1^*J*(^19^F,^125^Te)=3423 Hz, ^2^*J*(^19^F,^19^F)=187 Hz), –43.2 (A**B**_4_X, 4F, ^1^*J*(^19^F,^125^Te)=3577 Hz, ^2^*J*(^19^F,^19^F)=187 Hz) ppm.

^29^Si DEPT NMR (80 MHz, CH_2_Cl_2_, external lock acetone-d6, 18 °C): *δ*=39.4 ppm.

## Synthesis of *^i^*Pr_3_SiOTeF_5_ with HOTeF_5_ (1d)

At –78 °C *^i^*Pr_3_SiCl (516 g, 2.68 mmol) was added to HOTeF_5_ (678 g, 2.82 mmol). The reaction mixture was warmed up to –30 °C and stirred for 22 hours. The removal of HCl under reduced pressure at –50 °C was tracked by the IR spectrum of the gas phase. A colourless liquid was obtained and characterized as *^i^*Pr_3_SiOTeF_5_ (1.12 g, 2.82 mmol, 99%).

^1^H NMR (401 MHz, neat, external lock acetone-d6, 17 °C): *δ*=1.24 (m, 3H, Si–CH), 1.09 (d, 18H, CH_3_) ppm.

^13^C NMR (101 MHz, neat, external lock acetone-d6, 17 °C): *δ*=16.8 (Si–CH), 13.6 (CH(**CH_3_**)_2,_ ^2^*J*(^125^Te,^13^C)=57 Hz, ^1^*J(*^125^Te,^13^C)=31 Hz) ppm.

^19^F NMR (377 MHz, neat, external lock acetone-d6, 18 °C): *δ*=–43.7 (**A**B_4_X, 1F, ^1^*J*(^19^F,^125^Te)=3485 Hz, ^2^*J*(^19^F,^19^F)=182 Hz), –46.7 (A**B**_4_X, 4F, ^1^*J*(^19^F,^125^Te)=3607 Hz, ^2^*J*(^19^F, ^19^F)=182 Hz) ppm.

^29^Si DEPT NMR (80 MHz, neat, external lock acetone-d6, 17 °C): *δ*=39.1 ppm.

^125^Te NMR (126 MHz, neat, external lock acetone-d6, 17 °C): *δ*=602 (dquin, ^1^*J*(^19^F,^125^Te)=3485 Hz, ^1^*J*(^19^F,^125^Te)=3607 Hz) ppm.

## Formation of *^i^*Pr_3_SiOTeF_5_ with AgOTeF_5_ (1d)

A solution of *^i^*Pr_3_SiCl (132 mg, 0.69 mmol) in 0.3 mL of CH_2_Cl_2_ was added at r.t. to a suspension of AgOTeF_5_ (239 mg, 0.69 mmol) in 0.4 mL of CH_2_Cl_2_. The mixture was stirred for 24 hours at r.t. A white solid precipitated. The supernatant was isolated as colourless liquid with a PFA transfer canula (92% NMR yield).

^13^C NMR (101 MHz, CH_2_Cl_2_, external lock acetone-d6, 18 °C): *δ*=16.7 (CH(**CH_3_**)_2_), 13.3 (^1^*J*(^29^Si,^13^C)=59 Hz, ^2^*J*(^125^Te,^13^C)=16 Hz, Si–CH_2_) ppm.

^19^F NMR (376 MHz, CH_2_Cl_2_, external lock acetone-d6, 18 °C): *δ*=–38.7 (**A**B_4_X, 1F, ^1^*J*(^19^F,^125^Te)=3442 Hz, ^2^*J*(^19^F,^19^F)=190 Hz), –43.8 (A**B**_4_X, 4F, ^1^*J*(^19^F,^125^Te)=3586 Hz, ^2^*J*(^19^F,^19^F)=190 Hz) ppm.

^29^Si DEPT NMR (80 MHz, CH_2_Cl_2_, external lock acetone-d6, 18 °C): *δ*=36.2 (s, *^i^*Pr_3_SiCl), 35.8 (quin, ^2^*J*(^19^F,^29^Si)=4 Hz) ppm.

^125^Te NMR (126 MHz, CH_2_Cl_2_, external lock acetone-d6, 19 °C): *δ*=547 (dquin, ^1^*J*(^19^F,^125^Te)=3442 Hz, ^1^*J*(^19^F,^125^Te)=3586 Hz) ppm.

## Synthesis of *^t^*BuMe_2_SiOTeF_5_ with HOTeF_5_ (1e)

At –78 °C *^t^*BuMe_2_SiCl (407 mg, 2.70 mmol) was added to HOTeF_5_ (645 mg, 2.69 mmol). The reaction mixture was warmed up to –30 °C and stirred for 3 hours. The removal of HCl under reduced pressure at –50 °C was tracked by the IR spectrum of the gas phase. A colourless liquid was obtained and characterized as *^t^*BuMe_2_SiOTeF_5_ (807 mg, 2.28 mmol, 85%).

^1^H NMR (401 MHz, neat, external lock acetone-d6, 17 °C): *δ*=1.06 (s, 9H, Si–C(CH_3_)_3_), 0.43 (s, 6H, Si-CH_3_) ppm.

^13^C NMR (101 MHz, neat, external lock acetone-d6, 17 °C): *δ*=24.7 (Si–C(**C**H_3_)_3_), 18.7 (Si–**C**(CH_3_)_3_), –2.4 (Si–CH_3_, ^1^*J*(^29^Si,^13^C)=56 Hz) ppm.

^19^F NMR (377 MHz, neat, external lock acetone-d6, 16 °C): *δ*=–43.4 (**A**B_4_X, 1F, ^1^*J*(^19^F,^125^Te)=3466 Hz, ^2^*J*(^19^F,^19^F)=187 Hz), –46.5 (A**B**_4_X, 4F, ^1^*J*(^19^F,^125^Te)=3599 Hz, ^2^*J*(^19^F,^19^F)=187 Hz) ppm.

^29^Si DEPT NMR (80 MHz, neat, external lock acetone-d6, 17 °C): *δ*=38.5 (s, ^1^*J*(^29^Si,^13^C)=60 Hz) ppm.

## Formation of *^t^*BuMe_2_SiOTeF_5_ with AgOTeF_5_ (1e)

AgOTeF_5_ (232 mg, 0.67 mmol) was dissolved in 1.3 mL of CH_2_Cl_2_ and *^t^*BuMe_2_SiCl (104 mg, 0.69 mmol) was added. The suspension was stirred for 24 hours at r.t. A white solid precipitated. All volatiles were separated from the residue by condensation as colourless liquid (99% NMR yield).

^1^H NMR (401 MHz, CH_2_Cl_2_, external lock acetone-d6, 17 °C): *δ*=0.97 (m, 9H, Si–C(CH_3_)_3_), 0.34 (6H, Si-CH_3_) ppm.

^13^C NMR (101 MHz, CH_2_Cl_2_, external lock acetone-d6, 17 °C): *δ*=24.8 (Si–C(**C**H_3_)_3_), 19.3 (Si–**C**(CH_3_)_3_), –3.7 (Si–CH_3_, ^1^*J*(^29^Si,^13^C)=56 Hz) ppm.

^19^F NMR (377 MHz, CH_2_Cl_2_, external lock acetone-d6, 19 °C): *δ*=–39.1 (**A**B_4_X, 1F, ^1^*J*(^19^F,^125^Te)=3428 Hz, ^2^*J*(^19^F,^19^F)=187 Hz), –43.6 (A**B**_4_X, 4F, ^1^*J*(^19^F,^125^Te)=3583 Hz, ^2^*J*(^19^F,^19^F)=187 Hz) ppm.

^29^Si DEPT NMR (80 MHz, CH_2_Cl_2_, external lock acetone-d6, 17 °C): *δ*=39.7 ppm.

## Synthesis of Ph_3_SiOTeF_5_ with HOTeF_5_ (1f)

At –78 °C Ph_3_SiCl (385 mg, 1.31 mmol) was added to HOTeF_5_ (313 mg, 1.31 mmol). The reaction mixture was warmed up to –30 °C and stirred for 1 hour. To the mixture 0.5 mL of CH_2_Cl_2_ was added and it was stirred overnight at r.t. The removal of HCl under reduced pressure at –50 °C was tracked by the IR spectrum of the gas phase. Crystals suitable for sc-XRD were grown within 11 days by cooling the reaction solution slowly to –40 °C (99% NMR yield).

^1^H NMR (401 MHz, neat, CD_2_Cl_2_, 18 °C): δ=7.30-7.80 (m, 5H, CH_arom._) ppm.

^13^C NMR (101 MHz, neat, CD_2_Cl_2_, 18 °C): δ=135.3 (*ortho*-C), 131.5 (*para*-C), 131.0 (*ipso*-C*),* 128.3 (*meta*-C) ppm.

^19^F NMR (377 MHz, neat, external lock acetone-d6, 16 °C): *δ*=–41.7 (**A**B_4_X, 1F, ^1^*J*(^19^F,^125^Te)=3476 Hz, ^2^*J*(^19^F,^19^F)=189 Hz), –43.3 (A**B**_4_X, 4F, ^1^*J*(^19^F,^125^Te)=3612 Hz, ^2^*J*(^19^F,^19^F)=189 Hz) ppm.

^29^Si DEPT NMR (80 MHz, neat, CD_2_Cl_2_, 18 °C): *δ*=–1.1 ppm.

IR (ATR, 25 °C): $\tilde{\nu}$=1591 (w), 1487 (w, C–H), 1430 (m), 1191 (w), 1119 (m, Si–C), 1028 (w), 997 (w), 904 (m, Si–O), 743 (w), 714 (s), 692 (s, Te–F), 619 (w), 583 (w), 543 (m), 502 (s), 439 (w).

## Synthesis of Ph_3_SiOTeF_5_ with AgOTeF_5_ (1f)

AgOTeF_5_ (201 mg, 0.58 mmol) was dissolved in 0.8 mL of *o*-DFB and Ph_3_SiCl (170 mg, 0.58 mmol) was added. The suspension was stirred for 22 hours at r.t. A white solid precipitated. The supernatant was isolated with a glass filter PFA transfer canula and cooled to –40 °C. A colourless solid (77 mg, 0.55 mmol, 27%) was obtained. Crystals suitable for sc-XRD were grown within 12 days by cooling the reaction solution slowly to –40 °C.

^1^H NMR (401 MHz, *o*-DFB, CD_2_Cl_2_, 21 °C): *δ*=7.77 (m, 2H, *ortho*-H), 7.64 (m, 1H, *para*-H), 7.54 (m, 2H, *meta*-H) ppm.

^13^C NMR (101 MHz, *o*-DFB, CD_2_Cl_2_, 21 °C): *δ*=135.4 (*ortho-C*), 131.6 (*para*-C), 131.0 (*ipso*-C), 128.4 (*meta*-C) ppm.

^19^F NMR (376 MHz, *o*-DFB, CD_2_Cl_2_, 20 °C): *δ*=–39.8 (**A**B_4_X, 1F, ^1^*J*(^19^F,^125^Te)=3476 Hz, ^2^*J*(^19^F,^19^F)=189 Hz), –41.3 (A**B**_4_X, 4F, ^1^*J*(^19^F,^125^Te)=3605 Hz, ^2^*J*(^19^F,^19^F)=189 Hz) ppm.

^19^F NMR (376 MHz, CH_2_Cl_2_, external lock acetone-d6, 18 °C): *δ*=–39.3 (**A**B_4_X, 1F, ^1^*J*(^19^F,^125^Te)=3476 Hz, ^2^*J*(^19^F,^19^F)=189 Hz), –41.0 (A**B**_4_X, 4F, ^1^*J*(^19^F,^125^Te)=3612 Hz, ^2^*J*(^19^F,^19^F)=189 Hz) ppm.

^29^Si DEPT NMR (80 MHz, *o*-DFB, CD_2_Cl_2_, 21 °C): *δ*=–1.1 ppm.

^1^Δ^19^F(^130^Te,^128^Te)=–0.004 ppm

IR (ATR, 25 °C): $\tilde{\nu}$=3073(w), 1589 (w), 1507 (w), 1486 (m, C–H), 1429 (w), 1269 (w), 1191 (w), 1120 (m, Si–C), 997 (w), 904 (m, Si–O), 744 (m), 715 (s), 695 (s, Te–F), 679 (w, Te–F), 544 (m), 504 (s), 439 (m).

FT-Raman (25 °C): $\tilde{\nu}$=3058 (m, C–H), 1592 (m, C–C), 1570 (w), 1269 (w), 1191 (w), 1162 (w), 1107 (w), 1027 (m), 1000 (s, C–H), 906 (w, Si–O), 763 (m), 699 (m, Te–F), 643 (w), 620 (w), 548 (w), 299 (w), 239 (w), 202 (w), 172 (w), 74 (s).

## Formation of Me_2_Si(OTeF_5_)_2_ (2a)

A solution of Me_2_SiCl_2_ (65 mg, 0.50 mmol) in 0.5 mL of CH_2_Cl_2_ was added at r.t. to a suspension of AgOTeF_5_ (348 mg, 1.00 mmol) in 1.5 mL of CH_2_Cl_2_. The mixture was stirred for 24 hours at r.t. A white solid precipitated. The supernatant was isolated as colourless liquid with a PFA transfer canula and characterized as Me_2_Si(OTeF_5_)_2_ (92% NMR yield).

^1^H NMR (401 MHz, CH_2_Cl_2_, external lock acetone-d6, 18 °C): *δ*=0.64 (s, 6H, Si-CH_3_, ^1^*J*(^13^C,^1^H)=18 Hz,) ppm.

^13^C NMR (101 MHz, CH_2_Cl_2_, external lock acetone-d6, 18 °C): *δ*=–0.2 (Si-CH_3_) ppm.

^19^F NMR (377 MHz, CH_2_Cl_2_, external lock acetone-d6, 18 °C): *δ*=–41.9 (**A**B_4_X, 1F, ^1^*J*(^19^F,^125^Te)=3544 Hz, ^2^*J*(^19^F,^19^F)=188 Hz), –42.4 (A**B**_4_X, 4F, ^1^*J*(^19^F,^125^Te)=3586 Hz, ^2^*J*(^19^F,^19^F)=188 Hz) ppm.

^29^Si DEPT NMR (80 MHz, CH_2_Cl_2_, external lock acetone-d6, 18 °C): *δ*=11.2 ppm.

## Formation of Et_2_Si(OTeF_5_)_2_ (2b)

A solution of Et_2_SiCl_2_ (70 mg, 0.44 mmol) in 0.3 mL of CH_2_Cl_2_ was added at r.t. to a suspension of AgOTeF_5_ (307 mg, 0.89 mmol) in 0.7 mL of CH_2_Cl_2_. The mixture was stirred for 14 hours at r.t. A white solid precipitated. The supernatant was isolated as bronze liquid with a PFA transfer canula and characterized as Et_2_Si(OTeF_5_)_2_ (99% NMR yield).

^1^H NMR (401 MHz, CH_2_Cl_2_, external lock acetone-d6, 18 °C): *δ*=1.00-1.12 (A_2_B_3_, m, 10H, CH_2_CH_3_) ppm.

^13^C NMR (101 MHz, CH_2_Cl_2_, external lock acetone-d6, 19 °C): *δ*=6.6 (Si–CH_2_, ^1^*J*(^29^Si,^13^C)=75 Hz, ^1^*J*(^125^Te,^13^C)=14 Hz), 4.6 (CH_3_) ppm.

^19^F NMR (377 MHz, CH_2_Cl_2_, external lock acetone-d6, 18 °C): *δ*=–42.2 (**A**B_4_X, 1F, ^1^*J*(^19^F,^125^Te)=3538 Hz, ^2^*J*(^19^F,^19^F)=188 Hz), –43.0 (A**B**_4_X, 4F, ^1^*J*(^19^F,^125^Te)=3592 Hz, ^2^*J*(^19^F,^19^F)=188 Hz) ppm.

^29^Si DEPT NMR (80 MHz, CH_2_Cl_2_, external lock acetone-d6, 17 °C): *δ*=8.9 ppm.

^125^Te NMR (126 MHz, CH_2_Cl_2_, external lock acetone-d6, 20 °C): *δ*=546 (dquin, ^1^*J*(^125^Te,^19^F_ax_)=3538 Hz, ^1^*J*(^125^Te,^19^F_eq_)=3592 Hz) ppm.

## Formation of *^i^*Pr_2_Si(OTeF_5_)_2_ (2c)

A solution of *^i^*Pr_2_SiCl_2_ (71 mg, 0.39 mmol) in 0.3 mL of CH_2_Cl_2_ was added at r.t. to a suspension of AgOTeF_5_ (267 mg, 0.77 mmol) in 0.7 mL of CH_2_Cl_2_. The mixture was stirred for 14 hours at r.t. A white solid precipitated. The supernatant was isolated as colourless liquid with a PFA transfer canula and characterized as *^i^*PrSi(OTeF_5_)_3_ (99.7% NMR yield).

^1^H NMR (401 MHz, CH_2_Cl_2_, external lock acetone-d6, 18 °C): *δ*=1.38 (sept, 1H, CH), 1.18 (br. d, 6H, CH_3_) ppm.

^13^C NMR (101 MHz, CH_2_Cl_2_, external lock acetone-d6, 16 °C): *δ*=15.2 (CH(**CH_3_**)_2_), 14.3 (Si–CH, ^1^*J*(^29^Si,^13^C)=75 Hz, ^2^*J*(^125^Te,^13^C)=11 Hz) ppm.

^19^F NMR (377 MHz, CH_2_Cl_2_, external lock acetone-d6, 16 °C): *δ*=–42.3 (**A**B_4_X, 1F, ^1^*J*(^19^F,^125^Te)=3533 Hz, ^2^*J*(^19^F,^19^F)=188 Hz), –43.4 (A**B**_4_X, 4F, ^1^*J*(^19^F,^125^Te)=3580 Hz, ^2^*J*(^19^F,^19^F)=189 Hz) ppm.

^29^Si DEPT NMR (80 MHz, CH_2_Cl_2_, external lock acetone-d6, 17 °C): *δ*=3.6 (s, ^2^*J*(^125^Te,^29^Si)=166 Hz) ppm.

^125^Te NMR (126 MHz, CH_2_Cl_2_, external lock acetone-d6, 22 °C): *δ*=546 (dquin, ^1^*J*(^125^Te,^19^F_ax_)=3533 Hz, ^1^*J*(^125^Te,^19^F_eq_)=3580 Hz) ppm.

## Synthesis of Ph_2_Si(OTeF_5_)_2_ (2d)

With a syringe Ph_2_SiCl_2_ (341 mg, 1.35 mmol) was added to a suspension of AgOTeF_5_ (887 mg, 2.56 mmol) in 1 mL of *o*-DFB. The mixture was stirred for 17 hours at r.t. A white solid precipitated. The supernatant was transferred and the solvent was removed under reduced pressure. The residue was recrystallized in *o*-DFB and stirred for 1.5 hours at 40 °C and characterized as Ph_2_Si(OTeF_5_)_3_ (60 mg, 0.09 mmol, 38% yield). Crystals suitable for sc-XRD were grown within 12 days by cooling the reaction solution slowly to –40 °C.

^1^H NMR (399 MHz, CH_2_Cl_2_, external lock acetone-d6, 18 °C): *δ*=7.74 (m, 2 H, *ortho-H*), 7.67 (m, 1 H, *para*-H), 7.54 (m, 2 H, *meta*-H) ppm.

^13^C NMR (100 MHz, CH_2_Cl_2_, external lock acetone-d6, 20 °C): *δ*=134.8 (s, 2C, *ortho-C*), 133.0 (s, 2C, *para*-C), 128.5 (s, 2C, *meta*-C), 126.3 (s, 1C, *ipso*-C) ppm.

^19^F NMR (377 MHz, CH_2_Cl_2_, external lock acetone-d6, 17 °C): *δ*=–42.0 (**A**_4_BX, 4F, ^1^*J*(^19^F,^125^Te)=3611 Hz, ^2^*J*(^19^F,^19^F)=188 Hz), –43.0 (A_4_**B**X, 1F, ^1^*J*(^19^F,^125^Te)=3560 Hz, ^2^*J*(^19^F,^19^F)=188 Hz) ppm.

^29^Si DEPT NMR (79 MHz, CH_2_Cl_2_, external lock acetone-d6, 20 °C): *δ*=–26.5 ppm.

^125^Te NMR (126 MHz, CH_2_Cl_2_, external lock acetone-d6, *18* °C): *δ*=546 (dquin, ^1^*J*(^125^Te,^19^F_ax_)=3560 Hz, ^1^*J*(^125^Te,^19^F_eq_)=3611 Hz) ppm.

IR (ATR, 25 °C): $\tilde{\nu}$=3077 (w), 1593 (w), 1489 (w), 1432 (m, C–H), 1130 (m), 1118 (m, Si–C), 999 (w), 945 (m, Si–O), 929 (m), 913 (m, Si–O), 853 (w), 745 (w), 713 (s), 691 (s, Te–F), 620 (w, Te–F), 580 (m), 536 (m), 507 (m), 466 (w), 447 (w), 421 (w).

FT-Raman (25 °C): $\tilde{\nu}$=3050 (s), 2965 (w), 1592 (m), 1569 (w), 1190 (w), 1159 (w), 1121 (w, Si–C), 1031 (w), 1000 (s), 619 (w, Te–F), 561 (w), 236 (w), 202 (w), 84 (s), 71 (s).

## Formation of MeSi(OTeF_5_)_3_ (3a)

A solution of MeSiCl_3_ (32 mg, 0.21 mmol) in 0.3 mL of CH_2_Cl_2_ was added at r.t. to a suspension of AgOTeF_5_ (257 mg, 0.74 mmol) in 0.7 mL of CH_2_Cl_2_. The mixture was stirred for 19 hours at r.t. A white solid precipitated. The supernatant was isolated as colourless liquid with a PFA transfer canula and characterized as MeSi(OTeF_5_)_3_ (60% NMR yield).

^1^H NMR (401 MHz, CH_2_Cl_2_, external lock acetone-d6, 18 °C): *δ*=0.83 (s, 3H, CH_3_) ppm.

^13^C NMR (101 MHz, CH_2_Cl_2_, external lock acetone-d6, 19 °C): *δ*=54.4 (CH_3_) ppm.

^19^F NMR (377 MHz, CH_2_Cl_2_, external lock acetone-d6, 19 °C): *δ*=–41.7 (**A**_4_BX, 4F, ^1^*J*(^19^F,^125^Te)=3610 Hz, ^2^*J*(^19^F,^19^F)=187 Hz), –44.4 (A_4_**B**X, 1F, ^1^*J*(^19^F,^125^Te)=3643 Hz, ^2^*J*(^19^F,^19^F)=187 Hz) ppm.

^29^Si DEPT NMR (80 MHz, CH_2_Cl_2_, external lock acetone-d6, 20 °C): *δ*=–52.6 (s, ^2^*J*(^125^Te,^29^Si)=71 Hz) ppm.

^125^Te NMR (126 MHz, CH_2_Cl_2_, external lock acetone-d6, 19 °C): *δ*=538 (dquin, ^1^*J*(^125^Te,^19^F_eq_)=3610 Hz, ^1^*J*(^125^Te,^19^F_ax_)=3643 Hz) ppm.

## Formation of EtSi(OTeF_5_)_3_ (3b)

A solution of EtSiCl_3_ (41 mg, 0.25 mmol) in 0.4 mL of CH_2_Cl_2_ was added at r.t. to a suspension of AgOTeF_5_ (329 mg, 0.95 mmol) in 0.4 mL of CH_2_Cl_2_. The mixture was stirred for 22 hours at r.t. A white solid precipitated. The supernatant was isolated with a PFA transfer canula. The product was obtained as bronze liquid and characterized as EtSi(OTeF_5_)_3_ (67% NMR yield).

^1^H NMR (401 MHz, CH_2_Cl_2_, external lock acetone-d6, 18 °C): *δ*=1.20 (**A**_2_B_3_, m, 2H, ^3^*J*(^1^H, ^1^H)≈7 Hz, CH_2_), 1.10 (A_2_**B**_3_, m, ^3^*J*(^1^H,^1^H)=7 Hz, 3H, CH_3_) ppm.

^13^C NMR (100 MHz, CH_2_Cl_2_, external lock acetone-d6, 20 °C): *δ*=4.6 (s, 1C, CH_2_), 4.4 (s, 1C, CH_3_) ppm.

^19^F NMR (377 MHz, CH_2_Cl_2_, external lock acetone-d6, 19 °C): *δ*=–42.0 (**A**_4_BX, 4F, ^1^*J*(^19^F,^125^Te)=3610 Hz, ^2^*J*(^19^F,^19^F)=187 Hz), –44.5 (A_4_**B**X, 1F, ^1^*J*(^19^F,^125^Te)=3639 Hz, ^2^*J*(^19^F,^19^F)=187 Hz) ppm.

^29^Si DEPT NMR (80 MHz, CH_2_Cl_2_, external lock acetone-d6, 17 °C): *δ*=–55.3 ppm.

^125^Te NMR (126 MHz, CH_2_Cl_2_, external lock acetone-d6, 20 °C): *δ*=540 (dquin, ^1^*J*(^19^F,^125^Te)=3639 Hz, ^1^*J*(^19^F,^125^Te)=3610 Hz) ppm.

## Synthesis of *^t^*BuSi(OTeF_5_)_3_ (3c)

AgOTeF_5_ (354 mg, 1.02 mmol) was dissolved in 0.9 mL of CH_2_Cl_2_ and *^t^*BuSiCl_3_ (48.9 mg, 0.26 mmol) was added. The mixture was stirred for 3 hours at 50 °C. A white solid precipitated. The supernatant was isolated with a PFA transfer canula and characterized as *^t^*BuSi(OTeF_5_)_3_ (94 mg, 0.18 mmol, 43% NMR yield). Crystals suitable for sc-XRD were grown within 10 days by cooling the reaction solution slowly to –40 °C.

^1^H NMR (401 MHz, CH_2_Cl_2_, external lock acetone-d6, 18 °C): *δ*=1.14 (s, 9H, CH_3_) ppm.

^13^C NMR (101 MHz, CH_2_Cl_2_, external lock acetone-d6, 19 °C): *δ*=24.5 (**C**(CH**_3_**)_2_), 22.3 (C(**CH_3_**)_2_) ppm.

^19^F NMR (377 MHz, CH_2_Cl_2_, external lock acetone-d6, 19 °C): *δ*=–42.3 (**A**_4_BX, 4F, ^1^*J*(^19^F,^125^Te)=3625 Hz, ^2^*J*(^19^F,^19^F)=187 Hz), –44.6 (A_4_**B**X, 1F, ^1^*J*(^19^F,^125^Te)=3634 Hz, ^2^*J*(^19^F,^19^F)=187 Hz) ppm.

^29^Si DEPT NMR (80 MHz, CH_2_Cl_2_, external lock acetone-d6, 17 °C): *δ*=–62.4 (s, ^2^*J*(^125^Te,^29^Si)=177 Hz) ppm.

^125^Te NMR (126 MHz, CH_2_Cl_2_, external lock acetone-d6, 17 °C): *δ*=541 (dquin, ^1^*J*(^19^F,^125^Te)=3634 Hz, ^1^*J*(^19^F,^125^Te)=3625 Hz) ppm.

IR (ATR, 25 °C): $\tilde{\nu}$=2972 (w), 2877 (w), 1477 (w, C–H), 1374 (w), 1015 (w, Si–O), 969 (s, Si–O), 942 (w), 827 (m, si–C), 726 (s, Te–F), 710 (s, Te–F), 651 (m), 590 (m), 439 (m).

FT-Raman (25 °C): $\tilde{\nu}$=2988 (s, C–H), 704 (s, Te–F), 288 (w), 70 (w), 62 (w).

## Formation of PhSi(OTeF_5_)_3_ (3d)

A solution of PhSiCl_3_ (63 mg, 0.30 mmol) in 0.4 mL of *o*-DFB was added at r.t. to a suspension of AgOTeF_5_ (407 mg, 1.18 mmol) in 0.4 mL of *o*-DFB. The mixture was stirred for 22 hours at r.t. A white solid precipitated. The supernatant was isolated as colourless liquid with a PFA transfer canula (78% NMR yield).

^1^H NMR (401 MHz, *o*-DFB, external lock acetone-d6, 22 °C): *δ*=7.57 (m, 2H, CH_arom._), 7.49 (m, 1H, CH_arom._), 7.22 (m, 2H, CH_arom._) ppm.

^19^F NMR (377 MHz, *o*-DFB, external lock acetone-d6, 22°C): *δ*=–42.3 (**A**_4_BX, 4F, ^1^*J*(^19^F,^125^Te)=3629 Hz, ^2^*J*(^19^F,^19^F)=187 Hz), –45.8 (A_4_**B**X, 1F, ^1^*J*(^19^F,^125^Te)=3641 Hz, ^2^*J*(^19^F,^19^F)=187 Hz) ppm.

^29^Si DEPT NMR (80 MHz, *o*-DFB, external lock acetone-d6, 17 °C): *δ*=–71.4 ppm.

# NMR Spectra

## NMR spectra of Me_3_SiOTeF_5_ with HOTeF_5_ (1a)


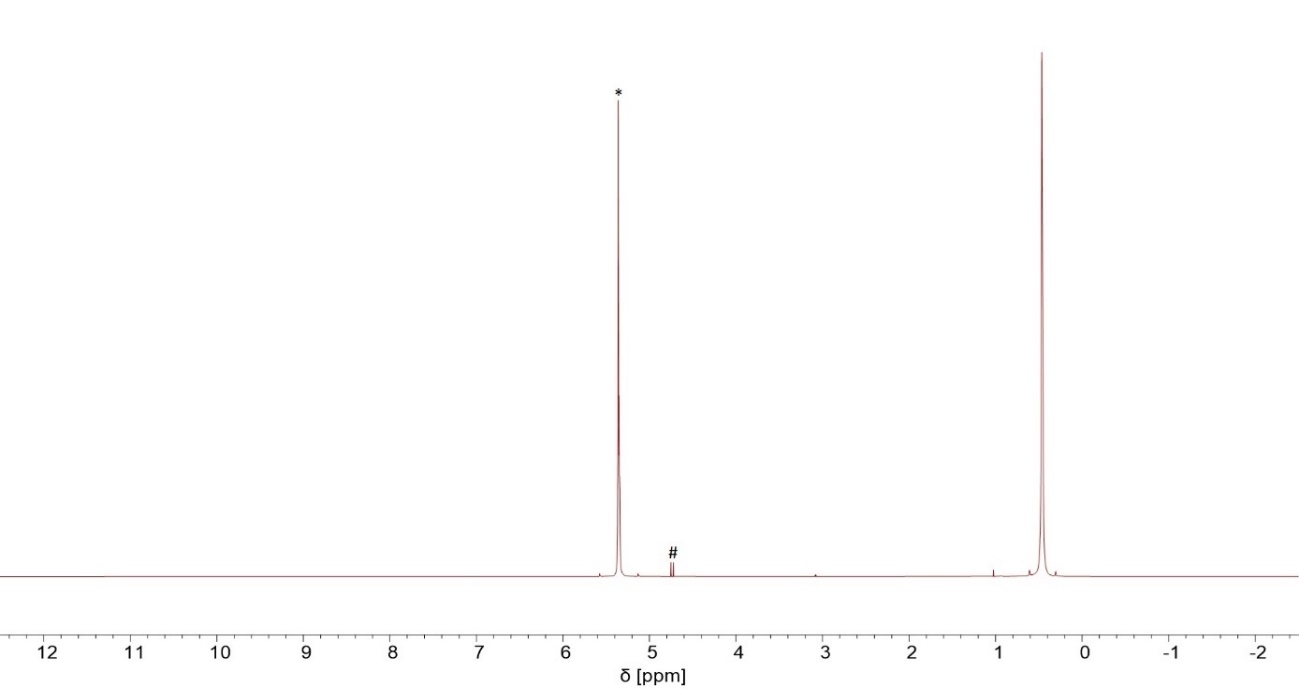


Figure S 1: ^1^H NMR spectrum (399 MHz, CH_2_Cl_2_, external lock acetone-d6, 20 °C) of Me_3_SiOTeF_5_ (*: CH_2_Cl_2_, #: ext. (CH_3_O)_3_PO).


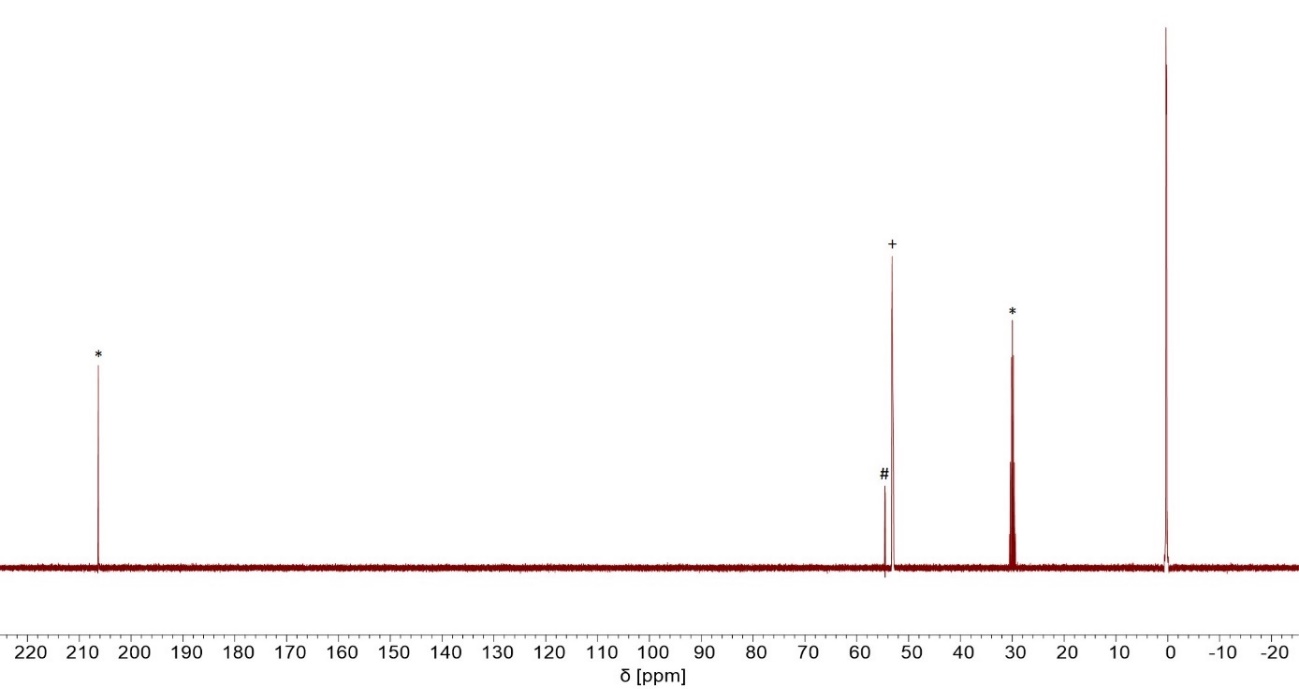


Figure S 2: ^13^C NMR spectrum (100 MHz, CH_2_Cl_2_, external lock acetone-d6, 20 °C) of Me_3_SiOTeF_5_ (*: ext. (CD_3_)_2_CO, #: ext. OP(OCH_3_)_3_, +: solvent).


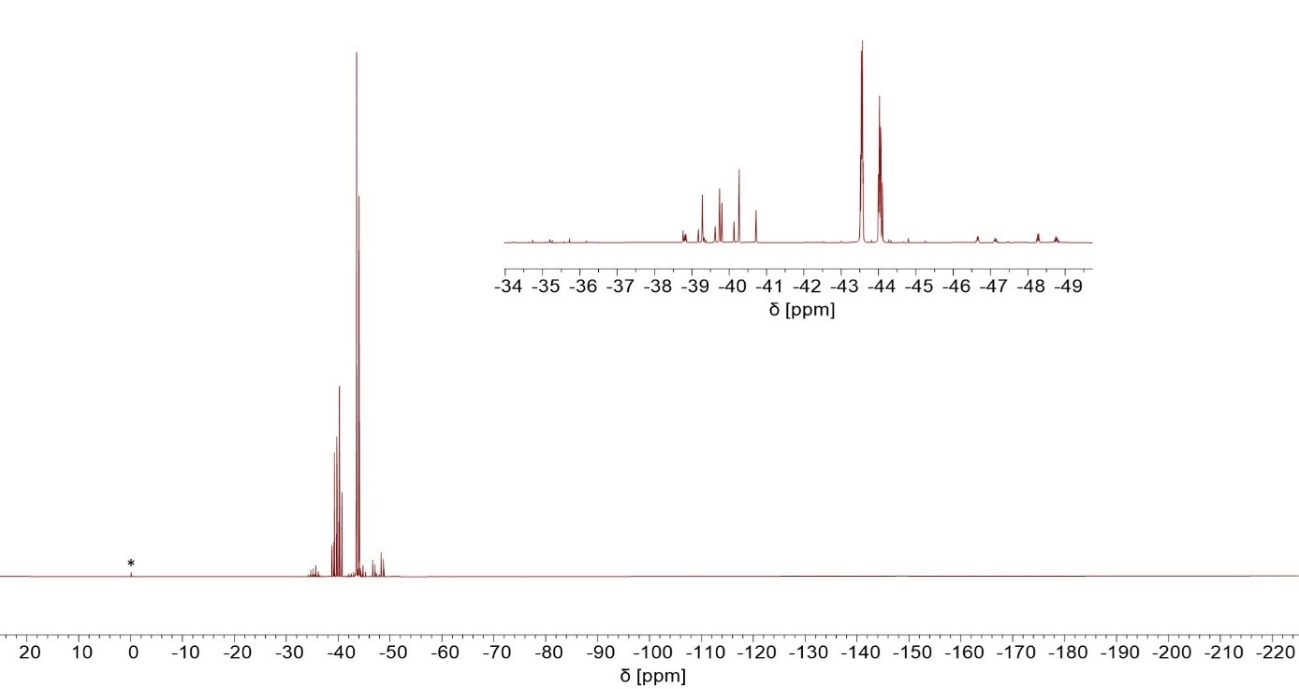


Figure S 3: ^19^F NMR spectrum (377 MHz, CH_2_Cl_2_, external lock acetone-d6, 20 °C) of Me_3_SiOTeF_5_ (*: ext. CFCl_3_).


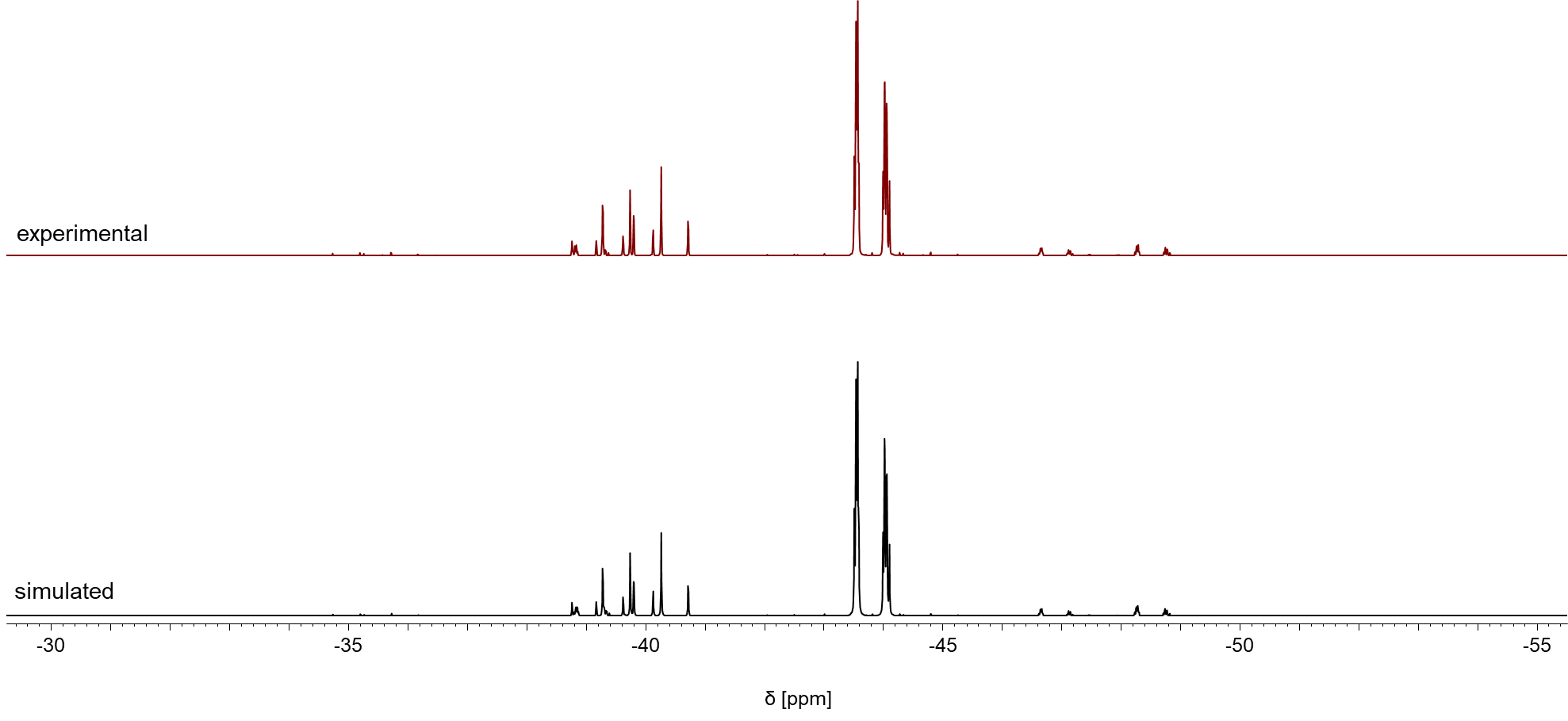


Figure S 4: Experimental (top) ^19^F NMR spectrum (377 MHz, CH_2_Cl_2_, external lock acetone-d6, 20 °C) and simulated (bottom) ^19^F NMR spectrum of Me_3_SiOTeF_5_.


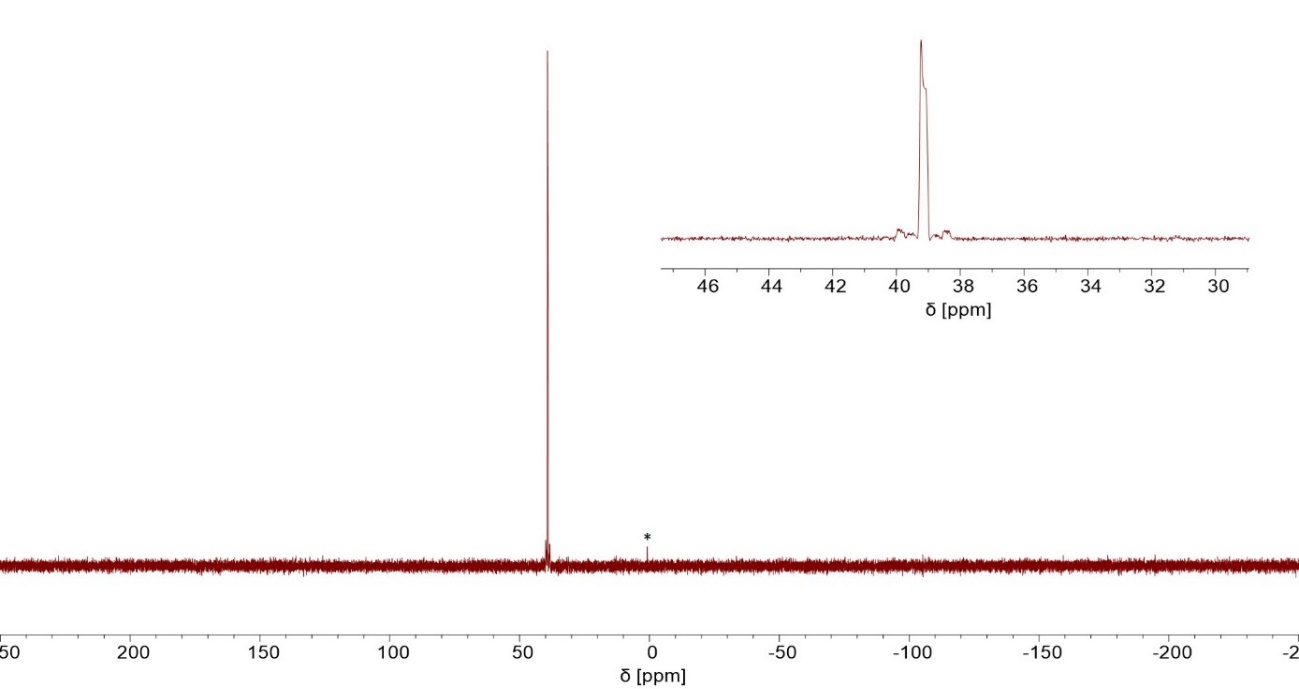


Figure S 5: ^29^Si DEPT NMR spectrum (79 MHz, CH_2_Cl_2_, external lock acetone-d6, 20 °C) of Me_3_SiOTeF_5_ (*: ext. Si(CH_3_)_4_).


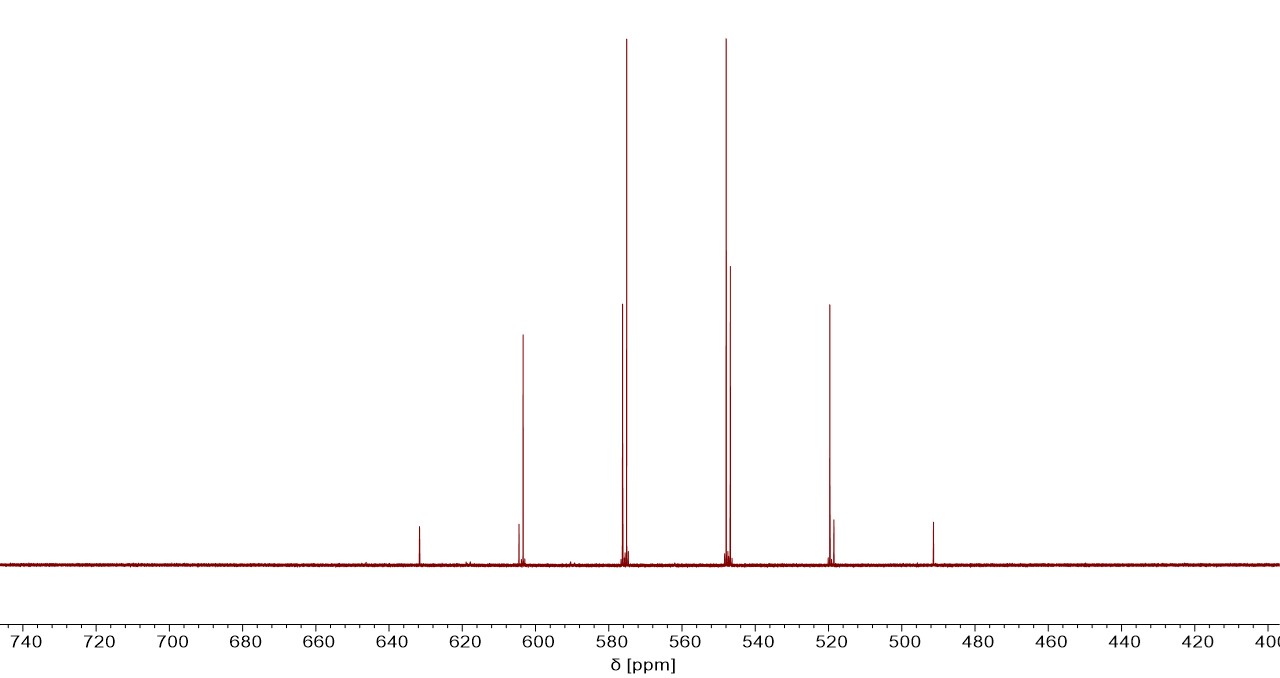


Figure S 6: ^125^Te NMR spectrum (126 MHz, CH_2_Cl_2_, external lock acetone-d6, 20 °C) of Me_3_SiOTeF_5_.

## NMR spectra of Et_3_SiOTeF_5_ with HOTeF_5_ (1b)


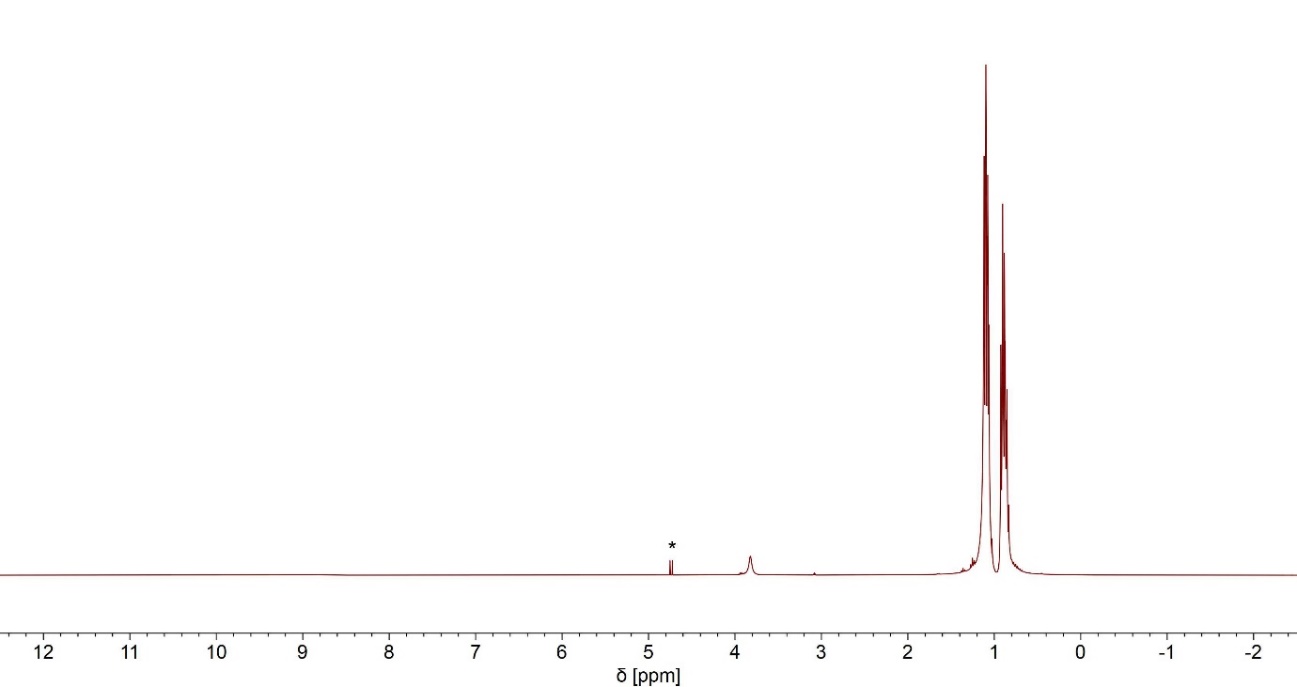


Figure S 7: ^1^H NMR spectrum (401 MHz, neat, external lock acetone-d6, 17 °C) of Et_3_SiOTeF_5_ (*: ext. (CH_3_O)_3_PO).


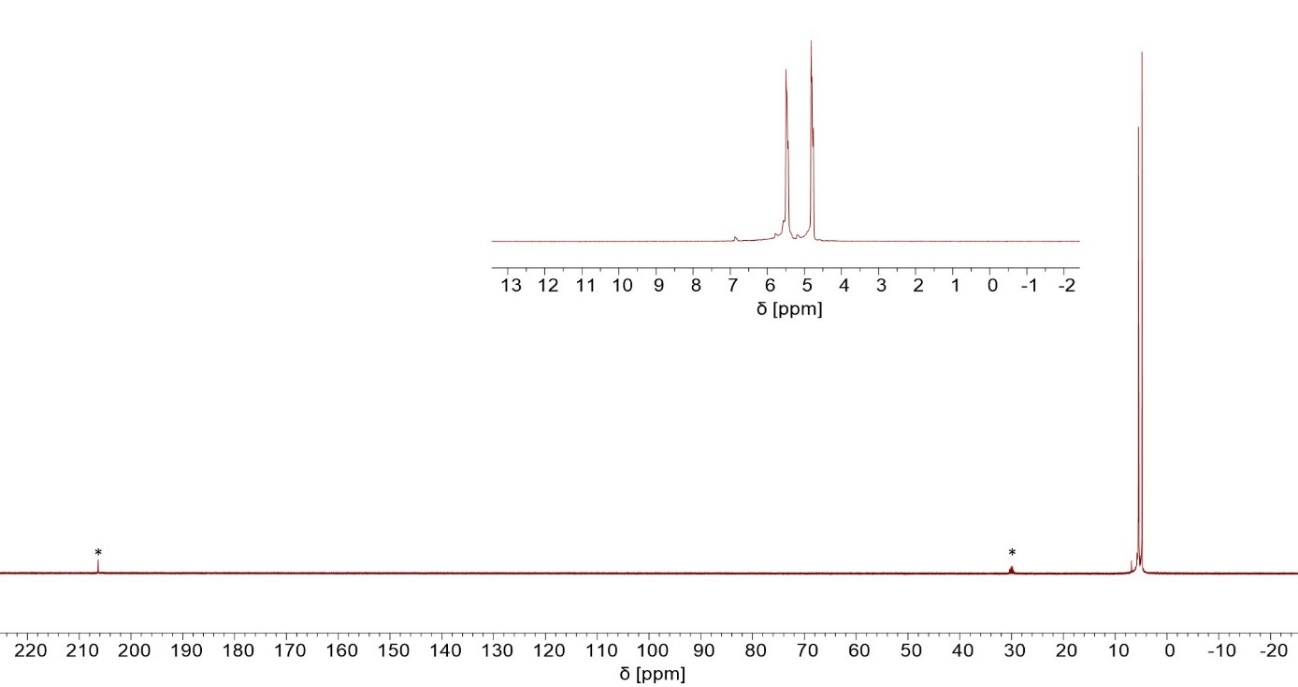


Figure S 8: ^13^C NMR spectrum (100 MHz, neat, external lock acetone-d6, 20 °C) of Et_3_SiOTeF_5_ (*: ext. (CD_3_)_2_CO).


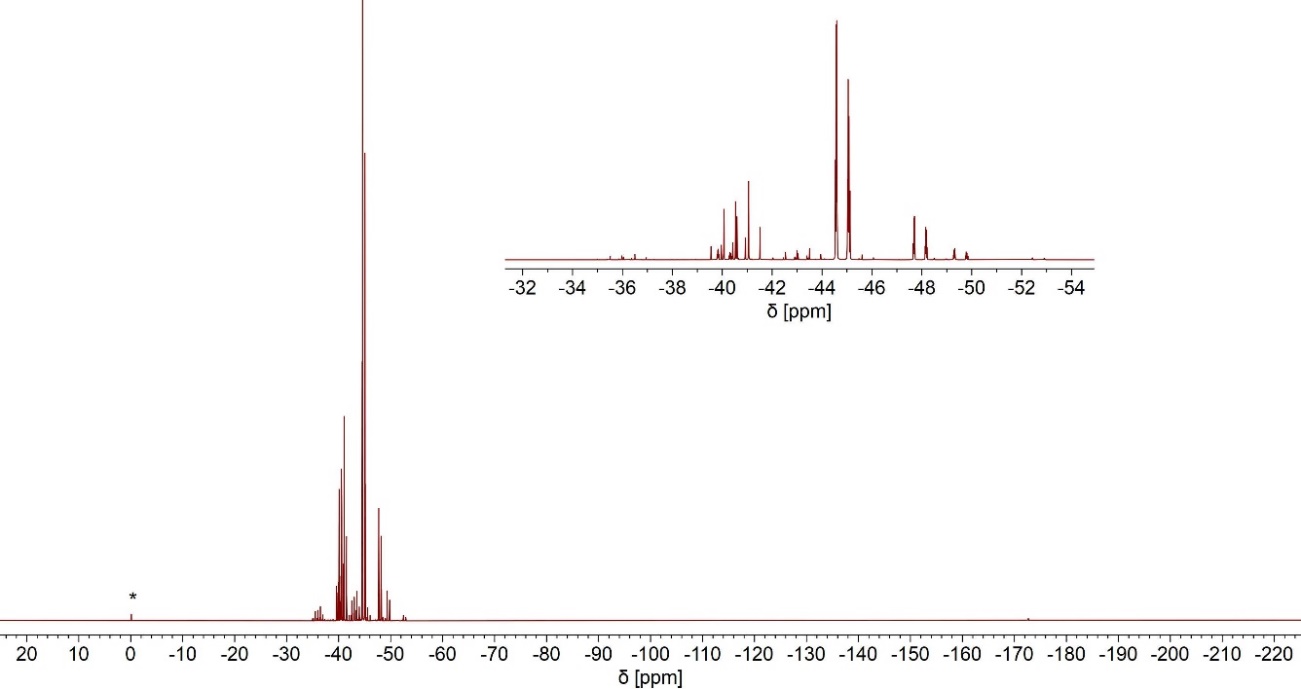


Figure S 9: ^19^F NMR spectrum (377 MHz, neat, external lock acetone-d6, 17 °C) of Et_3_SiOTeF_5_ (*: ext. CFCl_3_).


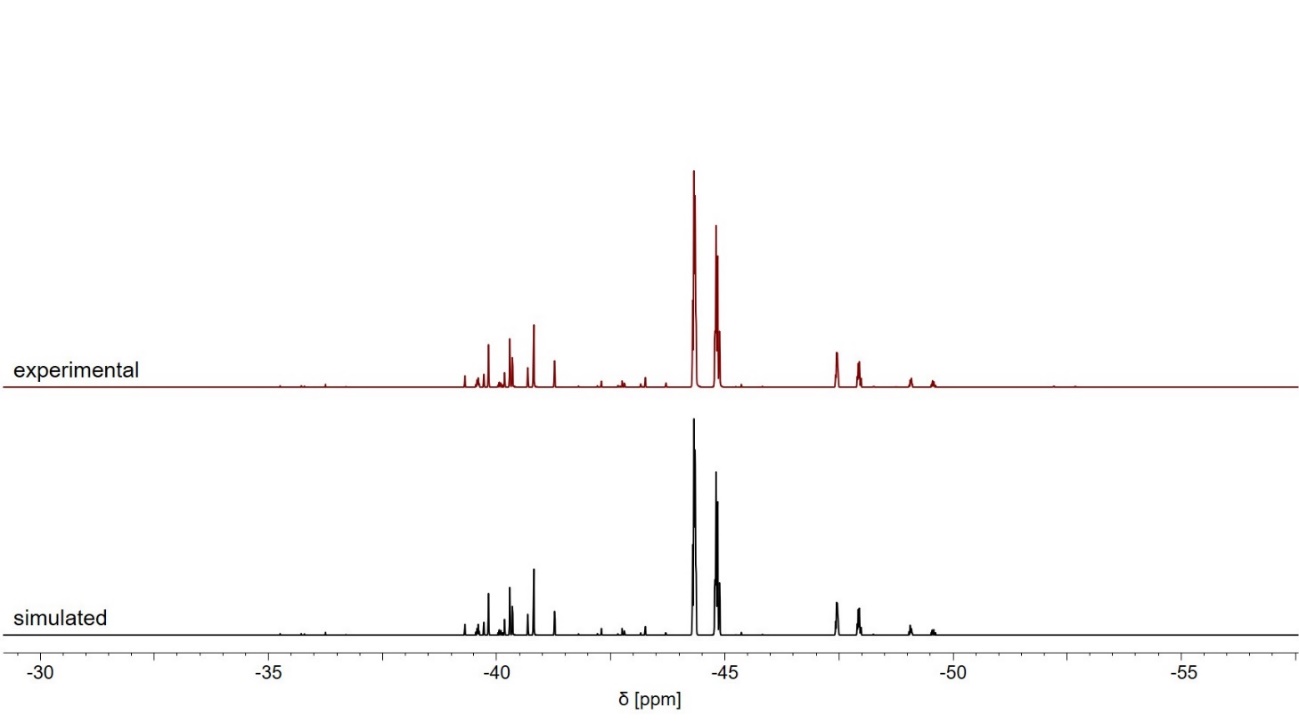


Figure S 10: Experimental (top) ^19^F NMR spectrum (377 MHz, neat, external lock acetone-d6, 17 °C) and simulated (bottom) ^19^F NMR spectrum of Et_3_SiOTeF_5_.


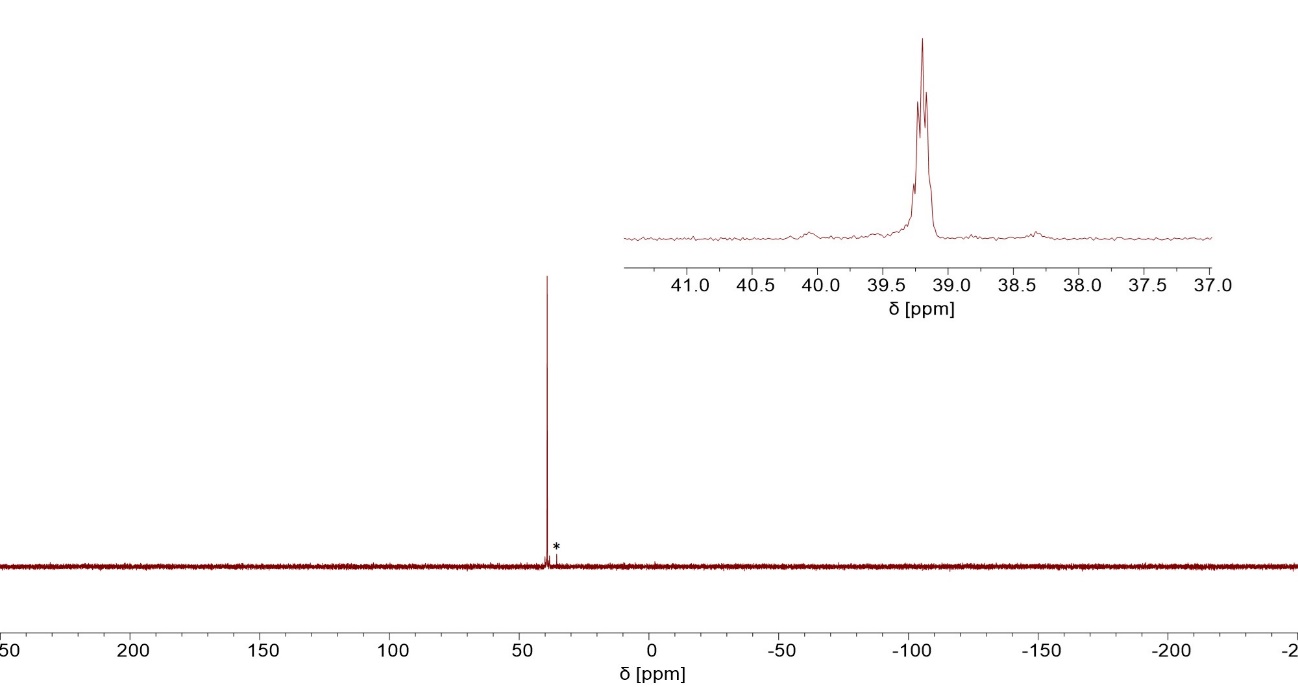


Figure S 11: ^29^Si DEPT NMR spectrum (79 MHz, neat, external lock acetone-d6, 20 °C) of Et_3_SiOTeF_5_ (*: Et_3_SiCl).

## NMR spectra of Et_3_SiOTeF_5_ with AgOTeF_5_ (1b)


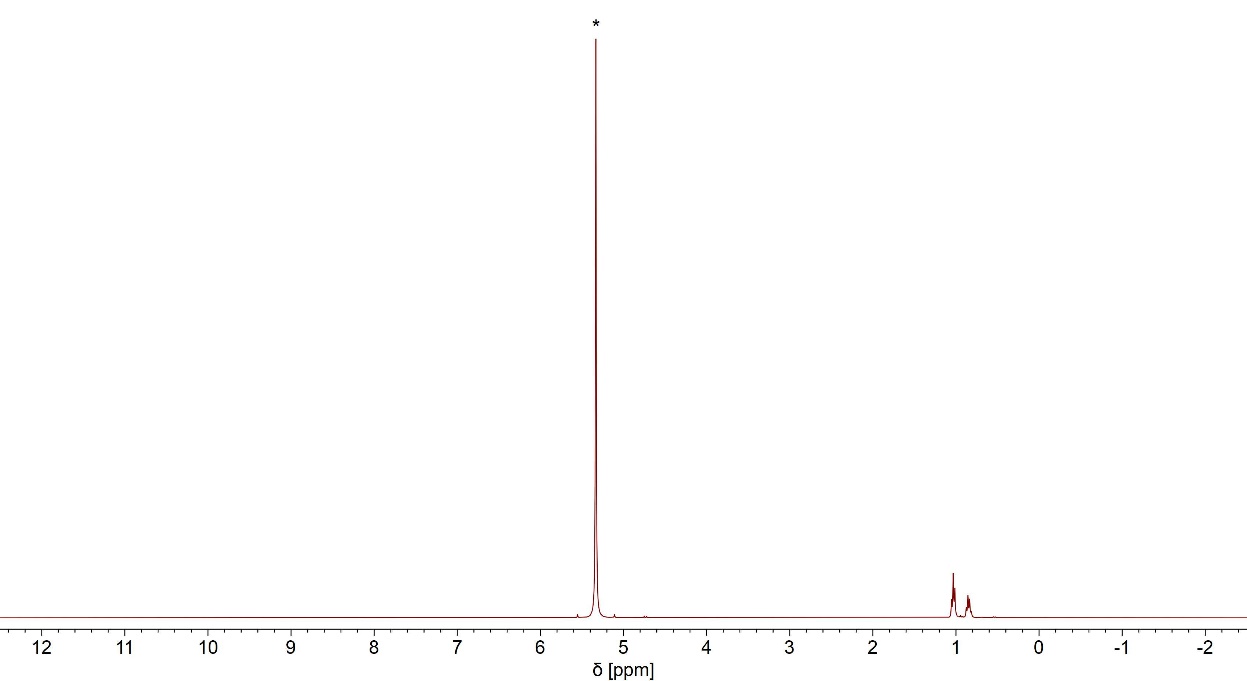


Figure S 12: ^1^H NMR spectrum (401 MHz, CH_2_Cl_2_, external lock acetone-d6, 18 °C) of Et_3_SiOTeF_5_ (*: solvent).


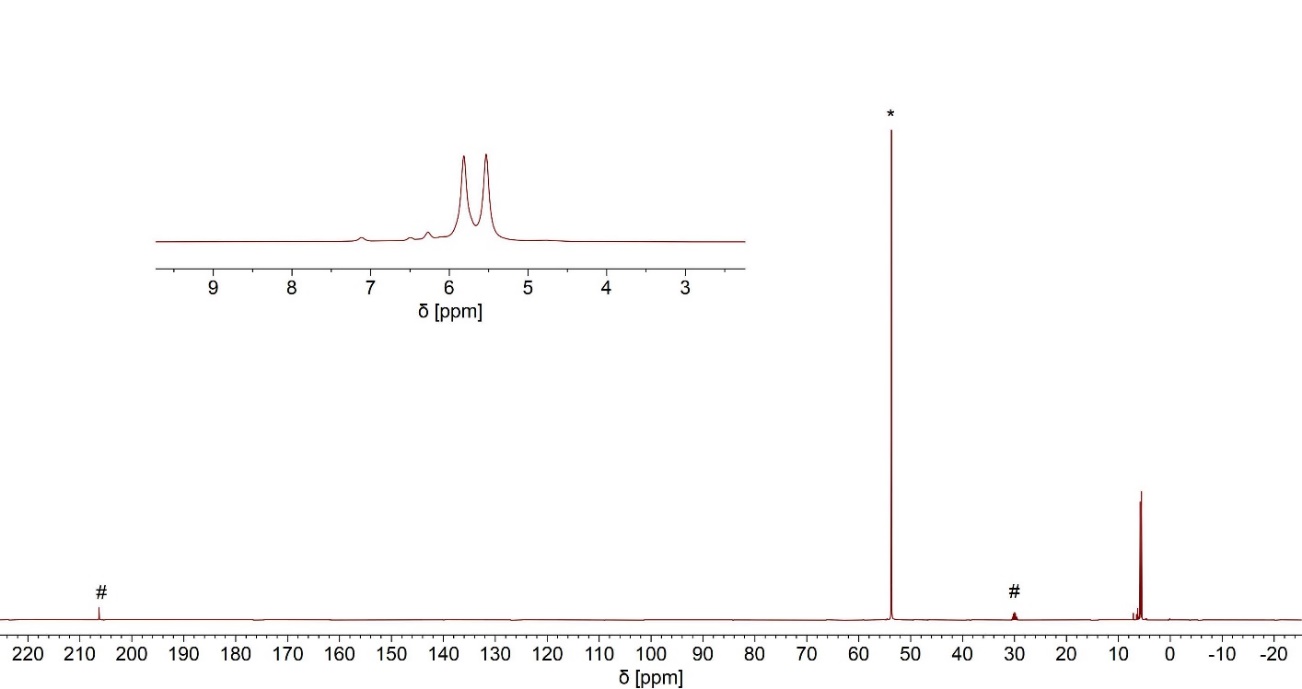


Figure S 13: ^13^C NMR spectrum (101 MHz, CH_2_Cl_2_, external lock acetone-d6, 18 °C) of Et_3_SiOTeF_5_ (*: solvent, #: ext. (CD_3_)_2_CO).


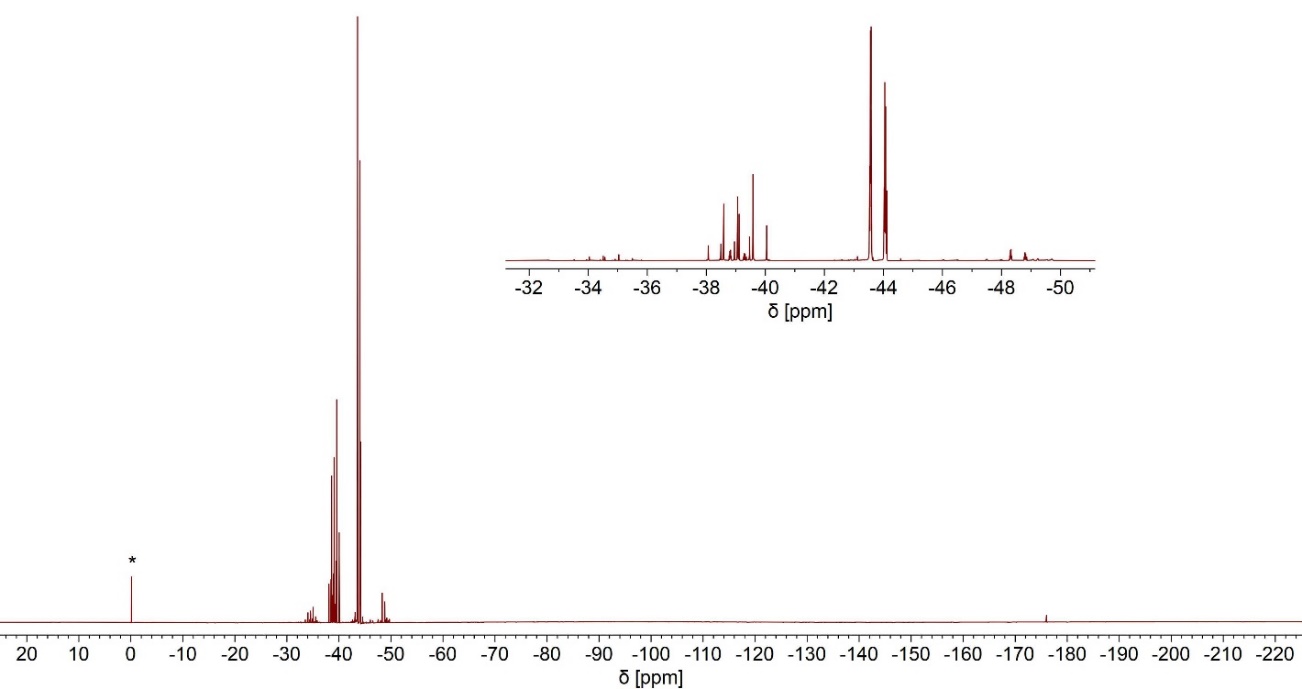


Figure S 14: ^19^F NMR spectrum (377 MHz, CH_2_Cl_2_, external lock acetone-d6, 18 °C) of Et_3_SiOTeF_5_ (*: ext. CFCl_3_).


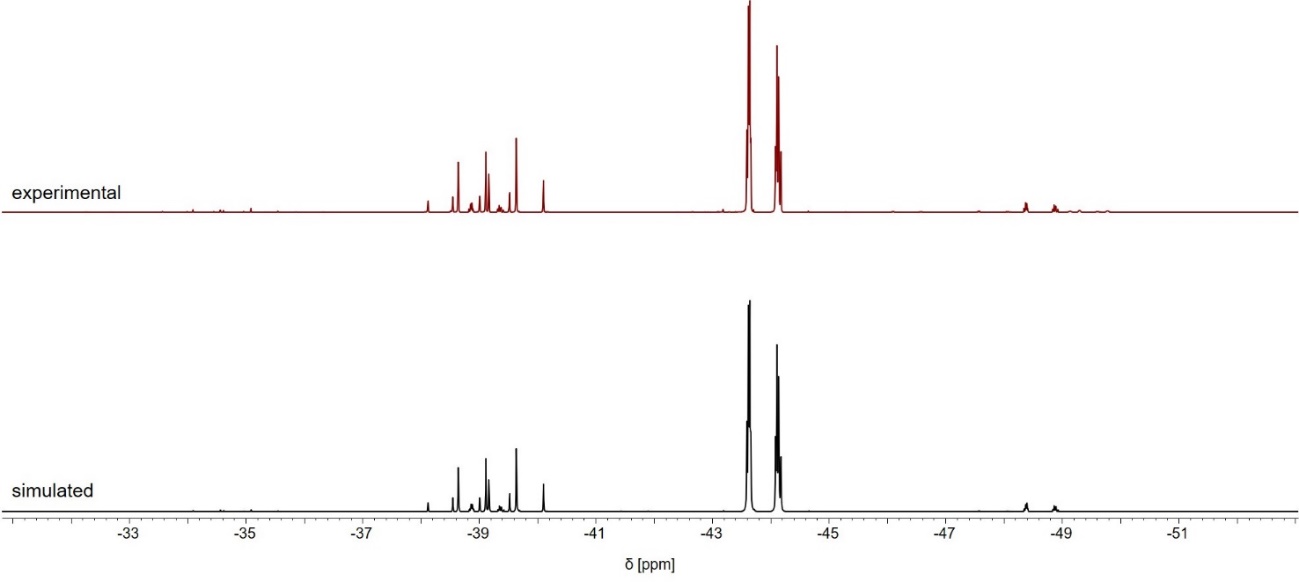


Figure S 15: Experimental (top) ^19^F NMR spectrum (377 MHz, CH_2_Cl_2_, external lock acetone-d6, 18 °C) and simulated (bottom) ^19^F NMR spectrum of Et_3_SiOTeF_5_.


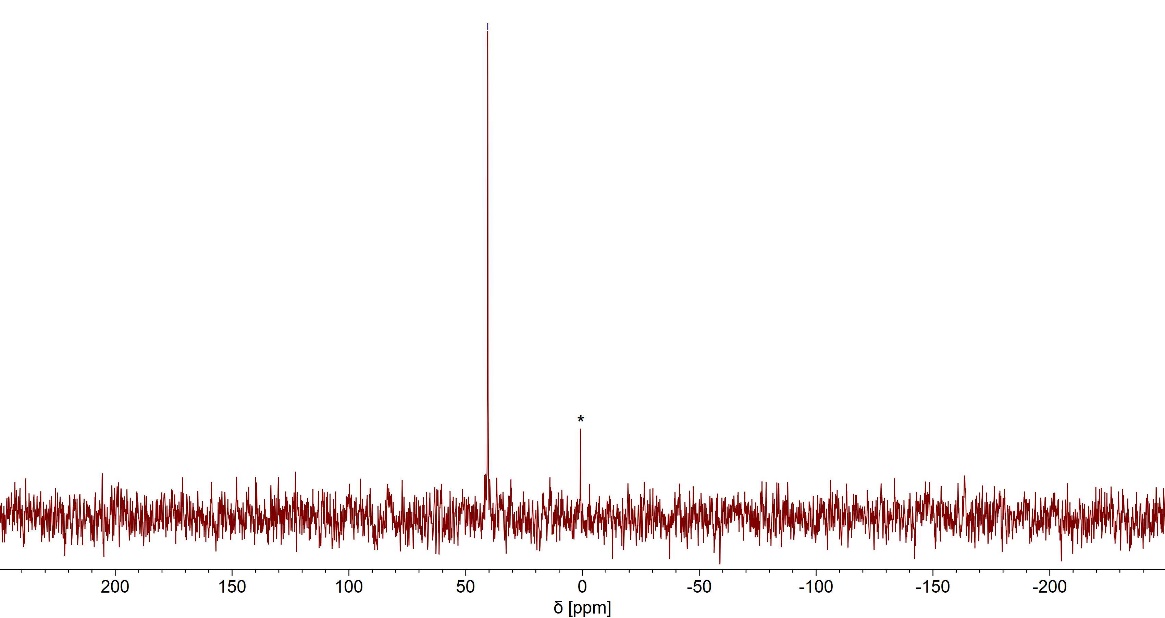


Figure S 16: ^29^Si DEPT NMR spectrum (80 MHz, CH_2_Cl_2_, external lock acetone-d6, 17 °C) of Et_3_SiOTeF_5_ (*: ext. Si(CH_3_)_4_).


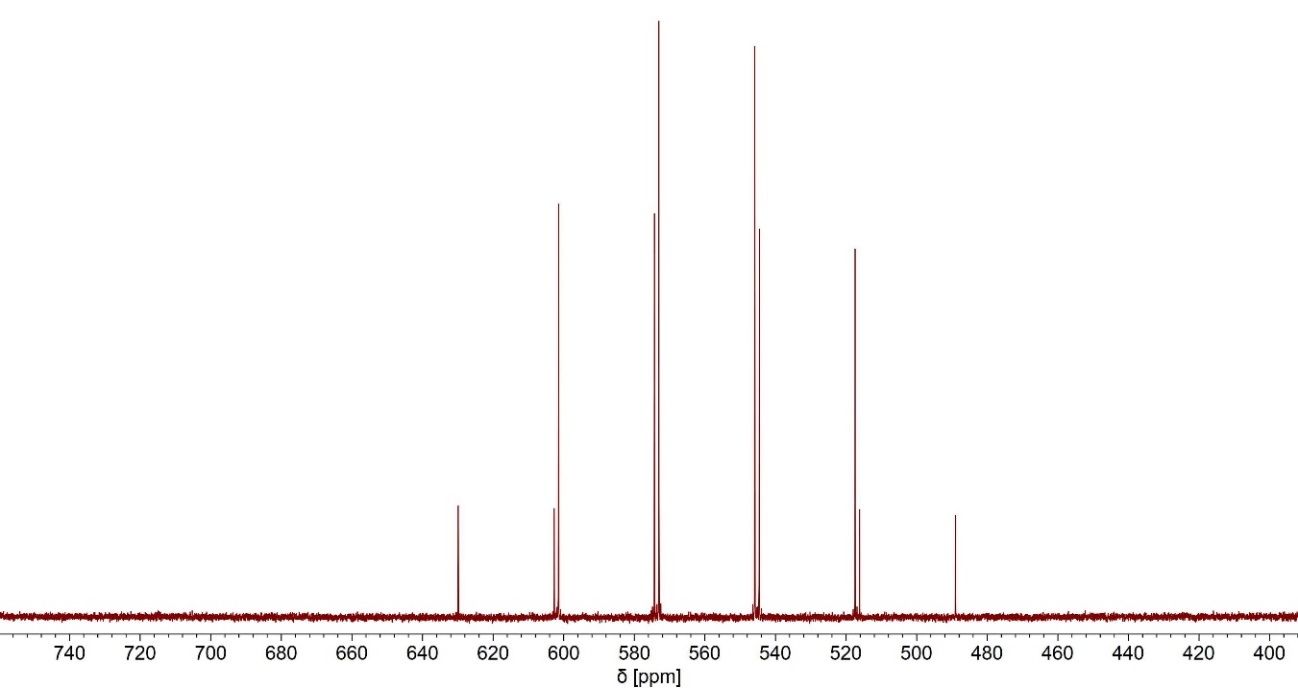


Figure S 17: ^125^Te NMR spectrum (126 MHz, neat, external lock acetone-d6, 19 °C) of Et_3_SiOTeF_5_.

## NMR spectra of Me_2_PrSiOTeF_5_ with HOTeF_5_ (1c)


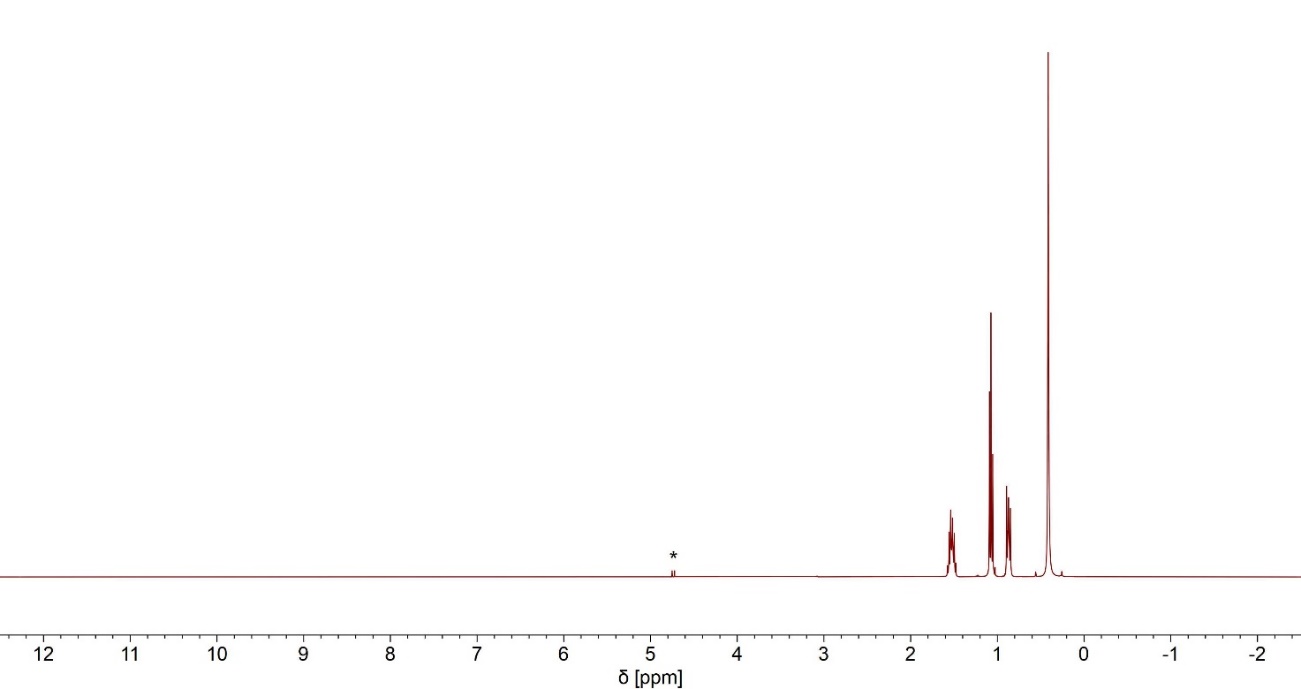


Figure S 18: ^1^H NMR spectrum (401 MHz, neat, external lock acetone-d6, 17 °C) of Me_2_PrSiOTeF_5_ (*: ext. (CH_3_O)_3_PO).


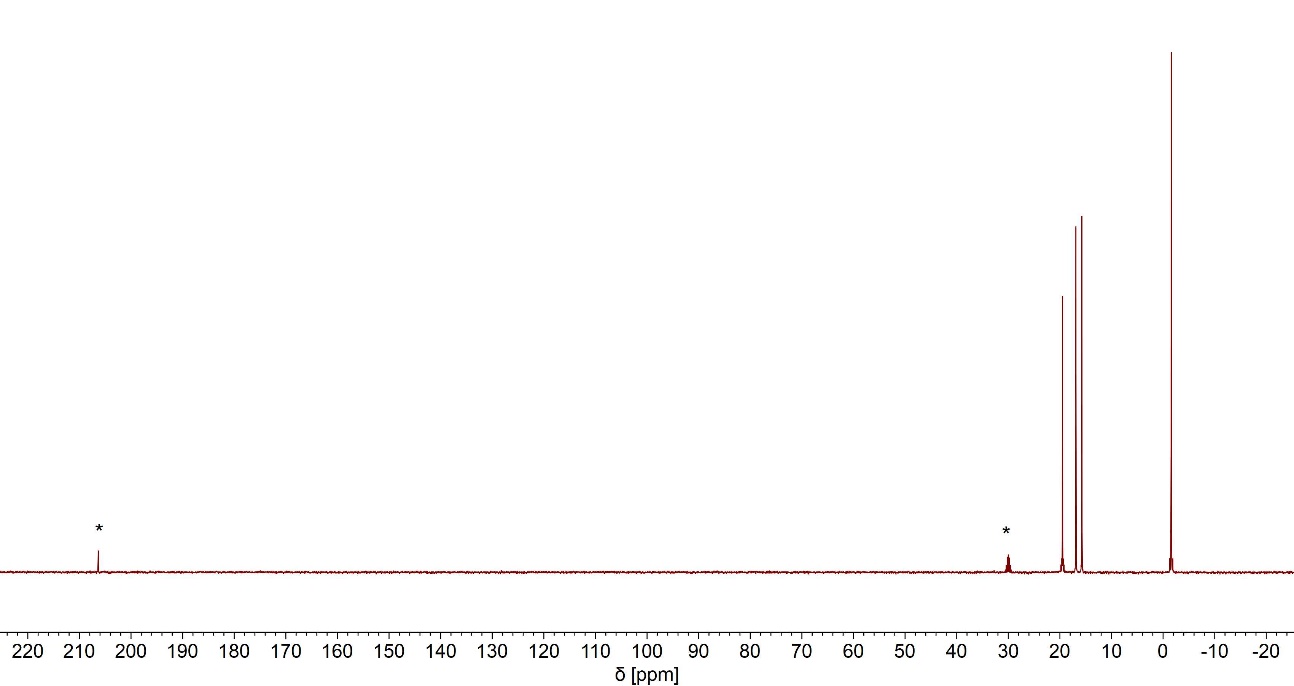


Figure S 19: ^13^C NMR spectrum (101 MHz, neat, external lock acetone-d6, 17 °C) of Me_2_PrSiOTeF_5_ (*: ext. (CD_3_)_2_CO).


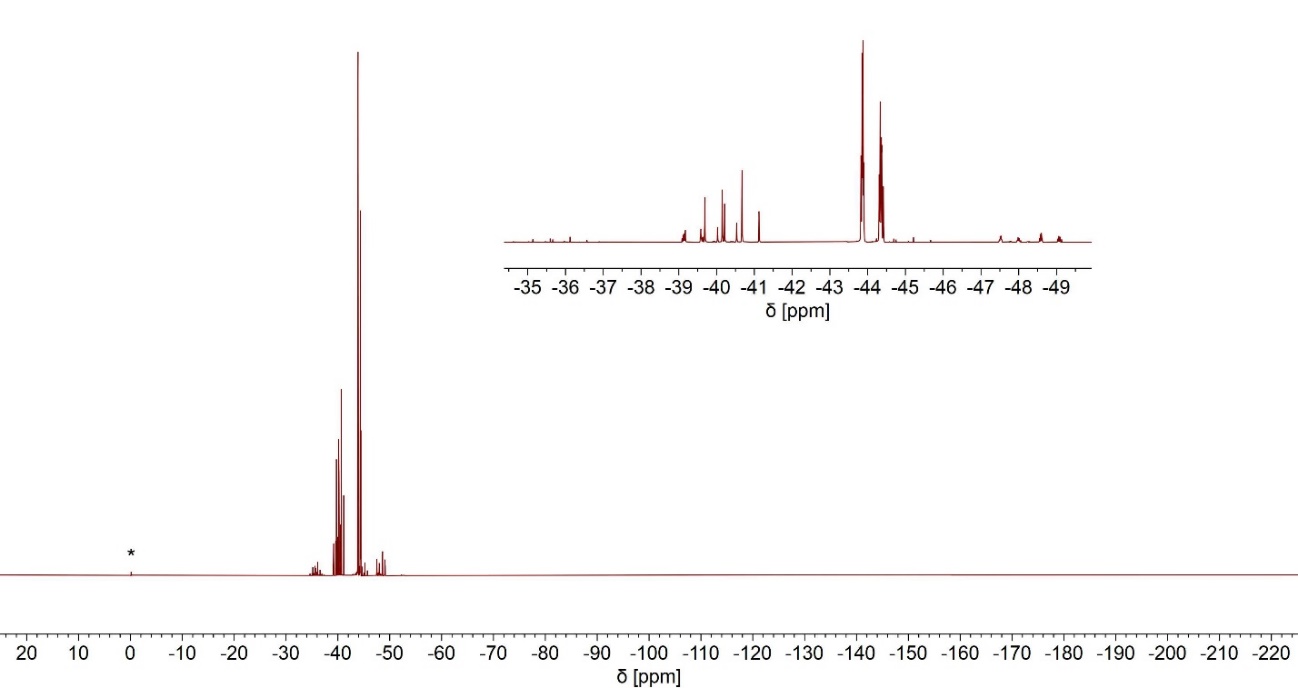


Figure S 20: ^19^F NMR spectrum (377 MHz, neat, external lock acetone-d6, 17 °C) of Me_2_PrSiOTeF_5_ (*: ext. CFCl_3_).


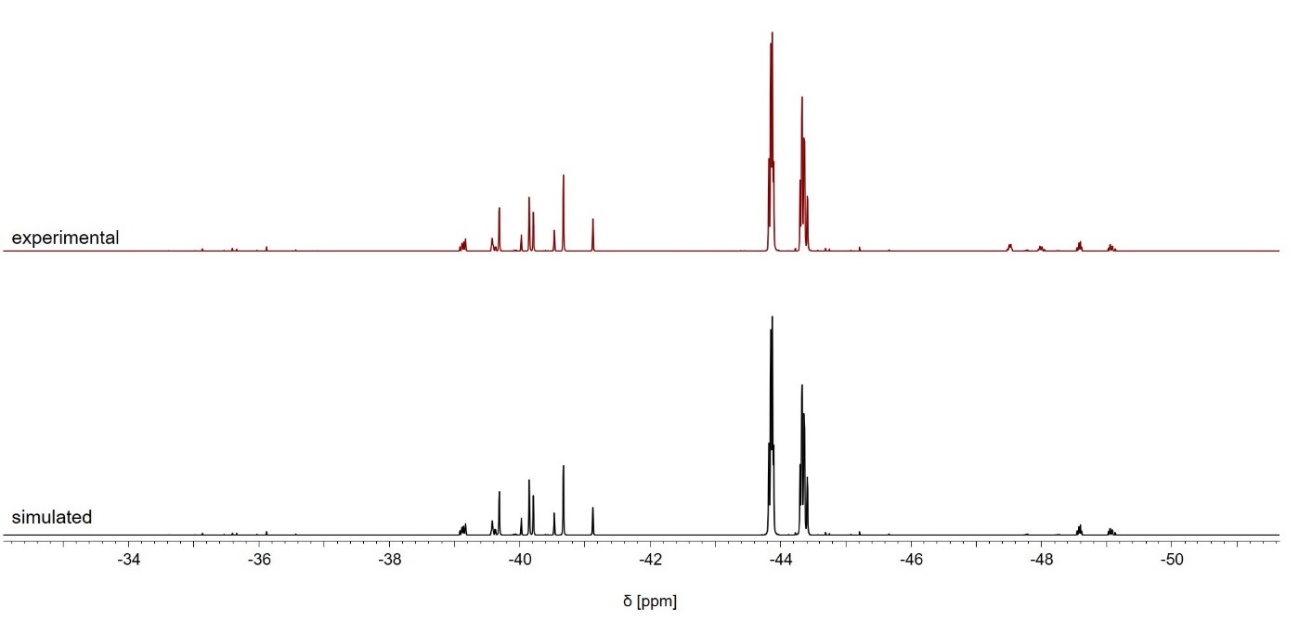


Figure S 21: Experimental (top) ^19^F NMR spectrum (377 MHz, neat, external lock acetone-d6, 17 °C) and simulated (bottom) ^19^F NMR spectrum of Me_2_PrSiOTeF_5_.


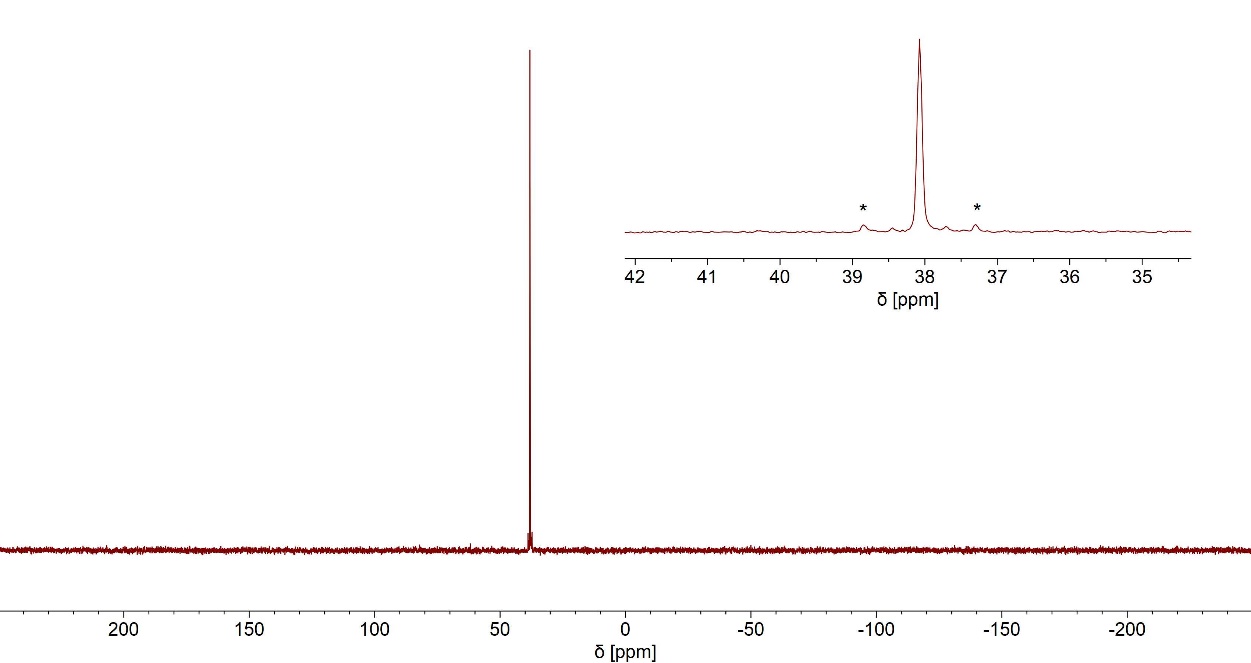


Figure S 22: ^29^Si DEPT NMR spectrum (80 MHz, neat, external lock acetone-d6, 17 °C) of Me_2_PrSiOTeF_5_. ^125^Te Satellites are marked with an asterix (*).


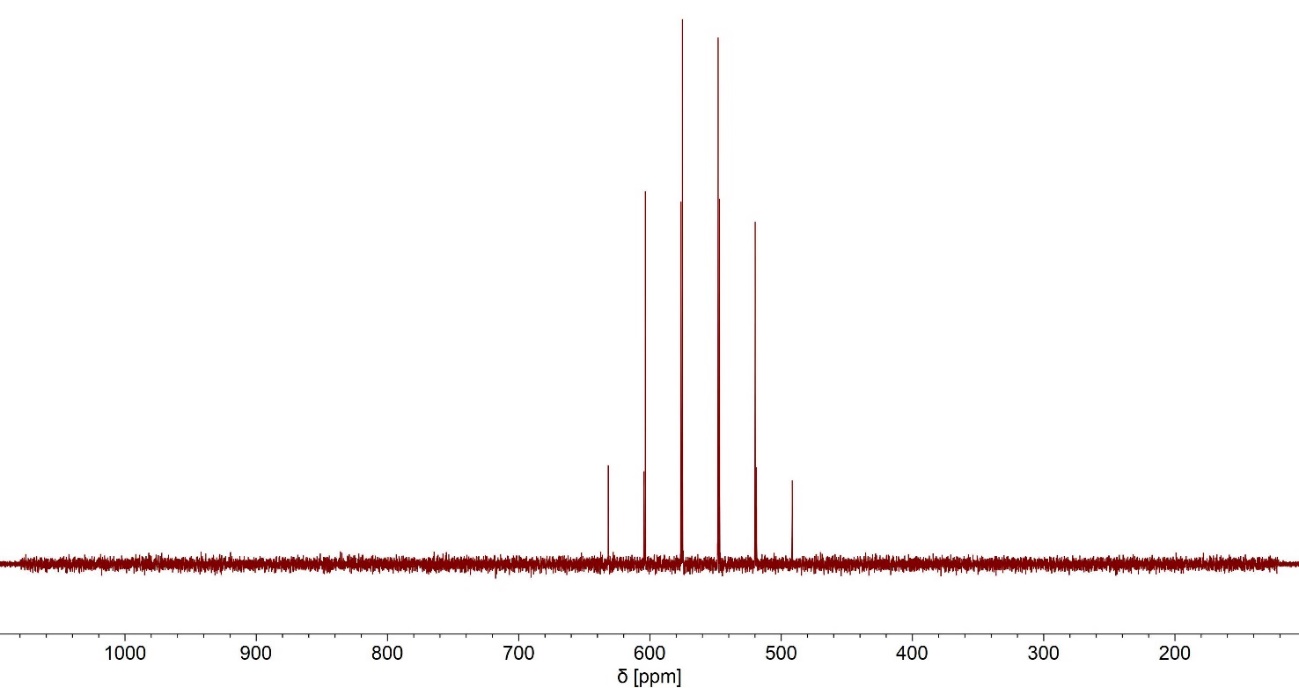


Figure S 23: ^125^Te NMR spectrum (126 MHz, neat, external lock acetone-d6, 16 °C) of Me_2_PrSiOTeF_5_.

## NMR spectra of Me_2_PrSiOTeF_5_ with AgOTeF_5_ (1c)


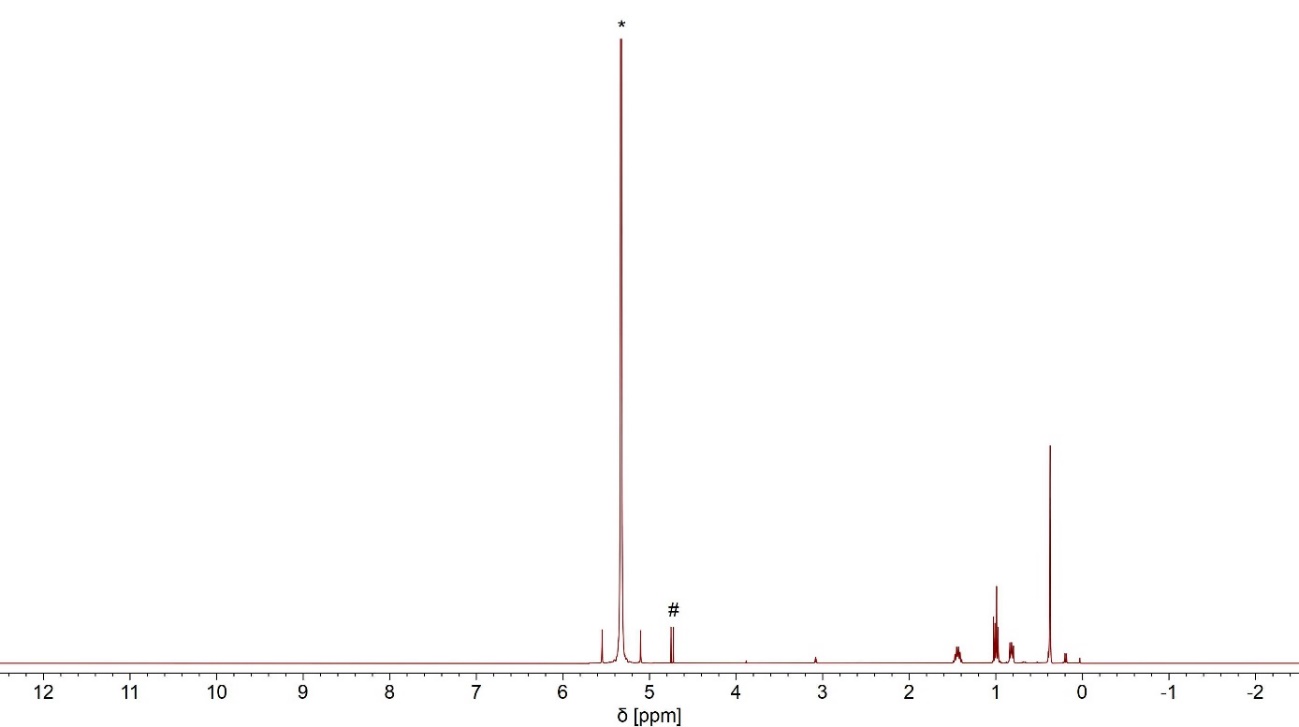


Figure S 24: ^1^H NMR spectrum (401 MHz, CH_2_Cl_2_, external lock acetone-d6, 18 °C) of Me_2_PrSiOTeF_5_ (*: solvent, #: ext. (CH_3_O)_3_PO).


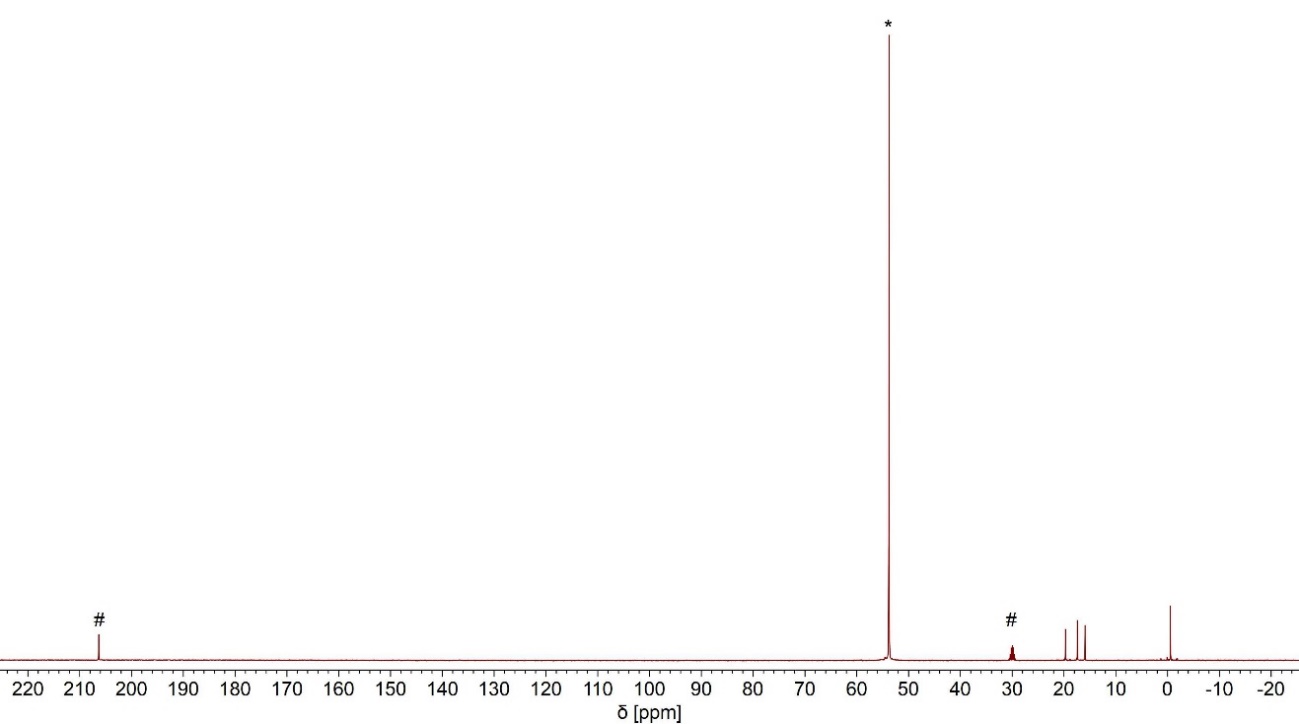


Figure S 25: ^13^C NMR spectrum (101 MHz, CH_2_Cl_2_, external lock acetone-d6, 17 °C) of Me_2_PrSiOTeF_5_ (*: solvent, #: ext. (CD_3_)_2_CO).


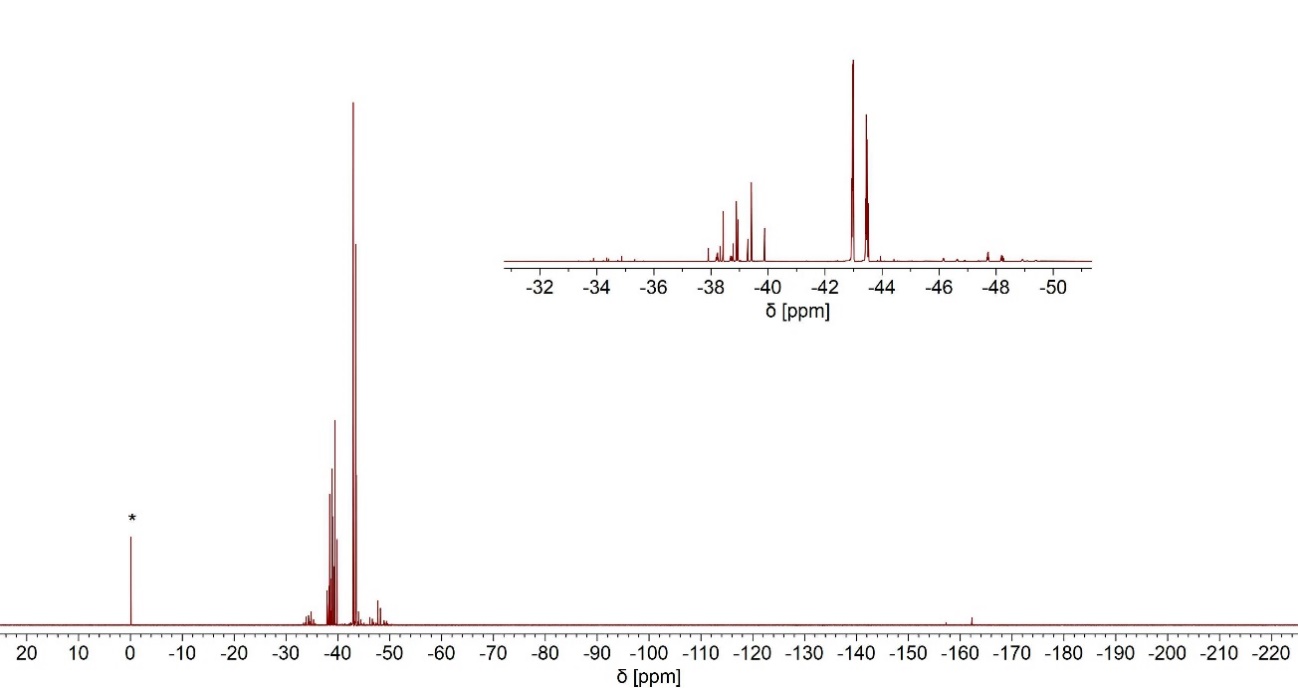


Figure S 26: ^19^F NMR spectrum (377 MHz, CH_2_Cl_2_, external lock acetone-d6, 18 °C) of Me_2_PrSiOTeF_5_ (*: ext. CFCl_3_).


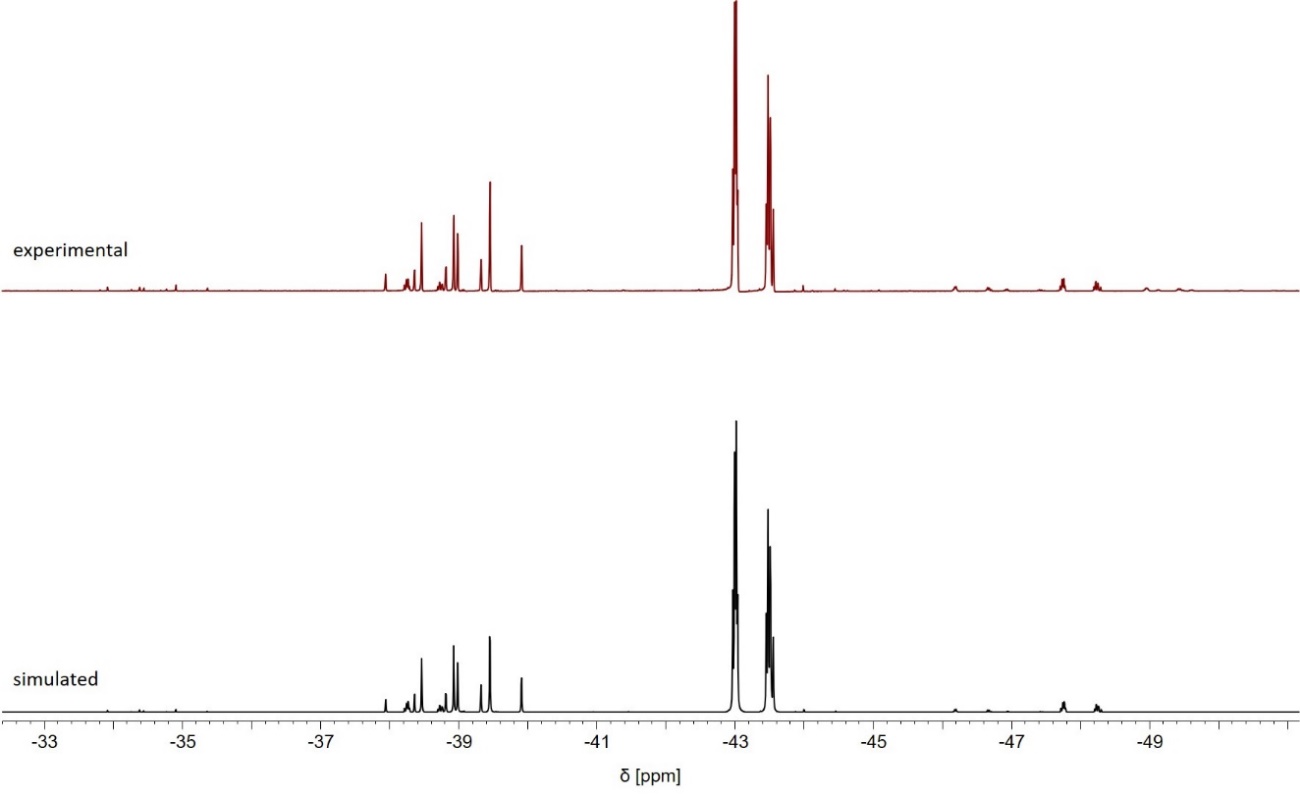


Figure S 27: Experimental (top) ^19^F NMR spectrum (377 MHz, CH_2_Cl_2_, external lock acetone-d6, 18 °C) and simulated (bottom) ^19^F NMR spectrum of Me_2_PrSiOTeF_5_.


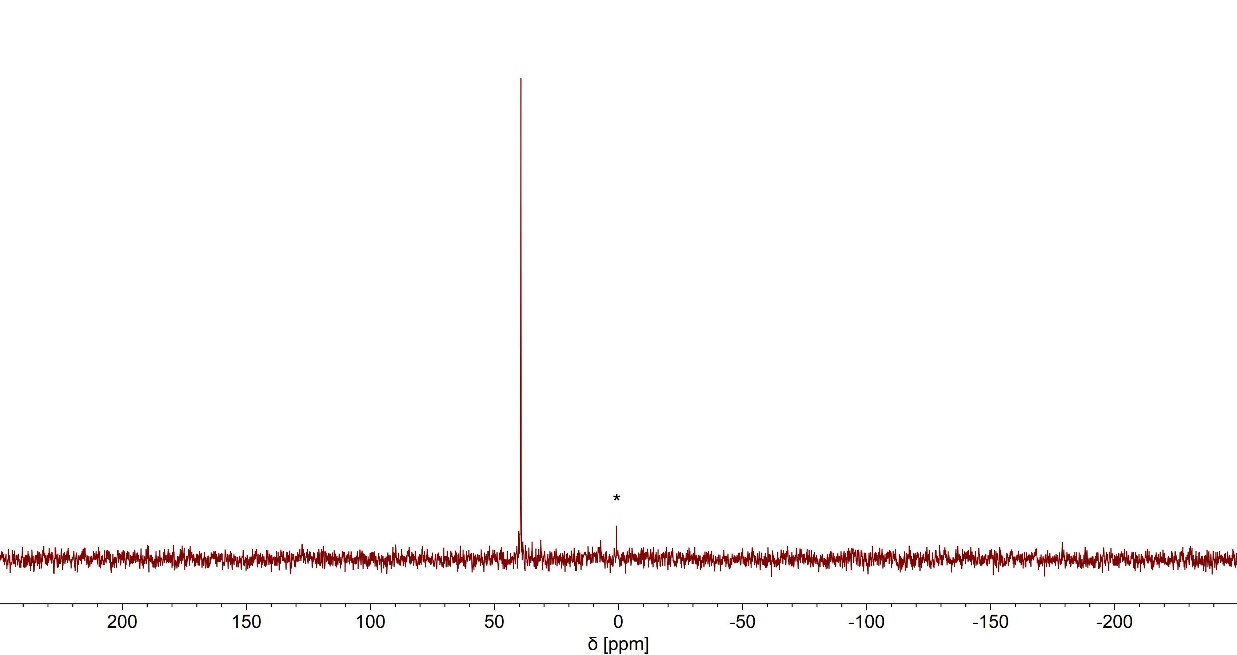


Figure S 28: ^29^Si DEPT NMR spectrum (80 MHz, CH_2_Cl_2_, external lock acetone-d6, 18 °C) of Me_2_PrSiOTeF_5_ (*: ext. Si(CH_3_)_4_).

## NMR spectra of *^i^*Pr_3_SiOTeF_5_ with HOTeF_5_ (1d)


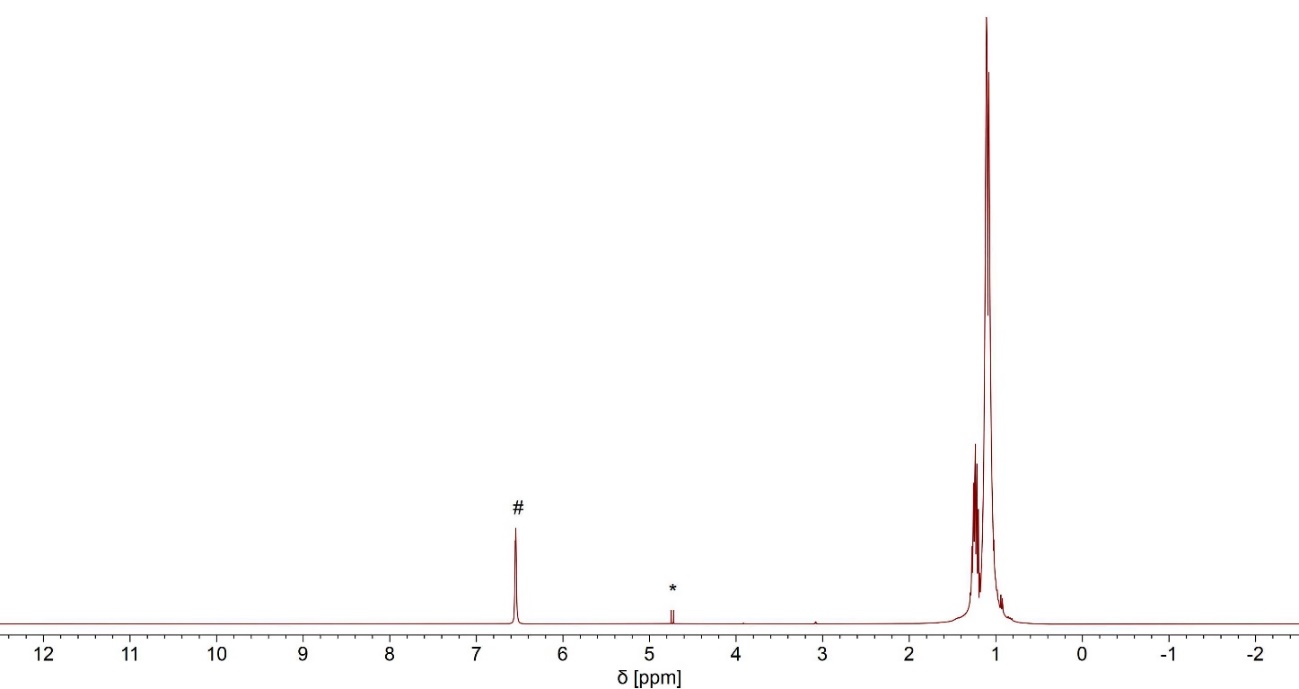


Figure S 29: ^1^H NMR spectrum (401 MHz, neat, external lock acetone-d6, 17 °C) of ^i^Pr_3_SiOTeF_5_ (*: ext. (CH_3_O)_3_PO, #: HCl).


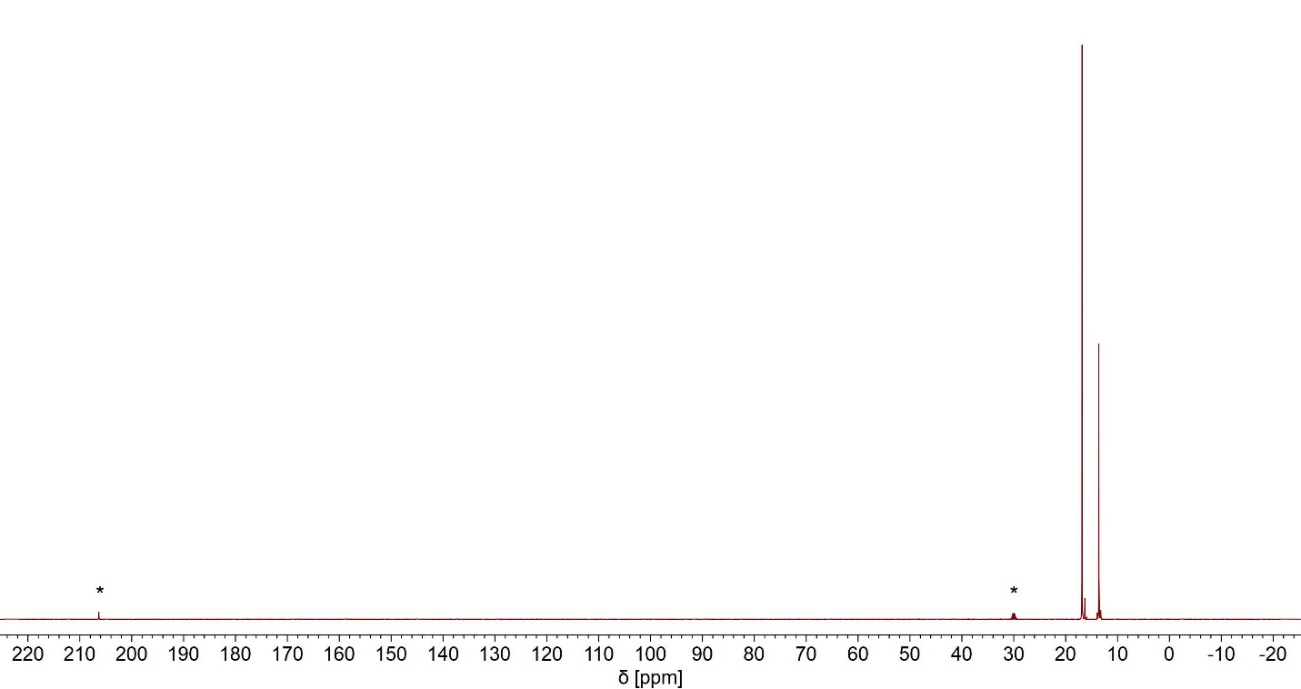


Figure S 30: ^13^C NMR spectrum (101 MHz, neat, external lock acetone-d6, 17 °C) of ^i^Pr_3_SiOTeF_5_ (*: ext. (CD_3_)_2_CO).


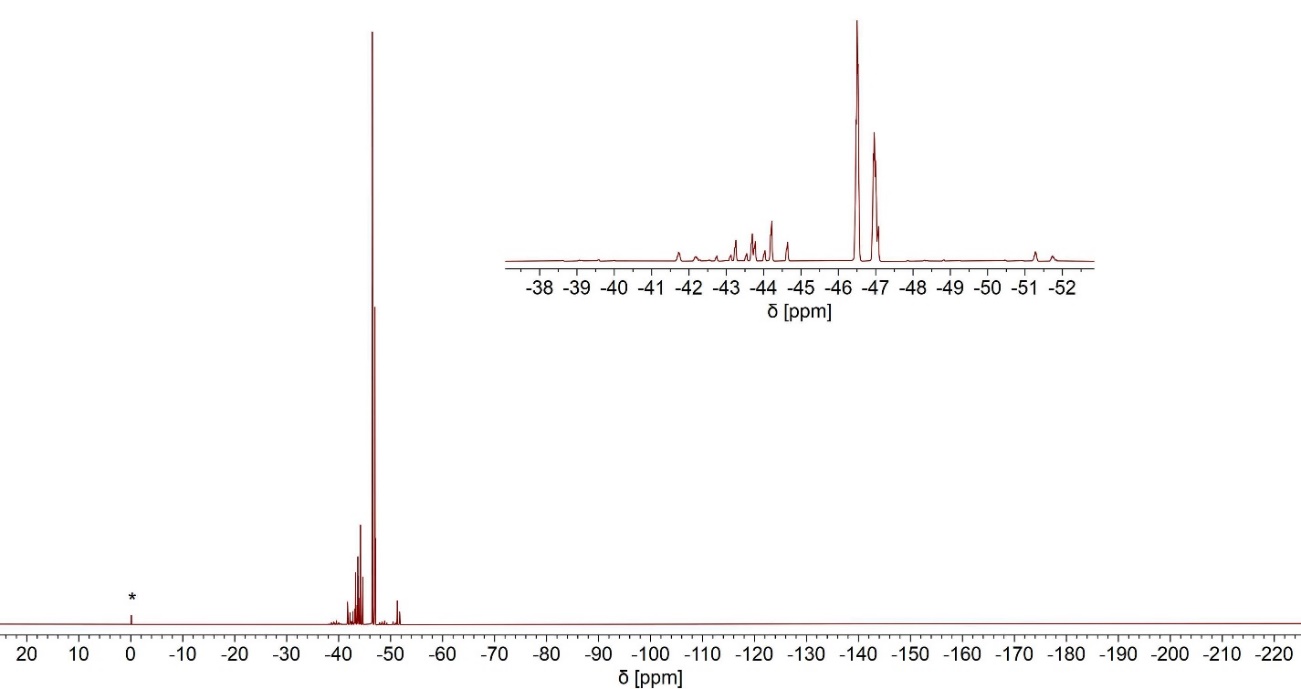


Figure S 31: ^19^F NMR spectrum (377 MHz, neat, external lock acetone-d6, 18 °C) of ^i^Pr_3_SiOTeF_5_ (*: ext. CFCl_3_).


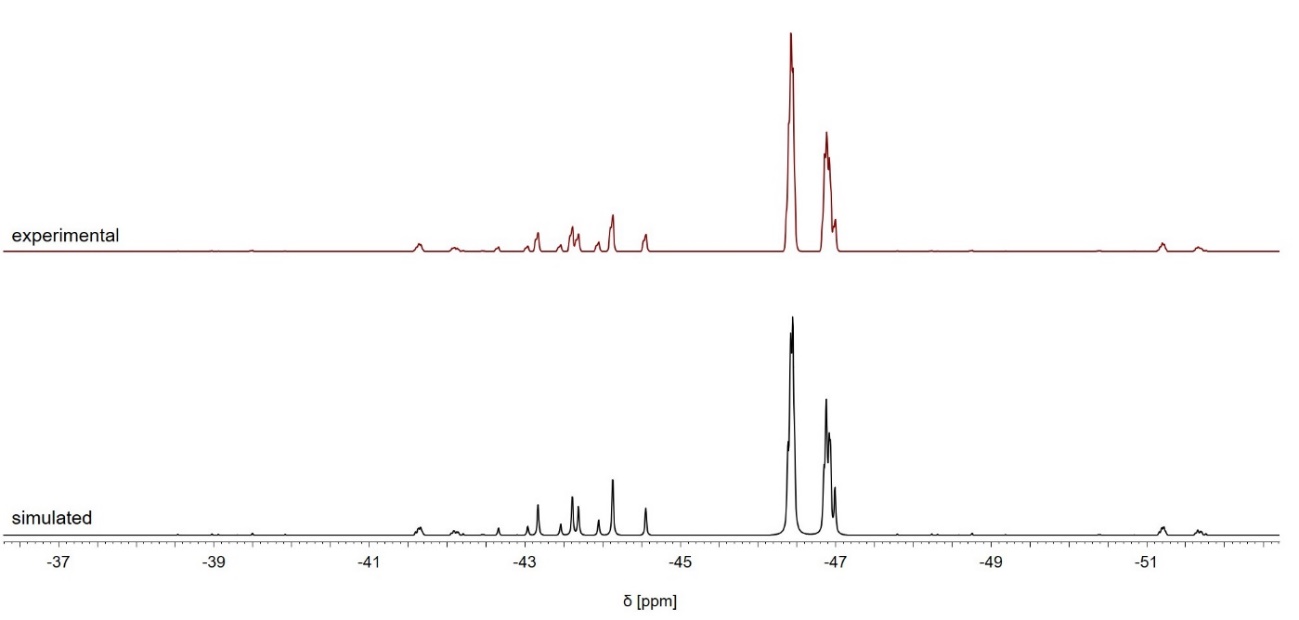


Figure S 32: Experimental (top) ^19^F NMR spectrum (377 MHz, neat, external lock acetone-d6, 18 °C) and simulated (bottom) ^19^F NMR spectrum of ^i^Pr_3_SiOTeF_5_.


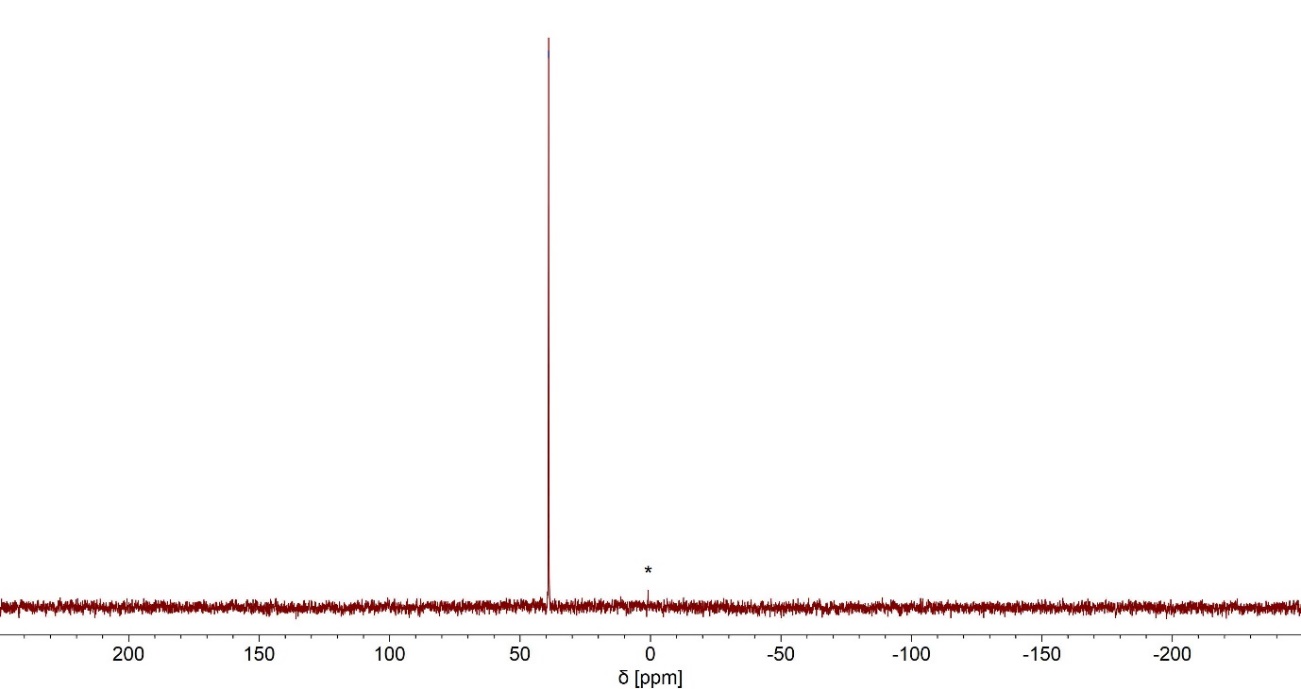


Figure S 33: ^29^Si DEPT NMR spectrum (101 MHz, neat, external lock acetone-d6, 17 °C) of ^i^Pr_3_SiOTeF_5_ (*: ext. Si(CH_3_)_4_).


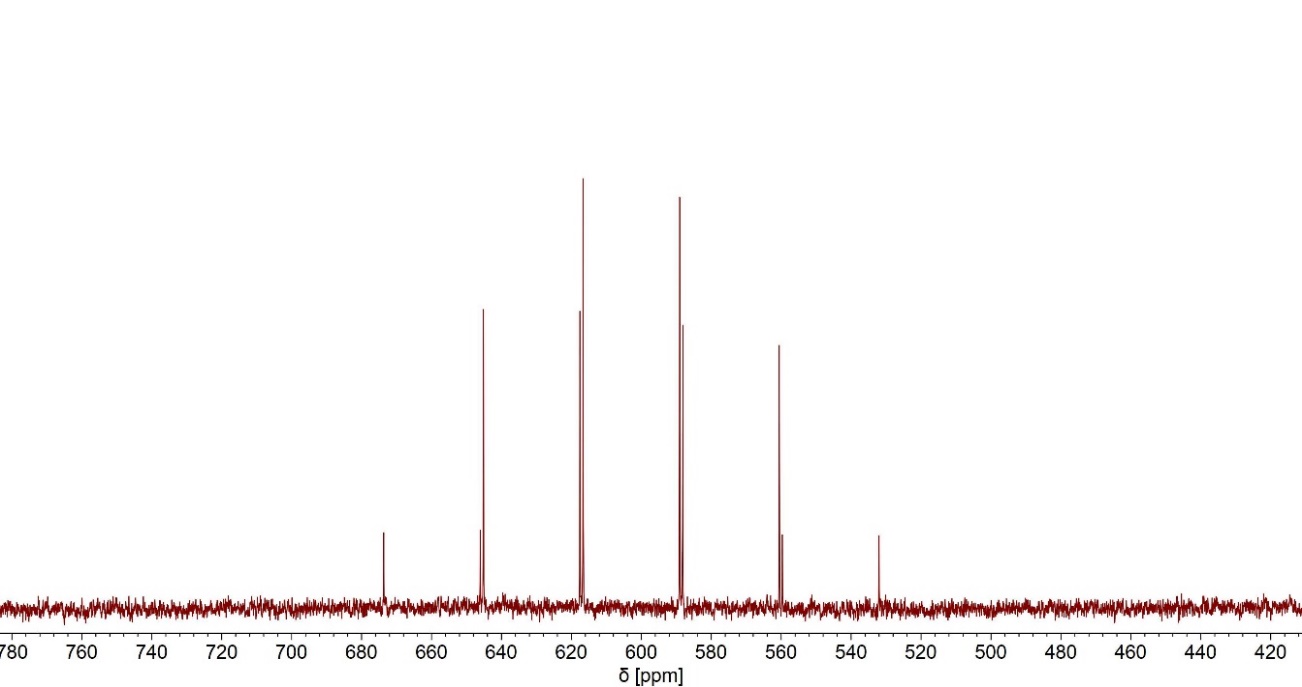


Figure S 34: ^125^Te NMR spectrum (126 MHz, neat, external lock acetone-d6, 17 °C) of ^i^Pr_3_SiOTeF_5_.

## NMR spectra of *^i^*Pr_3_SiOTeF_5_ with AgOTeF_5_ (1d)


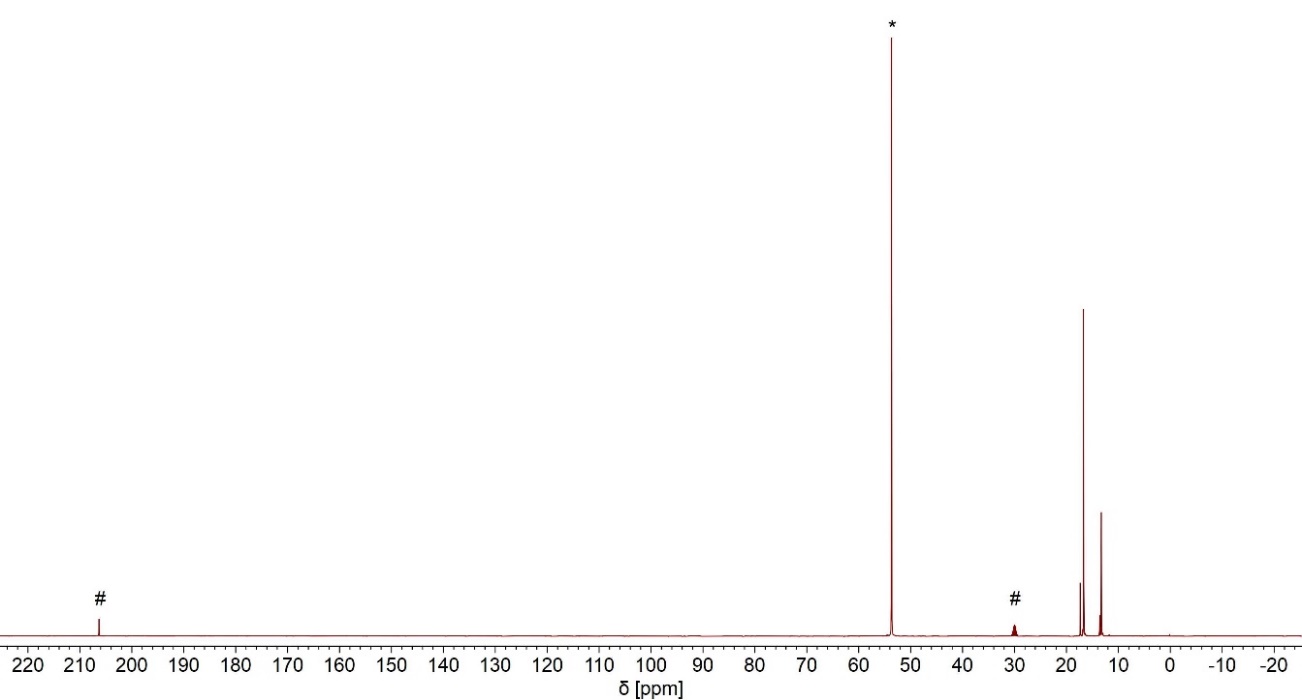


Figure S 35: ^13^C NMR spectrum (101 MHz, CH_2_Cl_2_, external lock acetone-d6, 18 °C) of ^i^Pr_3_SiOTeF_5_ (*: solvent, #: ext. (CD_3_)_2_CO).


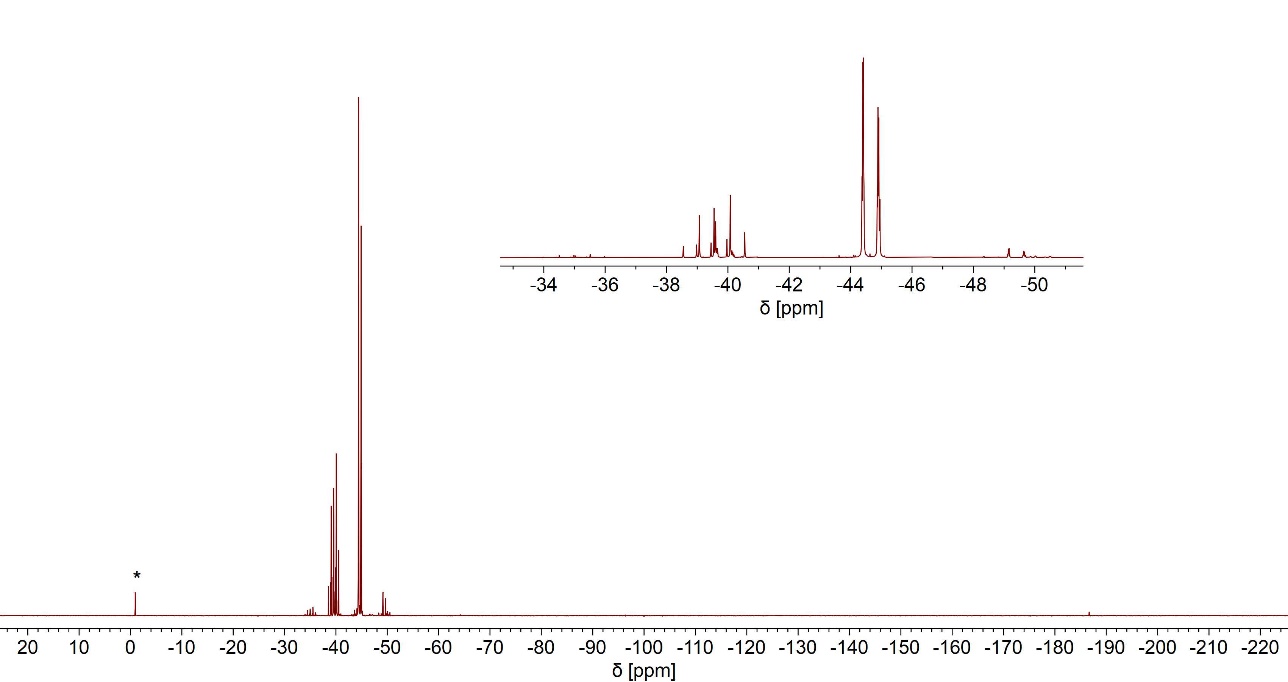


Figure S 36: ^19^F NMR spectrum (376 MHz, neat, external lock acetone-d6, 18 °C) of ^i^Pr_3_SiOTeF_5_ (*: ext. CFCl_3_).


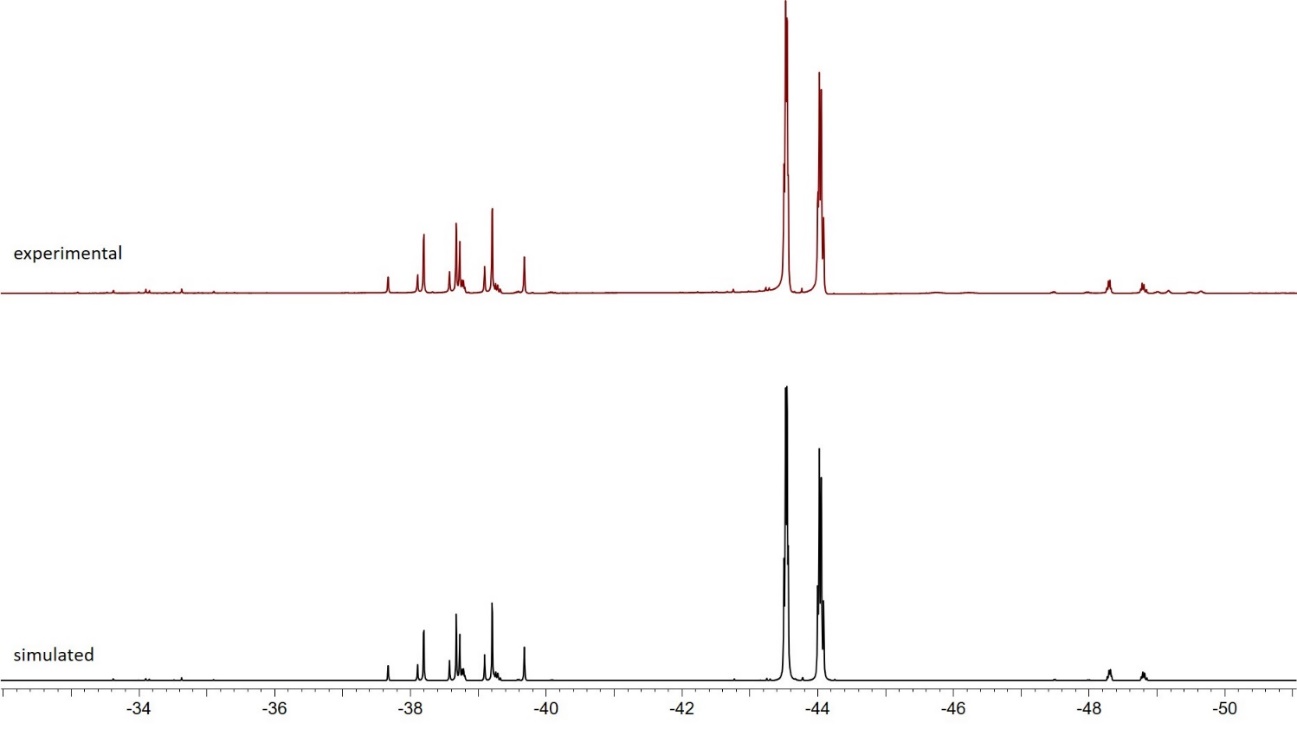


Figure S 37: Experimental (top) ^19^F NMR spectrum (376 MHz, neat, external lock acetone-d6, 18 °C) and simulated (bottom) ^19^F NMR spectrum of ^i^Pr_3_SiOTeF_5_.


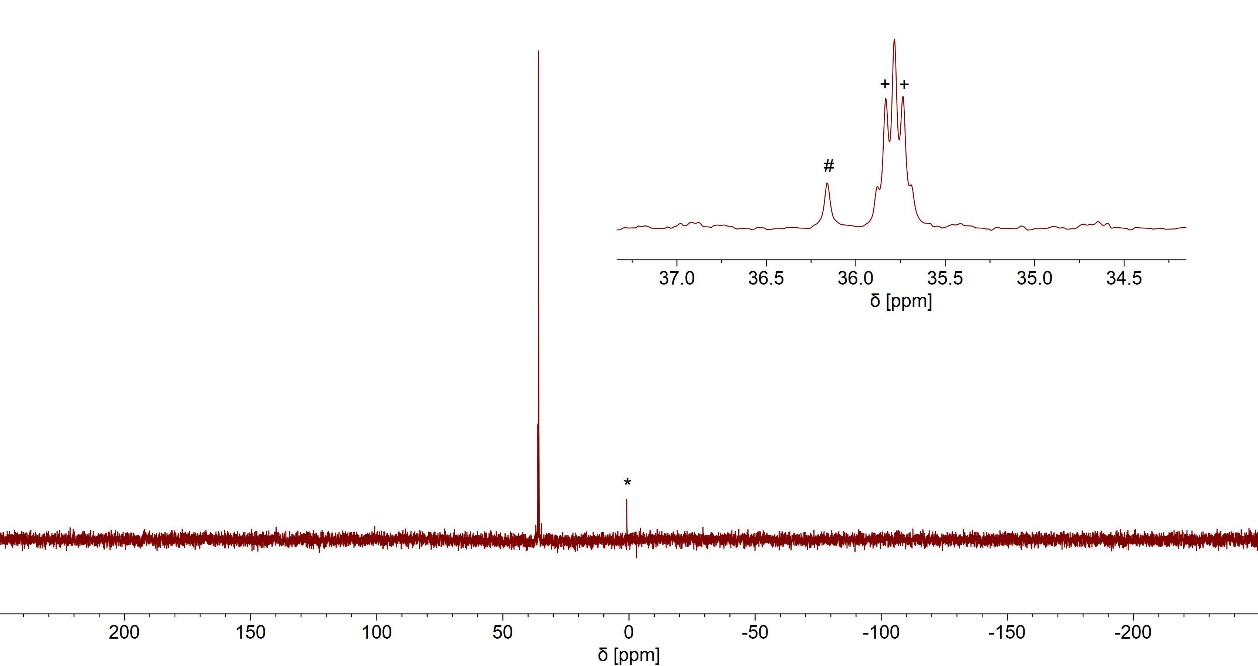


Figure S 38: ^29^Si DEPT NMR spectrum (80 MHz, CH_2_Cl_2_, external lock acetone-d6, 18 °C) of ^i^Pr_3_SiOTeF_5_ (*: ext. Si(CH_3_)_4_, #: ^i^Pr_3_SiCl). ^19^F Satellites are marked with a plus (+).


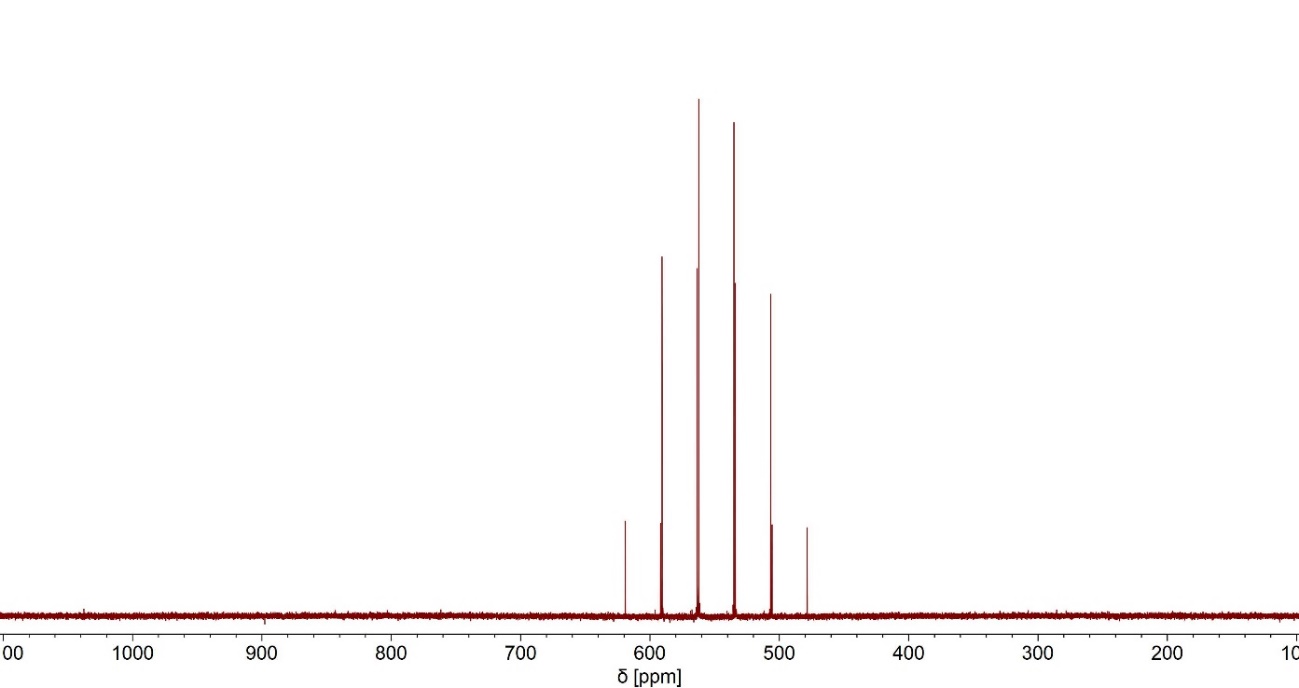


Figure S 39: ^125^Te NMR spectrum (126 MHz, CH_2_Cl_2_, external lock acetone-d6, 19 °C) of ^i^Pr_3_SiOTeF_5_.

## NMR spectra of *^t^*BuMe_2_SiOTeF_5_ with HOTeF_5_ (1e)


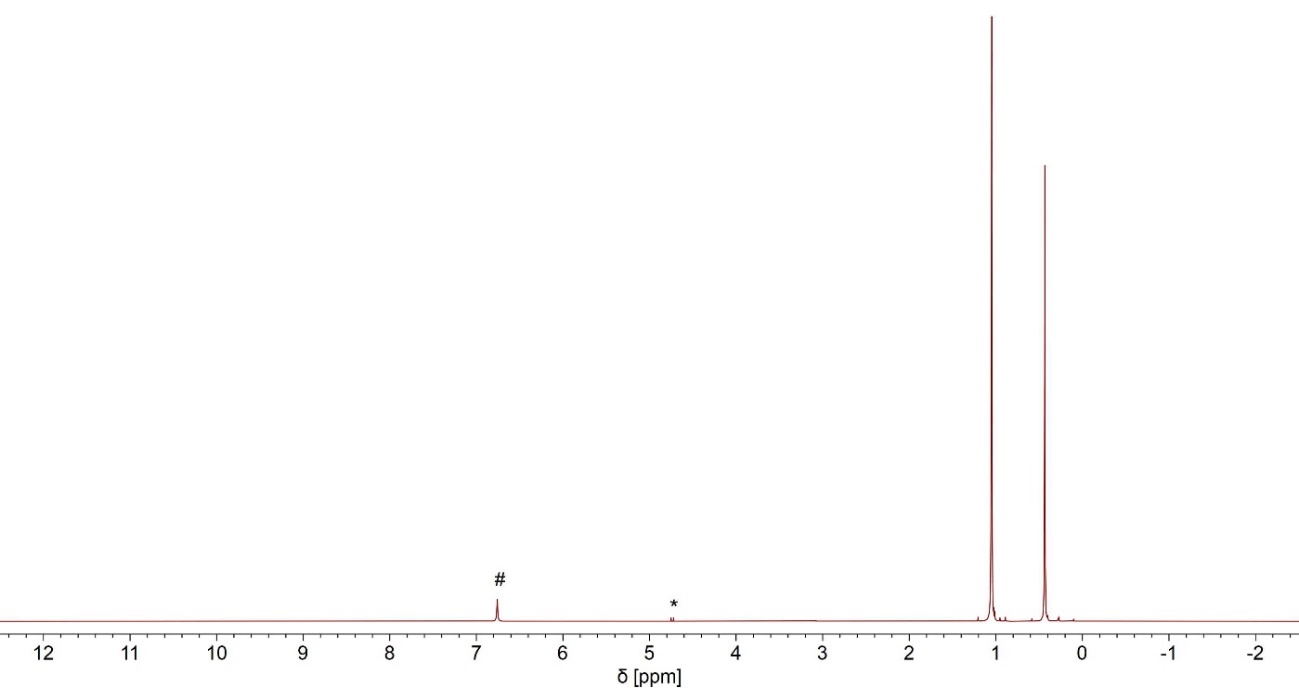


Figure S 40: ^1^H NMR spectrum (401 MHz, neat, external lock acetone-d6, 17 °C) of ^t^BuMe_2_SiOTeF_5_ (*: ext. (CH_3_O)_3_PO, #: HOTeF_5_).


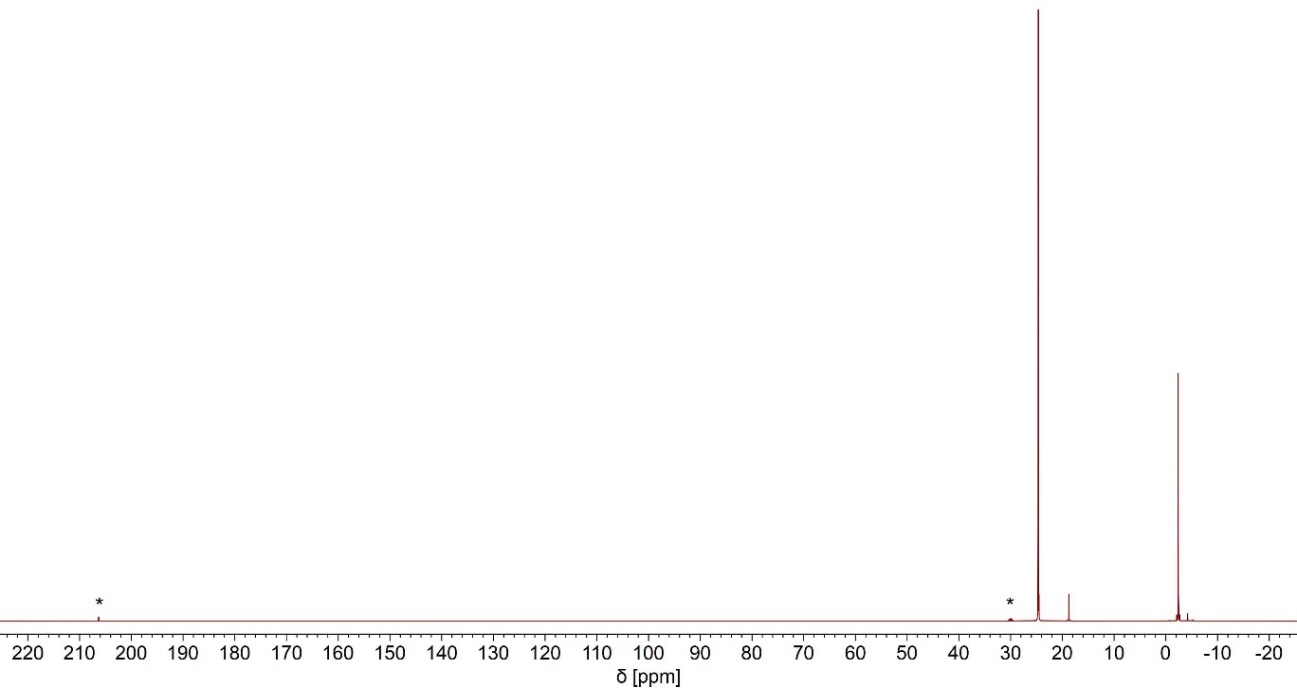


Figure S 41: ^13^C NMR spectrum (101 MHz, neat, external lock acetone-d6, 17 °C) of ^t^BuMe_2_SiOTeF_5_ (*: ext. (CD_3_)_2_CO).


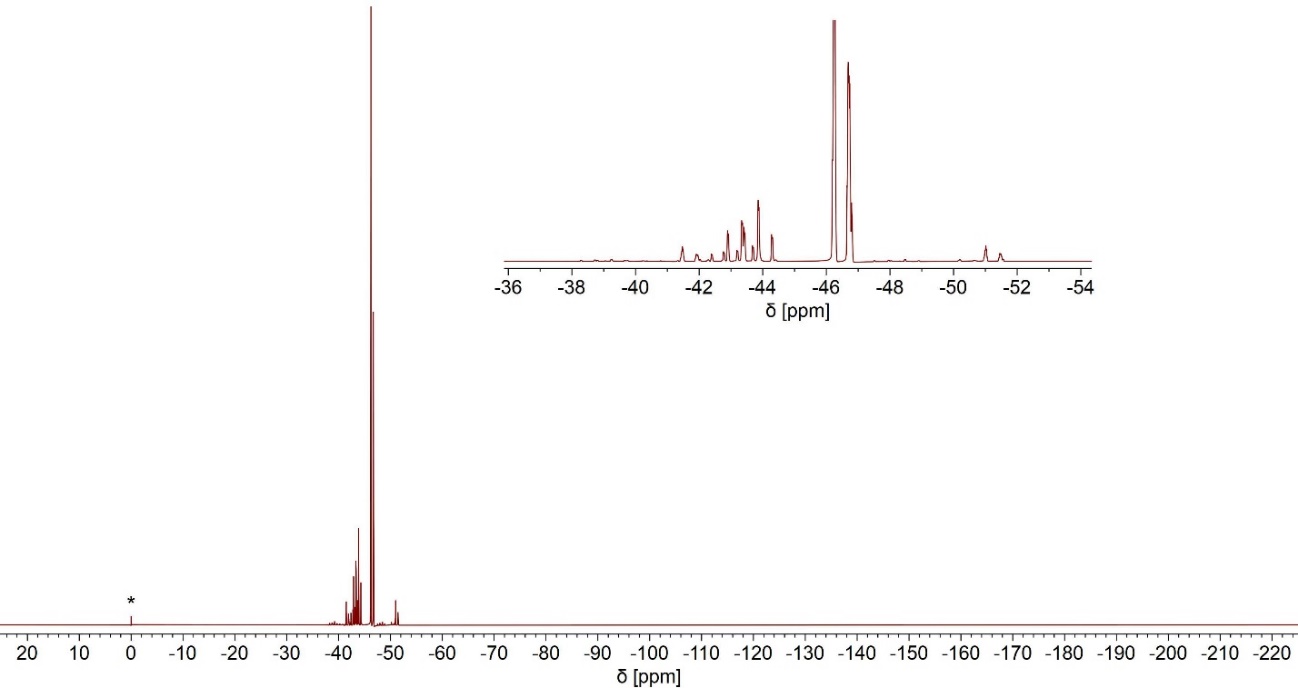


Figure S 42: ^19^F NMR spectrum (377 MHz, neat, external lock acetone-d6, 16 °C) of ^t^BuMe_2_SiOTeF_5_ (*: ext. CFCl_3_).


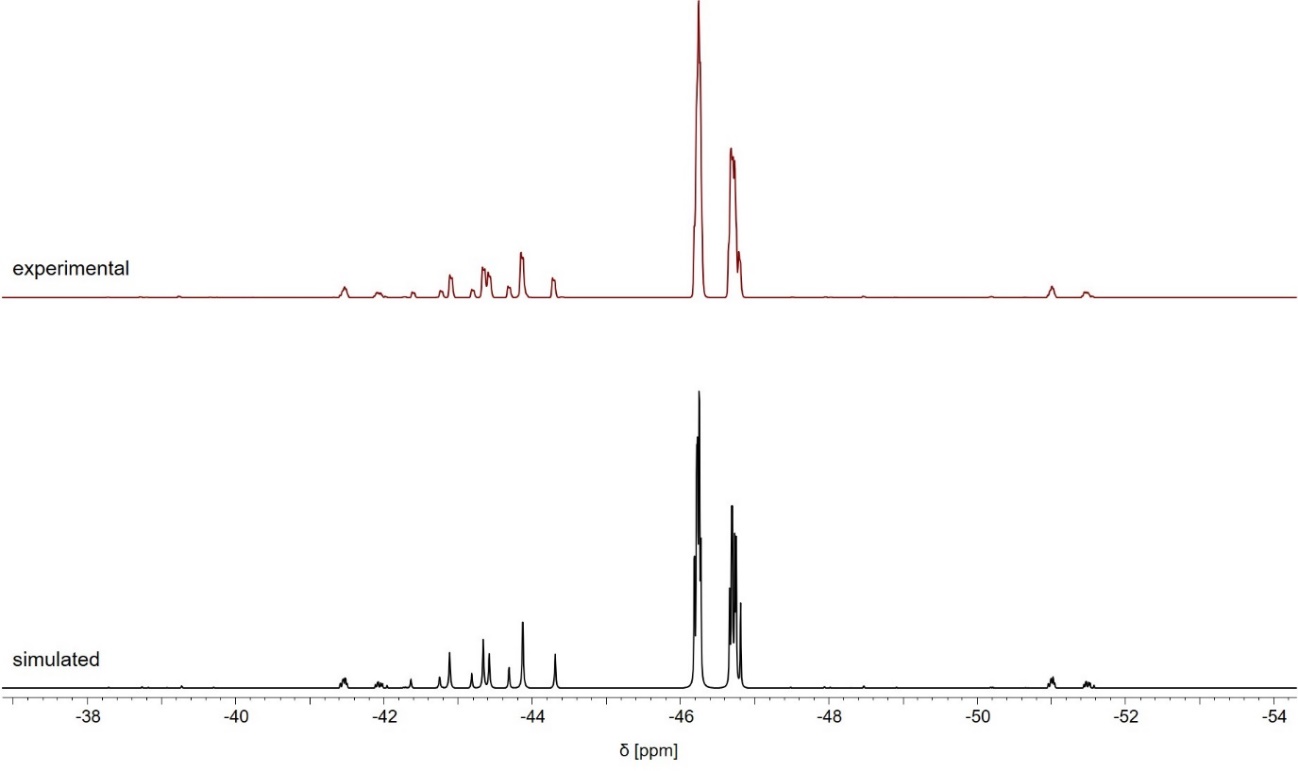


Figure S 43: Experimental (top) ^19^F NMR spectrum (377 MHz, neat, external lock acetone-d6, 16 °C) and simulated (bottom) ^19^F NMR spectrum of ^t^BuMe_2_SiOTeF_5_.


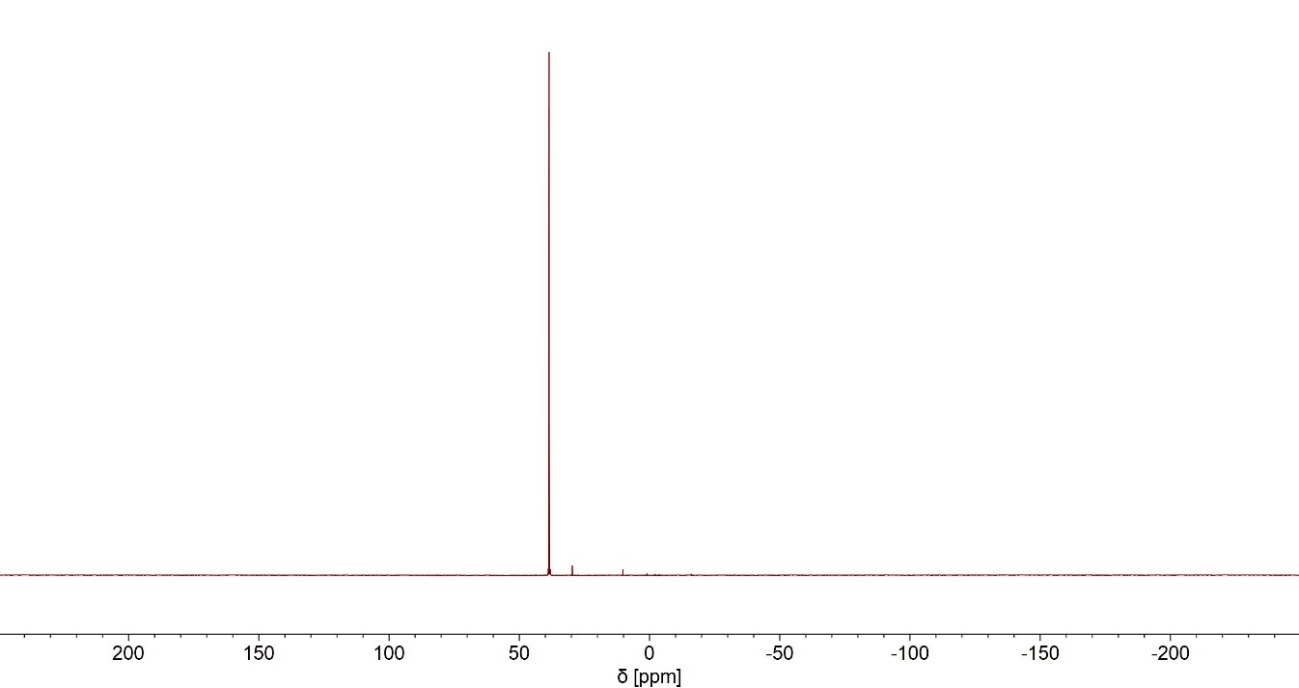


Figure S 44: ^29^Si DEPT NMR spectrum (80 MHz, neat, external lock acetone-d6, 17 °C) of ^t^BuMe_2_SiOTeF_5_.

## NMR spectra of *^t^*BuMe_2_SiOTeF_5_ with AgOTeF_5_ (1e)


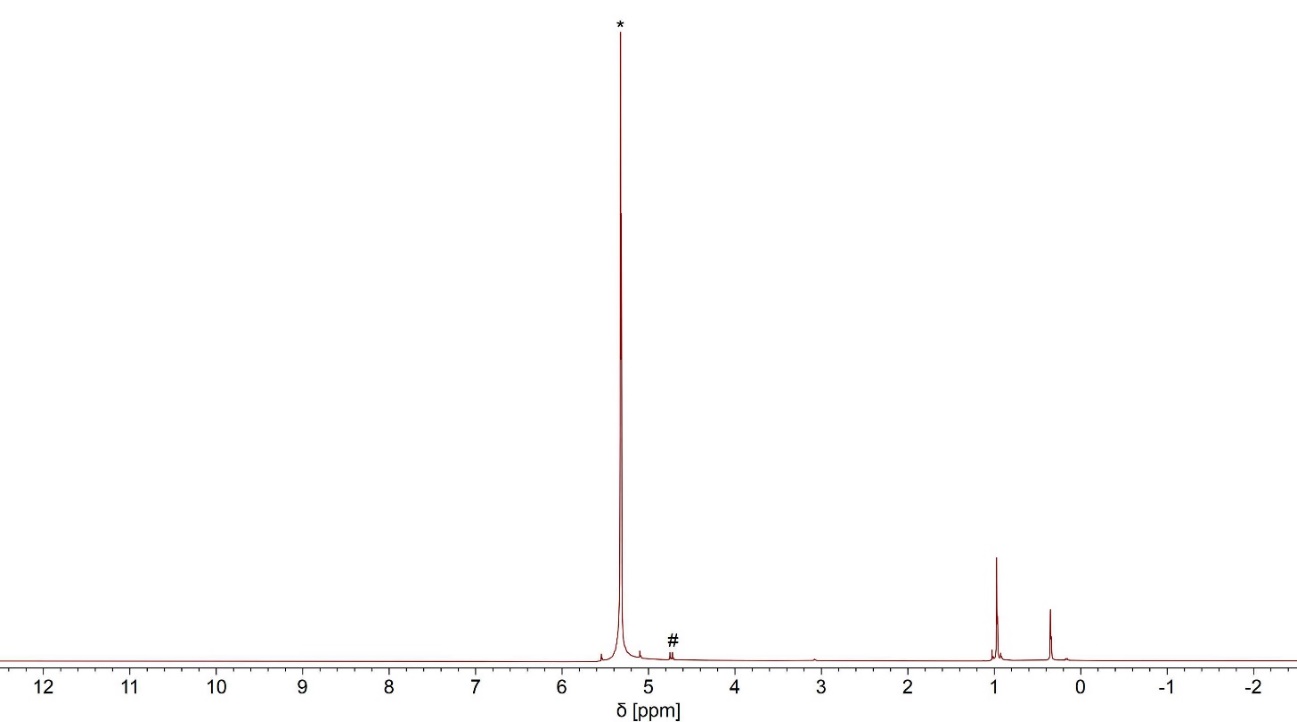


Figure S 45: ^1^H NMR spectrum (401 MHz, neat, external lock acetone-d6, 17 °C) of ^t^BuSiOTeF_5_ (*: solvent, #: ext. (CH_3_O)_3_PO).


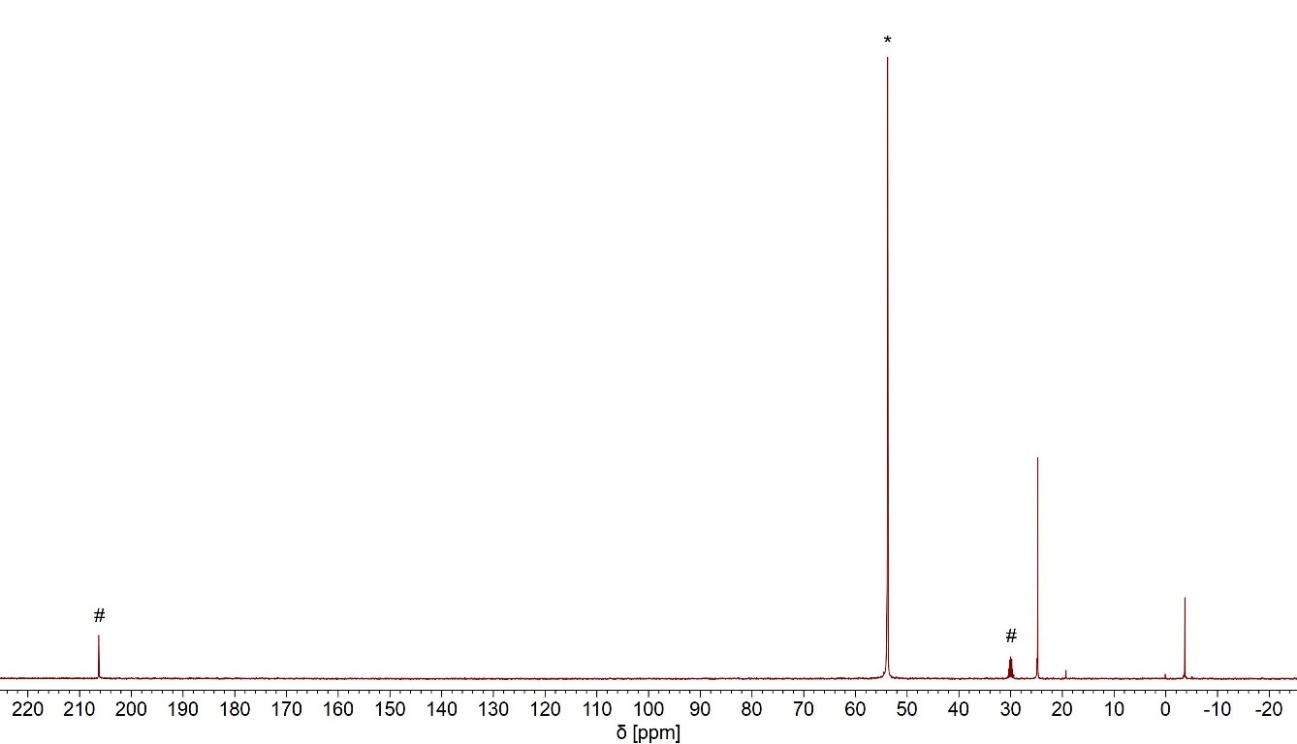


Figure S 46: ^13^C NMR spectrum (101 MHz, neat, external lock acetone-d6, 17 °C) of ^t^BuSiOTeF_5_ (*: solvent, #: ext. (CD_3_)_2_CO).


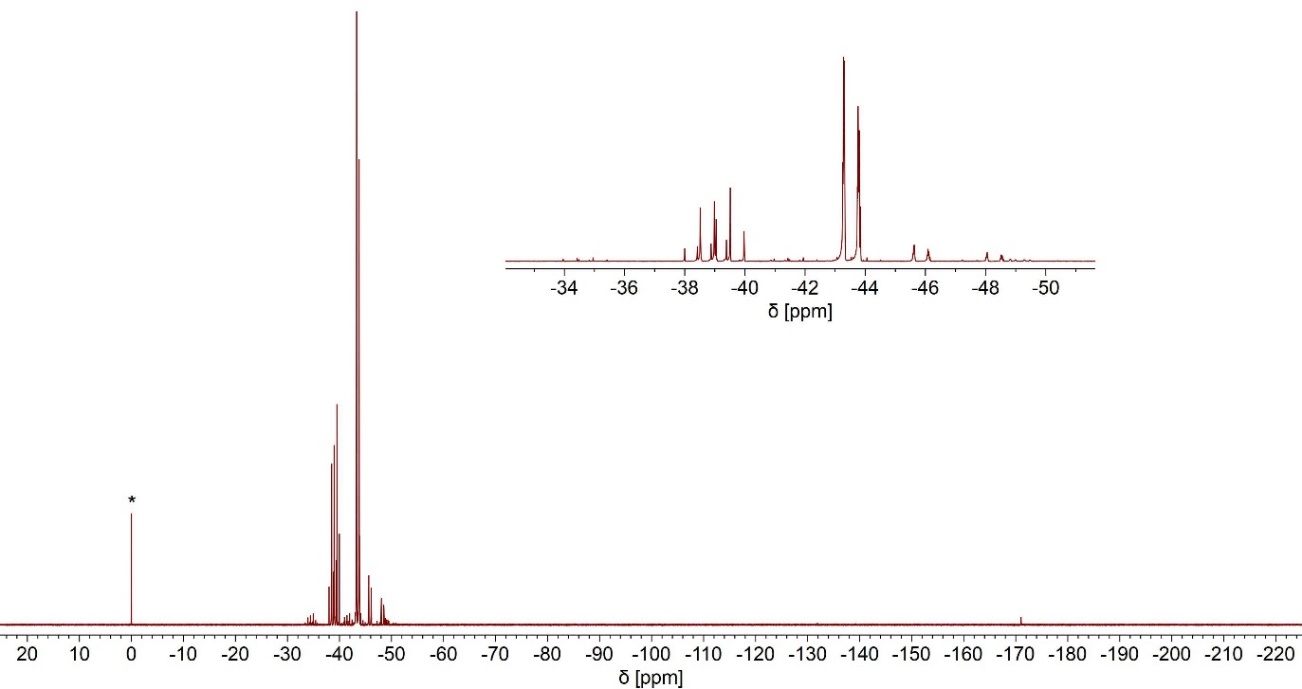


Figure S 47: ^19^F NMR spectrum (377 MHz, neat, external lock acetone-d6, 16 °C) of ^t^BuSiOTeF_5_ (*: ext. CFCl_3_).


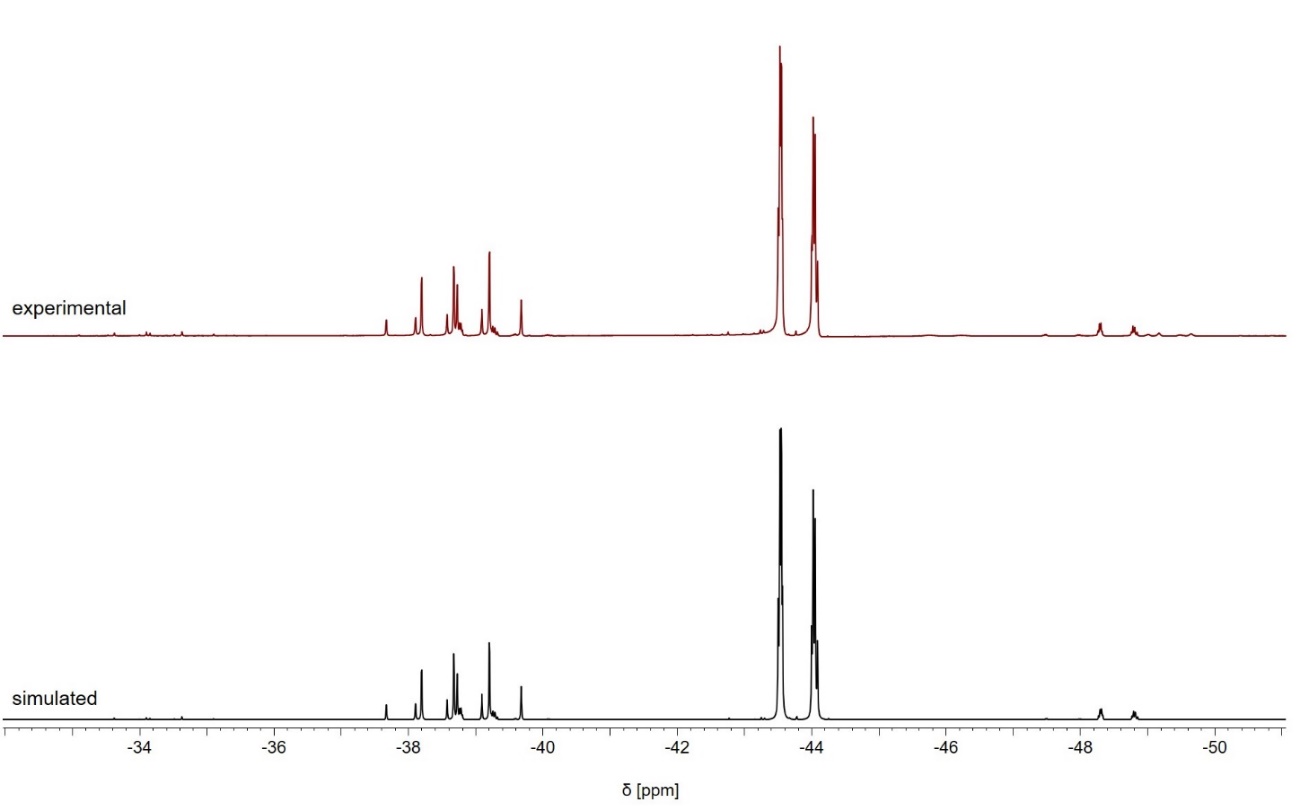


Figure S 48: Experimental (top) ^19^F NMR spectrum (377 MHz, neat, external lock acetone-d6, 16 °C) and simulated (bottom) ^19^F NMR spectrum of ^t^BuSiOTeF_5_.


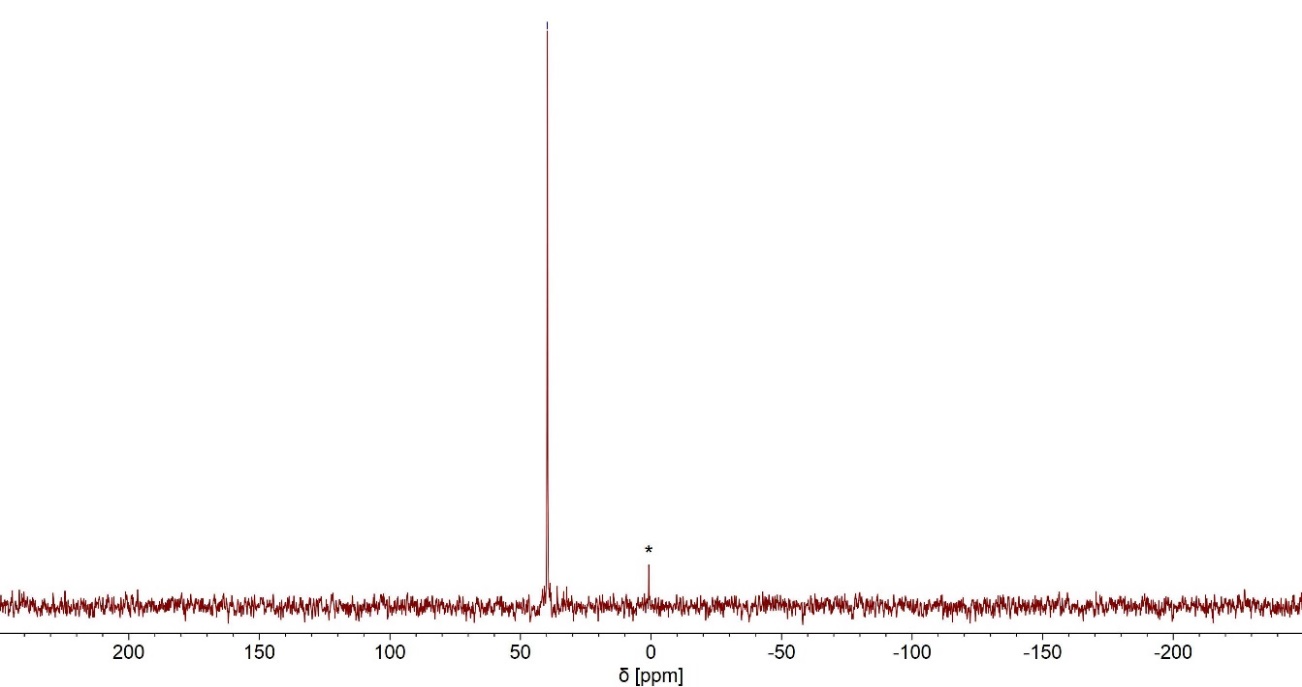


Figure S 49: ^29^Si DEPT NMR spectrum (80 MHz, neat, external lock acetone-d6, 17 °C) of ^t^BuSiOTeF_5_ (*: ext. Si(CH_3_)_4_).

## NMR spectra of Ph_3_SiOTeF_5_ with HOTeF_5_ (1f)


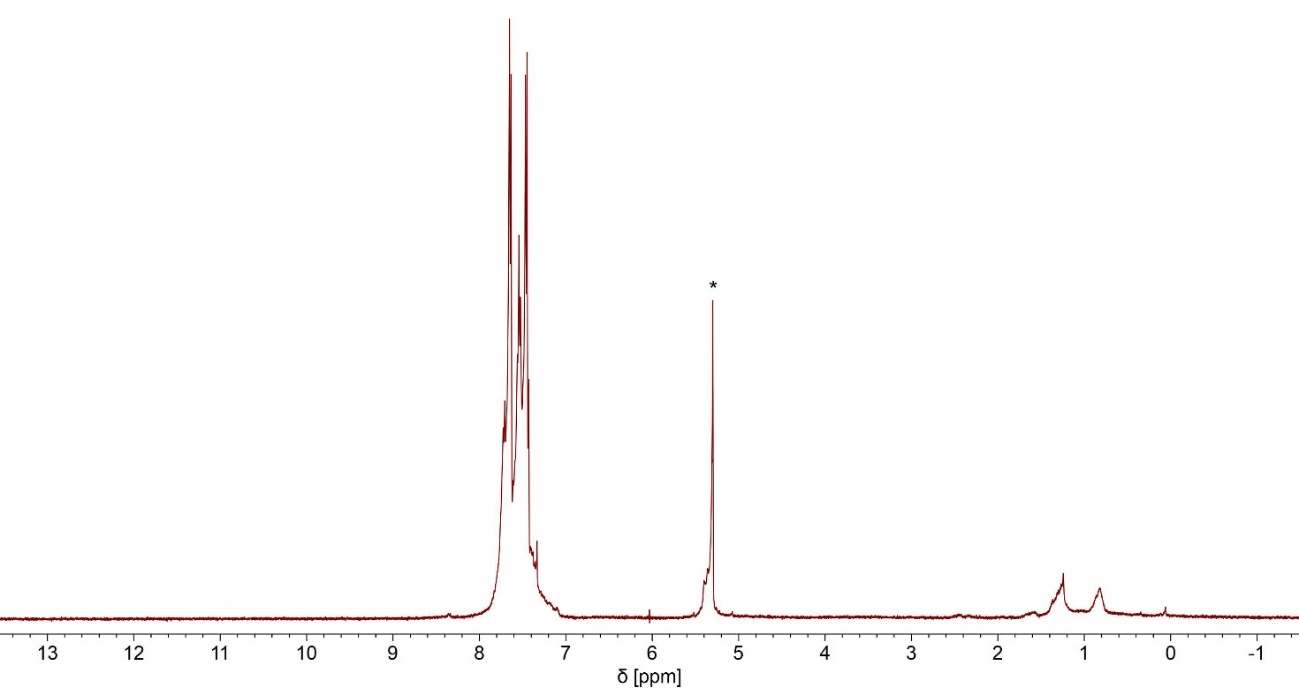


Figure S 50: ^1^H NMR spectrum (401 MHz, CD_2_Cl_2_, 18 °C) of Ph_3_SiOTeF_5_ (*: solvent).


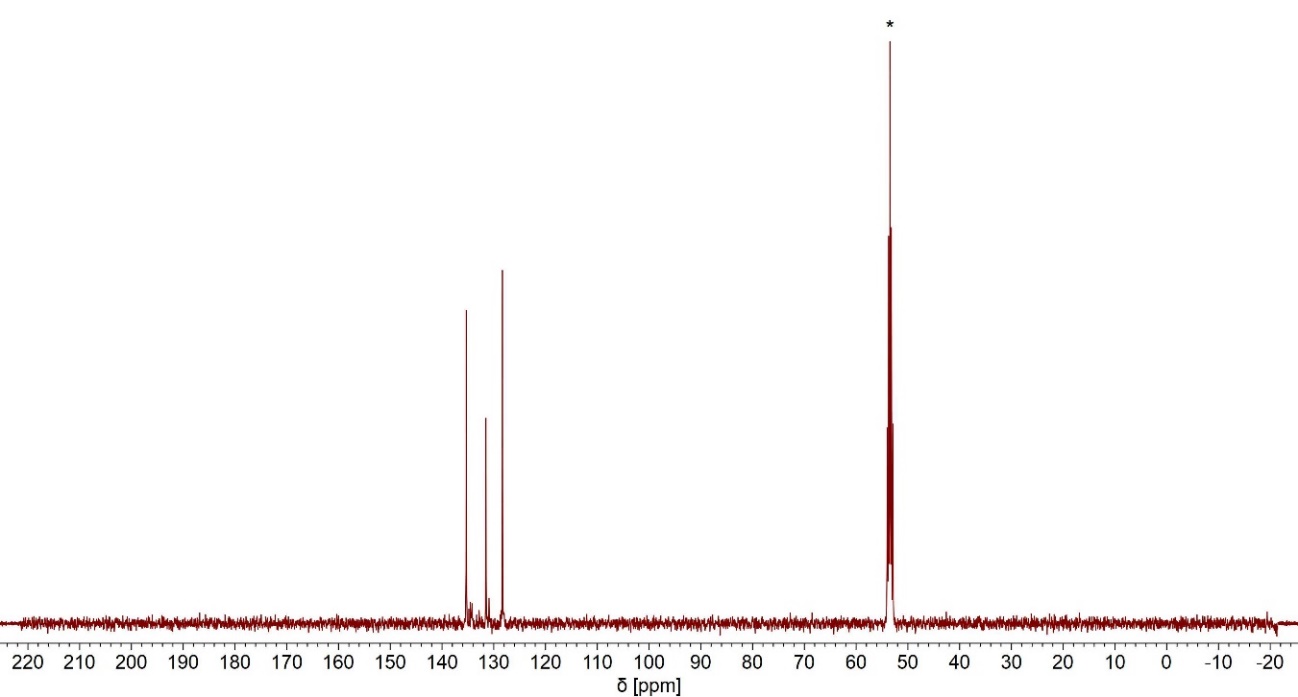


Figure S 51: ^13^C NMR spectrum (101 MHz, CD_2_Cl_2_, 18 °C) of Ph_3_SiOTeF_5_ (*: solvent).


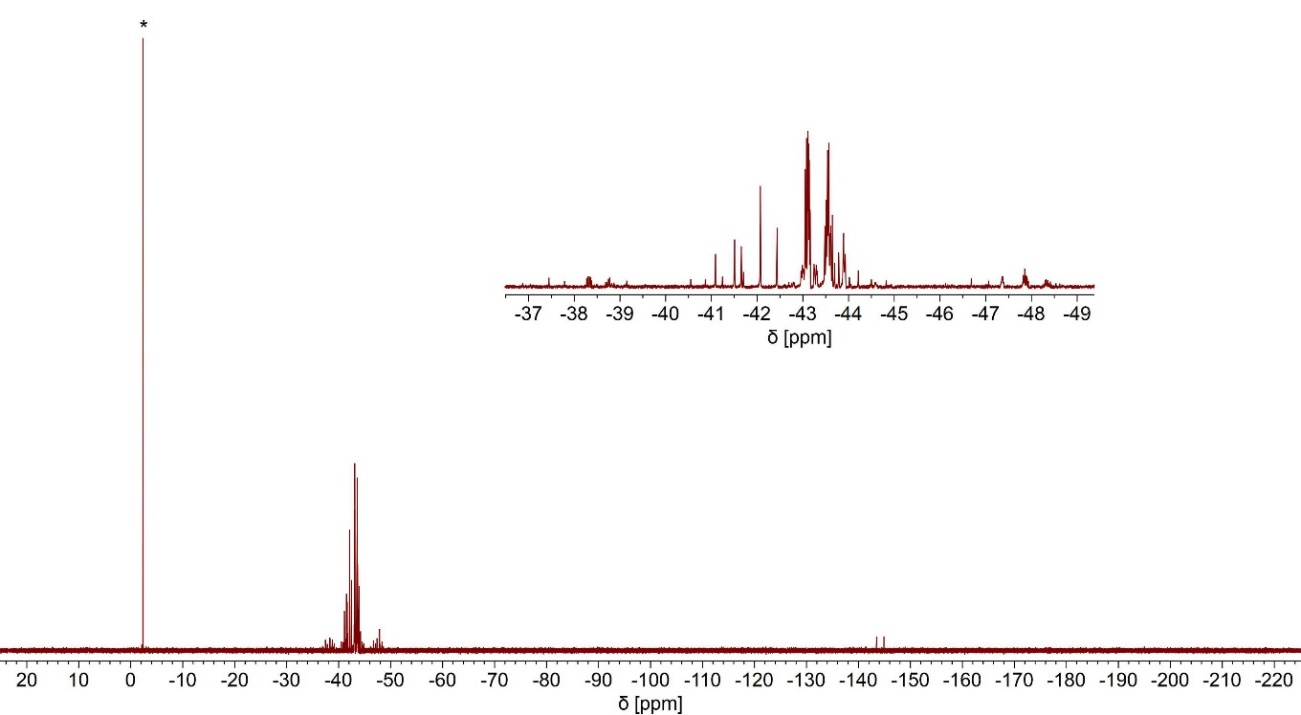


Figure S 52: ^19^F NMR spectrum (377 MHz, neat, external lock acetone-d6, 16 °C) of Ph_3_SiOTeF_5_ (*: ext. CFCl_3_).


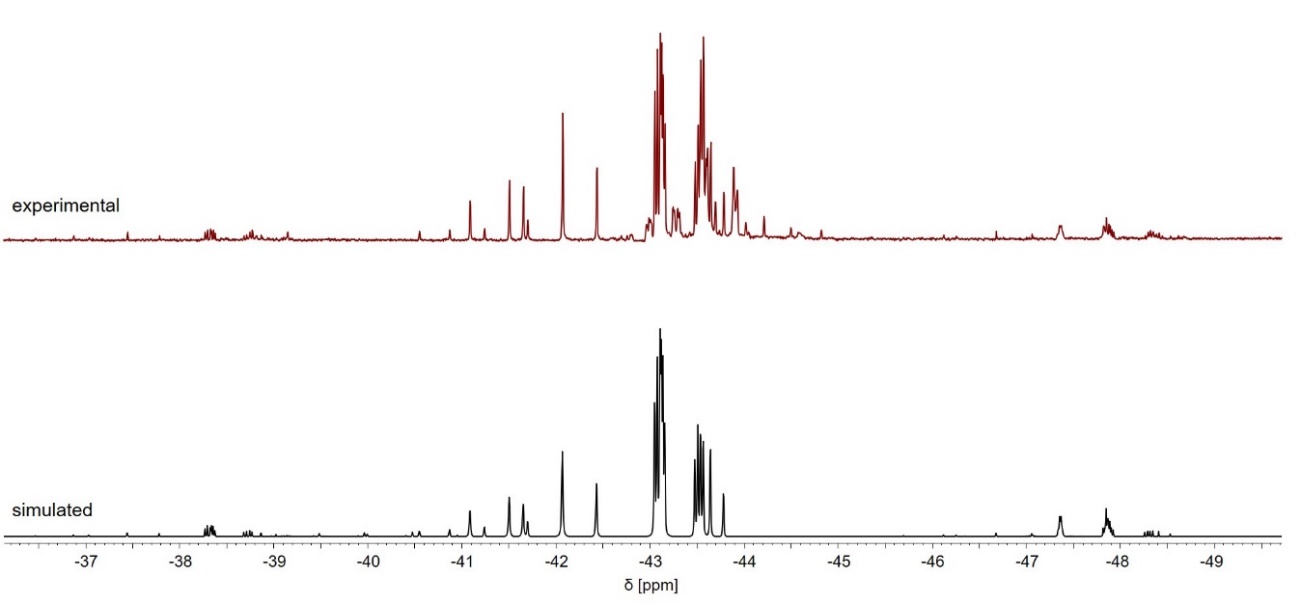


Figure S 53: Experimental (top) ^19^F NMR spectrum (377 MHz, neat, external lock acetone-d6, 16 °C) and simulated (bottom) ^19^F NMR spectrum of Ph_3_SiOTeF_5_.


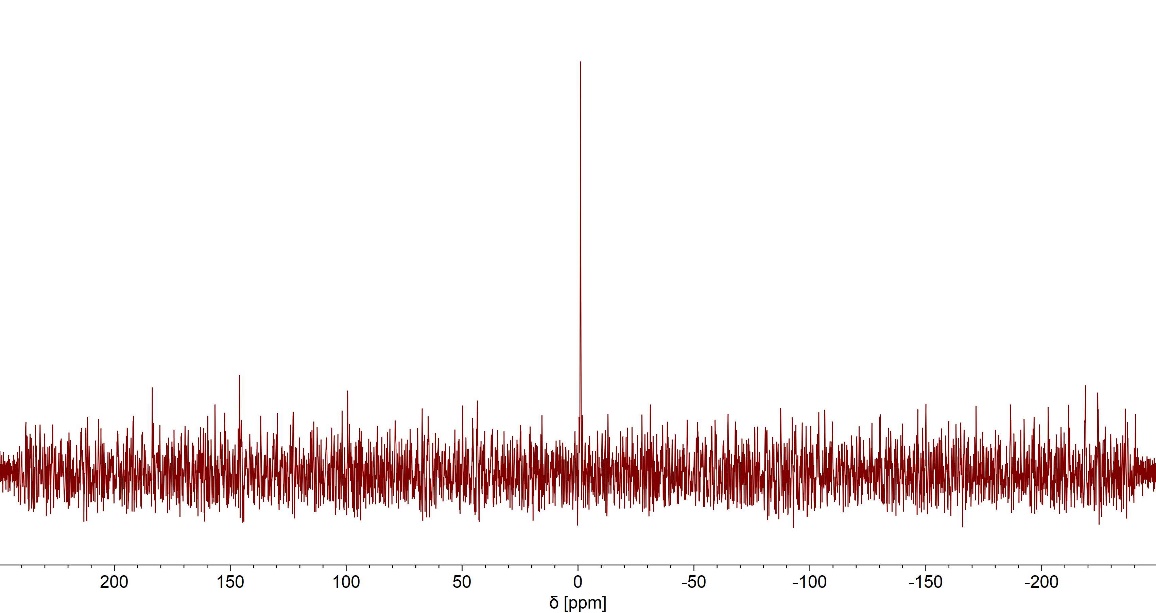


Figure S 54: ^29^Si DEPT NMR spectrum (80 MHz, CD_2_Cl_2_, 18 °C) of Ph_3_SiOTeF_5_.

## NMR spectra of Ph_3_SiOTeF_5_ with AgOTeF_5_ (1f)


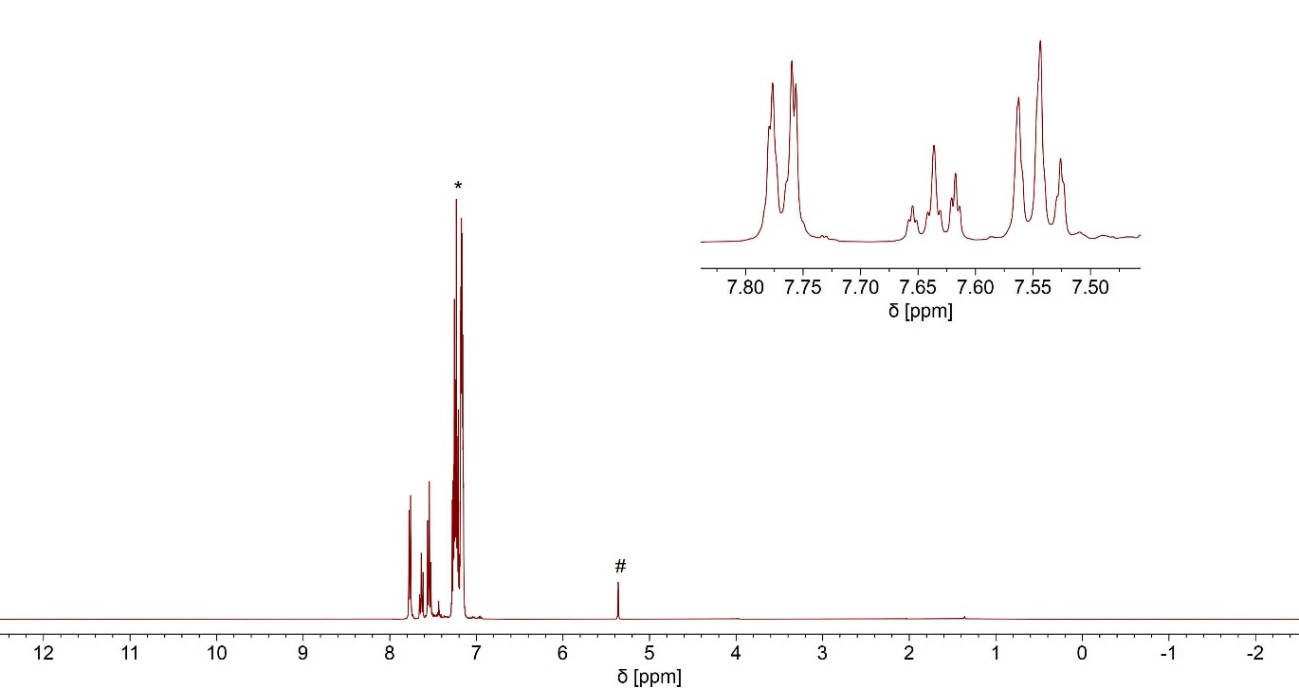


Figure S 55: ^1^H NMR spectrum (401 MHz, *o*-DFB, CD_2_Cl_2_, 21 °C) of Ph_3_SiOTeF_5_ (*: *o*-DFB, #: CD_2_Cl_2_).


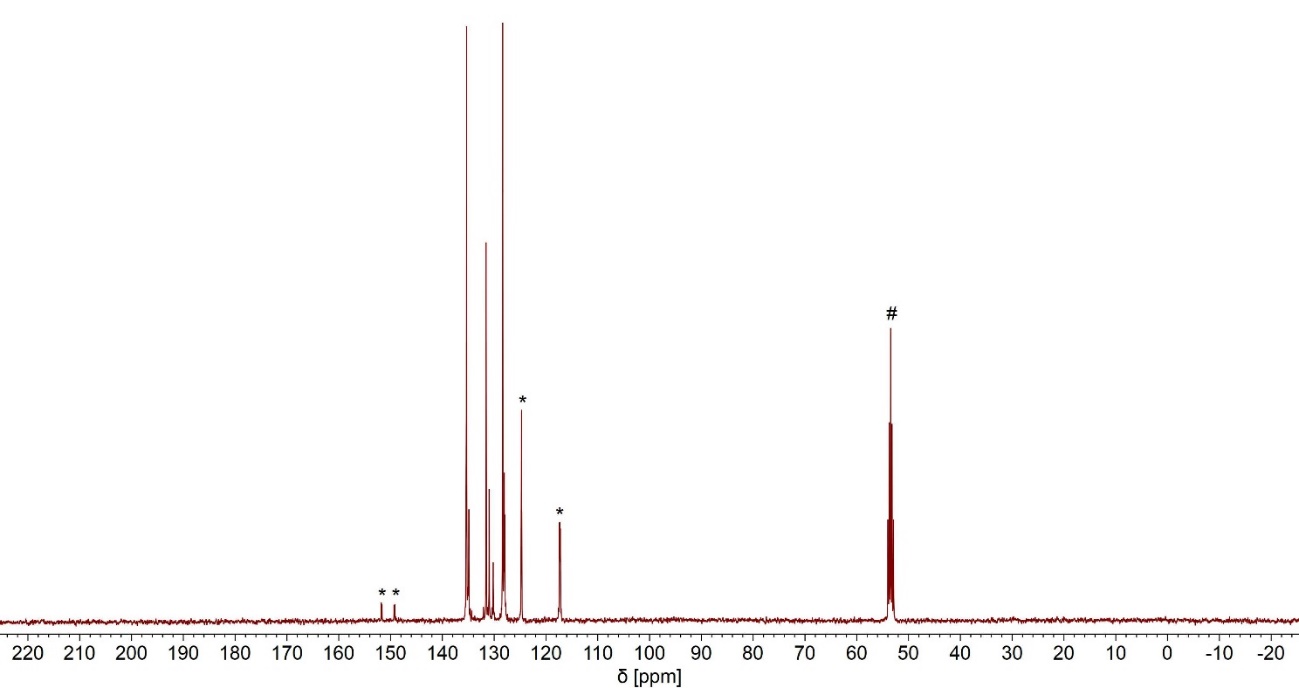


Figure S 56: ^13^C NMR spectrum (101 MHz, o-DFB, CD_2_Cl_2_, 21 °C) of Ph_3_SiOTeF_5_ (*: solvent, #: CD_2_Cl_2_).


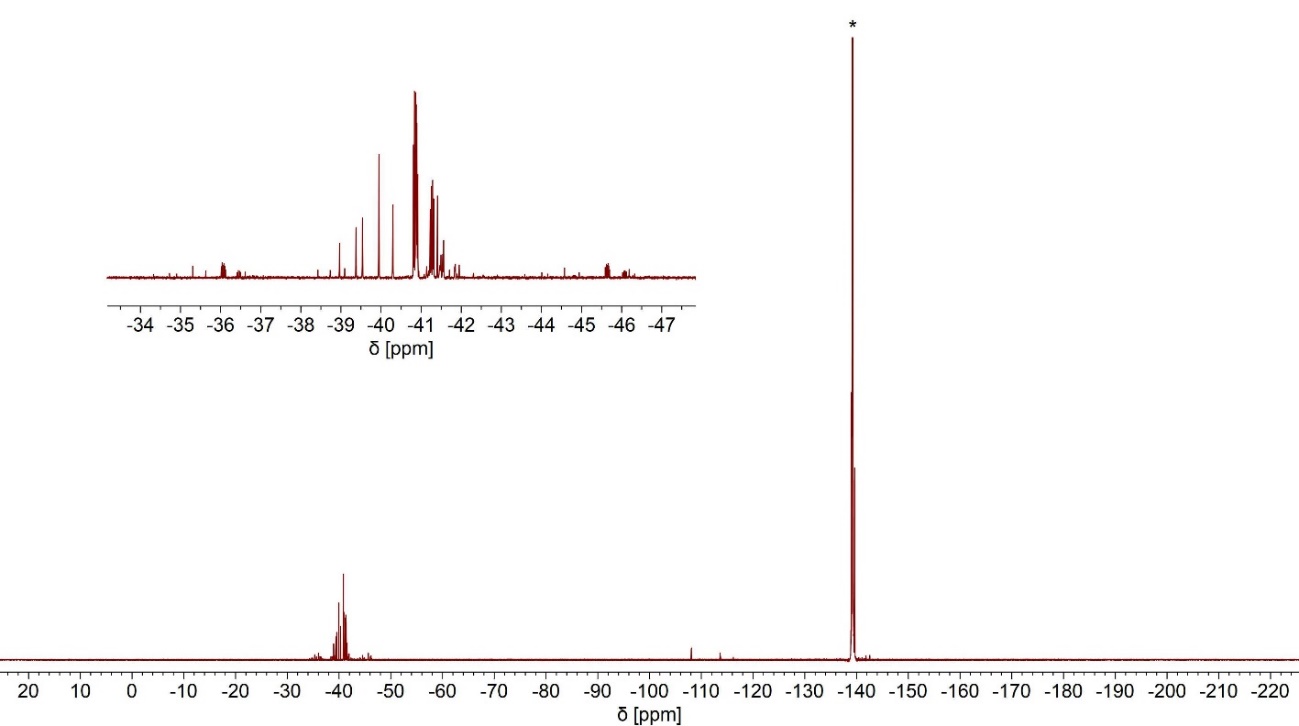


Figure S 57: ^19^F NMR spectrum (376 MHz, o-DFB, CD_2_Cl_2_, 20 °C) of Ph_3_SiOTeF_5_ (*: solvent).


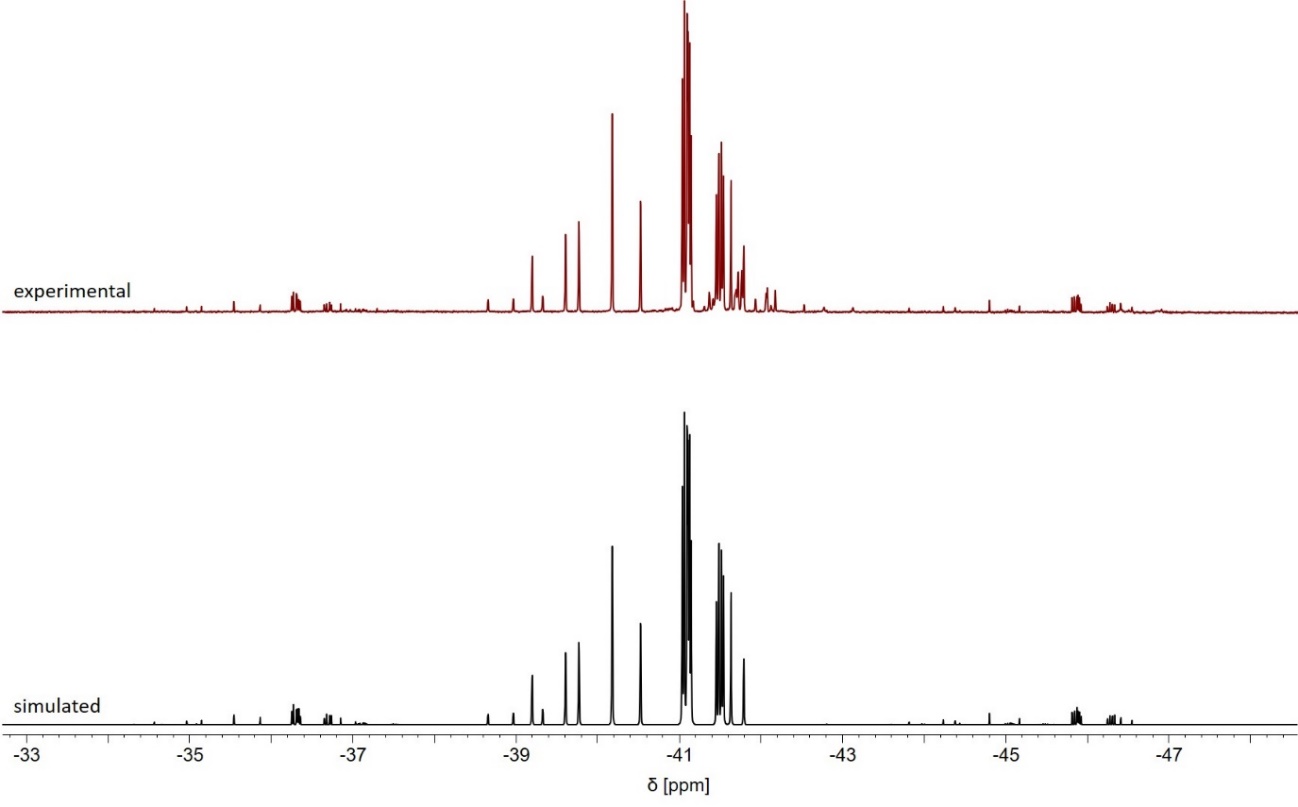


Figure S 58: Experimental (top) ^19^F NMR spectrum (376 MHz, o-DFB, CD_2_Cl_2_, 20 °C) and simulated (bottom) ^19^F NMR spectrum of Ph_3_SiOTeF_5_.


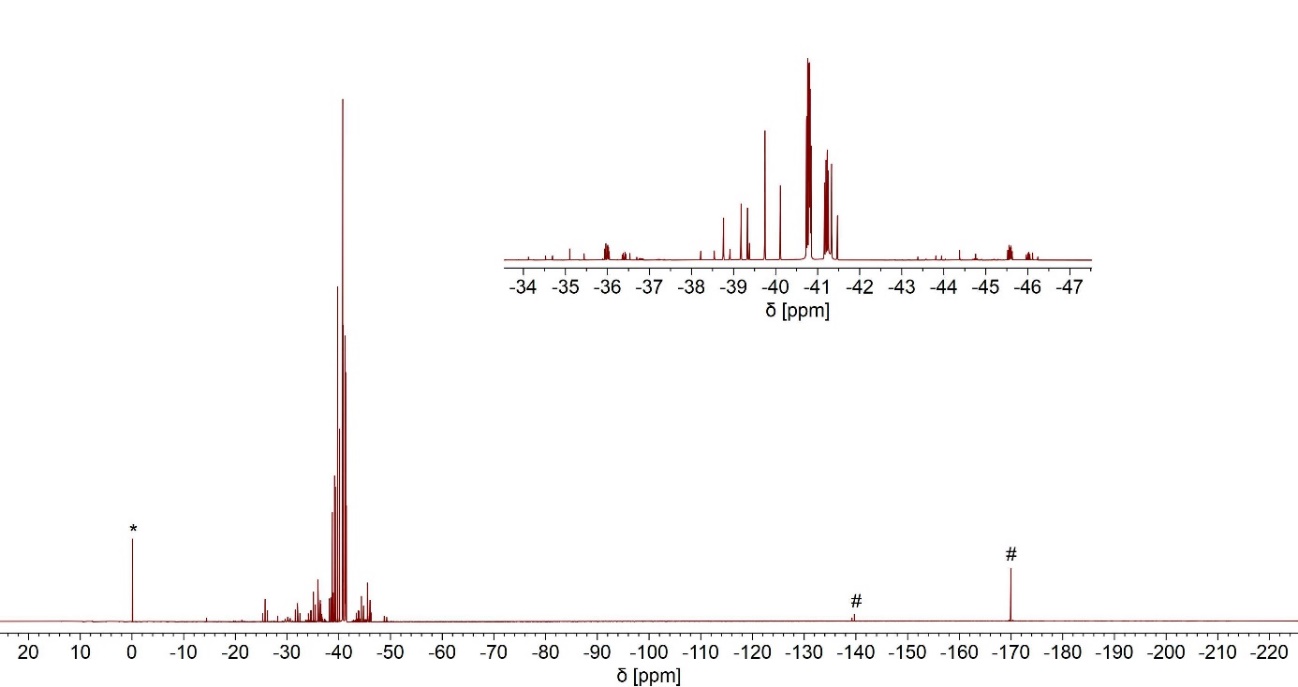


Figure S 59: ^19^F NMR spectrum (376 MHz, CH_2_Cl_2_, external lock acetone-d6, 18 °C) of Ph_3_SiOTeF_5_ (*: ext. CFCl_3_, #: unknown impurities).


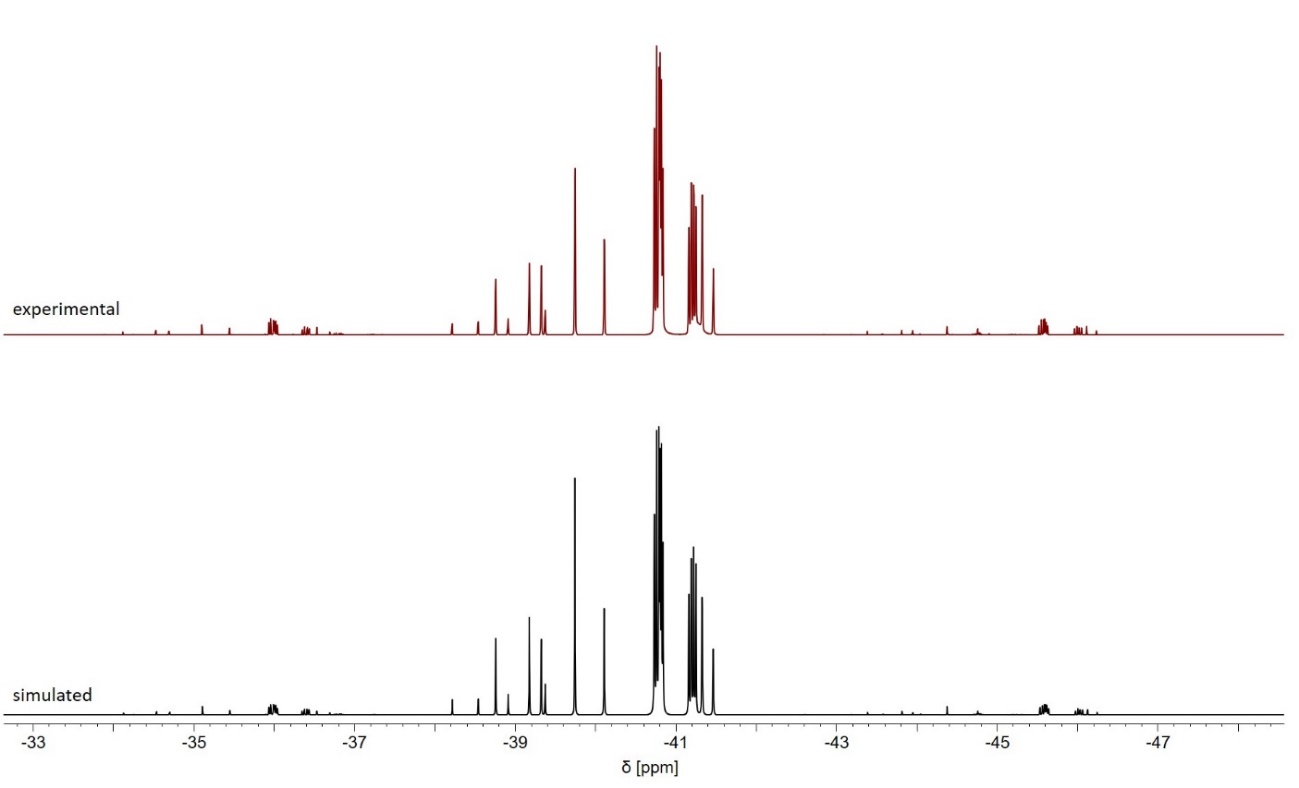


Figure S 60: Experimental (top) ^19^F NMR spectrum (376 MHz, CH_2_Cl_2_, external lock acetone-d6, 18 °C) and simulated (bottom) ^19^F NMR spectrum of Ph_3_SiOTeF_5_.


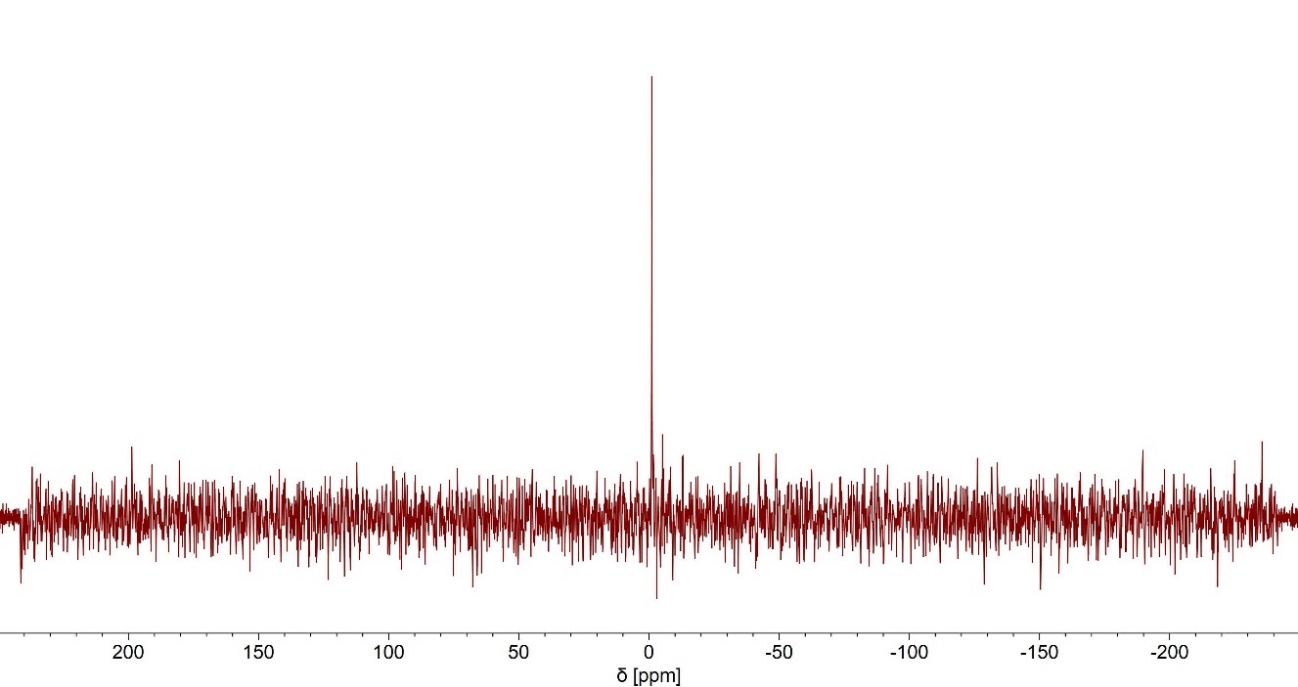


Figure S 61: ^29^Si DEPT NMR spectrum (80 MHz, o-DFB, CD_2_Cl_2_, 21 °C) of Ph_3_SiOTeF_5_.

## NMR spectra of Me_2_Si(OTeF_5_)_2_ (2a)


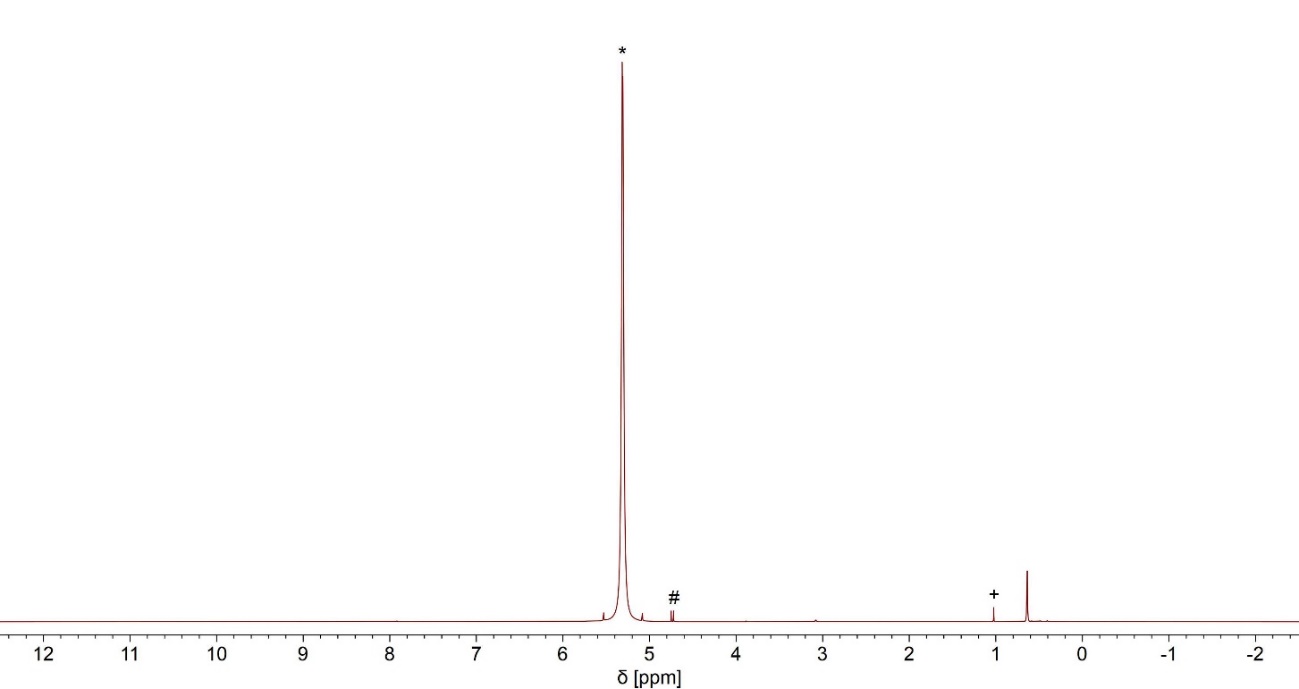


Figure S 62: ^1^H NMR spectrum (401 MHz, CH_2_Cl_2_, external lock acetone-d6, 18 °C) of Me_2_Si(OTeF_5_)_2_ (*: solvent, #: ext. (CH_3_O)_3_PO, +: ext. Si(CH_3_)_4_).


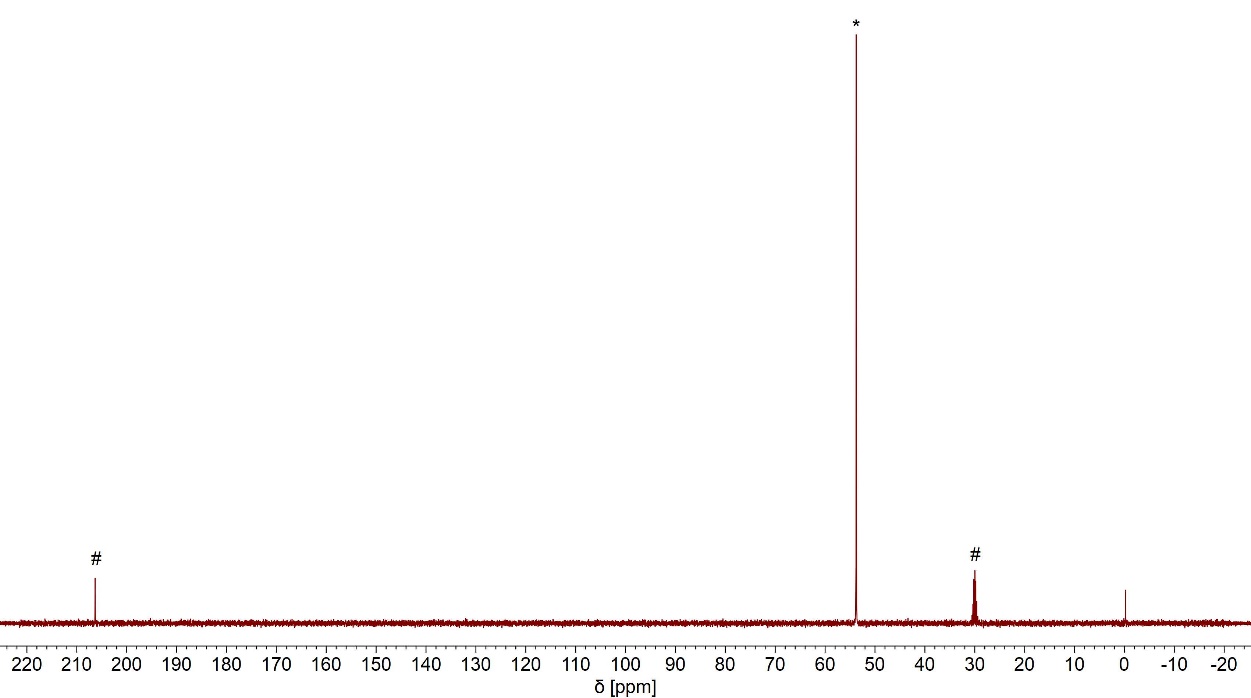


Figure S 63: ^13^C NMR spectrum (101 MHz, CH_2_Cl_2_, external lock acetone-d6, 18 °C) of Me_2_Si(OTeF_5_)_2_ (*: solvent, #: ext. (CD_3_)_2_CO).


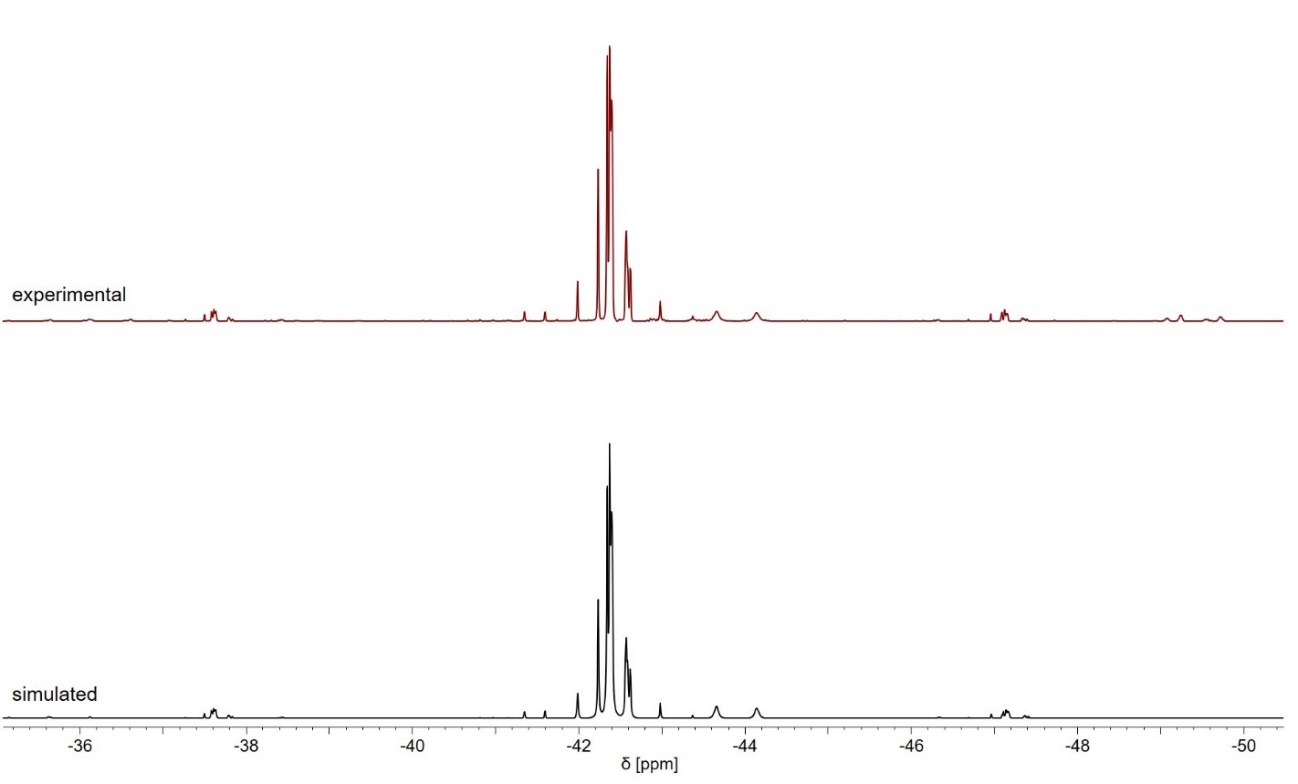


Figure S 64*:* Experimental (top) ^19^F NMR spectrum (377 MHz, CH_2_Cl_2_, external lock acetone-d6, 18 °C) and simulated (bottom) ^19^F NMR spectrum of Me_2_Si(OTeF_5_)_2_.


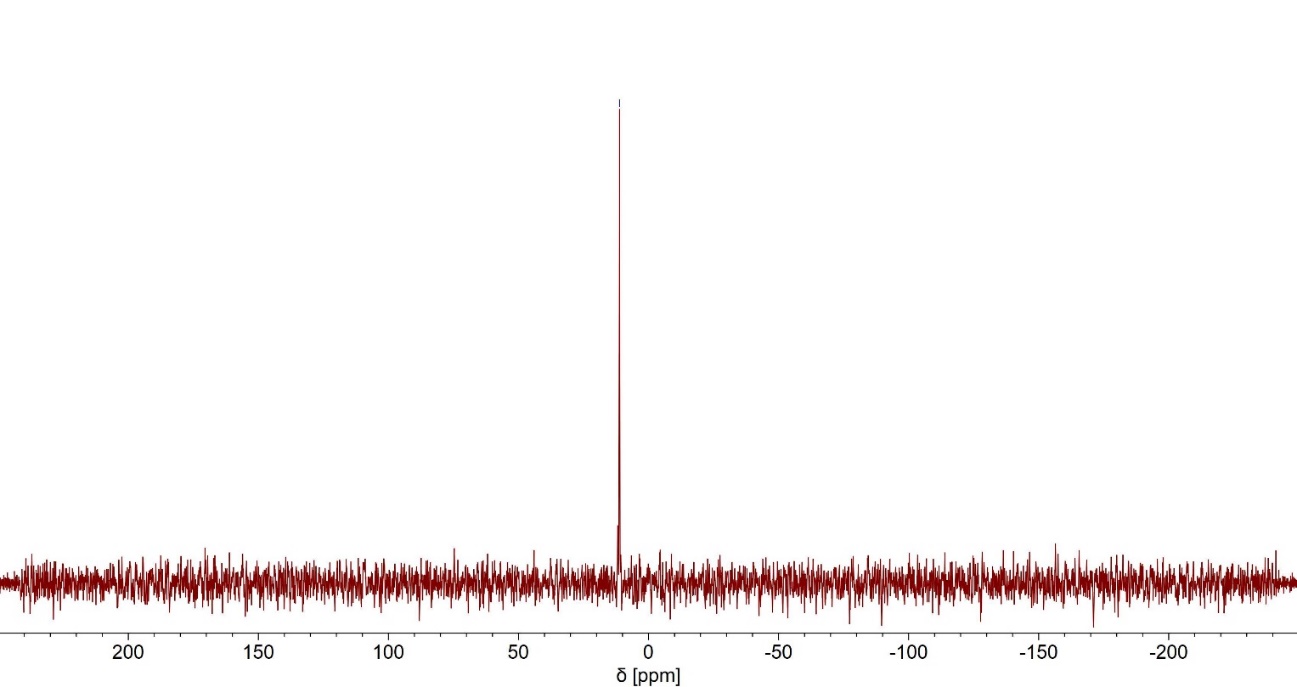


Figure S 65: ^29^Si DEPT NMR spectrum (80 MHz, CH_2_Cl_2_, external lock acetone-d6, 18 °C) of Me_2_Si(OTeF_5_)_2_.

## NMR spectra of Et_2_Si(OTeF_5_)_2_ (2b)


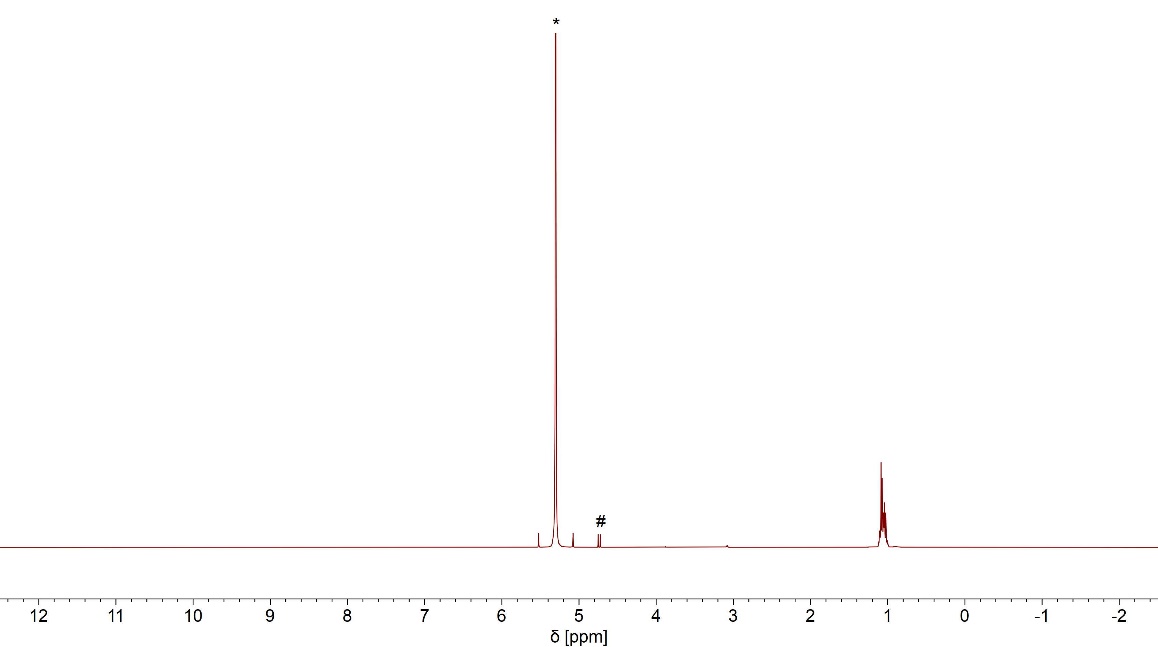


Figure S 66: ^1^H NMR spectrum (401 MHz, CH_2_Cl_2_, external lock acetone-d6, 18 °C) of Et_2_Si(OTeF_5_)_2_ (*: solvent, #: ext. (CH_3_O)_3_PO).


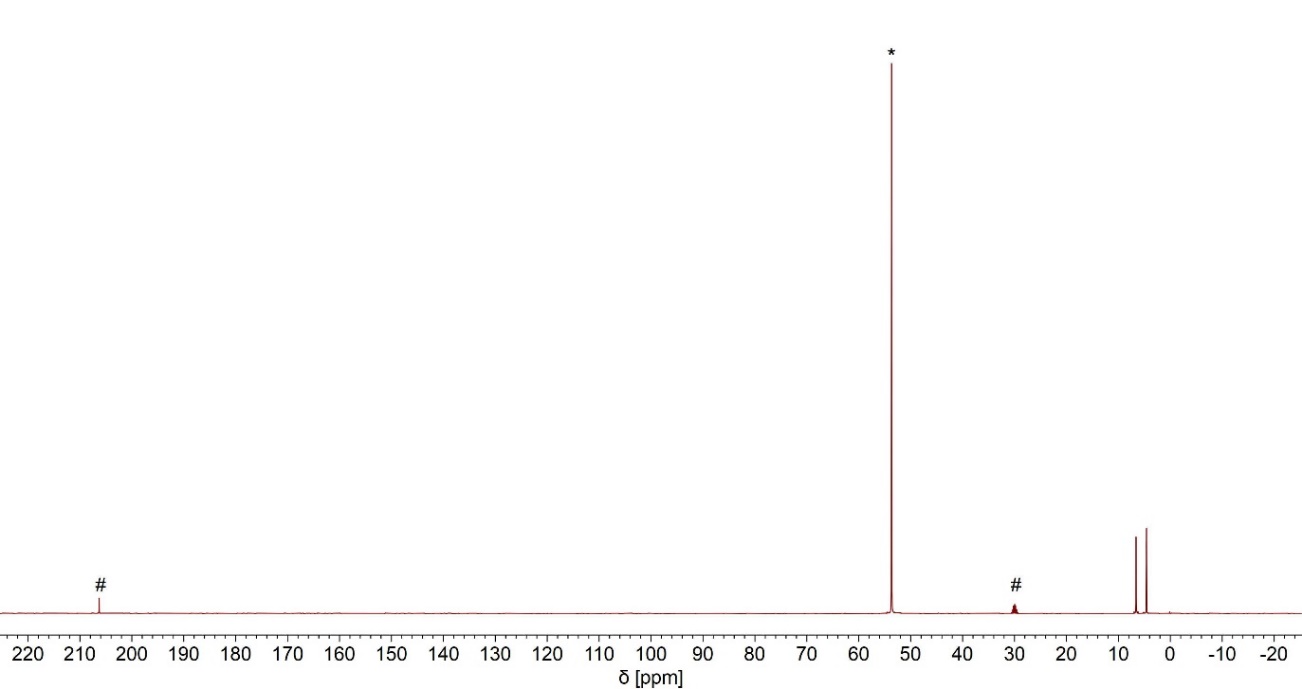


Figure S 67: ^13^C NMR spectrum (101 MHz, CH_2_Cl_2_, external lock acetone-d6, 19 °C) of Et_2_Si(OTeF_5_)_2_ (*: solvent, #: ext. (CD_3_)_2_CO).


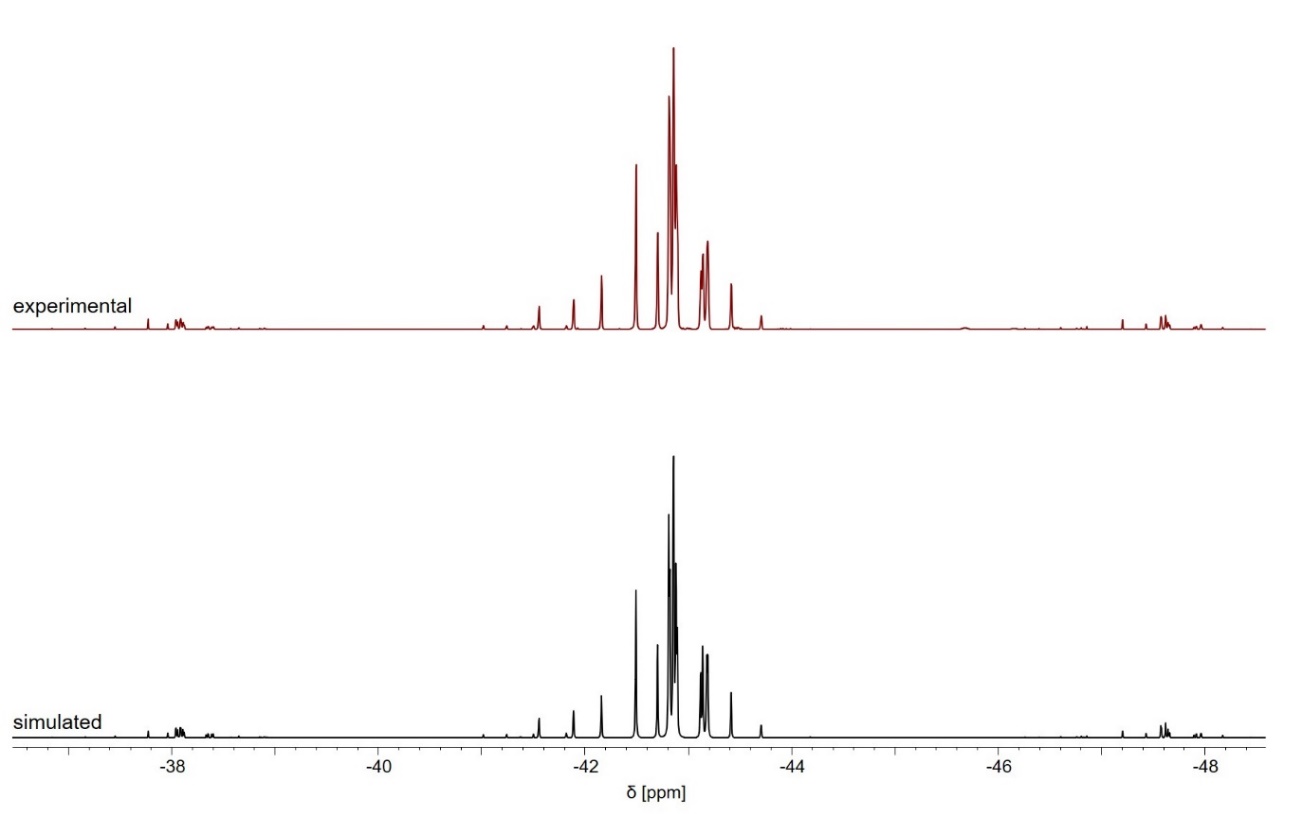


Figure S 68: Experimental (top) ^19^F NMR spectrum (377 MHz, CH_2_Cl_2_, external lock acetone-d6, 18 °C) and simulated (bottom) ^19^F NMR spectrum of Et_2_Si(OTeF_5_)_2_.


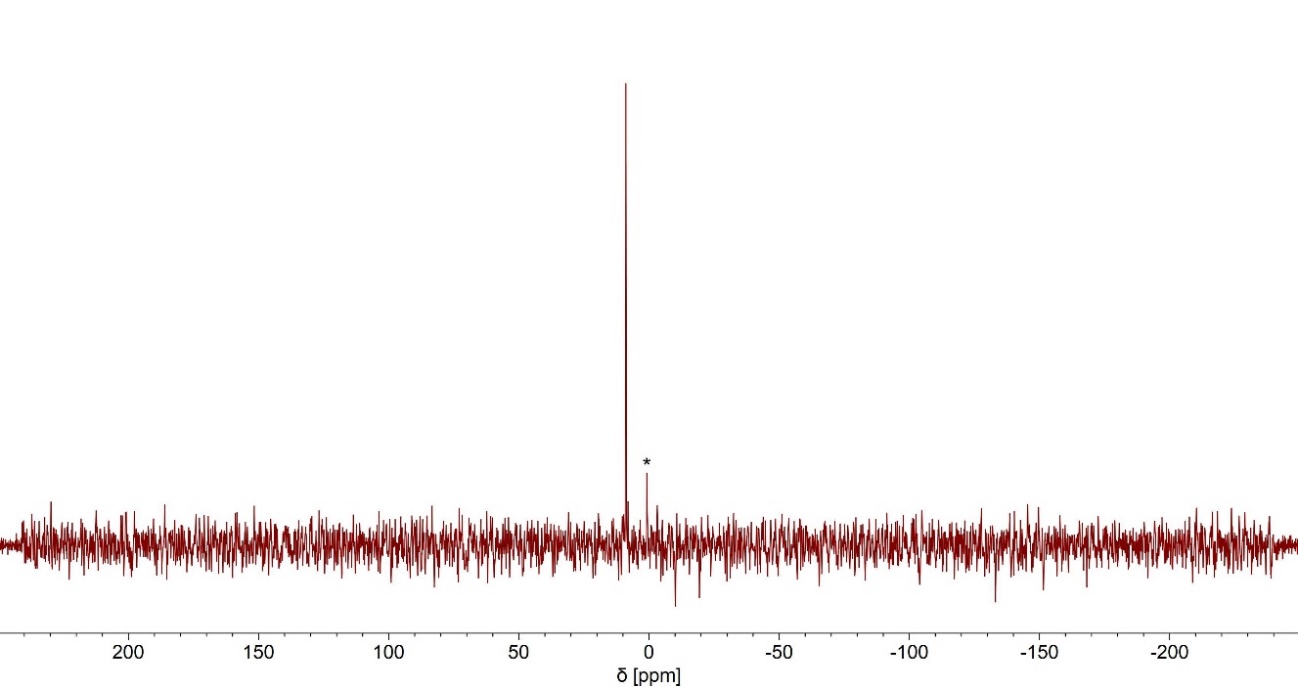


Figure S 69: ^29^Si DEPT NMR spectrum (80 MHz, CH_2_Cl_2_, external lock acetone-d6, 17 °C) of Et_2_Si(OTeF_5_)_2_ (*: ext. Si(CH_3_)_4_).


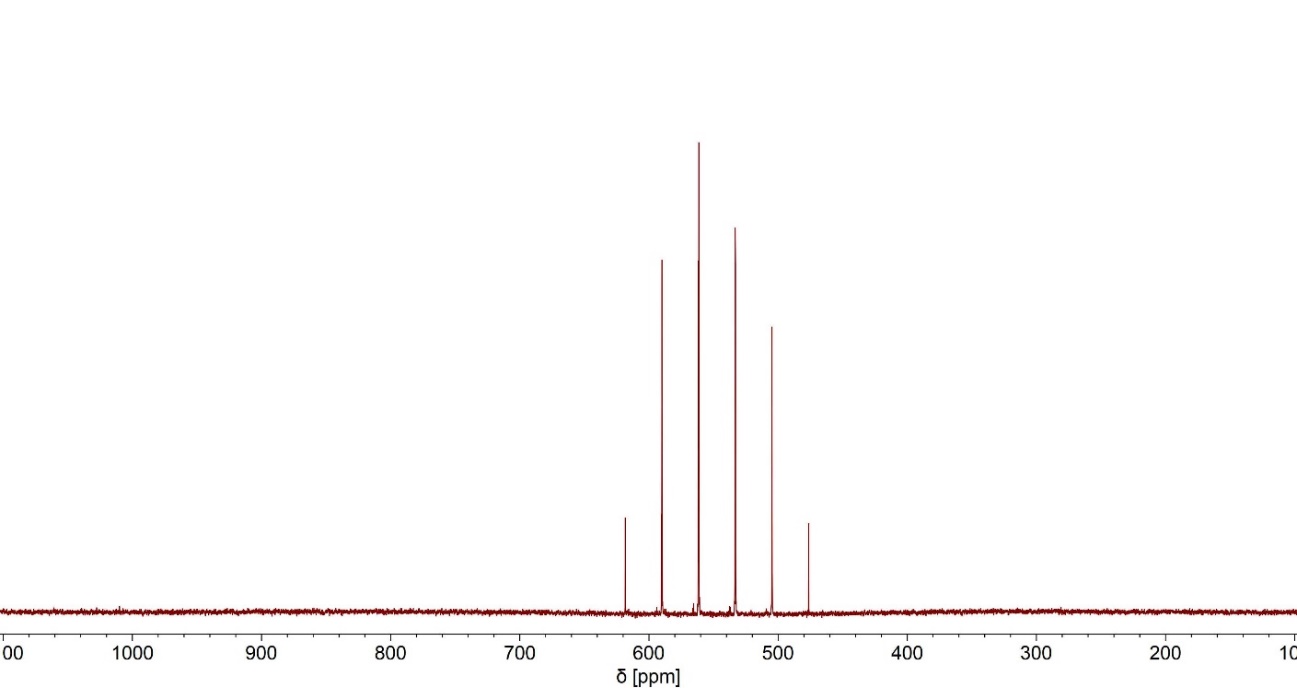


Figure S 70: ^125^Te NMR spectrum (126 MHz, neat, external lock acetone-d6, 20 °C) of Et_2_Si(OTeF_5_)_2_.


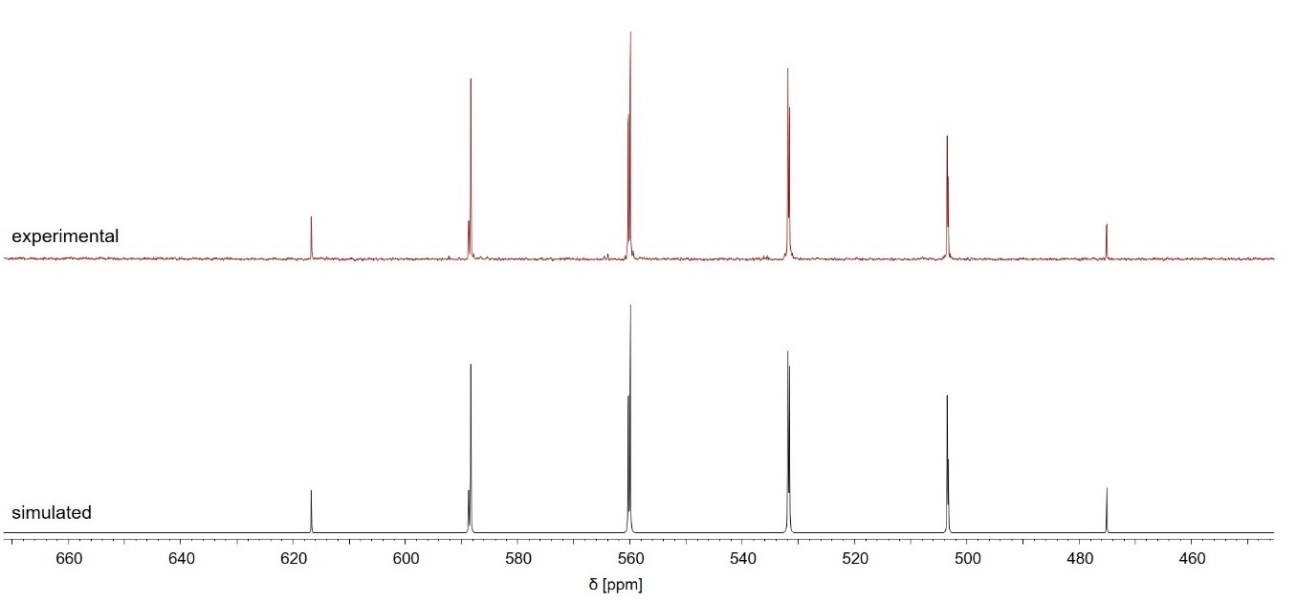


Figure S 71: Experimental (top) ^125^Te NMR spectrum (126 MHz, neat, external lock acetone-d6, 20 °C) and simulated (bottom) ^125^Te NMR spectrum of Et_2_Si(OTeF_5_)_2_.

## NMR spectra of *^i^*Pr_2_Si(OTeF_5_)_2_ (2c)


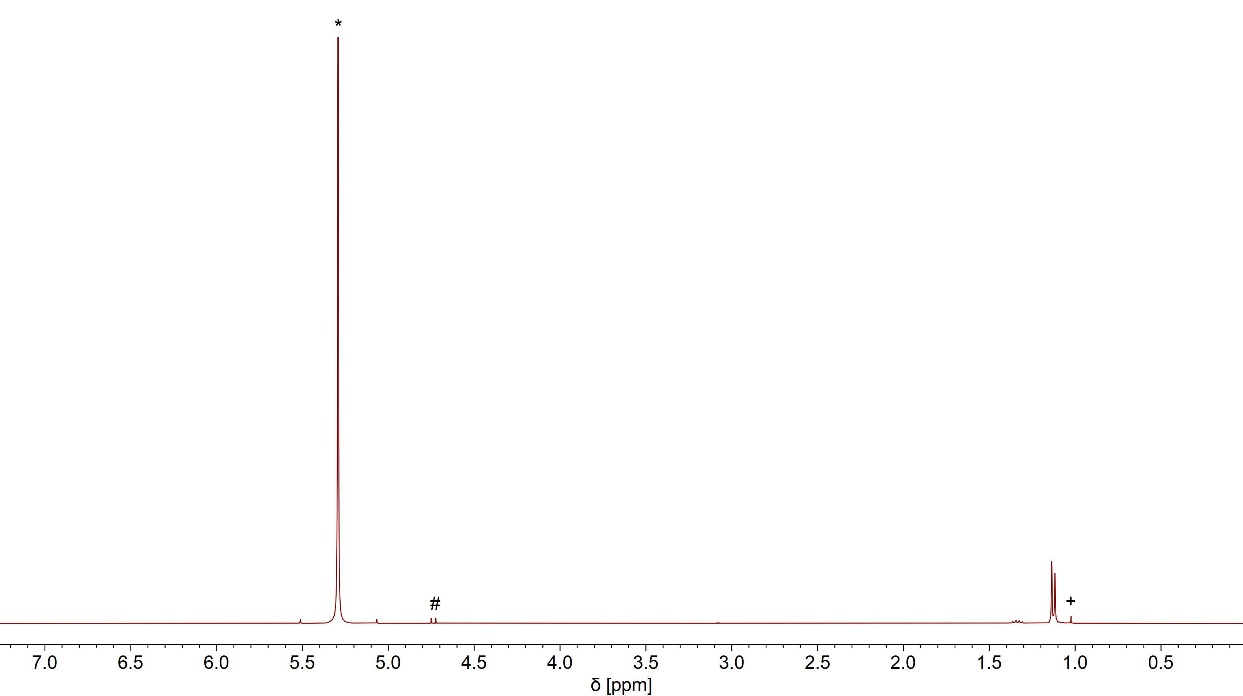


Figure S 72: ^1^H NMR spectrum (401 MHz, CH_2_Cl_2_, external lock acetone-d6, 18 °C) of ^i^Pr_2_Si(OTeF_5_)_2_ (*: solvent, #: ext. (CH_3_O)_3_PO, +: ext. Si(CH_3_)_4_).


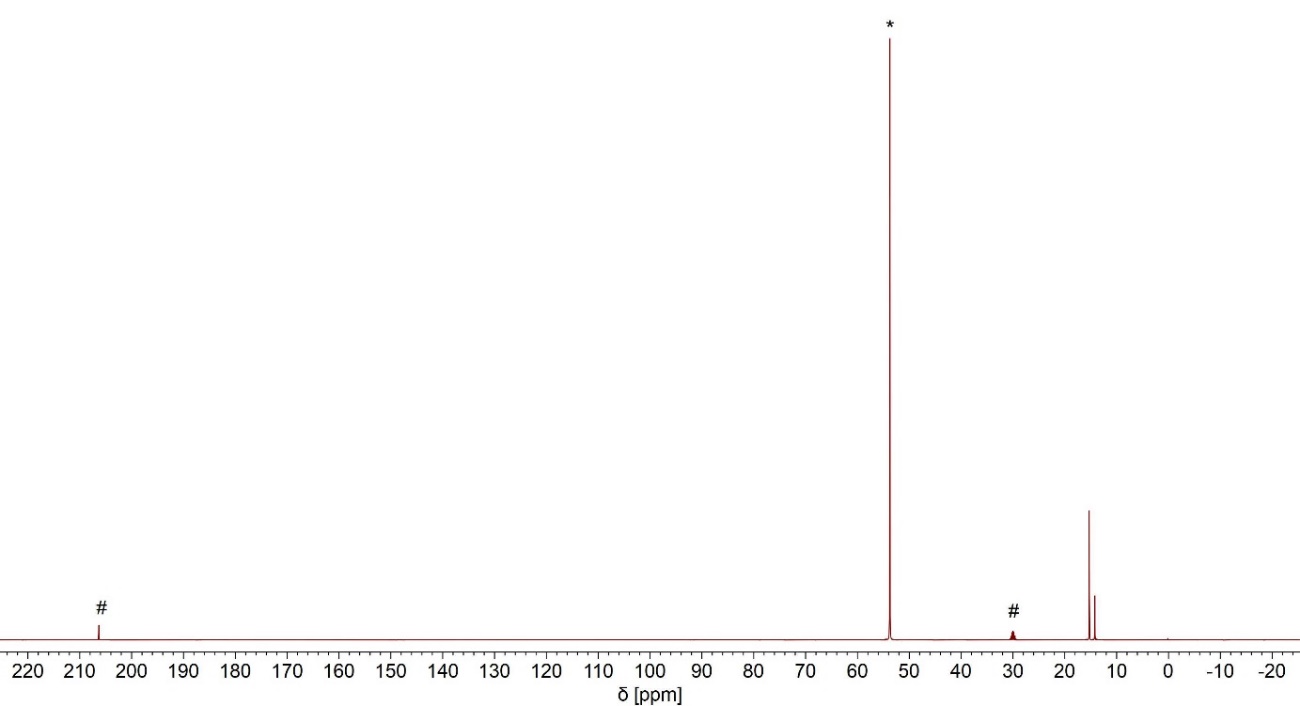


Figure S 73: ^13^C NMR spectrum (101 MHz, CH_2_Cl_2_, external lock acetone-d6, 16 °C) of ^i^Pr_2_Si(OTeF_5_)_2_ (*: solvent, #: ext. (CD_3_)_2_CO).


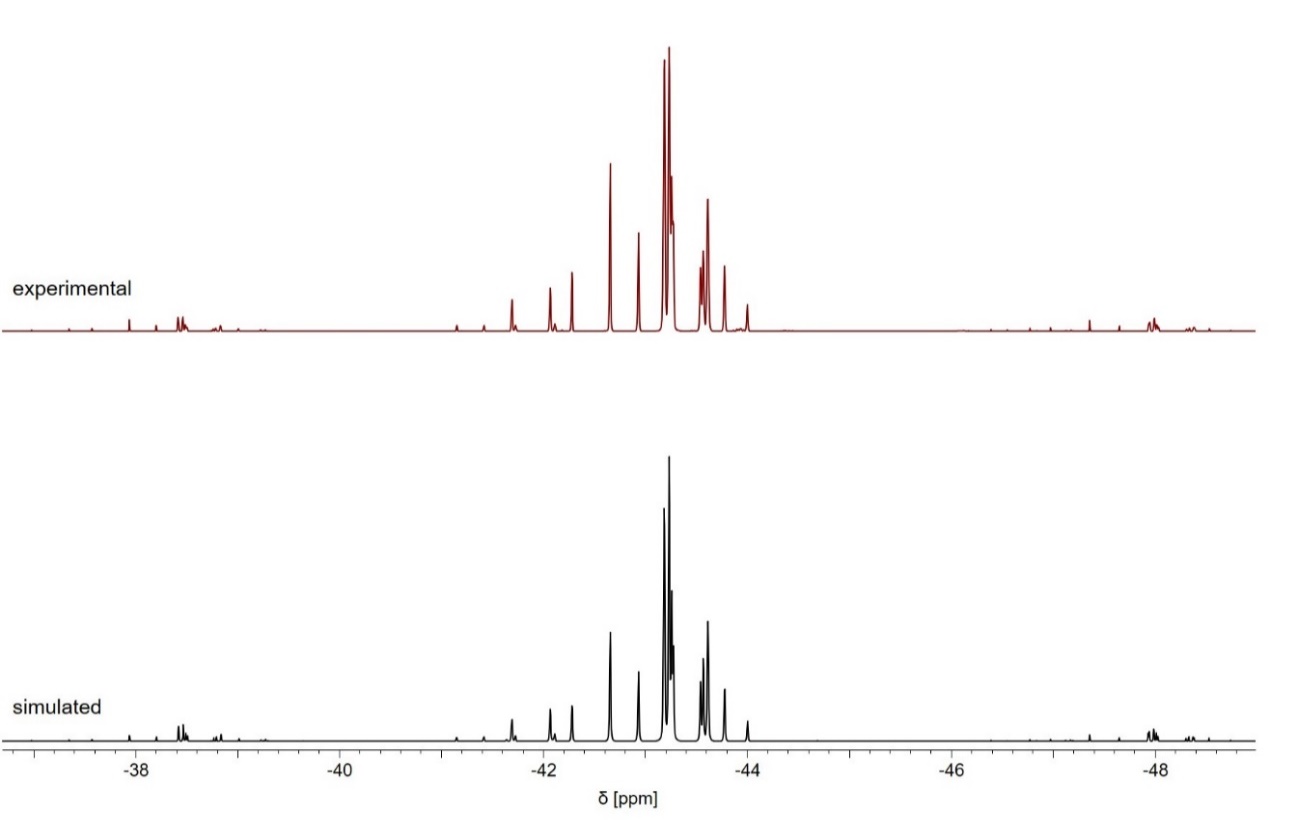


Figure S 74: Experimental (top) ^19^F NMR spectrum (377 MHz, CH_2_Cl_2_, external lock acetone-d6, 16 °C) and simulated (bottom) ^19^F NMR spectrum of ^i^Pr_2_Si(OTeF_5_)_2_.


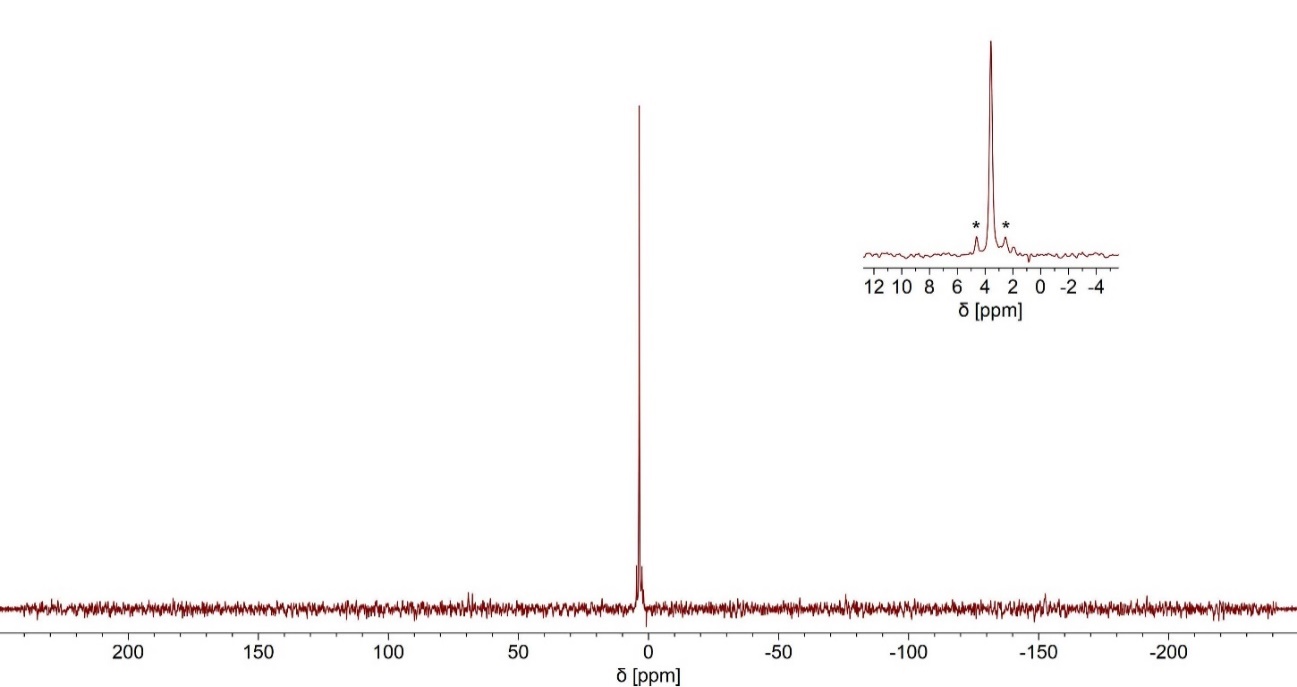


Figure S 75: ^29^Si DEPT NMR spectrum (80 MHz, CH_2_Cl_2_, external lock acetone-d6, 17 °C) of ^i^Pr_2_Si(OTeF_5_)_2_. ^125^Te satellites are marked with an asterix (*).


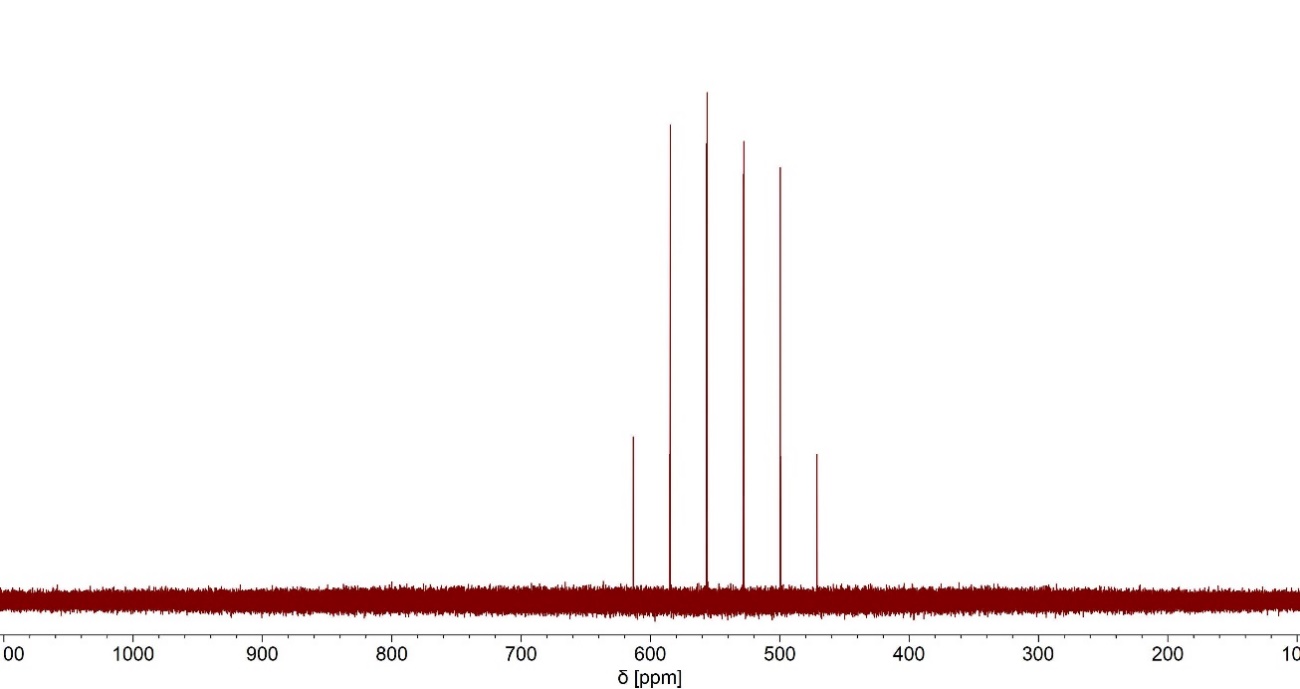


Figure S 76: ^125^Te NMR spectrum (126 MHz, CH_2_Cl_2_, external lock acetone-d6, 22 °C) of ^i^Pr_2_Si(OTeF_5_)_2_.


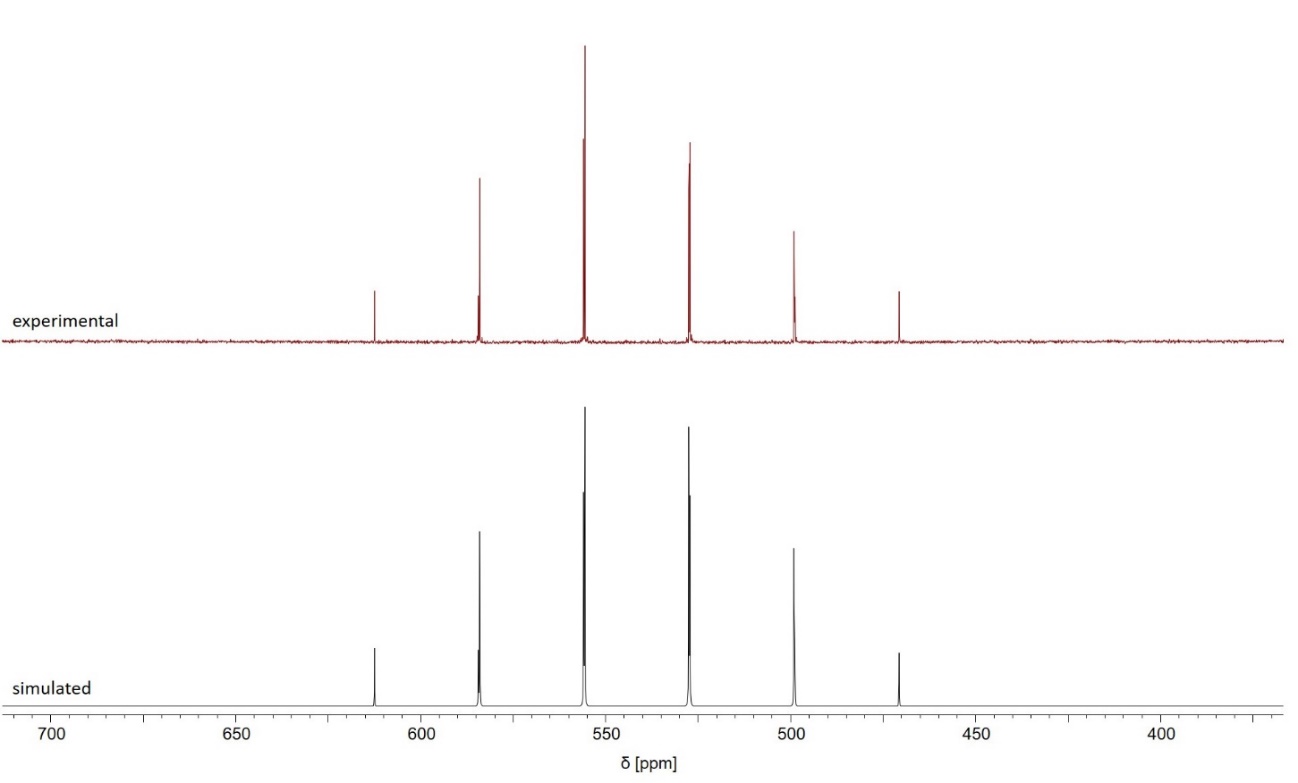


Figure S 77: Experimental (top) ^125^Te NMR spectrum (126 MHz, CH_2_Cl_2_, external lock acetone-d6, 22 °C) and simulated (bottom) ^125^Te NMR spectrum of ^i^Pr_2_Si(OTeF_5_)_2_.

## NMR spectra of Ph_2_Si(OTeF_5_)_2_ (2d)


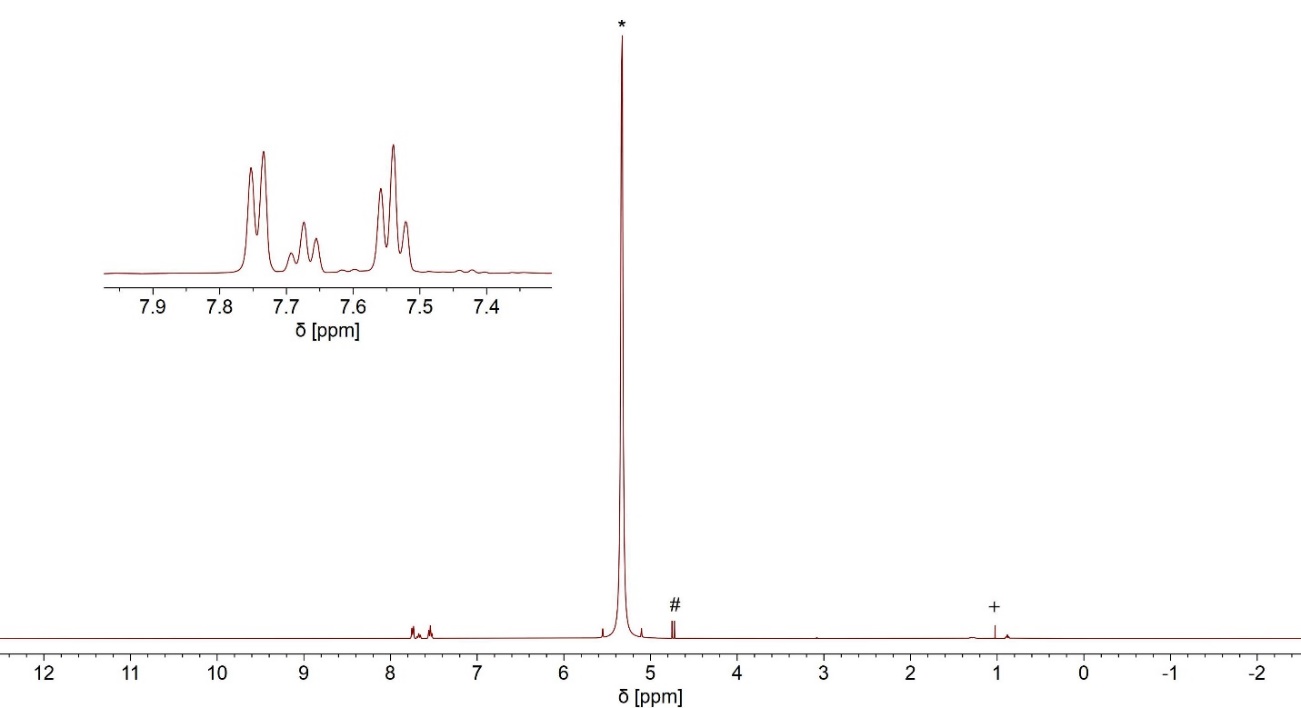


Figure S 78: ^1^H NMR spectrum (399 MHz, CH_2_Cl_2_, external lock acetone-d6, 18 °C) of Ph_2_Si(OTeF_5_)_2_ (*: solvent, #: ext. (CH_3_O)_3_PO, +: ext. Si(CH_3_)_4_).


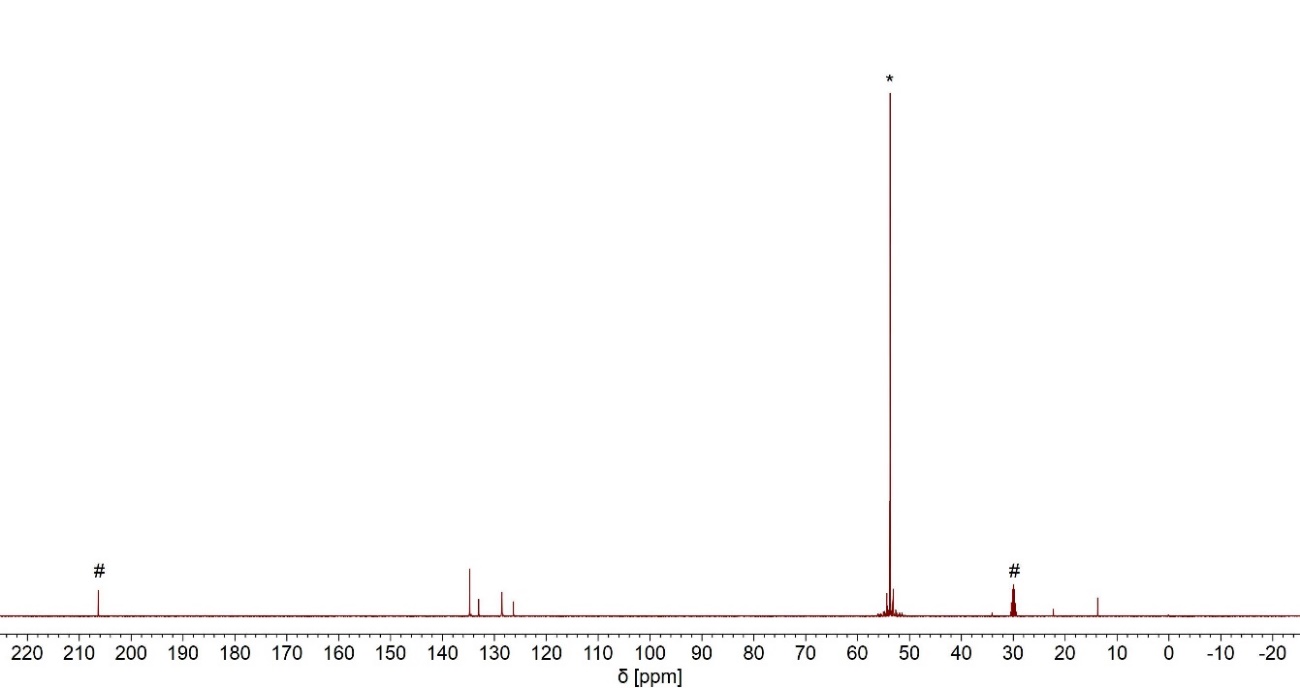


Figure S 79: ^13^C NMR spectrum (100 MHz, CH_2_Cl_2_, external lock acetone-d6, 20 °C) of Ph_2_Si(OTeF_5_)_2_ (*: solvent, , #: ext. (CD_3_)_2_CO).


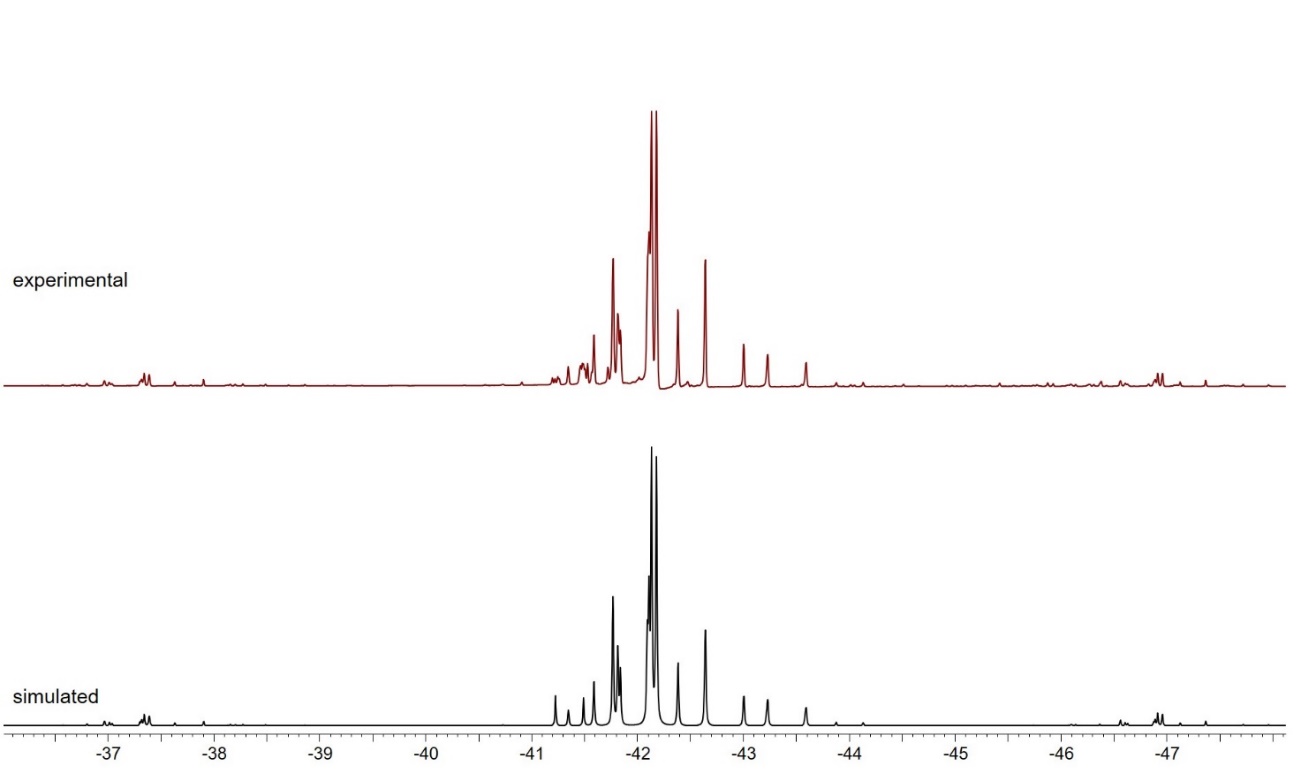


Figure S 80: Experimental (top) ^19^F NMR spectrum (377 MHz, CH_2_Cl_2_, external lock acetone-d6, 17 °C) and simulated (bottom) ^19^F NMR spectrum of Ph_2_Si(OTeF_5_)_2_.


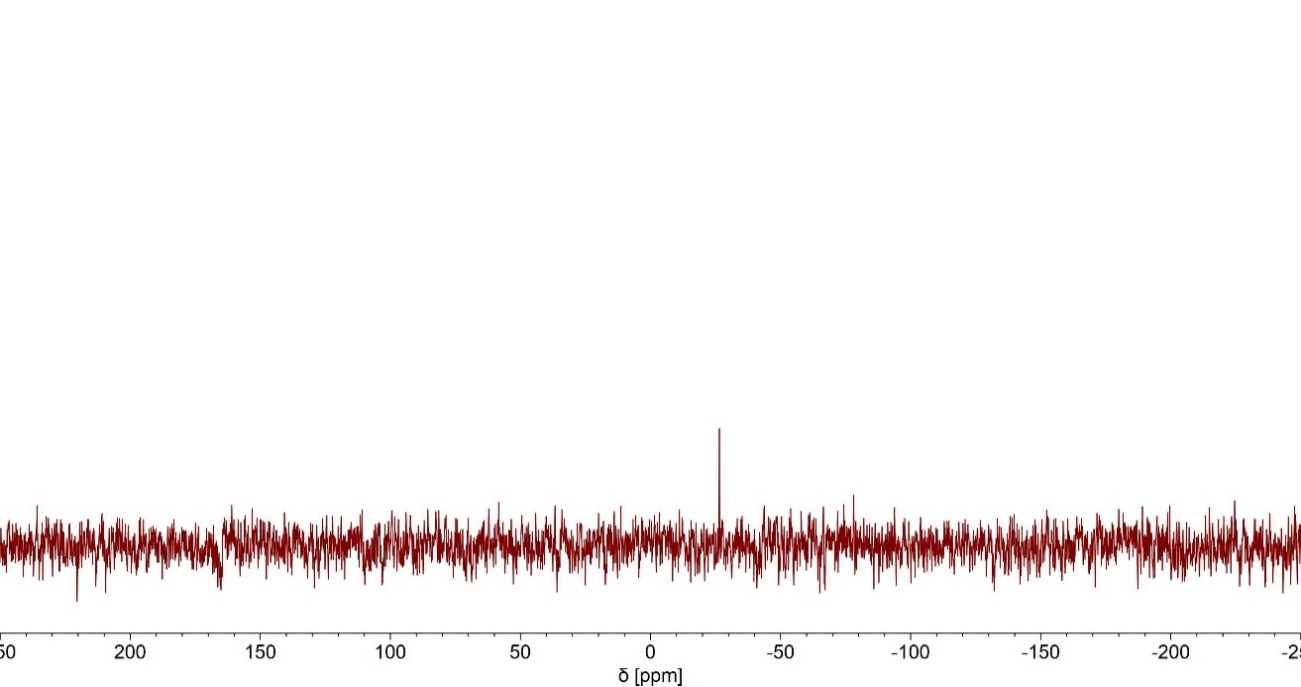


Figure S 81: ^29^Si DEPT NMR spectrum (79 MHz, CH_2_Cl_2_, external lock acetone-d6, 20 °C) of Ph_2_Si(OTeF_5_)_2_.


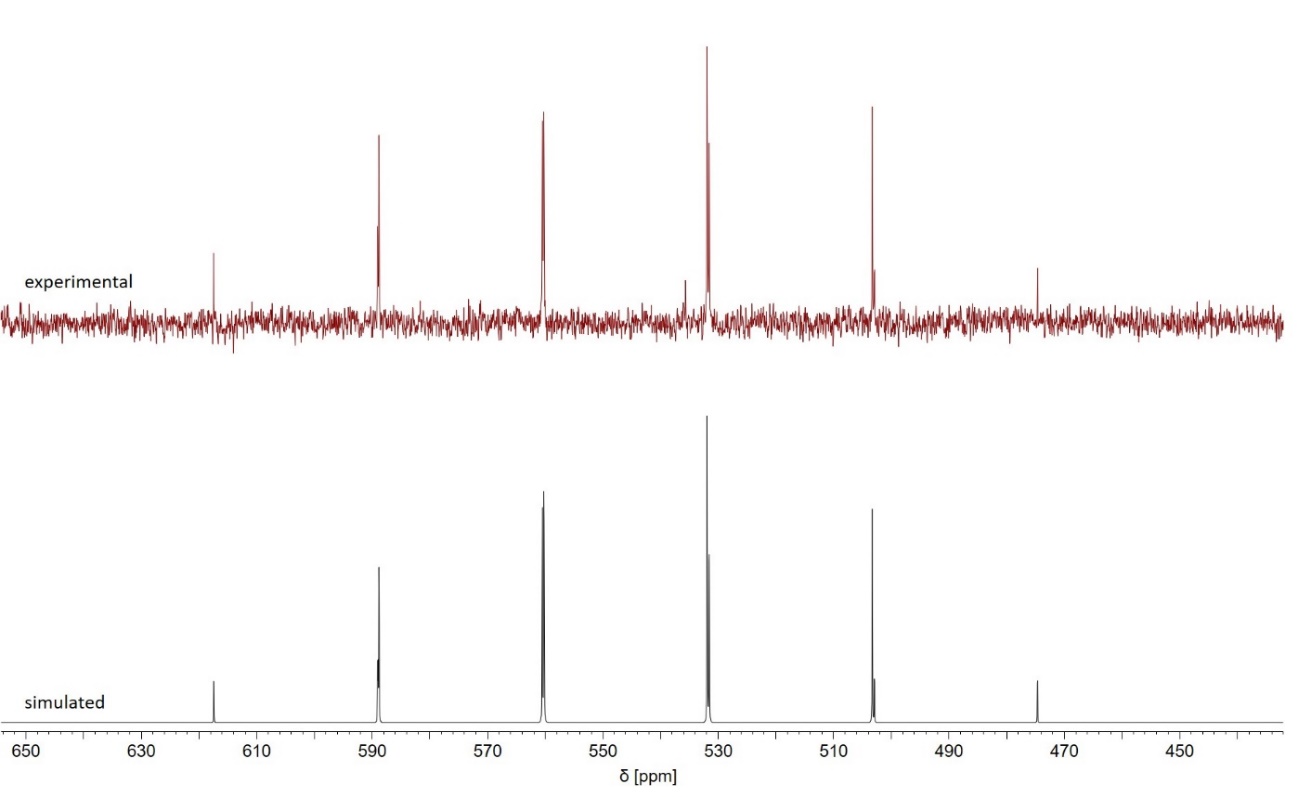


Figure S 82: Experimental (top) ^125^Te NMR spectrum (126 MHz, CH_2_Cl_2_, external lock acetone-d6, 18 °C) and simulated (bottom) ^125^Te NMR spectrum of Ph_2_Si(OTeF_5_)_2_.

## NMR spectra of MeSi(OTeF_5_)_3_ (3a)


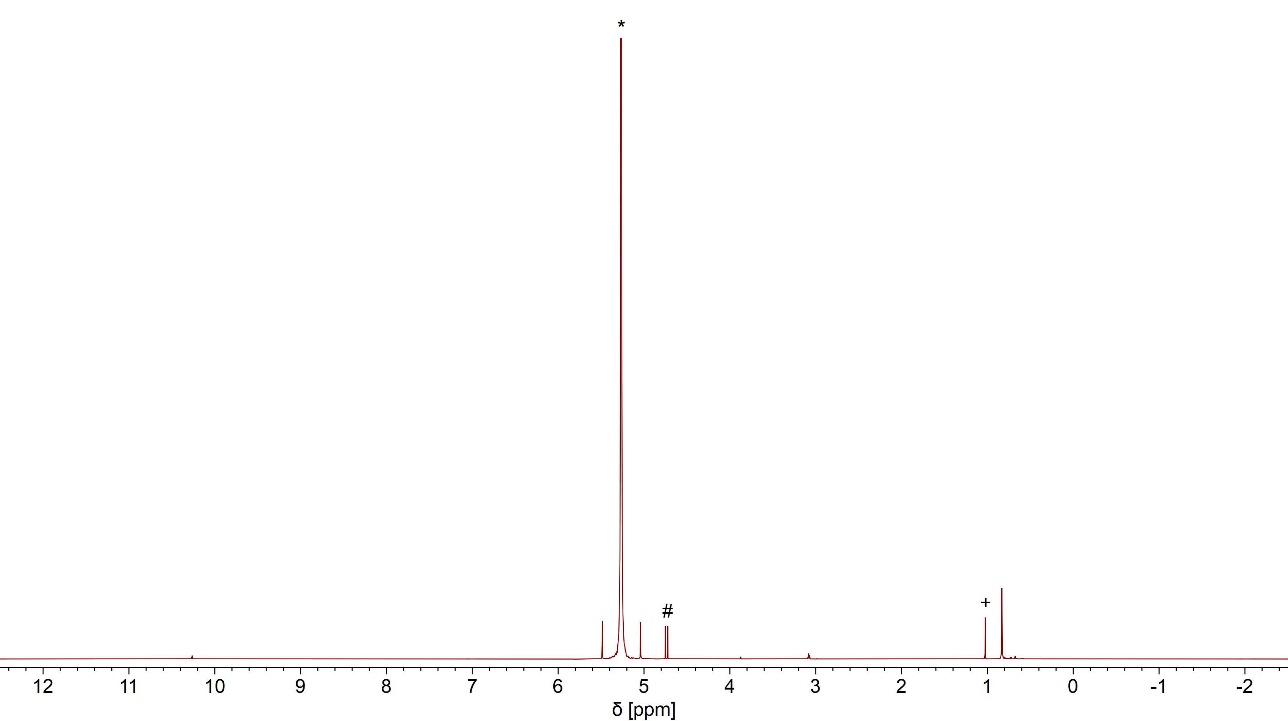


Figure S 83: ^1^H NMR spectrum (401 MHz, CH_2_Cl_2_, external lock acetone-d6, 18 °C) of MeSi(OTeF_5_)_3_ (*: solvent, #: ext. (CH_3_O)_3_PO, +: ext. Si(CH_3_)_4_).


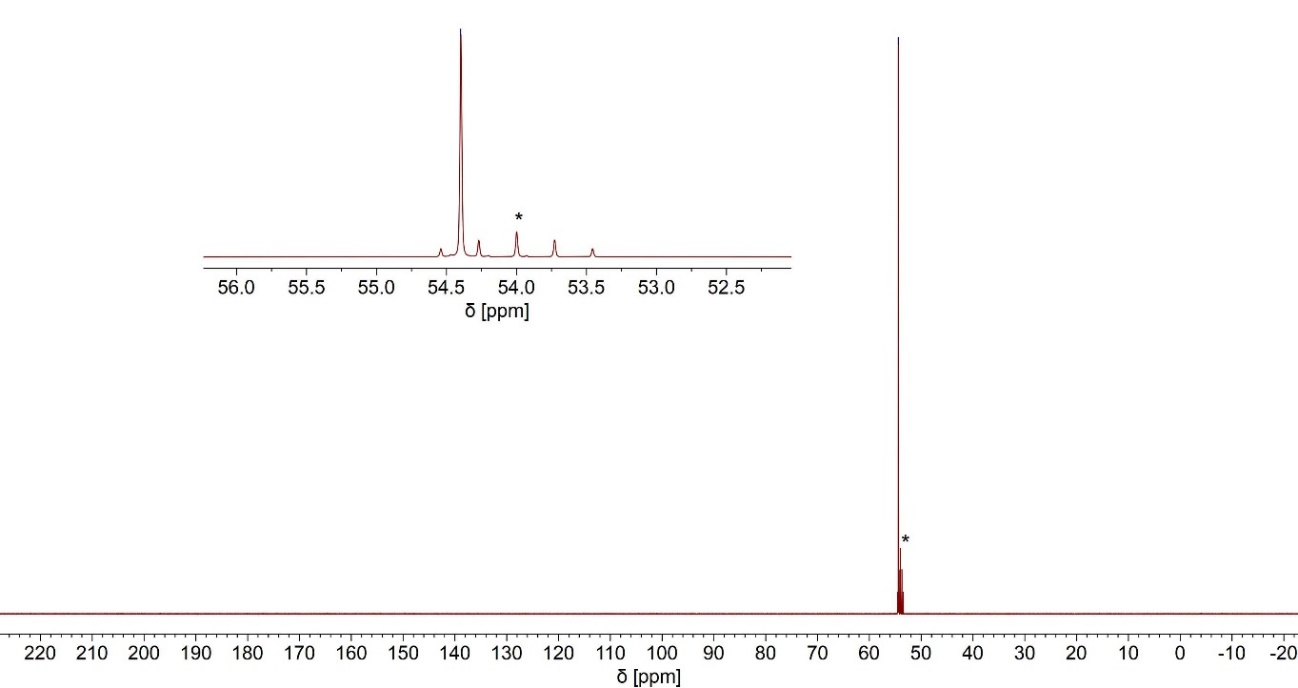


Figure S 84: ^13^C NMR spectrum (101 MHz, CH_2_Cl_2_, external lock acetone-d6, 19 °C) of MeSi(OTeF_5_)_3_ (*: solvent).


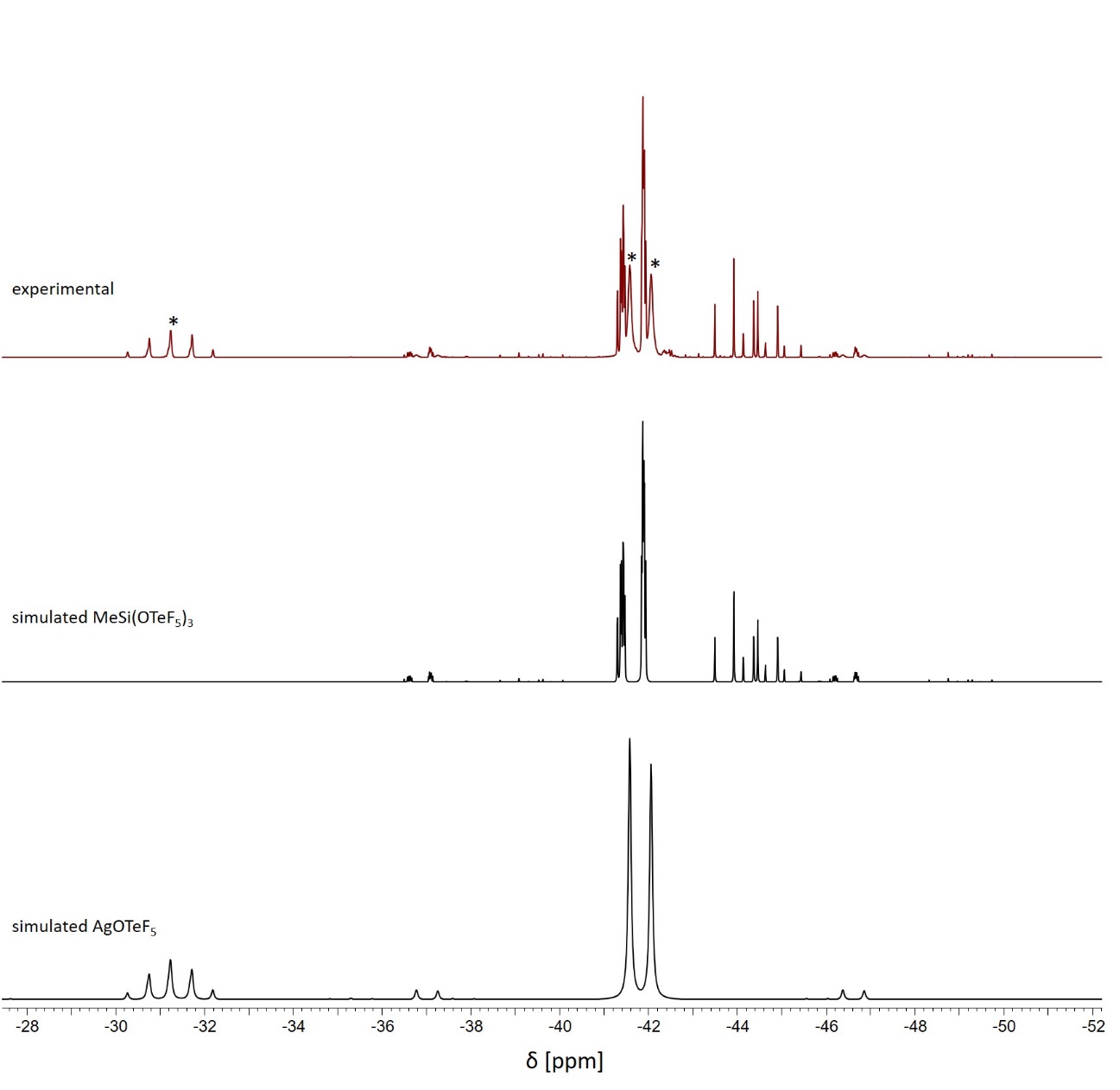


Figure S 85: Experimental (top) ^19^F NMR spectrum (377 MHz, CH_2_Cl_2_, external lock acetone-d6, 19 °C) and simulated (middle) ^19^F NMR spectrum of MeSi(OTeF_5_)_3_ as well as simulated (bottom) ^19^F NMR spectrum of AgOTeF_5_ (*: AgOTeF_5_).


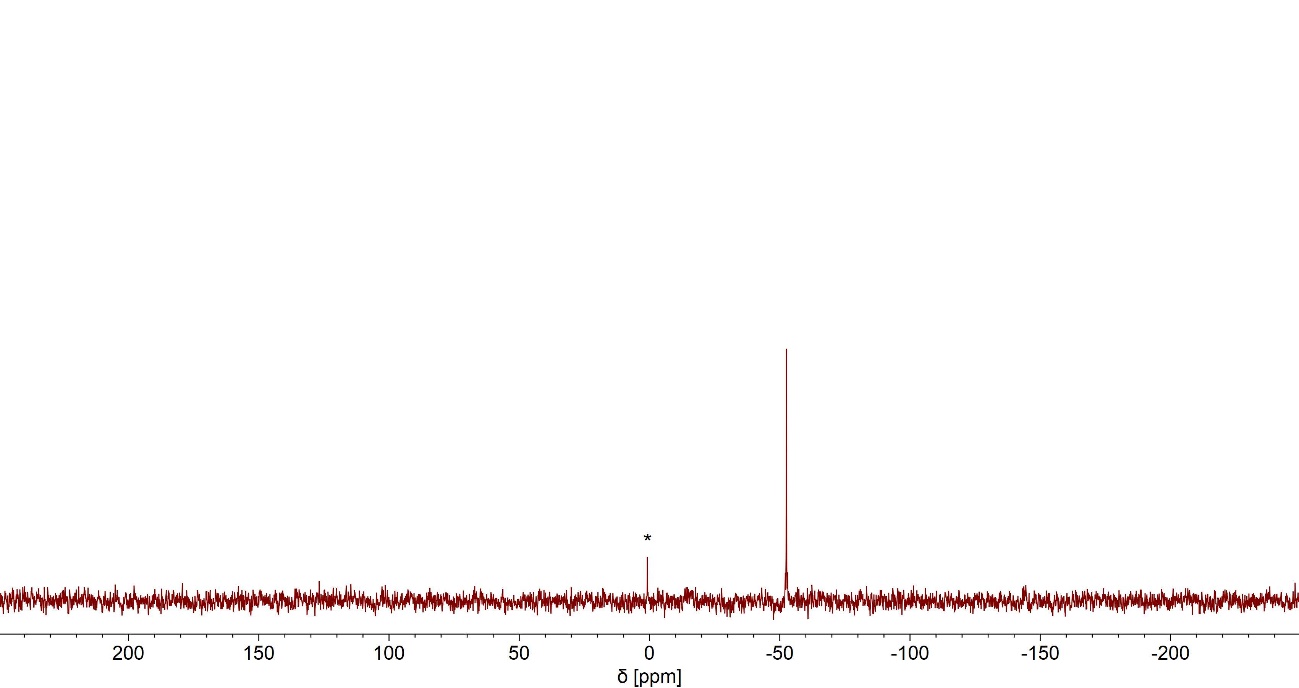


Figure S 86: ^29^Si DEPT NMR spectrum (80 MHz, CH_2_Cl_2_, external lock acetone-d6, 20 °C) of MeSi(OTeF_5_)_3_ (*: ext. Si(CH_3_)_4_).


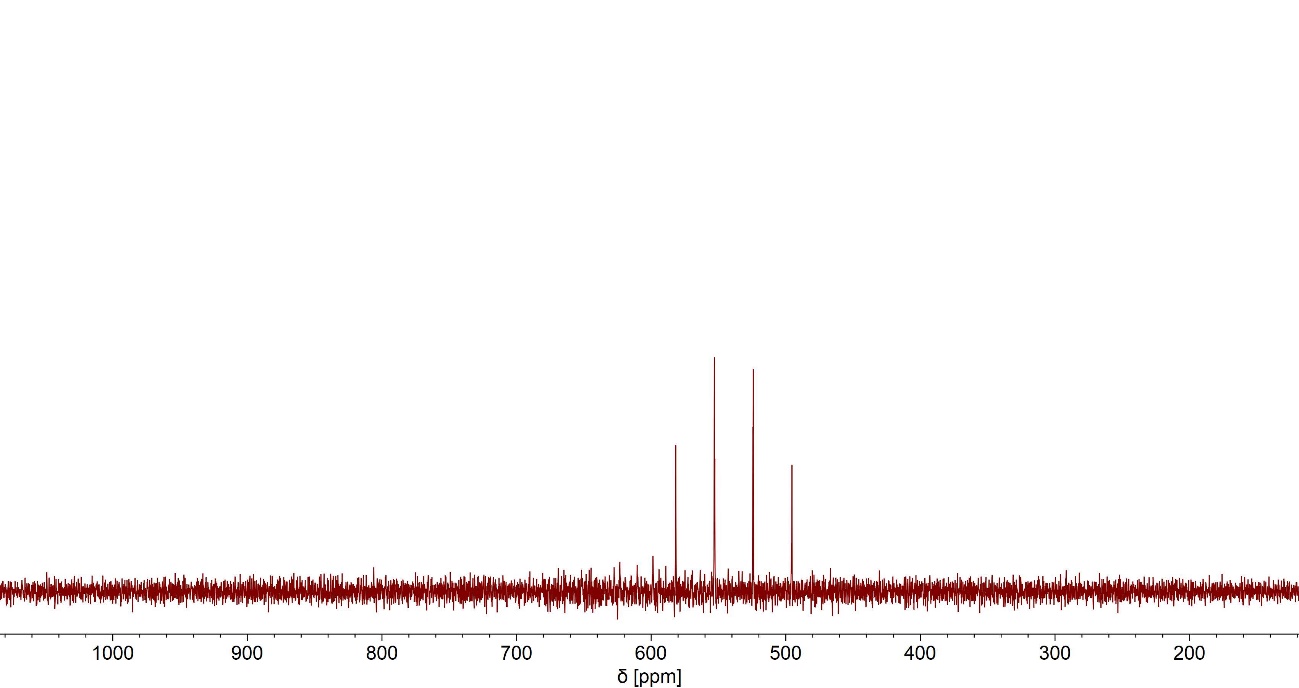


Figure S 87: ^125^Te NMR spectrum (126 MHz, CH_2_Cl_2_, external lock acetone-d6, 19 °C) of MeSi(OTeF_5_)_3_.

## NMR spectra of EtSi(OTeF_5_)_3_ (3b)


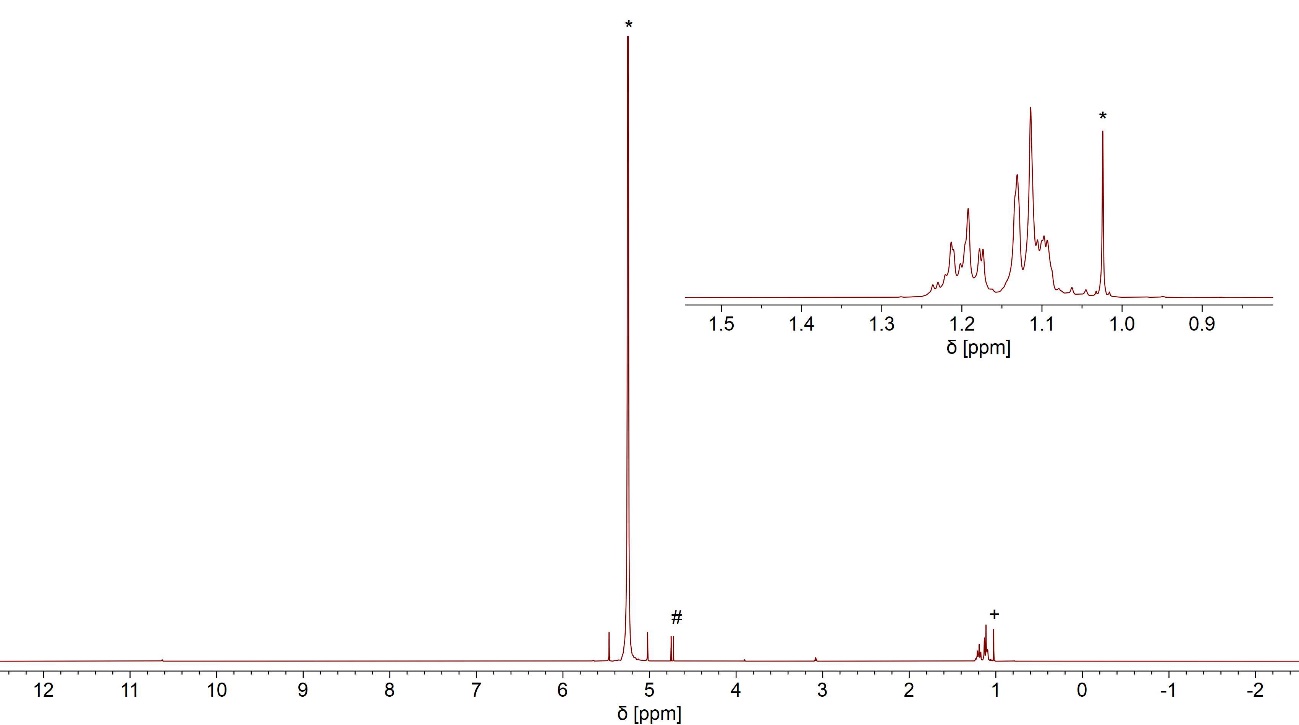


Figure S 88: ^1^H NMR spectrum (401 MHz, CH_2_Cl_2_, external lock acetone-d6, 18 °C) of EtSi(OTeF_5_)_3_ (*: solvent, #: ext. (CH_3_O)_3_PO, +: ext. Si(CH_3_)_4_).


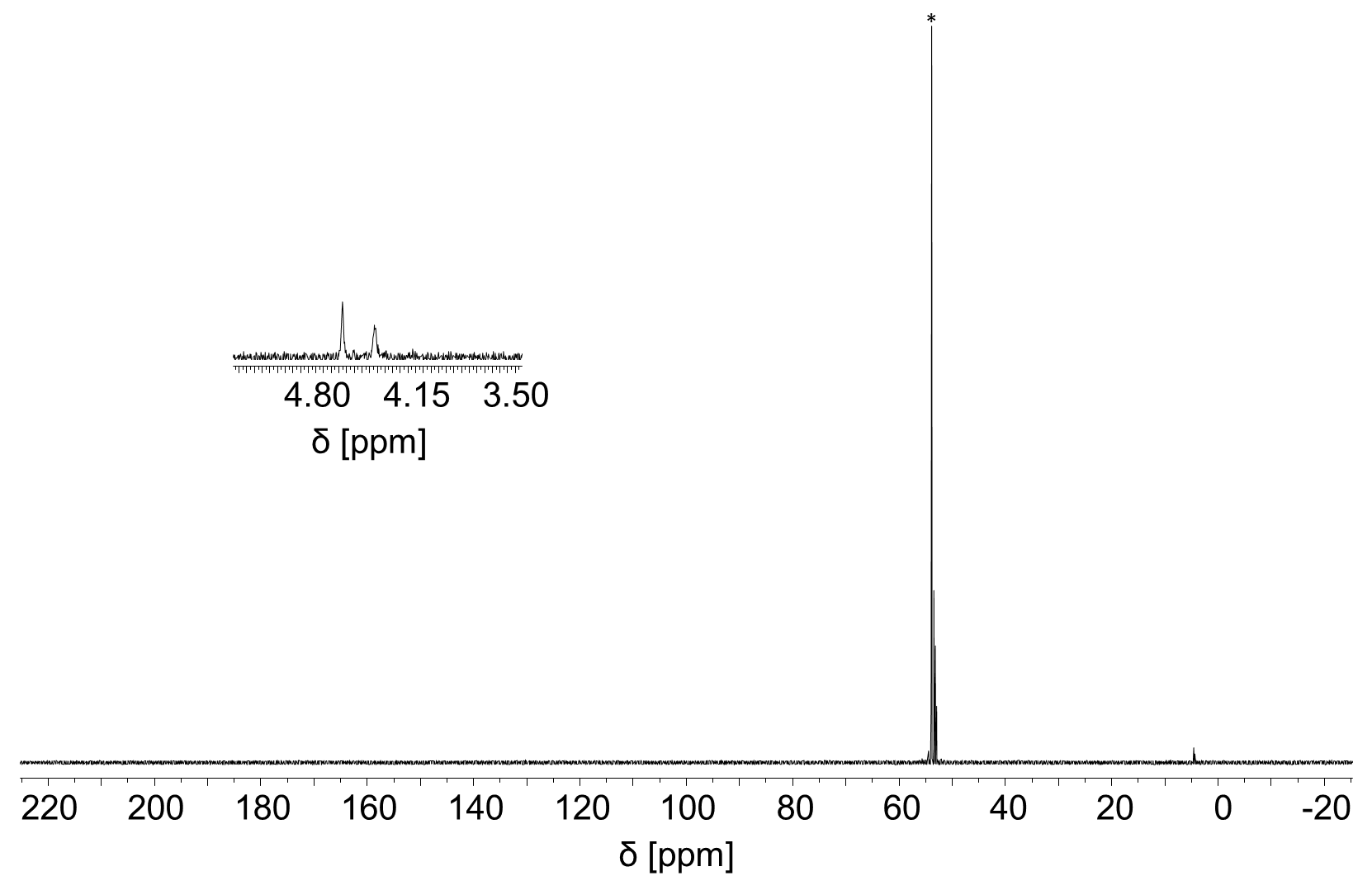


Figure S 89: ^13^C NMR spectrum (100 MHz, CH_2_Cl_2_, external lock acetone-d6, 20 °C) of EtSi(OTeF_5_)_3_ (*: solvent).


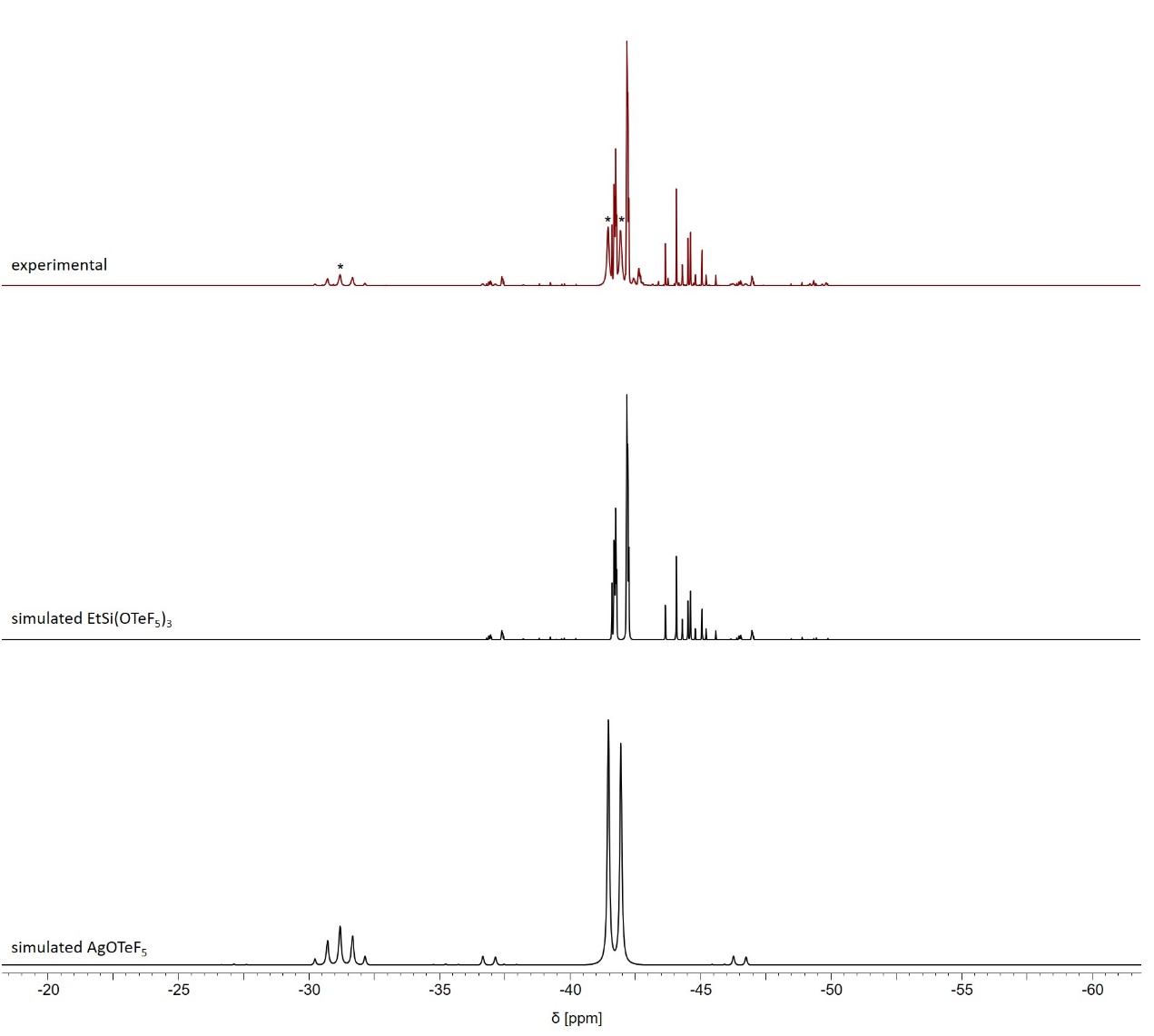


Figure S 90: Experimental (top) ^19^F NMR spectrum (377 MHz, CH_2_Cl_2_, external lock acetone-d6, 19 °C) and simulated (middle) ^19^F NMR spectrum of EtSi(OTeF_5_)_3_ as well as simulated (bottom) ^19^F NMR spectrum of AgOTeF_5_ (*: AgOTeF_5_).


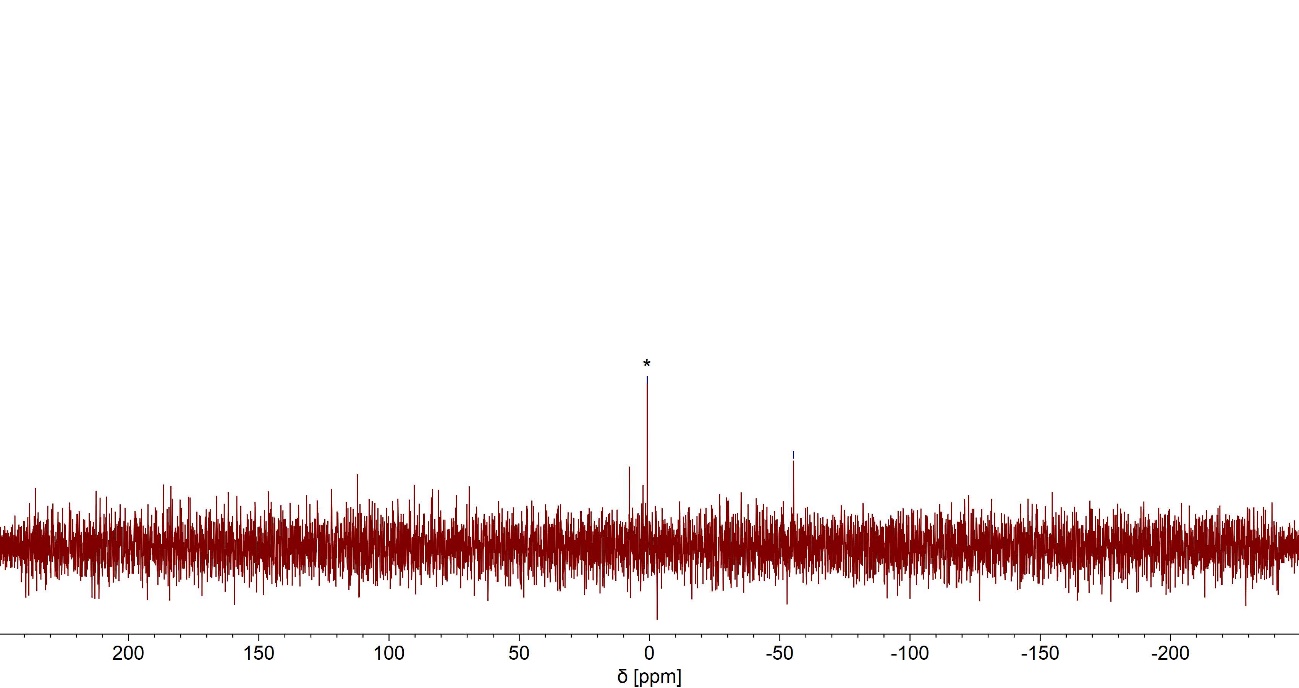


Figure S 91: ^29^Si DEPT NMR (80 MHz, CH_2_Cl_2_, external lock acetone-d6, 17 °C) of EtSi(OTeF_5_)_3_ (*: ext. Si(CH_3_)_4_).


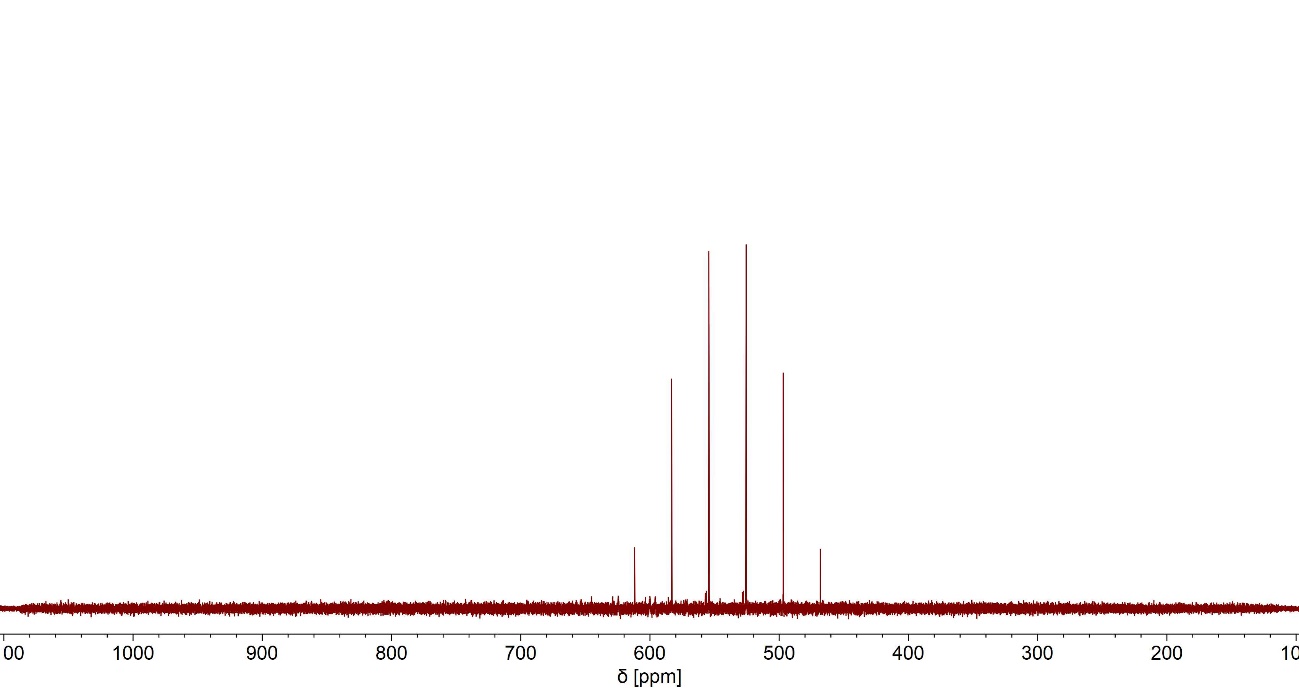


Figure S 92: ^125^Te NMR spectrum (126 MHz, CH_2_Cl_2_, external lock acetone-d6, 20 °C) of EtSi(OTeF_5_)_3_.

## NMR spectra of *^t^*BuSi(OTeF_5_)_3_ (3c)


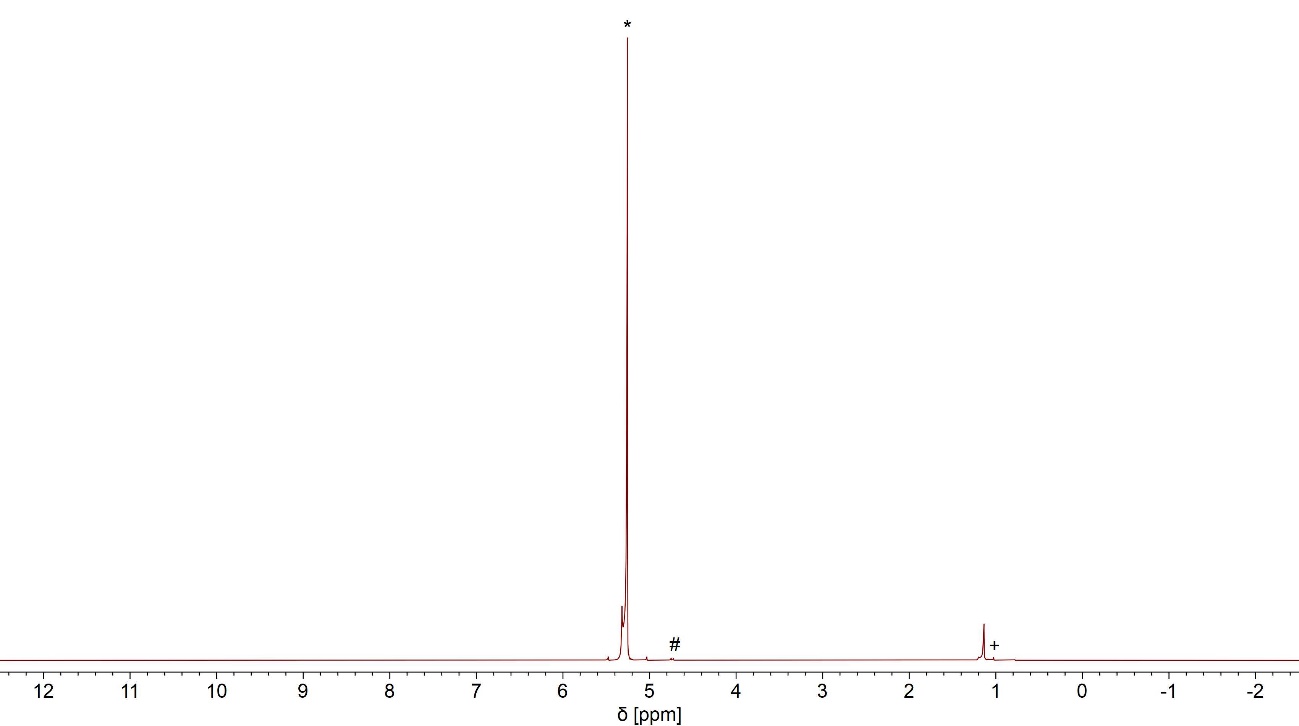


Figure S 93: ^1^H NMR spectrum (401 MHz, CH_2_Cl_2_, external lock acetone-d6, 18 °C) of ^t^BuSi(OTeF_5_)_3_ (*: solvent, #: ext. (CH_3_O)_3_PO, +: ext. Si(CH_3_)_4_).


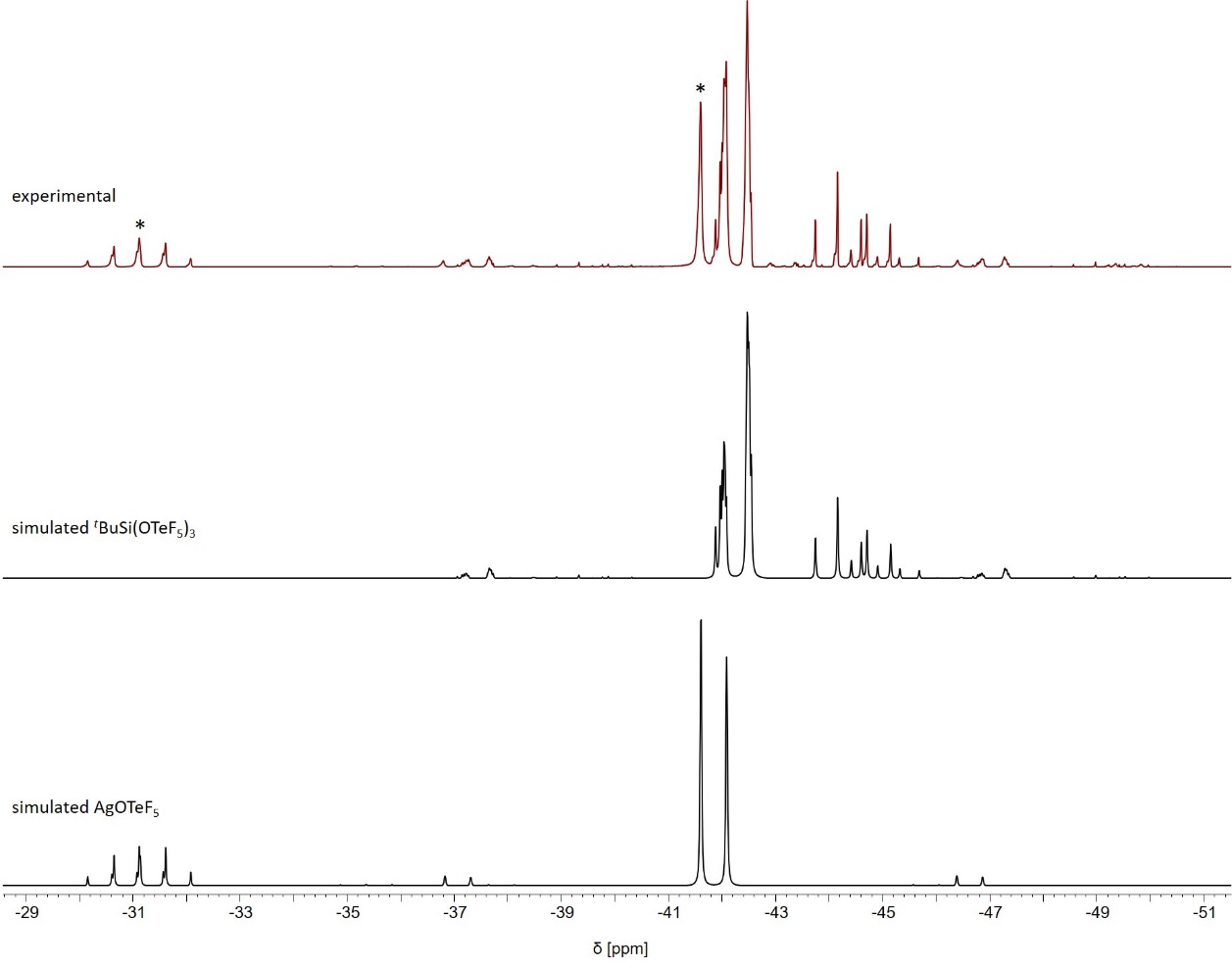


Figure S 94: Experimental (top) ^19^F NMR spectrum (377 MHz, CH_2_Cl_2_, external lock acetone-d6, 19 °C) and simulated (middle) ^19^F NMR spectrum of ^t^BuSi(OTeF_5_)_3_ as well as simulated (bottom) ^19^F NMR spectrum of AgOTeF_5_ (*: AgOTeF_5_).


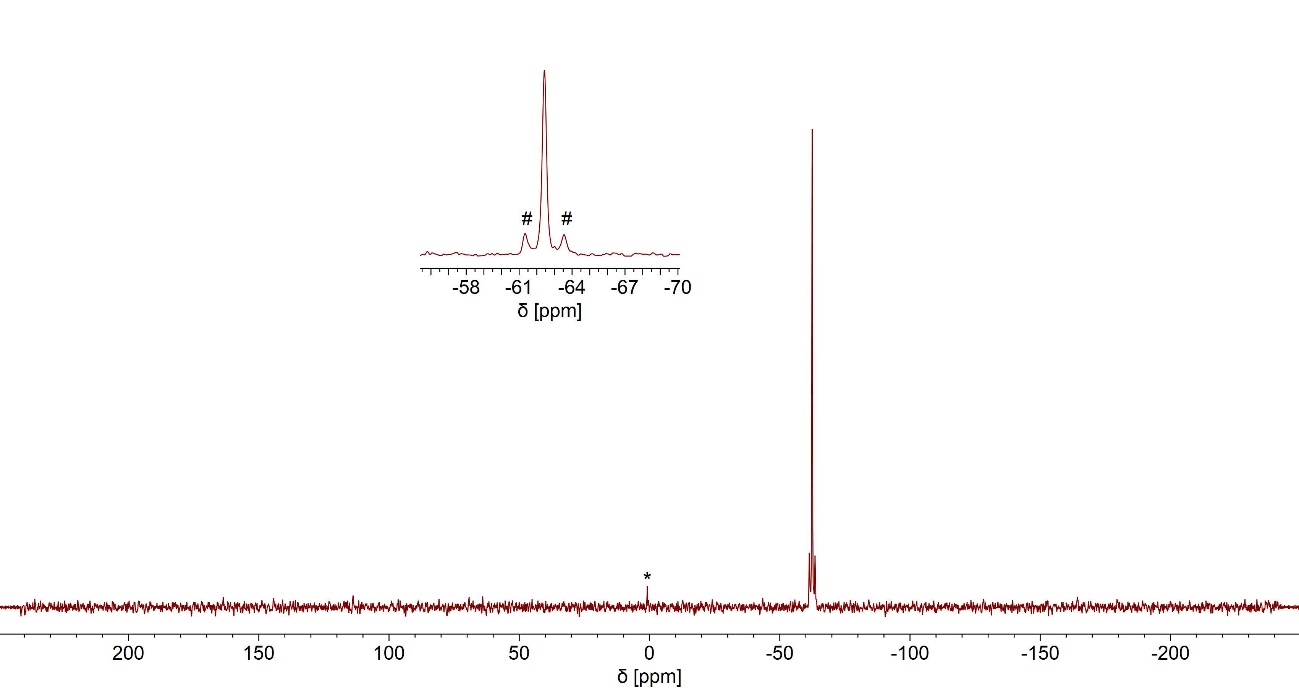


Figure S 95: ^29^Si DEPT NMR spectrum (80 MHz, CH_2_Cl_2_, external lock acetone-d6, 17 °C) of ^t^BuSi(OTeF_5_)_3_ (*: ext. Si(CH_3_)_4_). ^125^Te Satellites are marked with a hash (#).


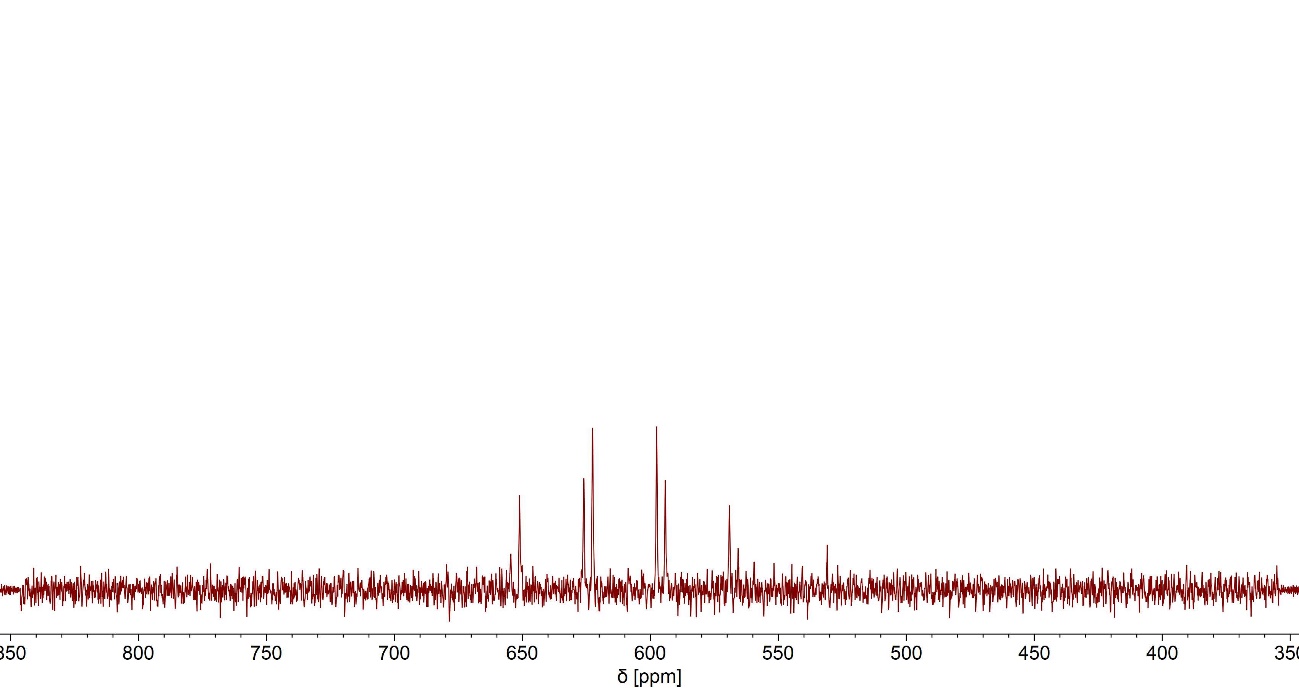


Figure S 96: ^125^Te NMR spectrum (126 MHz, CH_2_Cl_2_, external lock acetone-d6, 17 °C) of ^t^BuSi(OTeF_5_)_3._

## NMR spectra of PhSi(OTeF_5_)_3_ (3d)


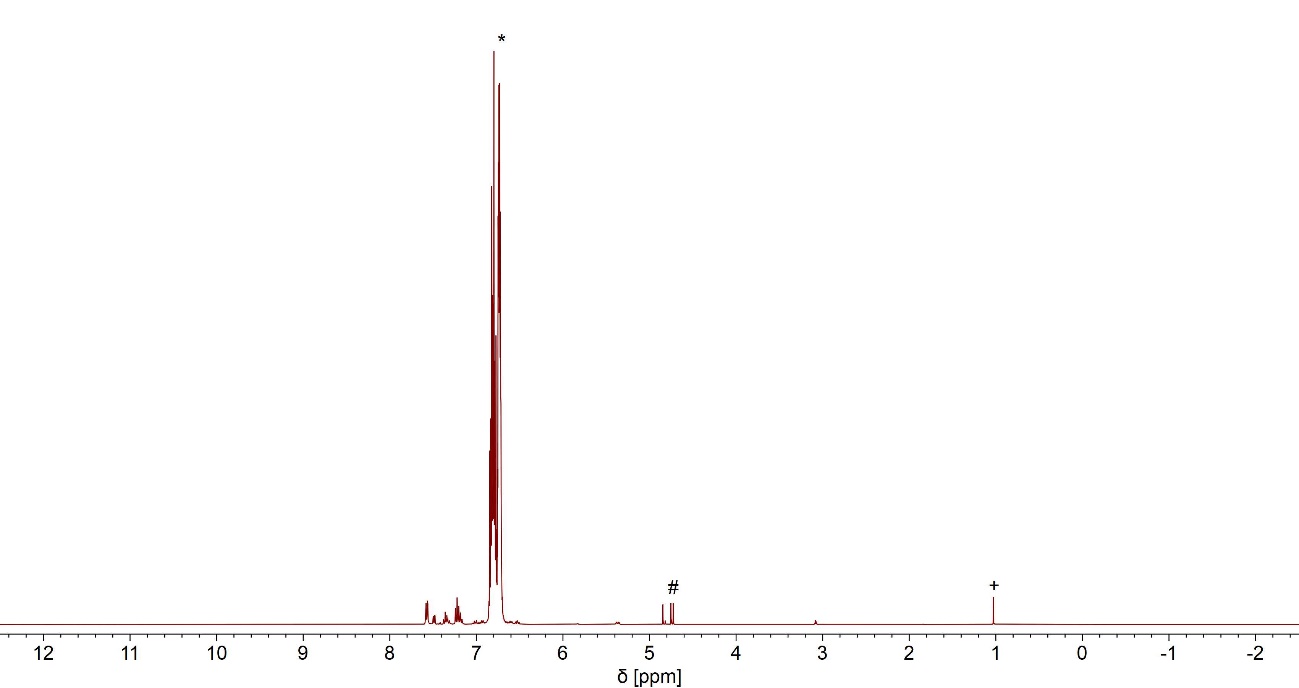


Figure S 97: ^1^H NMR spectrum (401 MHz, o-DFB, external lock acetone-d6, 18 °C) of PhSi(OTeF_5_)_3_ (*: solvent, #: ext. (CH_3_O)_3_PO, +: ext. Si(CH_3_)_4_, °: unknown impurity).


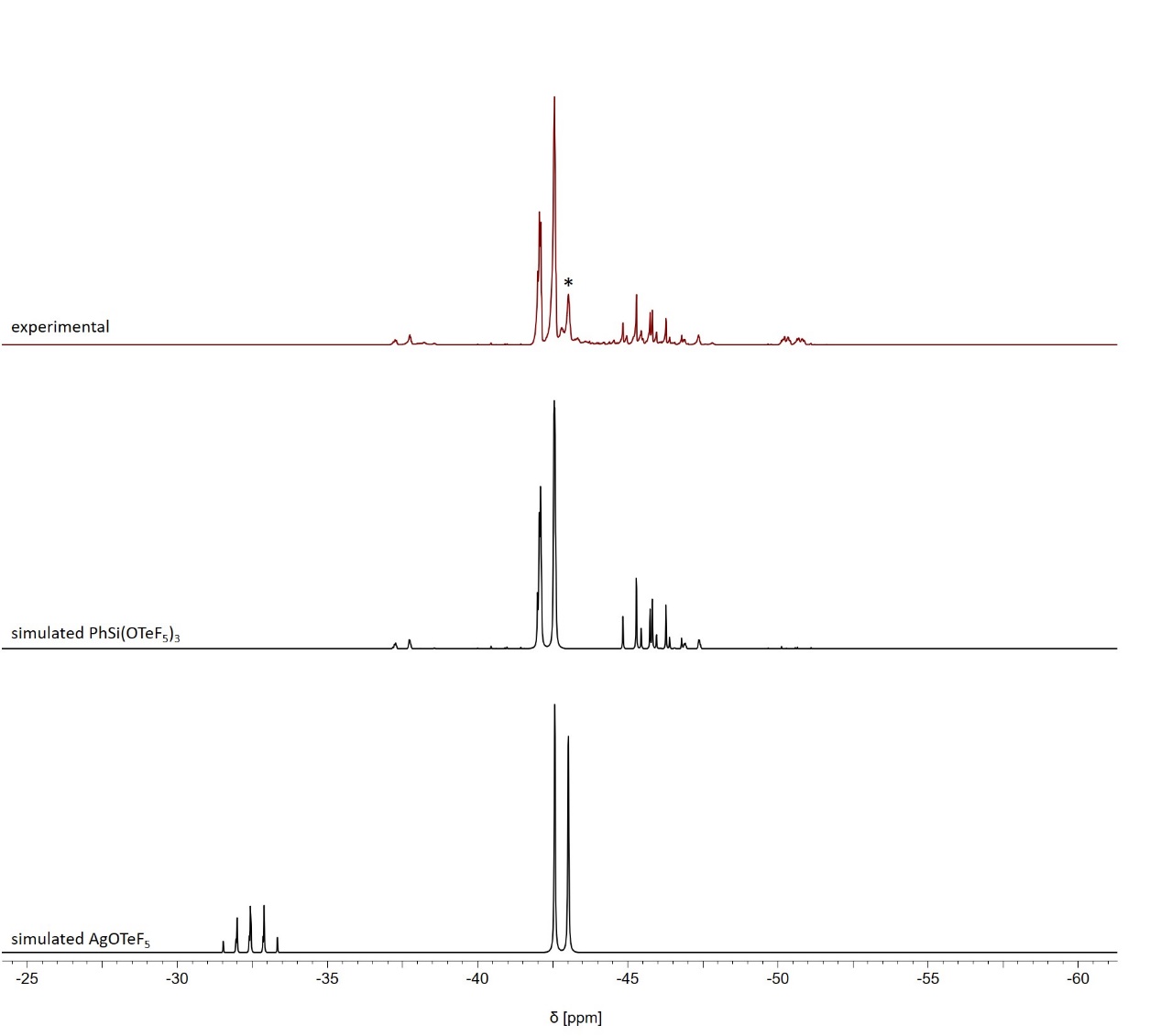


Figure S 98: Experimental (top) ^19^F NMR spectrum (377 MHz, o-DFB, external lock acetone-d6, 19 °C) and simulated (middle) ^19^F NMR spectrum of PhSi(OTeF_5_)_3_ as well as simulated (bottom) ^19^F NMR spectrum of AgOTeF_5_ (*: AgOTeF_5_).


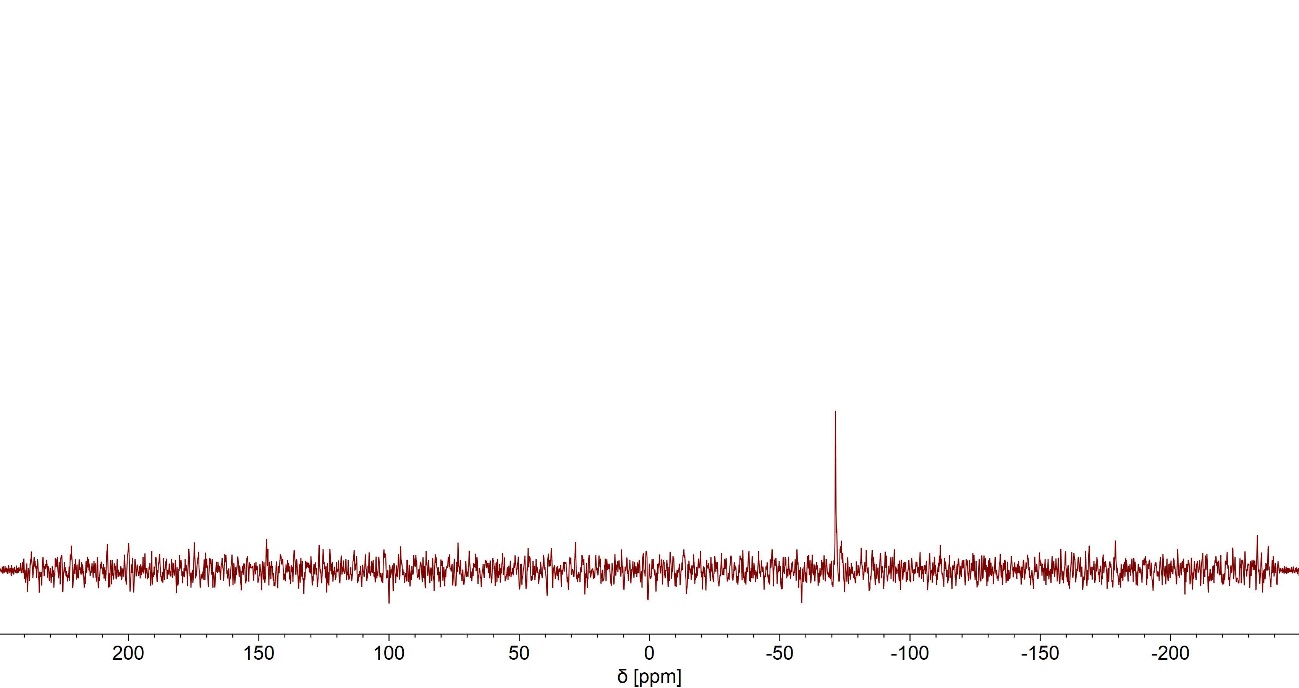


Figure S 99: ^29^Si DEPT NMR spectrum (80 MHz, o-DFB, external lock acetone-d6, 17 °C) of PhSi(OTeF_5_)_3_  (*: ext. Si(CH_3_)_4_).

# Vibrational Spectra

## Infrared spectrum of Ph_3_SiOTeF_5_ with AgOTeF_5_ (1f)


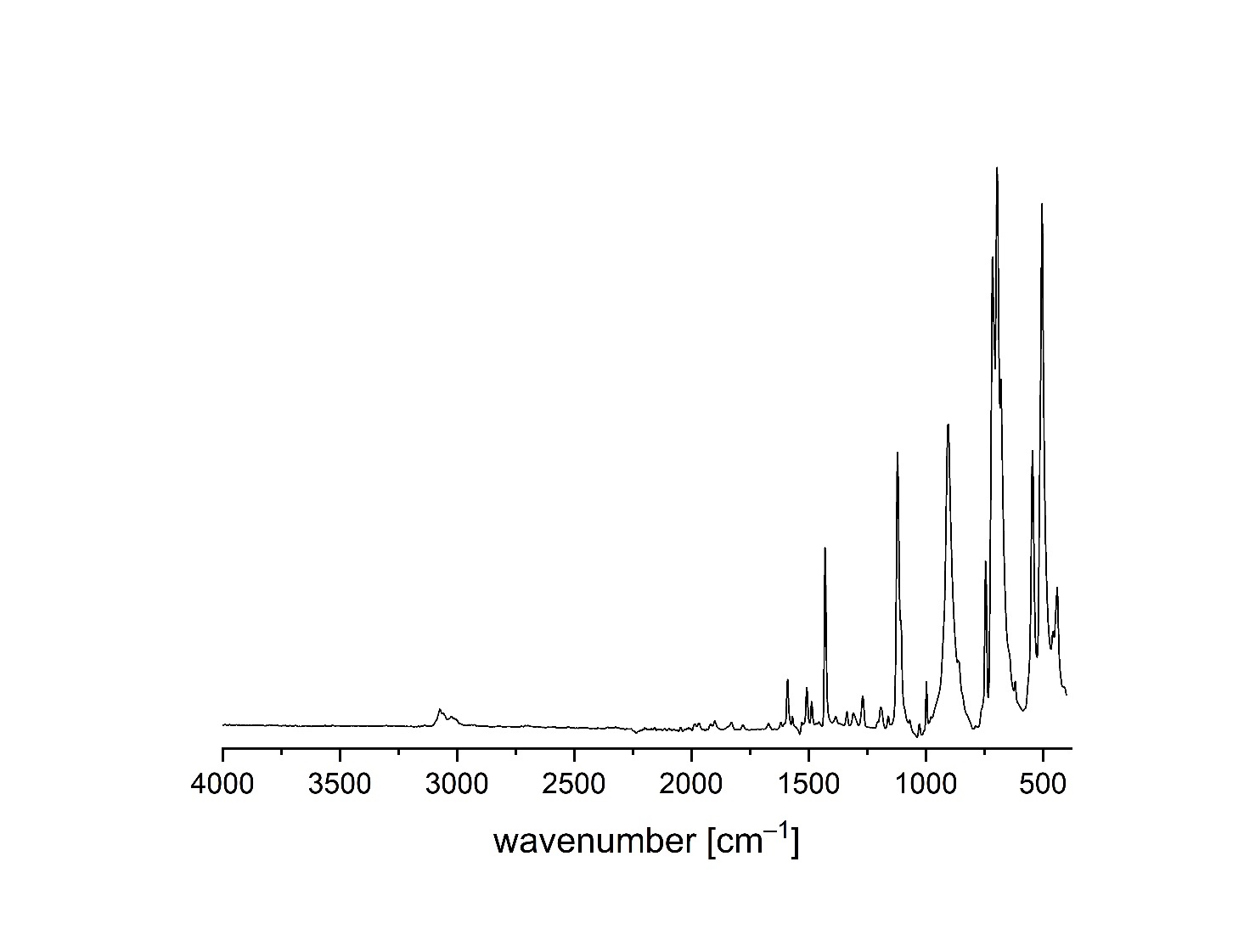


Figure S 107: IR (ATR, 25 °C) spectrum of Ph_3_SiOTeF_5_.

## Infrared spectrum of Ph_2_Si(OTeF_5_)_2_ (2d)

Figure S 108: IR (ATR, 25 °C) spectrum of Ph_2_Si(OTeF_5_)_2_.

## Infrared spectrum of *^t^*BuSi(OTeF_5_)_3_ (3c)

Figure S 109: IR (ATR, 25 °C) spectrum of ^t^BuSi(OTeF_5_)_3_.

## Raman spectrum of Ph_3_SiOTeF_5_ (1f)

Figure S 111: Raman spectrum of Ph_3_SiOTeF_5_.

## Raman spectrum of Ph_2_Si(OTeF_5_)_2_ (2d)

Figure S 112: Raman spectrum of Ph_2_Si(OTeF_5_)_2_.

## Raman spectrum of *^t^*BuSi(OTeF_5_)_3_ in CH_2_Cl_2_ (3c)

Figure S 113: Raman spectrum of ^t^BuSi(OTeF_5_)_3_ in CH_2_Cl_2_.

# Crystal data

Table S1. Crystal data and structure refinement for Ph_3_SiOTeF_5_ (**1**), Ph_2_Si(OTeF_5_)_2_ (**2**) and ^t^BuSi(TteF_5_)_3_ (**3**).

|  | **Ph_3_SiOTeF_5_ (1f)** | **Ph_2_SiO(TeF_5_)_2_ (2d)** | ***^t^*BuSiO(TeF_5_)_3_ (3c)** |
| --- | --- | --- | --- |
| CCDC number | 2407211 | 2407212 | 2499079 |
| empirical formula | C_18_H_15_F_5_OSiTe | C_12_H_10_F_10_O_2_SiTe_2_ | C_4_H_9_F_15_O_3_SiTe_3_ |
| formula weight [g mol^–1^] | 497.99 | 659.49 | 801.00 |
| temperature [K] | 100 | 135 | 100 |
| crystal system | monoclinic | monoclinic | monoclinic |
| space group | *P*2_1_/*c* | *P*2_1_/*n* | *P*2_1_/*c* |
| a [pm] | 10.1733(5) | 9.7567(5) | 10.3161(5) |
| b [pm] | 8.7688(4) | 19.7711(9) | 9.8886(5) |
| c [pm] | 20.6112(11) | 10.3412(6) | 17.6328(8) |
| α [°] | 90 | 90 | 90 |
| β [°] | 90.952(2) | 116.620(2) | 91.221(2) |
| γ [°] | 90 | 90 | 90 |
| volume [Å^3^] | 1838.42(16) | 1783.37(16) | 1798.35(15) |
| *Z* | 4 | 4 | 4 |
| ρ_calcd_ [g/cm^–3]^ | 1.799 | 2.456 | 2.958 |
| μ [mm^‑1^] | 1.735 | 3.444 | 5.052 |
| F(000) | 968.0 | 1224.0 | 1448.0 |
| dimension [mm^3^] | 0.181 × 0.133 × 0.091 | 0.196 × 0.196 × 0.147 | 0.201 × 0.189 × 0.182 |
| radiation | MoK_α_ (λ = 0.71073) | MoK_α_ (λ = 0.71073) | MoK_α_ (λ = 0.71073) |
| 2Θ range for data collection [°] | 5.048 to 52.742 | 4.774 to 52.76 | 4.622 to 52.73 |
| index ranges | –12 ≤ h ≤ 12,  –10 ≤ k ≤ 10,  –25 ≤ l ≤ 25 | -12 ≤ h ≤ 12,  -23 ≤ k ≤ 24,  -12 ≤ l ≤ 12 | -12 ≤ h ≤ 12,  -12 ≤ k ≤ 12,  -22 ≤ l ≤ 22 |
| reflections collected | 33890 3754 | 25429 3637 | 37976 3674 |
| independent reflections | [*R*_int_ = 0.0372, *R*_sigma_ = 0.0182] | [*R*_int_ = 0.0342, *R*_sigma_ = 0.0217] | [*R*_int_ = 0.0340, *R*_sigma_ = 0.0148] |
| data/restraints/parameters | 3754/0/235 | 3637/0/244 | 3674/0/238 |
| goodness-of-fit on *F*^2^ | 1.251 | 1.284 | 1.183 |
| final *R* indexes [I > 2*σ*(I)] | *R*_1_ = 0.0304, *wR*_2_ = 0.0609 | *R*_1_ = 0.0331, *wR*_2_ = 0.0730 | *R*_1_ = 0.0254, *wR*_2_ = 0.0590 |
| final *R* indexes [all data] | *R*_1_ = 0.0401,  *wR*_2_ = 0.0685 | *R*_1_ = 0.0405, *wR*_2_ = 0.0783 | *R*_1_ = 0.0304, *wR*_2_ = 0.0625 |
| largest diff. peak/hole [e Å^-3^] | 1.28/-0.78 | 0.87/-1.22 | 1.41/-1.19 |

# Quantum-chemical calculations

The optimized geometries of the theoretically studied compounds are listed below as minima on their respective potential energy surface at the RI-B3LYP(D3BJ)/def2-TZVPP level. All xyz coordinates are given in ångstrom.

## Me_3_SiOTeF_5_

| Figure S 116: Representation of the B3LYP/def2-TZVPP structure of Me_3_SiOTeF_5_. | Si | 0.076401 | 0.609138 | -0.483509 |
| --- | --- | --- | --- | --- |
|  | O | 0.525590 | -0.955498 | -0.235992 |
|  | Te | 0.148350 | -2.321389 | 1.178364 |
|  | F | 0.895129 | -3.689476 | -0.018304 |
|  | F | -1.654193 | -2.626934 | 0.408043 |
|  | F | -0.208117 | -3.704779 | 2.570078 |
|  | F | 1.941616 | -2.017201 | 1.954872 |
|  | F | -0.613052 | -0.890503 | 2.359827 |
|  | C | 0.855281 | 1.759363 | 0.785349 |
|  | H | 1.943073 | 1.661057 | 0.783368 |
|  | H | 0.607637 | 2.805841 | 0.584879 |
|  | H | 0.499874 | 1.504536 | 1.784964 |
|  | C | -1.792161 | 0.818114 | -0.457588 |
|  | H | -2.266781 | 0.156045 | -1.184499 |
|  | H | -2.186542 | 0.561443 | 0.526722 |
|  | H | -2.080961 | 1.847290 | -0.689921 |
|  | C | 0.725328 | 1.055033 | -2.200161 |
|  | H | 0.488069 | 2.090399 | -2.460464 |
|  | H | 1.809665 | 0.933130 | -2.246833 |
|  | H | 0.285794 | 0.404390 | -2.959196 |
|  |  |  |  |  |

## Et_3_SiOTeF_5_

| Figure S 117: Representation of the B3LYP/def2-TZVPP structure of Et_3_SiOTeF_5_. | Si | 0.271213 | -0.005186 | -0.085299 |
| --- | --- | --- | --- | --- |
|  | C | 0.782215 | 0.950066 | 1.447710 |
|  | C | 2.293768 | 1.217000 | 1.560895 |
|  | H | 0.438214 | 0.399500 | 2.328248 |
|  | H | 0.235343 | 1.896821 | 1.460551 |
|  | H | 2.525861 | 1.777979 | 2.467480 |
|  | H | 2.867637 | 0.289525 | 1.598808 |
|  | H | 2.667338 | 1.798669 | 0.716120 |
|  | C | 0.927833 | -1.762407 | -0.064758 |
|  | C | 0.505562 | -2.660799 | -1.238549 |
|  | H | 0.628336 | -2.220511 | 0.882977 |
|  | H | 2.020898 | -1.697389 | -0.022651 |
|  | H | 0.921592 | -3.664125 | -1.134165 |
|  | H | -0.578813 | -2.762010 | -1.296477 |
|  | H | 0.850419 | -2.263886 | -2.194568 |
|  | C | 0.681400 | 0.892533 | -1.683115 |
|  | C | 0.144831 | 2.326750 | -1.802452 |
|  | H | 0.308129 | 0.285589 | -2.512884 |
|  | H | 1.772744 | 0.888374 | -1.783668 |
|  | H | 0.422456 | 2.772077 | -2.759289 |
|  | H | -0.943194 | 2.350718 | -1.732373 |
|  | H | 0.538967 | 2.973416 | -1.016619 |
|  | O | -1.446218 | -0.058858 | -0.141216 |
|  | Te | -2.863106 | -0.529571 | 0.958007 |
|  | F | -4.042701 | 0.400773 | -0.131194 |
|  | F | -2.659811 | 0.980899 | 2.031247 |
|  | F | -4.308542 | -0.994897 | 2.026143 |
|  | F | -3.185090 | -2.083845 | -0.013537 |
|  | F | -1.777281 | -1.497206 | 2.134629 |
|  |  |  |  |  |

## Me_2_PrSiOTeF_5_

| Figure S 118: Representation of the B3LYP/def2-TZVPP structure of Me_2_PrSiOTeF_5_. | Si | 0.023442 | 0.216605 | -0.388007 |
| --- | --- | --- | --- | --- |
|  | C | 0.074092 | -1.138175 | 0.908048 |
|  | C | -0.016488 | -2.574381 | 0.363674 |
|  | H | -0.745287 | -0.956917 | 1.611221 |
|  | H | 0.994893 | -1.017846 | 1.488045 |
|  | C | 0.015937 | -3.630741 | 1.469202 |
|  | H | -0.936310 | -2.693292 | -0.215886 |
|  | H | 0.805925 | -2.760545 | -0.332908 |
|  | H | -0.053431 | -4.639262 | 1.057700 |
|  | H | 0.941267 | -3.567155 | 2.045260 |
|  | H | -0.815430 | -3.494764 | 2.163812 |
|  | C | 1.520916 | 0.214903 | -1.502523 |
|  | H | 2.445504 | 0.269634 | -0.925307 |
|  | H | 1.552686 | -0.700290 | -2.098413 |
|  | H | 1.504909 | 1.057525 | -2.195928 |
|  | C | -1.570878 | 0.228547 | -1.358672 |
|  | H | -2.436701 | 0.304015 | -0.698576 |
|  | H | -1.606278 | 1.064271 | -2.059183 |
|  | H | -1.671457 | -0.691793 | -1.938421 |
|  | O | 0.080880 | 1.620013 | 0.596019 |
|  | Te | -0.002173 | 3.460396 | 0.375331 |
|  | F | 0.165258 | 3.701986 | 2.206160 |
|  | F | -1.857411 | 3.473050 | 0.534382 |
|  | F | -0.087119 | 5.306211 | 0.194650 |
|  | F | 1.843776 | 3.604707 | 0.184555 |
|  | F | -0.170522 | 3.343296 | -1.484234 |
|  |  |  |  |  |

## *^i^*Pr_3_SiOTeF_5_

| Figure S 119: Representation of the B3LYP/def2-TZVPP structure of ^i^Pr_3_SiOTeF_5_. | Si | 0.063737 | -0.088359 | -0.035509 |
| --- | --- | --- | --- | --- |
|  | C | 0.950403 | 0.552364 | -1.591572 |
|  | C | 2.337022 | -0.082685 | -1.811889 |
|  | C | 1.058060 | 2.085539 | -1.670506 |
|  | H | 0.308389 | 0.225609 | -2.417766 |
|  | H | 2.302513 | -1.171746 | -1.843569 |
|  | H | 2.758739 | 0.258327 | -2.760789 |
|  | H | 3.043054 | 0.202499 | -1.029302 |
|  | H | 0.097817 | 2.586729 | -1.556130 |
|  | H | 1.729047 | 2.476421 | -0.902754 |
|  | H | 1.466778 | 2.385553 | -2.638701 |
|  | C | 0.515211 | 0.912707 | 1.510534 |
|  | C | 1.948486 | 0.608711 | 1.987727 |
|  | C | -0.478783 | 0.814944 | 2.680242 |
|  | H | 0.500798 | 1.953490 | 1.166983 |
|  | H | 2.693664 | 0.759436 | 1.205206 |
|  | H | 2.217864 | 1.262542 | 2.821078 |
|  | H | 2.044734 | -0.420107 | 2.341895 |
|  | H | -1.498433 | 1.058108 | 2.383585 |
|  | H | -0.493470 | -0.182536 | 3.120099 |
|  | H | -0.193511 | 1.513425 | 3.471285 |
|  | C | 0.239329 | -1.971933 | 0.115658 |
|  | C | -0.253280 | -2.689445 | -1.154566 |
|  | C | -0.433068 | -2.574991 | 1.359863 |
|  | H | 1.319247 | -2.145891 | 0.202461 |
|  | H | 0.237261 | -2.330689 | -2.060205 |
|  | H | -0.061605 | -3.762950 | -1.081855 |
|  | H | -1.328414 | -2.557619 | -1.286893 |
|  | H | -0.008709 | -2.193698 | 2.288245 |
|  | H | -1.505312 | -2.370531 | 1.373647 |
|  | H | -0.309241 | -3.661228 | 1.366762 |
|  | O | -1.632219 | 0.064143 | -0.276712 |
|  | Te | -3.001821 | 1.197291 | -0.782649 |
|  | F | -4.006283 | -0.242005 | -1.388539 |
|  | F | -2.305796 | 1.436900 | -2.496307 |
|  | F | -4.406566 | 2.303754 | -1.285802 |
|  | F | -3.812037 | 1.055590 | 0.885184 |
|  | F | -2.103609 | 2.732333 | -0.208437 |
|  |  |  |  |  |

## *^t^*BuMe_2_SiOTeF_5_

| Figure S 120: Representation of the B3LYP/def2-TZVPP structure of ^t^BuMe_2_SiOTeF_5_. | Si | -0.375106 | 0.200572 | -0.428988 |
| --- | --- | --- | --- | --- |
|  | C | -2.230788 | 0.146810 | -0.213940 |
|  | H | -2.668638 | 1.131231 | -0.387624 |
|  | H | -2.680654 | -0.542507 | -0.931693 |
|  | H | -2.518210 | -0.175611 | 0.787305 |
|  | C | 0.087213 | 0.920734 | -2.090165 |
|  | H | 1.165989 | 1.041630 | -2.195831 |
|  | H | -0.260170 | 0.278602 | -2.902144 |
|  | H | -0.375102 | 1.900082 | -2.225575 |
|  | C | 0.473372 | -1.465219 | -0.067872 |
|  | C | 2.003818 | -1.313695 | -0.150234 |
|  | C | 0.089983 | -1.966500 | 1.336993 |
|  | C | 0.007074 | -2.493507 | -1.118854 |
|  | H | 2.332579 | -0.988543 | -1.138982 |
|  | H | 2.379852 | -0.597994 | 0.581641 |
|  | H | 2.483493 | -2.276499 | 0.051276 |
|  | H | -0.985089 | -2.127554 | 1.436787 |
|  | H | 0.581585 | -2.923760 | 1.536813 |
|  | H | 0.399727 | -1.268717 | 2.115901 |
|  | H | 0.482521 | -3.460244 | -0.925842 |
|  | H | -1.072654 | -2.653882 | -1.090045 |
|  | H | 0.278273 | -2.198110 | -2.134412 |
|  | O | 0.284844 | 1.213992 | 0.788237 |
|  | Te | 0.060331 | 2.938655 | 1.434101 |
|  | F | 1.135979 | 2.547129 | 2.894530 |
|  | F | -1.451343 | 2.466322 | 2.414946 |
|  | F | -0.134365 | 4.655698 | 2.113210 |
|  | F | 1.545553 | 3.558314 | 0.499673 |
|  | F | -1.040066 | 3.452572 | 0.010788 |
|  |  |  |  |  |

## Ph_3_SiOTeF_5_

| Figure S 121: Representation of the B3LYP/def2-TZVPP structure of Ph_3_SiOTeF_5_. | Si | 0.148007 | 0.083938 | -0.223768 |
| --- | --- | --- | --- | --- |
|  | C | 0.241736 | 0.943491 | 1.435457 |
|  | C | -0.499173 | 2.108433 | 1.679796 |
|  | C | 1.059642 | 0.457699 | 2.467690 |
|  | C | -0.423218 | 2.766078 | 2.902688 |
|  | H | -1.146030 | 2.506526 | 0.908683 |
|  | C | 1.138518 | 1.113472 | 3.689862 |
|  | H | 1.646672 | -0.439126 | 2.318124 |
|  | C | 0.396787 | 2.269810 | 3.909334 |
|  | H | -1.003768 | 3.664397 | 3.068256 |
|  | H | 1.778845 | 0.722859 | 4.470145 |
|  | H | 0.457921 | 2.781033 | 4.861482 |
|  | C | -1.218847 | 0.771662 | -1.302643 |
|  | C | -2.467268 | 0.138046 | -1.356231 |
|  | C | -1.039695 | 1.941150 | -2.055052 |
|  | C | -3.503051 | 0.657809 | -2.125080 |
|  | H | -2.633764 | -0.776676 | -0.800436 |
|  | C | -2.072344 | 2.461523 | -2.826227 |
|  | H | -0.082008 | 2.445972 | -2.049464 |
|  | C | -3.306843 | 1.821248 | -2.860807 |
|  | H | -4.459120 | 0.151258 | -2.154483 |
|  | H | -1.912195 | 3.363356 | -3.402932 |
|  | H | -4.110517 | 2.224659 | -3.463221 |
|  | C | 0.051272 | -1.774792 | -0.053403 |
|  | C | 0.479404 | -2.608589 | -1.097054 |
|  | C | -0.501342 | -2.378102 | 1.084627 |
|  | C | 0.368883 | -3.990190 | -1.002634 |
|  | H | 0.911907 | -2.178902 | -1.991778 |
|  | C | -0.619878 | -3.760829 | 1.179157 |
|  | H | -0.838479 | -1.765466 | 1.911498 |
|  | C | -0.182318 | -4.568809 | 0.136152 |
|  | H | 0.714223 | -4.614810 | -1.816235 |
|  | H | -1.047923 | -4.205638 | 2.068261 |
|  | H | -0.267719 | -5.645248 | 0.210498 |
|  | O | 1.524780 | 0.462126 | -1.163343 |
|  | Te | 3.379363 | 0.472012 | -1.095441 |
|  | F | 3.435172 | 1.544215 | -2.608958 |
|  | F | 3.484728 | -1.041057 | -2.173072 |
|  | F | 5.236714 | 0.496657 | -1.081779 |
|  | F | 3.426151 | 1.989045 | -0.020648 |
|  | F | 3.454774 | -0.610242 | 0.422977 |
|  |  |  |  |  |

## Me_2_Si(OTeF_5_)_2_

| Figure S 122: Representation of the B3LYP/def2-TZVPP structure of Me_2_Si(OTeF_5_)_2_. | Si | -0.753552 | 0.077006 | -0.155264 |
| --- | --- | --- | --- | --- |
|  | C | -1.172779 | 0.072530 | 1.645892 |
|  | H | -1.561431 | -0.901584 | 1.947252 |
|  | H | -0.299964 | 0.293094 | 2.260985 |
|  | H | -1.938109 | 0.821190 | 1.858090 |
|  | C | -2.131632 | -0.339531 | -1.315642 |
|  | H | -2.954742 | 0.368242 | -1.205575 |
|  | H | -1.789614 | -0.308447 | -2.350559 |
|  | H | -2.513624 | -1.340953 | -1.109625 |
|  | O | -0.208266 | 1.582679 | -0.655977 |
|  | Te | 1.048810 | 2.856406 | -0.116401 |
|  | F | 0.334533 | 4.066319 | -1.321305 |
|  | F | 2.262518 | 2.289132 | -1.397297 |
|  | F | 2.272324 | 4.149917 | 0.396042 |
|  | F | -0.092277 | 3.497497 | 1.206240 |
|  | F | 1.819345 | 1.704487 | 1.129686 |
|  | O | 0.574126 | -0.914493 | -0.462218 |
|  | Te | 1.094311 | -2.670117 | -0.121755 |
|  | F | 2.838000 | -2.267386 | -0.582526 |
|  | F | 0.734142 | -3.164249 | -1.873462 |
|  | F | 1.615527 | -4.418112 | 0.202922 |
|  | F | 1.465456 | -2.293095 | 1.658912 |
|  | F | -0.643103 | -3.160532 | 0.361585 |
|  |  |  |  |  |

## [Me_2_Si(OTeF_5_)_2_F]^–^

| Figure S 123: Representation of the B3LYP/def2-TZVPP structure of [Me_2_Si(OTeF_5_)_2_F]^–^. | Si | -0.205329 | -0.189157 | -0.040911 |
| --- | --- | --- | --- | --- |
|  | C | -1.392514 | 1.118910 | -0.668612 |
|  | H | -1.023130 | 2.114367 | -0.420941 |
|  | H | -1.544484 | 1.054419 | -1.743044 |
|  | H | -2.352722 | 0.995425 | -0.165214 |
|  | C | 1.644852 | 0.052306 | 0.106900 |
|  | H | 2.026210 | -0.591014 | 0.901657 |
|  | H | 2.173156 | -0.179094 | -0.815766 |
|  | H | 1.868724 | 1.081338 | 0.389829 |
|  | F | -0.810707 | -1.567955 | 0.535973 |
|  | O | -0.148067 | -0.920965 | -1.791559 |
|  | Te | 0.328513 | -2.490472 | -2.593019 |
|  | F | -0.499128 | -1.993969 | -4.202468 |
|  | F | -1.217934 | -3.429956 | -2.104768 |
|  | F | 0.812103 | -4.063039 | -3.499228 |
|  | F | 1.952875 | -1.805498 | -3.249876 |
|  | F | 1.251062 | -3.220860 | -1.128937 |
|  | O | -0.444124 | 0.436766 | 1.828371 |
|  | Te | -0.410318 | 1.922405 | 2.863510 |
|  | F | -1.199034 | 1.059635 | 4.329639 |
|  | F | -2.105518 | 2.547923 | 2.333335 |
|  | F | -0.385709 | 3.443529 | 3.966944 |
|  | F | 1.288692 | 1.573342 | 3.589705 |
|  | F | 0.392531 | 3.051614 | 1.578480 |
|  |  |  |  |  |

## Et_2_Si(OTeF_5_)_2_

| Figure S 124: Representation of the B3LYP/def2-TZVPP structure of Et_2_Si(OTeF_5_)_2_. | Si | 0.246875 | 0.399374 | -0.006314 |
| --- | --- | --- | --- | --- |
|  | C | 0.423686 | 1.209149 | 1.658886 |
|  | C | -0.809410 | 1.973184 | 2.172136 |
|  | H | 1.279828 | 1.888099 | 1.586274 |
|  | H | 0.715067 | 0.439076 | 2.377921 |
|  | H | -0.598054 | 2.432817 | 3.138196 |
|  | H | -1.106118 | 2.771204 | 1.490046 |
|  | H | -1.667697 | 1.315147 | 2.306778 |
|  | C | -0.281652 | 1.509428 | -1.401893 |
|  | C | -0.244383 | 0.900013 | -2.812995 |
|  | H | 0.368971 | 2.389190 | -1.355509 |
|  | H | -1.287201 | 1.873375 | -1.169893 |
|  | H | -0.557974 | 1.634662 | -3.555280 |
|  | H | 0.760080 | 0.569315 | -3.076015 |
|  | H | -0.909567 | 0.041219 | -2.900069 |
|  | O | 1.723323 | -0.237944 | -0.496300 |
|  | Te | 3.011767 | -1.446183 | 0.108657 |
|  | F | 4.030263 | -1.106451 | -1.400820 |
|  | F | 2.187656 | -2.863871 | -0.757426 |
|  | F | 4.321694 | -2.627484 | 0.677456 |
|  | F | 3.916856 | -0.100795 | 1.019167 |
|  | F | 2.058601 | -1.838434 | 1.661756 |
|  | O | -0.726576 | -0.970145 | 0.123649 |
|  | Te | -2.481064 | -1.581245 | 0.104258 |
|  | F | -1.897785 | -3.320917 | 0.332543 |
|  | F | -2.454651 | -1.801741 | -1.740246 |
|  | F | -4.224869 | -2.209770 | 0.092157 |
|  | F | -2.632083 | -1.380345 | 1.944309 |
|  | F | -3.165582 | 0.140076 | -0.121429 |
|  |  |  |  |  |

## [Et_2_Si(OTeF_5_)_2_F]^–^

| Figure S 125: Representation of the B3LYP/def2-TZVPP structure of [Et_2_Si(OTeF_5_)_2_F]^–^. | Si | 0.463192 | -0.187103 | 0.034605 |
| --- | --- | --- | --- | --- |
|  | C | -0.029526 | -1.877399 | -0.650490 |
|  | H | -0.135839 | -2.587596 | 0.168256 |
|  | C | -1.299456 | -1.882489 | -1.516505 |
|  | H | 0.819122 | -2.214237 | -1.250913 |
|  | C | -0.718560 | 1.266289 | 0.252363 |
|  | C | -0.170515 | 2.405904 | 1.128659 |
|  | H | -0.979351 | 1.651540 | -0.733981 |
|  | H | -1.642280 | 0.883793 | 0.692272 |
|  | F | 1.998803 | 0.004904 | 0.490563 |
|  | O | 0.915182 | 0.301826 | -1.760148 |
|  | Te | 2.002423 | 1.421964 | -2.695268 |
|  | F | 1.658300 | 0.527126 | -4.309286 |
|  | F | 3.554295 | 0.392274 | -2.473628 |
|  | F | 3.086534 | 2.552191 | -3.731655 |
|  | F | 0.622611 | 2.635028 | -3.102972 |
|  | F | 2.504692 | 2.512465 | -1.251179 |
|  | O | 0.200389 | -0.722117 | 1.923566 |
|  | Te | -1.029074 | -1.422107 | 3.055432 |
|  | F | 0.167972 | -1.488163 | 4.496944 |
|  | F | -0.627378 | -3.216424 | 2.652474 |
|  | F | -2.276943 | -2.147756 | 4.258107 |
|  | F | -1.656188 | 0.241127 | 3.670910 |
|  | F | -2.447047 | -1.476987 | 1.809748 |
|  | H | -1.500028 | -2.884569 | -1.904181 |
|  | H | -1.201650 | -1.210780 | -2.369423 |
|  | H | -2.175809 | -1.577015 | -0.942559 |
|  | H | 0.734062 | 2.838319 | 0.700653 |
|  | H | 0.069638 | 2.053011 | 2.131059 |
|  | H | -0.907569 | 3.206981 | 1.226578 |
|  |  |  |  |  |

## *^i^*Pr_2_Si(OTeF_5_)_2_

| Figure S 126: Representation of the B3LYP/def2-TZVPP structure of ^i^Pr_2_Si(OTeF_5_)_2_. | Si | 0.066783 | -0.003932 | 0.065101 |
| --- | --- | --- | --- | --- |
|  | O | -0.770225 | 1.342293 | -0.502141 |
|  | Te | -2.131066 | 2.523206 | -0.018603 |
|  | F | -2.289880 | 3.067331 | -1.782823 |
|  | F | -0.897963 | 3.881123 | 0.284428 |
|  | F | -3.470319 | 3.724607 | 0.426406 |
|  | F | -3.445024 | 1.240722 | -0.287001 |
|  | F | -2.036392 | 2.047765 | 1.781513 |
|  | O | -1.099219 | -1.114027 | 0.551988 |
|  | Te | -2.092415 | -2.650904 | 0.231081 |
|  | F | -3.538252 | -1.943413 | 1.144349 |
|  | F | -1.462694 | -3.404265 | 1.805841 |
|  | F | -3.081624 | -4.194221 | -0.047515 |
|  | F | -2.772408 | -1.999636 | -1.371662 |
|  | F | -0.692742 | -3.458521 | -0.697730 |
|  | C | 0.964092 | 0.378953 | 1.669684 |
|  | C | 1.795128 | 1.673248 | 1.660969 |
|  | C | 1.799082 | -0.826167 | 2.142349 |
|  | H | 0.147114 | 0.515136 | 2.387287 |
|  | H | 1.212170 | 2.541985 | 1.355818 |
|  | H | 2.176848 | 1.871943 | 2.664981 |
|  | H | 2.657148 | 1.600654 | 0.996965 |
|  | H | 1.203990 | -1.736565 | 2.223649 |
|  | H | 2.634417 | -1.028578 | 1.468826 |
|  | H | 2.222072 | -0.624111 | 3.128870 |
|  | C | 1.045436 | -0.604637 | -1.421838 |
|  | C | 2.324560 | 0.221435 | -1.652487 |
|  | C | 0.207347 | -0.665168 | -2.711884 |
|  | H | 1.346316 | -1.626899 | -1.163156 |
|  | H | 3.002021 | 0.192263 | -0.799487 |
|  | H | 2.867494 | -0.172535 | -2.514447 |
|  | H | 2.095152 | 1.267480 | -1.865119 |
|  | H | -0.688458 | -1.275702 | -2.611233 |
|  | H | -0.103925 | 0.332424 | -3.023736 |
|  | H | 0.805436 | -1.093289 | -3.519243 |
|  |  |  |  |  |

## [*^i^*Pr_2_Si(OTeF_5_)_2_F]^–^

| Figure S 127: Representation of the B3LYP/def2-TZVPP structure of [^i^Pr_2_Si(OTeF_5_)_2_F]^–^. | Si | 0.077303 | -0.005182 | 0.051348 |
| --- | --- | --- | --- | --- |
|  | O | 1.264004 | -0.525780 | -1.366992 |
|  | Te | 2.879094 | -1.326725 | -1.627087 |
|  | F | 3.173331 | -0.393843 | -3.231732 |
|  | F | 2.136061 | -2.738995 | -2.623532 |
|  | F | 4.528852 | -2.151882 | -1.983670 |
|  | F | 3.893290 | -0.041174 | -0.713446 |
|  | F | 2.840565 | -2.416041 | -0.098969 |
|  | O | -1.047391 | 0.509363 | 1.521215 |
|  | Te | -0.961871 | 1.206211 | 3.200869 |
|  | F | 0.073345 | -0.168708 | 3.944609 |
|  | F | -2.518019 | 0.299834 | 3.737933 |
|  | F | -0.969078 | 1.929126 | 4.935133 |
|  | F | 0.575159 | 2.251641 | 2.935114 |
|  | F | -2.022506 | 2.692790 | 2.750571 |
|  | C | -0.285081 | 1.632615 | -0.861592 |
|  | C | -0.616647 | 1.489203 | -2.355110 |
|  | C | 0.857659 | 2.642555 | -0.657736 |
|  | H | -1.171839 | 2.030901 | -0.364200 |
|  | H | -1.433221 | 0.786491 | -2.534192 |
|  | H | -0.924738 | 2.457717 | -2.763071 |
|  | H | 0.245196 | 1.143161 | -2.924016 |
|  | H | 1.064732 | 2.822445 | 0.397294 |
|  | H | 1.780639 | 2.293073 | -1.123183 |
|  | H | 0.598371 | 3.602936 | -1.115568 |
|  | C | -0.990789 | -1.569053 | -0.194669 |
|  | C | -2.503759 | -1.309426 | -0.274486 |
|  | C | -0.681204 | -2.630823 | 0.874664 |
|  | H | -0.674740 | -1.968423 | -1.160545 |
|  | H | -2.758651 | -0.570015 | -1.036834 |
|  | H | -3.028269 | -2.235942 | -0.531218 |
|  | H | -2.897894 | -0.952283 | 0.675838 |
|  | H | 0.378344 | -2.885622 | 0.906496 |
|  | H | -0.971324 | -2.286286 | 1.868346 |
|  | H | -1.239760 | -3.549483 | 0.666982 |
|  | F | 1.330836 | -0.064374 | 1.075436 |
|  |  |  |  |  |

## Ph_2_Si(OTeF_5_)_2_

| Figure S 128: Representation of the B3LYP/def2-TZVPP structure of Ph_2_Si(OTeF_5_)_2_. | Si | 0.136431 | 0.243497 | 0.052433 |
| --- | --- | --- | --- | --- |
|  | C | 0.980353 | -1.211340 | -0.723306 |
|  | C | 0.329682 | -1.913155 | -1.748758 |
|  | C | 2.258332 | -1.632409 | -0.332369 |
|  | C | 0.939198 | -2.997195 | -2.367138 |
|  | H | -0.661861 | -1.615606 | -2.068260 |
|  | C | 2.866531 | -2.719024 | -0.948959 |
|  | H | 2.780921 | -1.109613 | 0.457100 |
|  | C | 2.208576 | -3.401042 | -1.966609 |
|  | H | 0.424624 | -3.526189 | -3.158441 |
|  | H | 3.853706 | -3.032150 | -0.635391 |
|  | H | 2.683689 | -4.246702 | -2.446679 |
|  | C | -1.652520 | 0.001004 | 0.466859 |
|  | C | -2.052328 | -1.181846 | 1.106036 |
|  | C | -2.626452 | 0.965936 | 0.177689 |
|  | C | -3.381661 | -1.390633 | 1.450083 |
|  | H | -1.323450 | -1.949436 | 1.337021 |
|  | C | -3.956851 | 0.755365 | 0.518969 |
|  | H | -2.344705 | 1.886170 | -0.315703 |
|  | C | -4.335088 | -0.421478 | 1.155847 |
|  | H | -3.673038 | -2.307438 | 1.945414 |
|  | H | -4.697115 | 1.510275 | 0.288715 |
|  | H | -5.371443 | -0.583859 | 1.422214 |
|  | O | 0.148740 | 1.604577 | -0.928706 |
|  | Te | 1.301058 | 2.577793 | -2.030600 |
|  | F | 1.243562 | 1.278285 | -3.357389 |
|  | F | 2.820383 | 1.778025 | -1.313078 |
|  | F | 2.405204 | 3.573652 | -3.137591 |
|  | F | -0.160036 | 3.418703 | -2.800933 |
|  | F | 1.421850 | 3.939459 | -0.778544 |
|  | O | 1.075925 | 0.590421 | 1.398754 |
|  | Te | 1.056394 | 1.660991 | 2.929392 |
|  | F | 2.272206 | 2.841396 | 2.177580 |
|  | F | 2.456349 | 0.668791 | 3.630011 |
|  | F | 1.078493 | 2.700444 | 4.464087 |
|  | F | -0.349575 | 2.705428 | 2.300914 |
|  | F | -0.156087 | 0.538905 | 3.779335 |
|  |  |  |  |  |

## [Ph_2_Si(OTeF_5_)_2_F]^–^

| Figure S 129: Representation of the B3LYP/def2-TZVPP structure of [Ph_2_Si(OTeF_5_)_2_F]^–^. | Si | -0.051893 | -0.405860 | 0.431387 |
| --- | --- | --- | --- | --- |
|  | C | 0.483239 | -0.935662 | -1.300813 |
|  | C | 1.464822 | -1.913747 | -1.501627 |
|  | C | -0.104353 | -0.360301 | -2.433851 |
|  | C | 1.834320 | -2.311869 | -2.781298 |
|  | H | 1.947907 | -2.370064 | -0.648258 |
|  | C | 0.279321 | -0.737029 | -3.716715 |
|  | H | -0.881479 | 0.383620 | -2.315407 |
|  | C | 1.247512 | -1.718673 | -3.894131 |
|  | H | 2.586668 | -3.080667 | -2.908686 |
|  | H | -0.188595 | -0.272705 | -4.576206 |
|  | H | 1.540250 | -2.022088 | -4.892015 |
|  | C | -0.431484 | 1.403518 | 0.817668 |
|  | C | 0.340006 | 2.427273 | 0.254662 |
|  | C | -1.475380 | 1.770777 | 1.675317 |
|  | C | 0.074042 | 3.765187 | 0.526785 |
|  | H | 1.168219 | 2.178427 | -0.396235 |
|  | C | -1.729640 | 3.105329 | 1.970606 |
|  | H | -2.098489 | 1.005445 | 2.118014 |
|  | C | -0.959818 | 4.108102 | 1.390591 |
|  | H | 0.683526 | 4.537738 | 0.074002 |
|  | H | -2.534604 | 3.361919 | 2.648473 |
|  | H | -1.162115 | 5.148812 | 1.613181 |
|  | O | 1.775612 | -0.190196 | 0.840128 |
|  | Te | 2.837936 | -0.193422 | 2.329885 |
|  | F | 3.181662 | 1.650296 | 2.242460 |
|  | F | 4.378914 | -0.492505 | 1.301808 |
|  | F | 3.998150 | -0.183995 | 3.805913 |
|  | F | 1.475250 | 0.125426 | 3.578446 |
|  | F | 2.713330 | -2.040036 | 2.632050 |
|  | F | -0.185432 | -1.514708 | 1.597496 |
|  | O | -1.880024 | -0.614657 | 0.019479 |
|  | Te | -3.096430 | -1.980121 | -0.057965 |
|  | F | -3.207902 | -1.941329 | -1.931754 |
|  | F | -4.537476 | -0.780778 | 0.015730 |
|  | F | -4.405563 | -3.322079 | -0.162486 |
|  | F | -1.854338 | -3.381088 | -0.165515 |
|  | F | -3.225671 | -2.208290 | 1.798879 |
|  |  |  |  |  |

## MeSi(OTeF_5_)_3_

| Figure S 130: Representation of the B3LYP/def2-TZVPP structure of MeSi(OTeF_5_)_3_. | Si | 0.002612 | 0.644002 | -0.165046 |
| --- | --- | --- | --- | --- |
|  | C | 0.538637 | 1.991655 | -1.289151 |
|  | H | -0.120060 | 2.853548 | -1.178053 |
|  | H | 1.555911 | 2.305132 | -1.053880 |
|  | H | 0.509937 | 1.661221 | -2.327724 |
|  | O | 0.094383 | 1.202590 | 1.382742 |
|  | Te | -0.376274 | 0.720930 | 3.137131 |
|  | F | 0.032074 | 2.437379 | 3.692059 |
|  | F | 1.364583 | 0.194194 | 3.488425 |
|  | F | -0.817683 | 0.288205 | 4.880070 |
|  | F | -2.137381 | 1.229024 | 2.859044 |
|  | F | -0.799838 | -1.015424 | 2.631192 |
|  | O | -1.572643 | 0.182767 | -0.371686 |
|  | Te | -2.613112 | -0.739957 | -1.631661 |
|  | F | -4.005195 | -0.828005 | -0.421542 |
|  | F | -1.984430 | -2.379918 | -1.047416 |
|  | F | -3.657689 | -1.628511 | -2.873591 |
|  | F | -3.278319 | 0.861961 | -2.285069 |
|  | F | -1.243098 | -0.662935 | -2.892574 |
|  | O | 0.876095 | -0.754326 | -0.306327 |
|  | Te | 2.628075 | -1.329427 | -0.642487 |
|  | F | 1.979565 | -2.905681 | -1.351574 |
|  | F | 2.725794 | -2.110367 | 1.029972 |
|  | F | 4.351636 | -1.911147 | -0.978195 |
|  | F | 2.610032 | -0.554605 | -2.331494 |
|  | F | 3.336389 | 0.247694 | 0.046835 |
|  |  |  |  |  |

## [MeSi(OTeF_5_)_3_F]^–^

| Figure S 131: Representation of the B3LYP/def2-TZVPP structure of [MeSi(OTeF_5_)_3_F]^–^. | Si | 0.168141 | -0.573693 | -0.317787 |
| --- | --- | --- | --- | --- |
|  | O | 0.124338 | 1.133228 | -0.273706 |
|  | Te | 0.608181 | 2.618267 | 0.723810 |
|  | F | -1.002381 | 3.445875 | 0.292430 |
|  | F | 1.385792 | 3.401867 | -0.774685 |
|  | F | 1.046837 | 4.186149 | 1.642647 |
|  | F | -0.153739 | 2.029987 | 2.317424 |
|  | F | 2.284264 | 1.985071 | 1.226366 |
|  | C | -1.360148 | -1.550763 | 0.120620 |
|  | H | -1.447418 | -2.400829 | -0.556775 |
|  | H | -1.364716 | -1.908145 | 1.146667 |
|  | H | -2.241841 | -0.926821 | -0.031609 |
|  | O | -0.306413 | -0.453023 | -2.102702 |
|  | Te | -1.568079 | 0.313202 | -3.178078 |
|  | F | -0.670587 | -0.240725 | -4.720914 |
|  | F | -0.720320 | 1.975166 | -3.287638 |
|  | F | -2.844318 | 1.041374 | -4.341811 |
|  | F | -2.611126 | -1.244795 | -3.248914 |
|  | F | -2.663038 | 0.953062 | -1.793713 |
|  | F | 1.508000 | -1.285118 | -0.847545 |
|  | O | 0.847042 | -0.534560 | 1.394910 |
|  | Te | 1.662948 | -1.736760 | 2.510362 |
|  | F | 2.137040 | -0.397666 | 3.720061 |
|  | F | 3.346013 | -1.608727 | 1.711009 |
|  | F | 2.471296 | -2.932177 | 3.706045 |
|  | F | 0.094595 | -2.042085 | 3.492769 |
|  | F | 1.269638 | -3.247362 | 1.470760 |
|  |  |  |  |  |

## EtSi(OTeF_5_)_3_

| Figure S 132: Representation of the B3LYP/def2-TZVPP structure of EtSi(OTeF_5_)_3_. | Si | -0.144364 | 0.227611 | -0.194999 |
| --- | --- | --- | --- | --- |
|  | C | -0.104332 | 1.510707 | -1.518585 |
|  | C | -0.845112 | 2.813695 | -1.170443 |
|  | H | -0.522739 | 1.064327 | -2.424617 |
|  | H | 0.947893 | 1.712319 | -1.739774 |
|  | H | -0.769069 | 3.521414 | -1.995767 |
|  | H | -1.903838 | 2.634495 | -0.983851 |
|  | H | -0.424551 | 3.291550 | -0.285715 |
|  | O | 0.722451 | -1.138941 | -0.540557 |
|  | Te | 1.176967 | -2.270022 | -1.963241 |
|  | F | 2.736976 | -2.741981 | -1.094818 |
|  | F | 2.070752 | -0.895565 | -2.835863 |
|  | F | 1.639389 | -3.393813 | -3.358522 |
|  | F | 0.292576 | -3.682059 | -1.163105 |
|  | F | -0.371325 | -1.819739 | -2.892996 |
|  | O | 0.451968 | 0.755656 | 1.257064 |
|  | Te | 2.116386 | 0.989732 | 2.089272 |
|  | F | 2.363079 | -0.835324 | 2.275959 |
|  | F | 1.287703 | 1.059102 | 3.738733 |
|  | F | 3.749416 | 1.251783 | 2.917652 |
|  | F | 3.000613 | 0.945719 | 0.449936 |
|  | F | 1.952412 | 2.829789 | 1.930845 |
|  | O | -1.717695 | -0.177012 | 0.097370 |
|  | Te | -2.761333 | -1.112227 | 1.350903 |
|  | F | -2.809579 | -2.600582 | 0.248991 |
|  | F | -4.288563 | -0.456377 | 0.537703 |
|  | F | -3.827084 | -2.021891 | 2.559308 |
|  | F | -1.261425 | -1.801519 | 2.204778 |
|  | F | -2.757572 | 0.339152 | 2.504336 |
|  |  |  |  |  |

## [EtSi(OTeF_5_)_3_F]^–^

| Figure S 133: Representation of the B3LYP/def2-TZVPP structure of [EtSi(OTeF_5_)_3_F]^–^. | Si | 0.250630 | -0.016356 | -0.104696 |
| --- | --- | --- | --- | --- |
|  | C | 0.507792 | 0.778598 | -1.794408 |
|  | C | 1.951224 | 1.232754 | -2.070115 |
|  | H | 0.214024 | 0.037378 | -2.540383 |
|  | H | -0.175556 | 1.622055 | -1.891127 |
|  | F | 1.287408 | 0.114083 | 1.120496 |
|  | O | -0.728846 | 1.397239 | 0.564048 |
|  | Te | -0.412859 | 3.012538 | 1.364011 |
|  | F | -2.190535 | 3.169808 | 1.910676 |
|  | F | -0.833034 | 3.971204 | -0.192649 |
|  | F | -0.155128 | 4.691376 | 2.155436 |
|  | F | 0.042870 | 2.305587 | 3.031371 |
|  | F | 1.399869 | 3.107332 | 0.892443 |
|  | O | 1.136116 | -1.492877 | -0.725925 |
|  | Te | 1.770389 | -3.078593 | -0.052385 |
|  | F | 2.649714 | -3.451232 | -1.664104 |
|  | F | 0.299561 | -4.067307 | -0.655252 |
|  | F | 2.448136 | -4.718501 | 0.549699 |
|  | F | 3.357478 | -2.339571 | 0.610745 |
|  | F | 0.981378 | -2.925913 | 1.636744 |
|  | O | -1.112270 | -1.020389 | 0.134197 |
|  | Te | -2.901662 | -1.028554 | 0.609042 |
|  | F | -2.664333 | -2.598036 | 1.578704 |
|  | F | -3.309578 | -2.051398 | -0.894393 |
|  | F | -4.714891 | -1.151418 | 1.046847 |
|  | F | -2.701570 | -0.050618 | 2.178103 |
|  | F | -3.352443 | 0.499296 | -0.357250 |
|  | H | 2.030926 | 1.667161 | -3.069664 |
|  | H | 2.646685 | 0.395606 | -2.014237 |
|  | H | 2.278505 | 1.988751 | -1.355974 |
|  |  |  |  |  |

## *^t^*BuSi(OTeF_5_)_3_

| Figure S 134: Representation of the B3LYP/def2-TZVPP structure of ^t^BuSi(OTeF_5_)_3_. | Si | 0.021613 | -0.070582 | 0.016019 |
| --- | --- | --- | --- | --- |
|  | C | 0.910891 | -1.648405 | 0.481479 |
|  | C | 0.842255 | -2.635708 | -0.701613 |
|  | C | 2.383832 | -1.325557 | 0.806732 |
|  | C | 0.228994 | -2.268652 | 1.718353 |
|  | H | -0.183738 | -2.912485 | -0.947152 |
|  | H | 1.312020 | -2.234501 | -1.600306 |
|  | H | 1.373105 | -3.552632 | -0.432303 |
|  | H | 2.478303 | -0.625956 | 1.637916 |
|  | H | 2.895951 | -2.248381 | 1.091623 |
|  | H | 2.913970 | -0.909407 | -0.050850 |
|  | H | 0.742883 | -3.196985 | 1.981512 |
|  | H | 0.276860 | -1.611523 | 2.587421 |
|  | H | -0.817532 | -2.512404 | 1.532216 |
|  | O | 0.133435 | 1.047012 | 1.229011 |
|  | Te | -0.856513 | 2.075497 | 2.444566 |
|  | F | 0.344155 | 1.637346 | 3.785948 |
|  | F | -1.900078 | 0.613371 | 2.916315 |
|  | F | -1.802557 | 3.089971 | 3.669790 |
|  | F | 0.152717 | 3.568532 | 2.034272 |
|  | F | -2.100679 | 2.544025 | 1.150872 |
|  | O | -1.579033 | -0.364925 | -0.277324 |
|  | Te | -2.915036 | -0.213521 | -1.585696 |
|  | F | -4.112921 | 0.397615 | -0.316761 |
|  | F | -3.443983 | -1.955120 | -1.238003 |
|  | F | -4.254198 | -0.091924 | -2.857120 |
|  | F | -2.428969 | 1.530666 | -1.988941 |
|  | F | -1.773589 | -0.838777 | -2.911119 |
|  | O | 0.662273 | 0.598215 | -1.353795 |
|  | Te | 1.580379 | 2.092760 | -2.015782 |
|  | F | 0.522665 | 2.091679 | -3.531574 |
|  | F | 2.788883 | 0.972375 | -2.863292 |
|  | F | 2.502067 | 3.544293 | -2.700464 |
|  | F | 0.413477 | 3.271905 | -1.186579 |
|  | F | 2.688097 | 2.142179 | -0.525373 |
|  |  |  |  |  |

## [*^t^*BuSi(OTeF_5_)_3_F]^–^

| Figure S 135: Representation of the B3LYP/def2-TZVPP structure of [^t^BuSi(OTeF_5_)_3_F]^–^. | Si | 0.193366 | -0.006744 | 0.330558 |
| --- | --- | --- | --- | --- |
|  | C | 0.927517 | 0.516791 | -1.380551 |
|  | C | 2.341317 | 1.083029 | -1.121738 |
|  | C | 1.032293 | -0.699862 | -2.325212 |
|  | C | 0.104324 | 1.592386 | -2.113516 |
|  | F | 0.779303 | 0.529206 | 1.737416 |
|  | O | -1.092767 | 1.300812 | 0.402825 |
|  | Te | -1.444208 | 2.815636 | 1.382699 |
|  | F | -3.289129 | 2.563794 | 1.255797 |
|  | F | -1.532660 | 3.885203 | -0.154239 |
|  | F | -1.857124 | 4.382271 | 2.326597 |
|  | F | -1.433607 | 1.978159 | 3.050758 |
|  | F | 0.344310 | 3.334733 | 1.582257 |
|  | O | -1.030778 | -1.208796 | 0.325122 |
|  | Te | -2.843670 | -1.529566 | 0.164452 |
|  | F | -2.731467 | -2.935361 | 1.376581 |
|  | F | -2.532964 | -2.737056 | -1.222503 |
|  | F | -4.654099 | -1.958727 | -0.013445 |
|  | F | -3.354169 | -0.395500 | 1.546267 |
|  | F | -3.148466 | -0.203969 | -1.110896 |
|  | O | 1.391174 | -1.393087 | 0.409380 |
|  | Te | 1.991135 | -2.596210 | 1.657305 |
|  | F | 3.404570 | -3.100766 | 0.535030 |
|  | F | 0.974153 | -4.005682 | 0.962350 |
|  | F | 2.667493 | -3.861335 | 2.862663 |
|  | F | 3.135205 | -1.383312 | 2.507478 |
|  | F | 0.664722 | -2.270078 | 2.934576 |
|  | H | 2.785663 | 1.391650 | -2.074094 |
|  | H | 2.999790 | 0.344513 | -0.666596 |
|  | H | 2.314704 | 1.960367 | -0.472554 |
|  | H | 1.441424 | -0.367536 | -3.285120 |
|  | H | 0.054872 | -1.143545 | -2.524117 |
|  | H | 1.680001 | -1.477957 | -1.930957 |
|  | H | 0.569099 | 1.781889 | -3.087370 |
|  | H | 0.072585 | 2.536767 | -1.576218 |
|  | H | -0.923912 | 1.277884 | -2.290986 |
|  |  |  |  |  |

## PhSi(OTeF_5_)_3_

| Figure S 136: Representation of the B3LYP/def2-TZVPP structure of PhSi(OTeF_5_)_3_. | Si | 0.346541 | -0.090840 | 0.066734 |
| --- | --- | --- | --- | --- |
|  | C | -1.388365 | -0.146817 | 0.660438 |
|  | C | -2.432339 | -0.363704 | -0.251751 |
|  | C | -1.697585 | 0.003844 | 2.018858 |
|  | C | -3.748053 | -0.428395 | 0.185903 |
|  | H | -2.224345 | -0.479840 | -1.307927 |
|  | C | -3.015859 | -0.059865 | 2.452562 |
|  | H | -0.909153 | 0.175710 | 2.739028 |
|  | C | -4.039995 | -0.276037 | 1.537575 |
|  | H | -4.544187 | -0.594963 | -0.527325 |
|  | H | -3.242250 | 0.060783 | 3.503525 |
|  | H | -5.066432 | -0.324492 | 1.876678 |
|  | O | 1.312719 | 0.340922 | 1.332504 |
|  | Te | 3.110670 | 0.224963 | 1.869844 |
|  | F | 2.584455 | 0.481900 | 3.625775 |
|  | F | 3.313105 | 2.053145 | 1.667958 |
|  | F | 4.869689 | 0.130934 | 2.435731 |
|  | F | 2.979879 | -1.609268 | 2.104002 |
|  | F | 3.691984 | -0.036291 | 0.125006 |
|  | O | 0.905611 | -1.550151 | -0.465864 |
|  | Te | 1.133754 | -2.585479 | -2.010498 |
|  | F | 2.530023 | -3.486165 | -1.202265 |
|  | F | 2.325841 | -1.372562 | -2.742804 |
|  | F | 1.350980 | -3.641758 | -3.515024 |
|  | F | -0.057779 | -3.842734 | -1.355942 |
|  | F | -0.273247 | -1.722690 | -2.871365 |
|  | O | 0.591293 | 0.927165 | -1.215457 |
|  | Te | 0.315281 | 2.718605 | -1.700982 |
|  | F | 0.797011 | 2.316083 | -3.437941 |
|  | F | 2.073043 | 3.136271 | -1.301297 |
|  | F | 0.052980 | 4.480383 | -2.204282 |
|  | F | -1.459813 | 2.383323 | -2.121243 |
|  | F | -0.185458 | 3.178018 | 0.029845 |
|  |  |  |  |  |

## [PhSi(OTeF_5_)_3_F]^–^

| Figure S 137: Representation of the B3LYP/def2-TZVPP structure of [PhSi(OTeF_5_)_3_F]^–^. | Si | 0.023617 | -0.287809 | 0.533842 |
| --- | --- | --- | --- | --- |
|  | C | 1.024235 | 0.507658 | -0.846578 |
|  | C | 0.394908 | 1.187293 | -1.896396 |
|  | C | 2.423554 | 0.453131 | -0.845425 |
|  | F | 0.464994 | -0.197650 | 2.077834 |
|  | O | -1.067083 | 1.166427 | 0.725755 |
|  | Te | -0.996172 | 2.776997 | 1.604224 |
|  | F | -2.844071 | 2.997022 | 1.468871 |
|  | F | -0.800652 | 3.748950 | 0.016369 |
|  | F | -0.981809 | 4.443153 | 2.461724 |
|  | F | -1.212483 | 2.050951 | 3.309464 |
|  | F | 0.862065 | 2.789192 | 1.826336 |
|  | O | -1.357387 | -1.219240 | 0.153191 |
|  | Te | -3.205470 | -1.185884 | 0.025635 |
|  | F | -3.334109 | -2.758839 | 1.008632 |
|  | F | -3.123321 | -2.196708 | -1.536279 |
|  | F | -5.063960 | -1.261829 | -0.155142 |
|  | F | -3.503983 | -0.211408 | 1.581255 |
|  | F | -3.276691 | 0.356128 | -1.019859 |
|  | O | 1.004572 | -1.803978 | 0.307610 |
|  | Te | 1.192271 | -3.420355 | 1.165851 |
|  | F | 2.584775 | -3.905046 | 0.013231 |
|  | F | -0.015470 | -4.305212 | 0.044097 |
|  | F | 1.453339 | -5.089056 | 1.976170 |
|  | F | 2.461808 | -2.781945 | 2.385758 |
|  | F | -0.163554 | -3.158355 | 2.428303 |
|  | C | 3.166931 | 1.070664 | -1.844435 |
|  | C | 2.523960 | 1.731657 | -2.885193 |
|  | C | 1.135051 | 1.782756 | -2.911866 |
|  | H | 0.624709 | 2.298084 | -3.716010 |
|  | H | 3.101765 | 2.206326 | -3.668795 |
|  | H | -0.683740 | 1.265566 | -1.919197 |
|  | H | 2.938790 | -0.076488 | -0.055919 |
|  | H | 4.248611 | 1.027849 | -1.813059 |
|  |  |  |  |  |

## Si(OTeF_5_)_4_

| Figure S 138: Representation of the B3LYP/def2-TZVPP structure of Si(OTeF_5_)_4_. | Si | 0.000000 | 0.000000 | 0.000000 |
| --- | --- | --- | --- | --- |
|  | O | -1.148713 | 0.716733 | 0.906144 |
|  | Te | -2.629098 | 0.263643 | 1.985542 |
|  | F | -4.078992 | -0.150412 | 3.052808 |
|  | F | -1.643700 | 0.626620 | 3.507527 |
|  | F | -3.654179 | -0.120319 | 0.493941 |
|  | F | -2.089266 | -1.511481 | 2.064031 |
|  | F | -3.183849 | 2.022210 | 1.924063 |
|  | O | 1.148713 | -0.716733 | 0.906144 |
|  | Te | 2.629098 | -0.263643 | 1.985542 |
|  | F | 4.078992 | 0.150412 | 3.052808 |
|  | F | 2.089266 | 1.511481 | 2.064031 |
|  | F | 3.183849 | -2.022210 | 1.924063 |
|  | F | 3.654179 | 0.120319 | 0.493941 |
|  | F | 1.643700 | -0.626620 | 3.507527 |
|  | O | 0.716733 | 1.148713 | -0.906144 |
|  | Te | 0.263643 | 2.629098 | -1.985542 |
|  | F | -0.150412 | 4.078992 | -3.052808 |
|  | F | -0.120319 | 3.654179 | -0.493941 |
|  | F | 0.626620 | 1.643700 | -3.507527 |
|  | F | -1.511481 | 2.089266 | -2.064031 |
|  | F | 2.022210 | 3.183849 | -1.924063 |
|  | O | -0.716733 | -1.148713 | -0.906144 |
|  | Te | -0.263643 | -2.629098 | -1.985542 |
|  | F | 0.150412 | -4.078992 | -3.052808 |
|  | F | -2.022210 | -3.183849 | -1.924063 |
|  | F | 1.511481 | -2.089266 | -2.064031 |
|  | F | 0.120319 | -3.654179 | -0.493941 |
|  | F | -0.626620 | -1.643700 | -3.507527 |
|  |  |  |  |  |

## [Si(OTeF_5_)_4_F]^–^

| Figure S 139: Representation of the B3LYP/def2-TZVPP structure of [Si(OTeF_5_)_4_F]^–^. | Si | 0.303183 | 0.087901 | -0.376961 |
| --- | --- | --- | --- | --- |
|  | F | 0.656343 | 0.260223 | -1.966504 |
|  | O | 1.714571 | 0.955846 | 0.002997 |
|  | Te | 2.622624 | 1.782857 | 1.391122 |
|  | F | 2.776991 | 3.337538 | 0.379797 |
|  | F | 4.242855 | 1.196089 | 0.689094 |
|  | F | 3.617445 | 2.660176 | 2.704969 |
|  | F | 1.106978 | 2.491078 | 2.204241 |
|  | F | 2.614268 | 0.309267 | 2.524303 |
|  | O | -1.178268 | 0.922672 | -0.508413 |
|  | Te | -2.085203 | 1.923945 | -1.788872 |
|  | F | -2.357352 | 3.282827 | -0.547001 |
|  | F | -3.696314 | 1.157042 | -1.264288 |
|  | F | -3.078119 | 2.957026 | -2.986732 |
|  | F | -0.580914 | 2.826811 | -2.410845 |
|  | F | -1.942335 | 0.662811 | -3.147497 |
|  | O | -0.027941 | -0.084559 | 1.355872 |
|  | Te | -1.338547 | -0.820144 | 2.429424 |
|  | F | -0.451810 | -0.213989 | 3.950231 |
|  | F | -0.456856 | -2.455603 | 2.584385 |
|  | F | -2.618599 | -1.537473 | 3.587797 |
|  | F | -2.375624 | 0.728348 | 2.437118 |
|  | F | -2.367890 | -1.504551 | 1.030343 |
|  | O | 0.359128 | -1.617913 | -0.454572 |
|  | Te | 0.692300 | -2.928708 | -1.734559 |
|  | F | -0.739330 | -3.932507 | -1.099538 |
|  | F | 1.811982 | -3.821360 | -0.544762 |
|  | F | 1.010594 | -4.326481 | -2.932072 |
|  | F | -0.419676 | -2.200761 | -3.035429 |
|  | F | 2.185517 | -2.098407 | -2.473647 |
|  |  |  |  |  |

## Me_2_Si(SO_3_CF_3_)_2_

| Figure S 140: Representation of the B3LYP/def2-TZVPP structure of Me_2_Si(SO_3_CF_3_)_2._ | Si | 0.369561 | 0.916236 | 0.299263 |
| --- | --- | --- | --- | --- |
|  | C | 2.202081 | 0.894971 | 0.493896 |
|  | O | -0.109199 | -0.150926 | -0.941998 |
|  | C | -0.445071 | 2.553532 | 0.073631 |
|  | O | -0.219174 | 0.113015 | 1.668910 |
|  | S | -1.655925 | -0.132410 | 2.259242 |
|  | S | 0.062749 | -0.047646 | -2.494118 |
|  | O | -1.195642 | -0.224357 | -3.131488 |
|  | O | 0.926841 | 1.050065 | -2.808625 |
|  | C | 1.040082 | -1.616254 | -2.767971 |
|  | O | -2.653127 | 0.251643 | 1.311574 |
|  | O | -1.681422 | 0.316100 | 3.609813 |
|  | C | -1.603903 | -2.000409 | 2.304684 |
|  | F | -0.607538 | -2.410268 | 3.085423 |
|  | F | -1.430445 | -2.488252 | 1.079156 |
|  | F | 0.318349 | -2.677433 | -2.427784 |
|  | F | 2.156852 | -1.593615 | -2.036655 |
|  | F | 1.360942 | -1.688808 | -4.055494 |
|  | F | -2.759722 | -2.436079 | 2.794702 |
|  | H | 2.682768 | 1.301576 | -0.396845 |
|  | H | 2.568988 | -0.119758 | 0.651443 |
|  | H | 2.498306 | 1.501644 | 1.351367 |
|  | H | -0.054361 | 3.043738 | -0.819635 |
|  | H | -0.247714 | 3.200212 | 0.930652 |
|  | H | -1.524278 | 2.443481 | -0.033141 |
|  |  |  |  |  |

## [Me_2_Si(SO_3_CF_3_)_2_F]^–^

| Figure S 141: Representation of the B3LYP/def2-TZVPP structure of [Me_2_Si(SO_3_CF_3_)_2_F]^–^. | Si | 0.384592 | 0.321510 | 0.056773 |
| --- | --- | --- | --- | --- |
|  | C | -0.683410 | -1.183250 | -0.198105 |
|  | H | -0.189192 | -1.853842 | -0.903363 |
|  | H | -0.853824 | -1.730646 | 0.725730 |
|  | H | -1.638404 | -0.889022 | -0.632653 |
|  | C | -0.248359 | 1.948278 | 0.720030 |
|  | H | -0.200073 | 1.994339 | 1.805403 |
|  | H | 0.348866 | 2.761473 | 0.304066 |
|  | H | -1.278565 | 2.107569 | 0.398813 |
|  | F | 1.912073 | 0.252194 | -0.441281 |
|  | O | 0.963550 | -0.178008 | 1.834989 |
|  | S | 1.862028 | -1.246655 | 2.395383 |
|  | O | 3.100344 | -0.725469 | 2.899863 |
|  | O | 1.875499 | -2.452131 | 1.612814 |
|  | C | 0.882793 | -1.677257 | 3.923264 |
|  | F | -0.348841 | -2.108987 | 3.611164 |
|  | F | 1.503497 | -2.654273 | 4.601395 |
|  | F | 0.755894 | -0.622117 | 4.740833 |
|  | O | 0.013328 | 0.931025 | -1.818949 |
|  | S | -1.121188 | 1.509847 | -2.595816 |
|  | O | -0.901119 | 2.878156 | -2.979463 |
|  | O | -2.419500 | 1.146049 | -2.083062 |
|  | C | -0.955341 | 0.549696 | -4.185360 |
|  | F | -1.116000 | -0.766118 | -3.981521 |
|  | F | -1.892743 | 0.949334 | -5.060363 |
|  | F | 0.244095 | 0.738306 | -4.750583 |
|  | O | 1.875499 | -2.452131 | 1.612814 |
|  |  |  |  |  |

## Ph_2_Si(SO_3_CF_3_)_2_

| Figure S 142: Representation of the B3LYP/def2-TZVPP structure of Ph_2_Si(SO_3_CF_3_)_2_. | Si | -0.186629 | -0.030834 | 0.166529 |
| --- | --- | --- | --- | --- |
|  | C | 1.640815 | -0.044741 | 0.433908 |
|  | O | -0.683858 | -1.072003 | -1.082694 |
|  | C | -0.973840 | 1.619043 | -0.071153 |
|  | O | -0.800014 | -0.870115 | 1.504927 |
|  | C | 2.481287 | 0.659734 | -0.441950 |
|  | C | -0.376832 | 2.756417 | 0.492909 |
|  | S | -2.232063 | -1.223464 | 2.042300 |
|  | S | -0.492224 | -1.124090 | -2.633883 |
|  | O | -1.765469 | -1.209184 | -3.264579 |
|  | O | 0.493668 | -0.174492 | -3.046250 |
|  | C | 0.286154 | -2.818628 | -2.742452 |
|  | O | -3.226173 | -0.915122 | 1.065591 |
|  | O | -2.334293 | -0.792764 | 3.395132 |
|  | C | -2.048820 | -3.082945 | 2.078445 |
|  | F | -1.023591 | -3.431336 | 2.854271 |
|  | F | -1.846497 | -3.554130 | 0.850670 |
|  | F | -0.551751 | -3.741587 | -2.281850 |
|  | F | 1.411354 | -2.852129 | -2.030150 |
|  | F | 0.564999 | -3.069868 | -4.018475 |
|  | F | -3.170698 | -3.599300 | 2.570614 |
|  | C | 3.857848 | 0.659134 | -0.257729 |
|  | C | 4.417979 | -0.041032 | 0.805711 |
|  | C | 3.599724 | -0.742240 | 1.684231 |
|  | C | 2.222432 | -0.745234 | 1.500437 |
|  | H | 2.063117 | 1.202572 | -1.279644 |
|  | H | 4.492267 | 1.203530 | -0.944597 |
|  | H | 1.598509 | -1.291601 | 2.194535 |
|  | C | -0.962300 | 4.008422 | 0.354325 |
|  | C | -2.151776 | 4.142934 | -0.353226 |
|  | C | -2.756741 | 3.025426 | -0.919200 |
|  | C | -2.175543 | 1.772442 | -0.778348 |
|  | H | -2.666695 | 0.912741 | -1.214493 |
|  | H | -2.607442 | 5.118339 | -0.463933 |
|  | H | -3.683042 | 3.128578 | -1.468334 |
|  | H | 0.552930 | 2.667463 | 1.040763 |
|  | H | -0.490067 | 4.876411 | 0.795134 |
|  | H | 5.490741 | -0.039980 | 0.949181 |
|  | H | 4.032537 | -1.286366 | 2.513329 |
|  |  |  |  |  |

## [Ph_2_Si(SO_3_CF_3_)_2_F]^–^

| Figure S 143: Representation of the B3LYP/def2-TZVPP structure of [Ph_2_Si(SO_3_CF_3_)_2_F]^–^. | Si | 0.000014 | -0.000187 | 0.735372 |
| --- | --- | --- | --- | --- |
|  | C | 0.276524 | 1.622030 | -0.176470 |
|  | C | 1.251597 | 2.533598 | 0.246247 |
|  | C | -0.500733 | 1.965096 | -1.288822 |
|  | C | 1.431301 | 3.747234 | -0.408465 |
|  | H | 1.879213 | 2.296942 | 1.095493 |
|  | C | -0.305173 | 3.165221 | -1.963721 |
|  | H | -1.276823 | 1.292279 | -1.630135 |
|  | C | 0.658596 | 4.063862 | -1.520238 |
|  | H | 2.181763 | 4.442218 | -0.052198 |
|  | H | -0.914843 | 3.402631 | -2.826997 |
|  | H | 0.804853 | 5.005096 | -2.036278 |
|  | C | -0.276538 | -1.622327 | -0.176607 |
|  | C | -1.251709 | -2.533867 | 0.245953 |
|  | C | 0.500764 | -1.965351 | -1.288946 |
|  | C | -1.431584 | -3.747349 | -0.409001 |
|  | H | -1.879217 | -2.297348 | 1.095315 |
|  | C | 0.305058 | -3.165329 | -1.964062 |
|  | H | 1.277041 | -1.292645 | -1.630040 |
|  | C | -0.658855 | -4.063911 | -1.520775 |
|  | H | -2.182235 | -4.442243 | -0.052954 |
|  | H | 0.914713 | -3.402664 | -2.827369 |
|  | H | -0.805188 | -5.005057 | -2.036953 |
|  | F | 0.000044 | -0.000282 | 2.344049 |
|  | O | 1.893549 | -0.300714 | 0.751738 |
|  | S | 2.950728 | -0.640185 | 1.769411 |
|  | O | 2.748634 | -1.906572 | 2.412629 |
|  | O | 3.344391 | 0.494156 | 2.559576 |
|  | C | 4.377358 | -0.917682 | 0.599375 |
|  | F | 4.659887 | 0.186320 | -0.105798 |
|  | F | 5.470073 | -1.250943 | 1.302916 |
|  | F | 4.123428 | -1.908661 | -0.265884 |
|  | O | -1.893508 | 0.300414 | 0.751847 |
|  | S | -2.950427 | 0.641143 | 1.769371 |
|  | O | -2.748095 | 1.908249 | 2.411096 |
|  | O | -3.343991 | -0.492265 | 2.560926 |
|  | C | -4.377284 | 0.917441 | 0.599326 |
|  | F | -4.659403 | -0.187028 | -0.105276 |
|  | F | -5.470084 | 1.250582 | 1.302789 |
|  | F | -4.123838 | 1.908101 | -0.266440 |
|  | Si | 0.000014 | -0.000187 | 0.735372 |

## MeSi(SO_3_CF_3_)_3_

| Figure S 144: Representation of the B3LYP/def2-TZVPP structure of MeSi(SO_3_CF_3_)_3_. | Si | -0.155694 | -0.213762 | -0.588099 |
| --- | --- | --- | --- | --- |
|  | C | 0.259818 | -0.320335 | -2.361723 |
|  | H | -0.471012 | 0.237222 | -2.948101 |
|  | H | 1.250812 | 0.098607 | -2.540334 |
|  | H | 0.255606 | -1.360729 | -2.687206 |
|  | O | 0.897742 | -0.991177 | 0.442553 |
|  | S | 1.486128 | -2.463921 | 0.480485 |
|  | O | 1.108390 | -3.154755 | -0.710920 |
|  | O | 1.284957 | -3.002692 | 1.778586 |
|  | C | 3.295802 | -2.014204 | 0.329011 |
|  | F | 3.663057 | -1.247711 | 1.346938 |
|  | F | 4.000152 | -3.139493 | 0.337142 |
|  | F | 3.504841 | -1.367088 | -0.815704 |
|  | O | -1.636790 | -0.856941 | -0.171307 |
|  | S | -3.092984 | -0.625352 | -0.759130 |
|  | O | -3.707379 | -1.890523 | -0.953629 |
|  | O | -3.030917 | 0.370159 | -1.781794 |
|  | C | -3.873374 | 0.154670 | 0.753287 |
|  | F | -3.223943 | 1.269118 | 1.074722 |
|  | F | -5.134601 | 0.442661 | 0.455177 |
|  | F | -3.835014 | -0.691519 | 1.774692 |
|  | O | -0.223409 | 1.339124 | 0.016970 |
|  | S | 0.806727 | 2.542758 | -0.017195 |
|  | O | 0.136942 | 3.704304 | -0.485609 |
|  | O | 2.041217 | 2.085580 | -0.572552 |
|  | C | 1.049104 | 2.735412 | 1.828052 |
|  | F | 1.532705 | 1.608448 | 2.340991 |
|  | F | 1.916363 | 3.722160 | 2.021867 |
|  | F | -0.105244 | 3.029979 | 2.412830 |
|  |  |  |  |  |

## [MeSi(SO_3_CF_3_)_3_F]^–^

| Figure S 145: Representation of the B3LYP/def2-TZVPP structure of [MeSi(SO_3_CF_3_)_3_F]^–^. | Si | -0.870850 | -0.308503 | 0.154550 |
| --- | --- | --- | --- | --- |
|  | O | 0.770664 | -0.413944 | -0.331818 |
|  | S | 2.169810 | -0.310980 | 0.327899 |
|  | O | 2.570261 | -1.549696 | 0.913331 |
|  | O | 2.338454 | 0.929406 | 1.018086 |
|  | C | 3.149819 | -0.158524 | -1.253187 |
|  | F | 2.820221 | 0.937488 | -1.927734 |
|  | F | 4.447734 | -0.085358 | -0.922433 |
|  | F | 2.972263 | -1.224787 | -2.033827 |
|  | F | -1.658575 | -1.694183 | 0.278113 |
|  | O | -0.274891 | -0.496793 | 1.908382 |
|  | S | -0.976626 | -0.819920 | 3.215881 |
|  | O | -0.700303 | -2.147713 | 3.672399 |
|  | O | -2.323483 | -0.320398 | 3.249686 |
|  | C | -0.003935 | 0.300849 | 4.345505 |
|  | F | -0.125604 | 1.581201 | 3.975088 |
|  | F | -0.482274 | 0.179972 | 5.593743 |
|  | F | 1.289833 | -0.018852 | 4.358306 |
|  | C | -1.662243 | 1.350485 | 0.402166 |
|  | H | -2.635141 | 1.372814 | -0.088947 |
|  | H | -1.786396 | 1.599714 | 1.451460 |
|  | H | -1.038098 | 2.108819 | -0.075033 |
|  | O | -1.295350 | -0.377770 | -1.663573 |
|  | S | -1.293642 | 0.551256 | -2.854530 |
|  | O | -0.273668 | 1.559708 | -2.784995 |
|  | O | -2.624021 | 0.924933 | -3.240762 |
|  | C | -0.730944 | -0.647731 | -4.166371 |
|  | F | -1.586923 | -1.668157 | -4.288603 |
|  | F | -0.664763 | -0.012012 | -5.345464 |
|  | F | 0.478672 | -1.141327 | -3.887319 |
|  |  |  |  |  |

## PhSi(SO_3_CF_3_)_3_

| Figure S 146: Representation of the B3LYP/def2-TZVPP structure of PhSi(SO_3_CF_3_)_3_. | Si | 0.377677 | 0.080500 | 0.188162 |
| --- | --- | --- | --- | --- |
|  | O | 1.987258 | -0.327063 | 0.131218 |
|  | O | 0.144506 | 1.572280 | -0.485125 |
|  | O | 0.075584 | 0.146899 | 1.829075 |
|  | C | -0.545802 | -1.172398 | -0.769011 |
|  | S | 3.327768 | 0.274830 | 0.736552 |
|  | S | 0.177845 | 3.127922 | -0.149221 |
|  | C | -1.330209 | 3.590448 | -1.155364 |
|  | C | 4.235336 | 0.498678 | -0.884492 |
|  | S | -1.235934 | 0.397267 | 2.685109 |
|  | C | -1.447537 | -1.331975 | 3.367633 |
|  | C | 0.016159 | -2.435813 | -1.004116 |
|  | C | -0.688566 | -3.397234 | -1.716262 |
|  | C | -1.959640 | -3.110463 | -2.201775 |
|  | C | -2.528783 | -1.861590 | -1.975452 |
|  | C | -1.828861 | -0.896985 | -1.263793 |
|  | H | -2.287938 | 0.066867 | -1.089016 |
|  | H | -2.506964 | -3.860313 | -2.757823 |
|  | H | -3.517735 | -1.638274 | -2.352553 |
|  | H | 1.006787 | -2.669712 | -0.637170 |
|  | H | -0.244557 | -4.367627 | -1.893461 |
|  | F | 5.415957 | 1.046454 | -0.623551 |
|  | F | 3.539569 | 1.293718 | -1.689654 |
|  | F | 4.410813 | -0.681830 | -1.471523 |
|  | F | -1.537117 | 4.893054 | -1.001192 |
|  | F | -1.122761 | 3.320787 | -2.440746 |
|  | F | -0.387374 | -1.673274 | 4.089729 |
|  | F | -1.603713 | -2.206691 | 2.374562 |
|  | F | -2.532204 | -1.337143 | 4.134387 |
|  | F | -2.395879 | 2.916364 | -0.731257 |
|  | O | -0.131206 | 3.332753 | 1.224633 |
|  | O | 1.302050 | 3.713118 | -0.790995 |
|  | O | 3.067829 | 1.567064 | 1.278105 |
|  | O | 3.994311 | -0.750791 | 1.460031 |
|  | O | -0.917596 | 1.233793 | 3.784710 |
|  | O | -2.329074 | 0.646378 | 1.799650 |
|  |  |  |  |  |

## [PhSi(SO_3_CF_3_)_3_F]^–^

| Figure S 147: Representation of the B3LYP/def2-TZVPP structure of [PhSi(SO_3_CF_3_)_3_F]^–^. | Si | -0.147680 | 0.874653 | -0.093023 |
| --- | --- | --- | --- | --- |
|  | F | -0.251164 | 2.460150 | -0.289638 |
|  | C | 1.408642 | -0.020500 | 0.427467 |
|  | C | 2.654389 | 0.323672 | -0.109650 |
|  | C | 1.361149 | -1.046930 | 1.378532 |
|  | C | 3.813405 | -0.325881 | 0.302077 |
|  | H | 2.727327 | 1.102344 | -0.857085 |
|  | C | 2.514456 | -1.715776 | 1.770170 |
|  | H | 0.415713 | -1.330455 | 1.820915 |
|  | C | 3.746306 | -1.351384 | 1.237713 |
|  | H | 4.767529 | -0.034324 | -0.118891 |
|  | H | 2.450427 | -2.515648 | 2.497267 |
|  | H | 4.647580 | -1.864899 | 1.549509 |
|  | O | -1.579335 | -0.041646 | -0.305937 |
|  | S | -2.155510 | -1.064523 | -1.316834 |
|  | O | -1.264709 | -2.166700 | -1.506736 |
|  | O | -2.793868 | -0.424467 | -2.419707 |
|  | C | -3.530402 | -1.690418 | -0.221273 |
|  | F | -4.396013 | -0.724434 | 0.068717 |
|  | F | -4.175416 | -2.661888 | -0.882110 |
|  | F | -3.047558 | -2.200547 | 0.912947 |
|  | O | 0.213502 | 0.675442 | -1.895880 |
|  | S | 0.644437 | 1.561616 | -3.055990 |
|  | O | 1.886182 | 2.234793 | -2.790417 |
|  | O | -0.438934 | 2.285354 | -3.646075 |
|  | C | 1.068984 | 0.208467 | -4.268469 |
|  | F | 0.000434 | -0.523425 | -4.578613 |
|  | F | 1.538361 | 0.770924 | -5.393074 |
|  | F | 2.015774 | -0.599714 | -3.779829 |
|  | O | -0.641118 | 1.057470 | 1.670069 |
|  | S | -1.616094 | 1.894952 | 2.481030 |
|  | O | -2.878603 | 2.080136 | 1.829046 |
|  | O | -0.970095 | 3.002835 | 3.118718 |
|  | C | -1.931725 | 0.669498 | 3.851602 |
|  | F | -0.807042 | 0.384241 | 4.519387 |
|  | F | -2.811456 | 1.191077 | 4.717067 |
|  | F | -2.437873 | -0.474064 | 3.376997 |
|  |  |  |  |  |

## Si(SO_3_CF_3_)_4_

| Figure S 148: Representation of the B3LYP/def2-TZVPP structure of Si(SO_3_CF_3_)_4_. | Si | -0.016758 | -0.052684 | 0.226340 |
| --- | --- | --- | --- | --- |
|  | O | -0.934189 | 0.699120 | -0.905136 |
|  | S | -0.999093 | 2.190680 | -1.488173 |
|  | O | -2.353898 | 2.611028 | -1.463067 |
|  | C | -0.565111 | 1.771815 | -3.262365 |
|  | O | 0.070632 | 2.950372 | -0.933375 |
|  | F | 0.646215 | 1.227098 | -3.315030 |
|  | F | -1.455975 | 0.922458 | -3.757528 |
|  | F | -0.580810 | 2.899451 | -3.959538 |
|  | O | 1.531690 | -0.016041 | -0.301230 |
|  | S | 2.912102 | -0.616492 | 0.262951 |
|  | O | 3.921138 | 0.362291 | 0.089605 |
|  | C | 3.167782 | -1.927407 | -1.051638 |
|  | O | 2.643496 | -1.275212 | 1.497979 |
|  | F | 2.126746 | -2.754536 | -1.076448 |
|  | F | 3.307885 | -1.355774 | -2.240567 |
|  | F | 4.267419 | -2.600109 | -0.741670 |
|  | O | -0.499142 | -1.619460 | 0.239612 |
|  | S | -1.847588 | -2.349709 | 0.713252 |
|  | O | -1.483990 | -3.467875 | 1.505813 |
|  | C | -2.384183 | -2.994764 | -0.962478 |
|  | O | -2.786075 | -1.360885 | 1.127068 |
|  | F | -2.537403 | -1.983667 | -1.812423 |
|  | F | -1.477821 | -3.839097 | -1.436850 |
|  | F | -3.541953 | -3.620621 | -0.798021 |
|  | O | -0.253691 | 0.614106 | 1.689659 |
|  | S | 0.541458 | 1.344477 | 2.884455 |
|  | O | 0.514373 | 0.492917 | 4.018365 |
|  | C | -0.706762 | 2.716316 | 3.166679 |
|  | O | 1.732911 | 1.916609 | 2.358390 |
|  | F | -0.906293 | 3.389711 | 2.041630 |
|  | F | -1.851644 | 2.202865 | 3.592350 |
|  | F | -0.201467 | 3.523018 | 4.091387 |
|  |  |  |  |  |

## [Si(SO_3_CF_3_)_4_F]^–^

| Figure S 149: Representation of the B3LYP/def2-TZVPP structure of [Si(SO_3_CF_3_)_4_F]^–^. | Si | 0.000000 | 0.000000 | 0.544063 |
| --- | --- | --- | --- | --- |
|  | O | 0.299267 | -1.750637 | 0.457397 |
|  | S | 0.478810 | -2.997957 | 1.328714 |
|  | O | 1.019868 | -2.710812 | 2.618867 |
|  | O | 1.014756 | -4.051656 | 0.525616 |
|  | C | -1.305278 | -3.461770 | 1.617160 |
|  | F | -1.922687 | -3.738721 | 0.467918 |
|  | F | -1.962734 | -2.470217 | 2.227163 |
|  | F | -1.351825 | -4.547276 | 2.398893 |
|  | O | -0.299267 | 1.750637 | 0.457397 |
|  | S | -0.478810 | 2.997957 | 1.328714 |
|  | O | -1.019868 | 2.710812 | 2.618867 |
|  | O | -1.014756 | 4.051656 | 0.525616 |
|  | C | 1.305278 | 3.461770 | 1.617160 |
|  | F | 1.922687 | 3.738721 | 0.467918 |
|  | F | 1.962734 | 2.470217 | 2.227163 |
|  | F | 1.351825 | 4.547276 | 2.398893 |
|  | O | 1.380653 | 0.309760 | -0.400144 |
|  | S | 2.781580 | -0.252308 | -0.774721 |
|  | O | 3.215528 | -1.268893 | 0.125472 |
|  | O | 3.620530 | 0.851535 | -1.108397 |
|  | C | 2.375128 | -1.076499 | -2.402789 |
|  | F | 1.892010 | -0.179963 | -3.266011 |
|  | F | 1.489031 | -2.051804 | -2.252313 |
|  | F | 3.506928 | -1.584944 | -2.899611 |
|  | O | -1.380653 | -0.309760 | -0.400144 |
|  | S | -2.781580 | 0.252308 | -0.774721 |
|  | O | -3.215528 | 1.268893 | 0.125472 |
|  | O | -3.620530 | -0.851535 | -1.108397 |
|  | C | -2.375128 | 1.076499 | -2.402789 |
|  | F | -1.892010 | 0.179963 | -3.266011 |
|  | F | -1.489031 | 2.051804 | -2.252313 |
|  | F | -3.506928 | 1.584944 | -2.899611 |
|  | F | 0.000000 | 0.000000 | 2.129510 |
|  |  |  |  |  |

## Me_2_Si(C_2_F_5_)_2_

| Figure S 150: Representation of the B3LYP/def2-TZVPP structure of Me_2_Si(C_2_F_5_)_2_. | Si | 0.423233 | -0.463091 | 0.279634 |
| --- | --- | --- | --- | --- |
|  | C | 1.057153 | -2.205398 | 0.453651 |
|  | C | 1.181360 | 0.753896 | 1.467843 |
|  | C | 0.872088 | 0.034765 | -1.561335 |
|  | C | 0.428602 | 1.383078 | -2.169894 |
|  | C | -1.530775 | -0.565404 | 0.475884 |
|  | C | -2.266987 | 0.647440 | 1.089177 |
|  | F | 2.252137 | 0.038243 | -1.600978 |
|  | F | 0.453509 | -0.940264 | -2.424251 |
|  | F | 0.971208 | 1.570522 | -3.375904 |
|  | F | 0.814716 | 2.396108 | -1.373944 |
|  | F | -0.901666 | 1.428587 | -2.293414 |
|  | F | -1.865460 | 0.834661 | 2.357835 |
|  | F | -1.982295 | 1.760287 | 0.394618 |
|  | F | -3.589251 | 0.476063 | 1.089110 |
|  | F | -2.120302 | -0.821834 | -0.730586 |
|  | F | -1.818597 | -1.631790 | 1.295013 |
|  | H | 0.566864 | -2.880496 | -0.247673 |
|  | H | 0.877719 | -2.575076 | 1.463851 |
|  | H | 2.130867 | -2.235257 | 0.262428 |
|  | H | 2.260198 | 0.793135 | 1.308246 |
|  | H | 1.002238 | 0.441891 | 2.497739 |
|  | H | 0.783440 | 1.759937 | 1.342949 |
|  |  |  |  |  |

## [Me_2_Si(C_2_F_5_)_2_F]^–^

| Figure S 151: Representation of the B3LYP/def2-TZVPP structure of [Me_2_Si(C_2_F_5_)_2_F]^–^. | Si | -0.218148 | -0.186805 | 0.000000 |
| --- | --- | --- | --- | --- |
|  | C | -1.200981 | -1.813391 | 0.000000 |
|  | H | -2.269685 | -1.588805 | 0.000000 |
|  | H | -0.988406 | -2.409529 | -0.887138 |
|  | H | -0.988406 | -2.409529 | 0.887138 |
|  | F | -0.963334 | 1.288433 | 0.000000 |
|  | C | -0.366875 | -0.079885 | -2.084031 |
|  | C | 0.137847 | 1.136455 | -2.883666 |
|  | F | 0.290684 | -1.162221 | -2.678841 |
|  | F | -1.695532 | -0.213303 | -2.483639 |
|  | F | -0.553689 | 2.250226 | -2.603943 |
|  | F | 0.051302 | 0.954745 | -4.227069 |
|  | F | 1.438685 | 1.387133 | -2.617606 |
|  | C | -0.366875 | -0.079885 | 2.084031 |
|  | C | 0.137847 | 1.136455 | 2.883666 |
|  | F | -1.695532 | -0.213303 | 2.483639 |
|  | F | 0.290684 | -1.162221 | 2.678841 |
|  | F | 1.438685 | 1.387133 | 2.617606 |
|  | F | 0.051302 | 0.954745 | 4.227069 |
|  | F | -0.553689 | 2.250226 | 2.603943 |
|  | C | 1.674152 | -0.333117 | 0.000000 |
|  | H | 2.033235 | -0.855843 | -0.886553 |
|  | H | 2.124763 | 0.661685 | 0.000000 |
|  | H | 2.033235 | -0.855843 | 0.886553 |
|  |  |  |  |  |

## Ph_2_Si(C_2_F_5_)_2_

| Figure S 152: Representation of the B3LYP/def2-TZVPP structure of Ph_2_Si(C_2_F_5_)_2_. | Si | -0.318663 | 0.027380 | -0.415994 |
| --- | --- | --- | --- | --- |
|  | C | 0.259998 | -1.745449 | -0.319869 |
|  | C | 1.458100 | -2.050885 | 0.339426 |
|  | C | -0.447820 | -2.791924 | -0.930135 |
|  | C | 1.933253 | -3.356654 | 0.389533 |
|  | H | 2.027497 | -1.265927 | 0.819844 |
|  | C | 0.025117 | -4.095990 | -0.877268 |
|  | H | -1.374265 | -2.594118 | -1.452420 |
|  | C | 1.216934 | -4.380531 | -0.218097 |
|  | H | 2.861448 | -3.571902 | 0.902544 |
|  | H | -0.535906 | -4.890021 | -1.352342 |
|  | H | 1.585157 | -5.397553 | -0.179311 |
|  | C | 0.478870 | 1.120255 | 0.869181 |
|  | C | 1.300537 | 2.215989 | 0.574266 |
|  | C | 0.266680 | 0.797342 | 2.219784 |
|  | C | 1.882578 | 2.964437 | 1.591589 |
|  | H | 1.497330 | 2.493843 | -0.450470 |
|  | C | 0.846115 | 1.546131 | 3.235112 |
|  | H | -0.359058 | -0.045408 | 2.487567 |
|  | C | 1.655370 | 2.633196 | 2.921718 |
|  | H | 2.513543 | 3.807258 | 1.341691 |
|  | H | 0.665302 | 1.281637 | 4.268643 |
|  | H | 2.107350 | 3.218474 | 3.711903 |
|  | C | 0.104804 | 0.568120 | -2.266377 |
|  | C | -0.465923 | 1.837645 | -2.942982 |
|  | C | -2.278526 | -0.024503 | -0.194203 |
|  | C | -3.035252 | 1.255104 | 0.247116 |
|  | F | 1.475069 | 0.701550 | -2.308383 |
|  | F | -0.218522 | -0.467182 | -3.100092 |
|  | F | 0.097980 | 2.028223 | -4.140510 |
|  | F | -0.221167 | 2.923837 | -2.191202 |
|  | F | -1.785880 | 1.728449 | -3.114960 |
|  | F | -2.742534 | 1.555487 | 1.518714 |
|  | F | -2.684840 | 2.297705 | -0.519446 |
|  | F | -4.357579 | 1.088533 | 0.150961 |
|  | F | -2.854534 | -0.439243 | -1.367640 |
|  | F | -2.578563 | -0.973304 | 0.752110 |
|  |  |  |  |  |

## [Ph_2_Si(C_2_F_5_)_2_F]^–^

| Figure S 153: Representation of the B3LYP/def2-TZVPP structure of [Ph_2_Si(C_2_F_5_)_2_F]^–^. | Si | 0.413571 | 0.306672 | -0.064853 |
| --- | --- | --- | --- | --- |
|  | F | 0.286424 | 2.004964 | 0.047482 |
|  | C | -0.518388 | 0.322004 | -1.734005 |
|  | C | 0.091282 | 0.053754 | -2.965621 |
|  | C | -1.862241 | 0.719121 | -1.758947 |
|  | C | -0.607791 | 0.171647 | -4.163872 |
|  | H | 1.126945 | -0.254907 | -2.997463 |
|  | C | -2.573214 | 0.818842 | -2.950405 |
|  | H | -2.364597 | 0.966918 | -0.831698 |
|  | C | -1.946333 | 0.546709 | -4.161926 |
|  | H | -0.103657 | -0.035461 | -5.100713 |
|  | H | -3.614597 | 1.118940 | -2.932775 |
|  | H | -2.493711 | 0.630086 | -5.093405 |
|  | C | -0.512505 | 0.145549 | 1.607113 |
|  | C | -1.757546 | -0.475419 | 1.766782 |
|  | C | 0.034349 | 0.768550 | 2.739526 |
|  | C | -2.422526 | -0.477478 | 2.990857 |
|  | H | -2.220719 | -0.975307 | 0.927987 |
|  | C | -0.611345 | 0.749086 | 3.970456 |
|  | H | 0.982843 | 1.284089 | 2.662706 |
|  | C | -1.849468 | 0.128440 | 4.101872 |
|  | H | -3.389234 | -0.960709 | 3.073776 |
|  | H | -0.150615 | 1.229033 | 4.826209 |
|  | H | -2.360450 | 0.119193 | 5.057427 |
|  | C | 0.638765 | -1.825182 | -0.244735 |
|  | C | 1.355777 | -2.748918 | 0.769779 |
|  | F | -0.668350 | -2.316998 | -0.263802 |
|  | F | 1.169630 | -2.228080 | -1.463253 |
|  | F | 2.690608 | -2.624402 | 0.700243 |
|  | F | 1.080070 | -4.057967 | 0.542683 |
|  | F | 0.985455 | -2.491985 | 2.036254 |
|  | C | 2.407687 | 0.523467 | -0.053651 |
|  | C | 3.037970 | 1.929002 | -0.245636 |
|  | F | 2.914852 | 0.090746 | 1.159994 |
|  | F | 3.019288 | -0.242418 | -1.023165 |
|  | F | 2.642784 | 2.493252 | -1.395321 |
|  | F | 4.390148 | 1.834431 | -0.301206 |
|  | F | 2.758838 | 2.760737 | 0.765305 |
|  | Si | 0.413571 | 0.306672 | -0.064853 |

| Figure S 154: Representation of the B3LYP/def2-TZVPP structure of [Ph_2_Si(C_2_F_5_)_2_F]^–^. | Si | 0.815860 | -0.007102 | 0.004341 |
| --- | --- | --- | --- | --- |
|  | F | 2.457229 | -0.024618 | 0.011823 |
|  | C | -0.190015 | -1.439086 | -0.761608 |
|  | C | -1.504401 | -1.266561 | -1.217011 |
|  | C | 0.385627 | -2.704389 | -0.944591 |
|  | C | -2.216485 | -2.306653 | -1.805472 |
|  | H | -1.981142 | -0.299244 | -1.130924 |
|  | C | -0.316106 | -3.745636 | -1.543284 |
|  | H | 1.398874 | -2.885327 | -0.612736 |
|  | C | -1.624822 | -3.553847 | -1.970796 |
|  | H | -3.232914 | -2.138592 | -2.142102 |
|  | H | 0.161621 | -4.709723 | -1.672901 |
|  | H | -2.175241 | -4.364747 | -2.432930 |
|  | C | -0.172655 | 1.442915 | 0.759619 |
|  | C | 0.422880 | 2.697649 | 0.951521 |
|  | C | -1.495518 | 1.293314 | 1.198363 |
|  | C | -0.268062 | 3.750776 | 1.541969 |
|  | H | 1.443868 | 2.860529 | 0.634532 |
|  | C | -2.196821 | 2.345550 | 1.778060 |
|  | H | -1.987616 | 0.334257 | 1.106899 |
|  | C | -1.585589 | 3.582107 | 1.951751 |
|  | H | 0.225063 | 4.706074 | 1.678898 |
|  | H | -3.220145 | 2.195058 | 2.101839 |
|  | H | -2.127978 | 4.402702 | 2.406207 |
|  | C | 1.000802 | 0.999645 | -1.826766 |
|  | C | 1.628919 | 0.333165 | -3.071641 |
|  | F | -0.236475 | 1.449695 | -2.277823 |
|  | F | 1.765565 | 2.153310 | -1.664802 |
|  | F | 2.859096 | -0.143219 | -2.814774 |
|  | F | 1.755567 | 1.202131 | -4.105754 |
|  | F | 0.882575 | -0.690365 | -3.523334 |
|  | C | 0.963090 | -1.017315 | 1.837080 |
|  | C | 1.583656 | -0.360466 | 3.090825 |
|  | F | -0.286120 | -1.450590 | 2.271538 |
|  | F | 1.713519 | -2.181438 | 1.684140 |
|  | F | 2.822474 | 0.101788 | 2.849565 |
|  | F | 1.687634 | -1.232682 | 4.124705 |
|  | F | 0.844187 | 0.670935 | 3.535574 |
|  |  |  |  |  |

## MeSi(C_2_F_5_)_3_

| Figure S 155: Representation of the B3LYP/def2-TZVPP structure of MeSi(C_2_F_5_)_3_. | Si | -0.068109 | -0.186179 | -0.123861 |
| --- | --- | --- | --- | --- |
|  | C | 0.826077 | -1.932339 | -0.261768 |
|  | C | 0.233995 | -3.079895 | 0.597635 |
|  | F | 0.774785 | -2.365309 | -1.556854 |
|  | F | 2.140656 | -1.811645 | 0.076289 |
|  | F | 0.152271 | -2.706048 | 1.883094 |
|  | F | 0.981641 | -4.178030 | 0.520353 |
|  | F | -1.005097 | -3.374784 | 0.171096 |
|  | C | 0.341022 | 0.626788 | -1.861074 |
|  | C | 0.010498 | 2.121508 | -2.101397 |
|  | F | 1.655725 | 0.455307 | -2.171896 |
|  | F | -0.400410 | -0.071257 | -2.781977 |
|  | F | -1.275928 | 2.357503 | -1.784733 |
|  | F | 0.195664 | 2.448391 | -3.380047 |
|  | F | 0.781900 | 2.905074 | -1.344732 |
|  | C | 0.860677 | 0.761880 | 1.329090 |
|  | C | 0.020758 | 1.807380 | 2.105031 |
|  | F | 1.296451 | -0.137074 | 2.259784 |
|  | F | 1.957341 | 1.405876 | 0.838705 |
|  | F | -0.513249 | 2.694305 | 1.249700 |
|  | F | 0.758131 | 2.465084 | 2.995524 |
|  | F | -0.984054 | 1.194380 | 2.749966 |
|  | C | -1.901834 | -0.327472 | 0.115803 |
|  | H | -2.337690 | -0.888807 | -0.711270 |
|  | H | -2.138027 | -0.844014 | 1.045722 |
|  | H | -2.363194 | 0.659374 | 0.141818 |
|  |  |  |  |  |

## [MeSi(C_2_F_5_)_3_F]^–^

| Figure S 156: Representation of the B3LYP/def2-TZVPP structure of [MeSi(C_2_F_5_)_3_F]^–^. | Si | -0.229678 | -0.174798 | 0.334813 |
| --- | --- | --- | --- | --- |
|  | F | -0.187679 | 0.281368 | 1.969033 |
|  | C | -1.009683 | -1.839350 | 0.791337 |
|  | H | -0.292675 | -2.373109 | 1.419847 |
|  | H | -1.883064 | -1.642102 | 1.415782 |
|  | H | -1.298497 | -2.477528 | -0.036351 |
|  | C | 1.756349 | 0.046826 | 0.278671 |
|  | C | 2.516054 | 0.662636 | 1.483316 |
|  | F | 2.336089 | -1.204094 | 0.122914 |
|  | F | 2.160061 | 0.802548 | -0.795050 |
|  | F | 2.084390 | 1.898390 | 1.763368 |
|  | F | 3.838315 | 0.762607 | 1.199667 |
|  | F | 2.420014 | -0.091683 | 2.585730 |
|  | C | -1.325350 | 1.473586 | -0.026331 |
|  | C | -2.120109 | 2.101084 | 1.155242 |
|  | F | -0.501160 | 2.488736 | -0.470291 |
|  | F | -2.281434 | 1.297442 | -0.995652 |
|  | F | -2.903222 | 1.180717 | 1.748320 |
|  | F | -2.942353 | 3.078674 | 0.703370 |
|  | F | -1.338142 | 2.660506 | 2.080022 |
|  | C | -0.284917 | -0.597220 | -1.749813 |
|  | C | 0.596499 | -1.677593 | -2.433428 |
|  | F | -1.576290 | -1.066226 | -1.991741 |
|  | F | -0.137560 | 0.507945 | -2.565072 |
|  | F | 1.863347 | -1.279856 | -2.603258 |
|  | F | 0.126555 | -1.996705 | -3.663599 |
|  | F | 0.614139 | -2.822802 | -1.720844 |
|  |  |  |  |  |

| Figure S 157: Representation of the B3LYP/def2-TZVPP structure of [MeSi(C_2_F_5_)_3_F]^–^. | Si | 0.199708 | -0.216941 | 0.107544 |
| --- | --- | --- | --- | --- |
|  | C | -0.924653 | -1.736757 | 0.241936 |
|  | H | -0.316528 | -2.603282 | 0.508433 |
|  | H | -1.662444 | -1.602470 | 1.031727 |
|  | H | -1.434883 | -1.952875 | -0.693542 |
|  | F | 1.838304 | -0.089899 | 0.163693 |
|  | C | 0.352282 | -0.154380 | 2.190788 |
|  | C | 1.153904 | 0.904776 | 2.984797 |
|  | F | -0.864172 | -0.226973 | 2.848652 |
|  | F | 0.994414 | -1.353891 | 2.513724 |
|  | F | 2.448080 | 0.926720 | 2.637353 |
|  | F | 1.113904 | 0.660908 | 4.318894 |
|  | F | 0.661416 | 2.142081 | 2.804067 |
|  | C | -0.832861 | 1.502153 | -0.038032 |
|  | C | -1.932425 | 1.751645 | -1.107912 |
|  | F | -1.507568 | 1.720768 | 1.142112 |
|  | F | 0.054041 | 2.548976 | -0.177057 |
|  | F | -1.439842 | 1.922685 | -2.335286 |
|  | F | -2.636015 | 2.867748 | -0.813274 |
|  | F | -2.807789 | 0.730218 | -1.133482 |
|  | C | 0.507374 | -0.427656 | -1.929102 |
|  | C | 1.451025 | -1.557271 | -2.413873 |
|  | F | -0.678560 | -0.683471 | -2.610915 |
|  | F | 1.031169 | 0.715495 | -2.507723 |
|  | F | 2.731445 | -1.332053 | -2.098500 |
|  | F | 1.401684 | -1.710105 | -3.758201 |
|  | F | 1.098991 | -2.746152 | -1.876823 |
|  |  |  |  |  |

## PhSi(C_2_F_5_)_3_

| Figure S 158: Representation of the B3LYP/def2-TZVPP structure of PhSi(C_2_F_5_)_3_. | Si | -0.329595 | 0.328587 | -0.340721 |
| --- | --- | --- | --- | --- |
|  | C | 0.473483 | -0.825681 | 0.870602 |
|  | C | 1.849575 | -1.096316 | 0.861753 |
|  | C | -0.328994 | -1.468442 | 1.827928 |
|  | C | 2.402835 | -1.978479 | 1.781235 |
|  | H | 2.500705 | -0.624290 | 0.141091 |
|  | C | 0.228674 | -2.349377 | 2.743935 |
|  | H | -1.394097 | -1.284533 | 1.864139 |
|  | C | 1.595254 | -2.605360 | 2.722426 |
|  | H | 3.466571 | -2.174496 | 1.760077 |
|  | H | -0.404867 | -2.834594 | 3.474412 |
|  | H | 2.029235 | -3.291575 | 3.437823 |
|  | C | 1.052507 | 1.199149 | -1.431423 |
|  | C | 0.692327 | 2.295159 | -2.468601 |
|  | C | -1.467127 | 1.664889 | 0.550445 |
|  | C | -0.828810 | 2.555094 | 1.648323 |
|  | F | 1.976639 | 1.754622 | -0.588970 |
|  | F | 1.692430 | 0.203712 | -2.124951 |
|  | F | 1.769879 | 2.627191 | -3.183370 |
|  | F | 0.238677 | 3.390188 | -1.852911 |
|  | F | -0.252567 | 1.849378 | -3.304362 |
|  | F | -0.371961 | 1.799115 | 2.655003 |
|  | F | 0.196504 | 3.251363 | 1.141983 |
|  | F | -1.724963 | 3.413620 | 2.137469 |
|  | F | -2.015113 | 2.509133 | -0.367804 |
|  | F | -2.499599 | 0.996468 | 1.156613 |
|  | C | -1.639733 | -0.630123 | -1.459034 |
|  | C | -1.124724 | -1.658596 | -2.501100 |
|  | F | -2.451115 | 0.235523 | -2.127210 |
|  | F | -2.429735 | -1.340316 | -0.590096 |
|  | F | -0.491621 | -1.039722 | -3.501280 |
|  | F | -2.140831 | -2.356792 | -3.012320 |
|  | F | -0.269844 | -2.514499 | -1.921105 |
|  |  |  |  |  |

## [PhSi(C_2_F_5_)_3_F]^–^

| Figure S 159: Representation of the B3LYP/def2-TZVPP structure of [PhSi(C_2_F_5_)_3_F]^–^. | Si | 0.293087 | 0.350131 | 0.216083 |
| --- | --- | --- | --- | --- |
|  | C | -0.647855 | -1.311115 | 0.174543 |
|  | C | -0.176745 | -2.460786 | -0.471246 |
|  | C | -1.848160 | -1.422685 | 0.889879 |
|  | C | -0.870441 | -3.665501 | -0.404223 |
|  | H | 0.743766 | -2.422824 | -1.035090 |
|  | C | -2.558305 | -2.617710 | 0.939283 |
|  | H | -2.237439 | -0.567279 | 1.425781 |
|  | C | -2.068895 | -3.748294 | 0.294705 |
|  | H | -0.472580 | -4.539994 | -0.905577 |
|  | H | -3.489712 | -2.666489 | 1.490994 |
|  | H | -2.613935 | -4.683698 | 0.339171 |
|  | C | 0.231964 | 0.460858 | -1.921243 |
|  | C | 0.412564 | 1.681517 | -2.872969 |
|  | C | 2.303604 | 0.273906 | 0.188224 |
|  | C | 3.085814 | 0.639778 | 1.480794 |
|  | C | -0.708501 | 2.078072 | 0.336737 |
|  | C | -1.158424 | 2.644782 | 1.709924 |
|  | F | 0.359152 | 0.327244 | 1.904489 |
|  | F | 1.169955 | -0.453422 | -2.388846 |
|  | F | -0.994269 | -0.046591 | -2.325959 |
|  | F | 0.518102 | 1.267523 | -4.160460 |
|  | F | 1.512286 | 2.398767 | -2.608995 |
|  | F | -0.640866 | 2.510506 | -2.827643 |
|  | F | 4.416829 | 0.662700 | 1.229703 |
|  | F | 2.749590 | 1.861836 | 1.919351 |
|  | F | 0.049123 | 3.095662 | -0.209171 |
|  | F | 2.899821 | -0.242394 | 2.466508 |
|  | F | -0.124911 | 2.941947 | 2.501282 |
|  | F | -1.859547 | 3.789904 | 1.533347 |
|  | F | -1.970145 | 1.792757 | 2.358138 |
|  | F | 2.707849 | -1.017362 | -0.099671 |
|  | F | -1.873249 | 2.011400 | -0.395548 |
|  | F | 2.860472 | 1.076855 | -0.772295 |
|  |  |  |  |  |

## Si(C_2_F_5_)_4_

| Figure S 160: Representation of the B3LYP/def2-TZVPP structure of Si(C_2_F_5_)_4_. | Si | -0.054105 | -0.048190 | -0.238183 |
| --- | --- | --- | --- | --- |
|  | C | 0.454842 | 0.638230 | -2.015216 |
|  | C | 0.570853 | 2.167780 | -2.280506 |
|  | F | -0.481401 | 0.157433 | -2.883960 |
|  | F | 1.654976 | 0.096993 | -2.362114 |
|  | F | 1.602255 | 2.679633 | -1.602779 |
|  | F | -0.544222 | 2.800626 | -1.904373 |
|  | F | 0.767148 | 2.383852 | -3.581354 |
|  | C | 0.584062 | -1.905955 | -0.312536 |
|  | C | 0.160857 | -2.909923 | 0.793606 |
|  | F | 1.947921 | -1.853134 | -0.289733 |
|  | F | 0.209399 | -2.439917 | -1.506333 |
|  | F | -1.140785 | -3.188751 | 0.700740 |
|  | F | 0.401110 | -2.376602 | 1.999974 |
|  | F | 0.850312 | -4.043635 | 0.680399 |
|  | C | -2.025097 | 0.020867 | -0.188629 |
|  | C | -2.766574 | 0.083732 | 1.174264 |
|  | F | -2.461259 | -1.100190 | -0.831870 |
|  | F | -2.448451 | 1.096948 | -0.904559 |
|  | F | -2.540679 | 1.260563 | 1.764859 |
|  | F | -2.338730 | -0.895732 | 1.980671 |
|  | F | -4.078221 | -0.057373 | 0.990624 |
|  | C | 0.725425 | 0.967999 | 1.249103 |
|  | C | 2.262467 | 0.913446 | 1.482024 |
|  | F | 0.139827 | 0.587434 | 2.416461 |
|  | F | 0.394906 | 2.274898 | 1.026112 |
|  | F | 2.908828 | 1.096377 | 0.323810 |
|  | F | 2.611190 | -0.273120 | 1.983136 |
|  | F | 2.633147 | 1.865712 | 2.336364 |
|  |  |  |  |  |

## [Si(C_2_F_5_)_4_F]^–^

| Figure S 161: Representation of the B3LYP/def2-TZVPP structure of [Si(C_2_F_5_)_4_F]^–^. | Si | 0.192896 | -0.264536 | 0.146851 |
| --- | --- | --- | --- | --- |
|  | F | 0.977420 | -1.736523 | 0.152130 |
|  | C | 0.854169 | 0.069526 | 2.041779 |
|  | C | -0.026903 | 0.567298 | 3.228190 |
|  | F | 1.372974 | -1.103971 | 2.518724 |
|  | F | 1.911882 | 0.951892 | 1.971235 |
|  | F | -0.342765 | 1.860695 | 3.147737 |
|  | F | 0.635183 | 0.404236 | 4.395341 |
|  | F | -1.158453 | -0.147303 | 3.309511 |
|  | C | -0.819709 | 1.555926 | 0.056686 |
|  | C | -1.415441 | 2.177717 | -1.246497 |
|  | F | -1.886464 | 1.465911 | 0.929544 |
|  | F | -0.024788 | 2.565044 | 0.561544 |
|  | F | -0.474822 | 2.807887 | -1.963176 |
|  | F | -2.362689 | 3.094938 | -0.953878 |
|  | F | -1.986686 | 1.259779 | -2.040448 |
|  | C | -1.494378 | -1.363715 | -0.049006 |
|  | C | -1.476852 | -2.504424 | -1.113261 |
|  | F | -1.712599 | -1.993738 | 1.159184 |
|  | F | -2.639022 | -0.669670 | -0.322358 |
|  | F | -1.031003 | -2.031445 | -2.290790 |
|  | F | -2.723624 | -2.981883 | -1.318598 |
|  | F | -0.720788 | -3.545112 | -0.762992 |
|  | C | 1.461173 | 0.329960 | -1.293168 |
|  | C | 2.736702 | -0.503642 | -1.592721 |
|  | F | 0.780591 | 0.366630 | -2.496163 |
|  | F | 1.931983 | 1.598433 | -1.073176 |
|  | F | 3.498427 | -0.636202 | -0.498575 |
|  | F | 3.488488 | 0.123551 | -2.527218 |
|  | F | 2.455100 | -1.717258 | -2.076430 |
|  |  |  |  |  |

## Ph_2_Si(cat^Cl^)

| Figure S 162: Representation of the B3LYP/def2-TZVPP structure of Ph_2_Si(cat^Cl^). | C | -3.466338 | -0.018306 | -2.350597 |
| --- | --- | --- | --- | --- |
|  | C | -3.799261 | -1.368504 | -2.389268 |
|  | C | -3.027325 | -2.295221 | -1.696799 |
|  | C | -2.363616 | 0.403058 | -1.618185 |
|  | C | -1.568358 | -0.517280 | -0.920256 |
|  | C | -1.919325 | -1.873736 | -0.971379 |
|  | H | -2.122329 | 1.458878 | -1.591384 |
|  | H | -4.066280 | 0.704548 | -2.887879 |
|  | H | -4.660305 | -1.697344 | -2.956735 |
|  | Si | -0.057493 | 0.035847 | -0.001516 |
|  | H | -1.327365 | -2.602962 | -0.433296 |
|  | H | -3.287686 | -3.345323 | -1.721911 |
|  | C | -0.280289 | 1.623271 | 0.927533 |
|  | O | 1.341165 | 0.137198 | -0.955928 |
|  | O | 0.524077 | -1.248371 | 0.942183 |
|  | C | 0.764420 | 2.554244 | 1.015171 |
|  | C | -1.478195 | 1.901624 | 1.601140 |
|  | C | -1.624382 | 3.065730 | 2.345196 |
|  | C | 0.616553 | 3.722549 | 1.752888 |
|  | C | -0.576526 | 3.977458 | 2.420639 |
|  | H | 1.695725 | 2.368407 | 0.495526 |
|  | H | -2.306251 | 1.205279 | 1.545671 |
|  | H | -2.553978 | 3.262793 | 2.863017 |
|  | H | -0.690714 | 4.886464 | 2.997083 |
|  | H | 1.430951 | 4.433252 | 1.806427 |
|  | C | 1.774959 | -1.582279 | 0.522881 |
|  | C | 2.231187 | -0.808474 | -0.549314 |
|  | C | 2.560557 | -2.584284 | 1.062692 |
|  | C | 3.481418 | -1.021568 | -1.100581 |
|  | C | 3.833599 | -2.811807 | 0.510710 |
|  | C | 4.290011 | -2.037422 | -0.560970 |
|  | Cl | 1.947333 | -3.512560 | 2.382889 |
|  | Cl | 5.856564 | -2.320035 | -1.235762 |
|  | Cl | 4.830706 | -4.061272 | 1.169560 |
|  | Cl | 3.996790 | -0.033853 | -2.419445 |
|  |  |  |  |  |

## [Ph_2_Si(cat^Cl^)F]^–^

| Figure S 163: Representation of the B3LYP/def2-TZVPP structure of [Ph_2_Si(cat^Cl^)F]^–^. | C | -3.828607 | -1.400795 | -1.671456 |
| --- | --- | --- | --- | --- |
|  | C | -3.417228 | -1.212417 | -2.986194 |
|  | C | -2.138336 | -0.725755 | -3.232629 |
|  | C | -2.970217 | -1.097452 | -0.618688 |
|  | C | -1.670743 | -0.613010 | -0.837288 |
|  | C | -1.280905 | -0.439145 | -2.174331 |
|  | H | -3.309572 | -1.238671 | 0.397580 |
|  | H | -4.821881 | -1.782214 | -1.463300 |
|  | H | -4.084315 | -1.443660 | -3.808579 |
|  | Si | -0.531805 | -0.191326 | 0.647102 |
|  | H | -0.284541 | -0.080034 | -2.388303 |
|  | H | -1.802667 | -0.573806 | -4.252041 |
|  | C | -0.284103 | 1.663811 | 1.071910 |
|  | O | 0.982510 | -0.174369 | -0.475579 |
|  | O | 0.332226 | -1.455914 | 1.505354 |
|  | C | 0.592830 | 2.503606 | 0.367451 |
|  | C | -1.019214 | 2.254793 | 2.112526 |
|  | C | -0.884669 | 3.601785 | 2.435888 |
|  | C | 0.725999 | 3.854029 | 0.677948 |
|  | C | -0.011855 | 4.410523 | 1.716641 |
|  | H | 1.184771 | 2.088626 | -0.435382 |
|  | H | -1.707358 | 1.642415 | 2.677152 |
|  | H | -1.465279 | 4.020157 | 3.250268 |
|  | H | 0.092548 | 5.460838 | 1.963012 |
|  | H | 1.412071 | 4.470544 | 0.108554 |
|  | C | 1.521191 | -1.800059 | 1.017968 |
|  | C | 1.885883 | -1.055533 | -0.124915 |
|  | C | 2.359093 | -2.774394 | 1.527521 |
|  | C | 3.103771 | -1.304668 | -0.746830 |
|  | C | 3.593447 | -3.026749 | 0.894872 |
|  | C | 3.959961 | -2.296030 | -0.232650 |
|  | Cl | 1.859223 | -3.660445 | 2.936601 |
|  | Cl | 5.483078 | -2.603079 | -1.018820 |
|  | Cl | 4.650074 | -4.254945 | 1.531641 |
|  | Cl | 3.525117 | -0.369798 | -2.152566 |
|  | F | -1.750499 | -0.396859 | 1.779563 |
|  |  |  |  |  |

## Si(cat^Cl^)_2_

| Figure S 164: Representation of the B3LYP/def2-TZVPP structure of Si(cat^Cl^)_2_. | Si | -0.012582 | 0.014386 | 0.000000 |
| --- | --- | --- | --- | --- |
|  | O | 0.811706 | 0.735570 | 1.238129 |
|  | O | -0.017692 | -1.638988 | 0.000000 |
|  | O | 0.811706 | 0.735570 | -1.238129 |
|  | O | -1.652533 | 0.221479 | 0.000000 |
|  | C | -1.324289 | -2.066572 | 0.000000 |
|  | C | 1.772040 | 1.557390 | 0.696667 |
|  | C | 1.772040 | 1.557390 | -0.696667 |
|  | C | -2.244225 | -1.020003 | 0.000000 |
|  | C | -1.734144 | -3.386161 | 0.000000 |
|  | C | 2.669811 | 2.321830 | 1.417077 |
|  | C | 3.590946 | 3.105663 | -0.700926 |
|  | C | 3.590946 | 3.105663 | 0.700926 |
|  | C | -3.115377 | -3.646896 | 0.000000 |
|  | C | -4.040921 | -2.593972 | 0.000000 |
|  | Cl | 2.624216 | 2.281775 | 3.137775 |
|  | Cl | 4.727057 | 4.071772 | 1.568788 |
|  | Cl | -0.552302 | -4.638385 | 0.000000 |
|  | C | -3.605416 | -1.257538 | 0.000000 |
|  | Cl | -5.734037 | -2.926874 | 0.000000 |
|  | Cl | -4.695733 | 0.074901 | 0.000000 |
|  | C | 2.669811 | 2.321830 | -1.417077 |
|  | Cl | 4.727057 | 4.071772 | -1.568788 |
|  | Cl | 2.624216 | 2.281775 | -3.137775 |
|  | Cl | -3.662299 | -5.283379 | 0.000000 |
|  |  |  |  |  |

## [Si(cat^Cl^)_2_F]^–^

| Figure S 165: Representation of the B3LYP/def2-TZVPP structure of [Si(cat^Cl^)_2_F]^–^. | O | 1.146399 | 1.113584 | -0.241496 |
| --- | --- | --- | --- | --- |
|  | Si | 0.000000 | 0.000000 | -0.889727 |
|  | O | -1.239066 | 1.255640 | -0.706211 |
|  | O | 1.239066 | -1.255640 | -0.706211 |
|  | O | -1.146399 | -1.113584 | -0.241496 |
|  | C | -0.730656 | 2.412027 | -0.314011 |
|  | C | -0.646566 | -2.340132 | -0.040763 |
|  | C | 0.730656 | -2.412027 | -0.314011 |
|  | C | -1.347222 | -3.446047 | 0.401005 |
|  | C | 1.411602 | -3.608942 | -0.145769 |
|  | C | -0.660596 | -4.663260 | 0.571699 |
|  | C | 0.705696 | -4.741220 | 0.299643 |
|  | Cl | -3.043149 | -3.295037 | 0.729178 |
|  | Cl | 1.552431 | -6.245003 | 0.509067 |
|  | Cl | -1.529255 | -6.064766 | 1.121694 |
|  | Cl | 3.110237 | -3.660873 | -0.489593 |
|  | F | 0.000000 | 0.000000 | -2.499113 |
|  | C | 0.646566 | 2.340132 | -0.040763 |
|  | C | -1.411602 | 3.608942 | -0.145769 |
|  | C | -0.705696 | 4.741220 | 0.299643 |
|  | C | 1.347222 | 3.446047 | 0.401005 |
|  | C | 0.660596 | 4.663260 | 0.571699 |
|  | Cl | -3.110237 | 3.660873 | -0.489593 |
|  | Cl | 3.043149 | 3.295037 | 0.729178 |
|  | Cl | -1.552431 | 6.245003 | 0.509067 |
|  | Cl | 1.529255 | 6.064766 | 1.121694 |
|  |  |  |  |  |

## Me_3_Si^+^

| Si | 0.000000 | 0.000000 | 0.428274 |
| --- | --- | --- | --- |
| F | 0.000000 | 0.000000 | 2.048877 |
| C | -0.893436 | 1.547477 | -0.119678 |
| H | -0.931994 | 1.614262 | -1.209254 |
| H | -1.919897 | 1.560879 | 0.251608 |
| H | -0.391813 | 2.443119 | 0.251608 |
| C | -0.893436 | -1.547477 | -0.119678 |
| H | -0.931994 | -1.614262 | -1.209254 |
| H | -0.391813 | -2.443119 | 0.251608 |
| H | -1.919897 | -1.560879 | 0.251608 |
| C | 1.786872 | 0.000000 | -0.119678 |
| H | 2.311710 | -0.882240 | 0.251608 |
| H | 1.863989 | 0.000000 | -1.209254 |
| H | 2.311710 | 0.882240 | 0.251608 |
|  |  |  |  |

## Me_3_SiF

| C | -1.786651 | 0.377937 | 0.121750 |
| --- | --- | --- | --- |
| H | -2.309594 | -0.060461 | -0.735828 |
| H | -2.205782 | -0.106556 | 1.010344 |
| H | -1.995609 | 1.445595 | 0.160506 |
| C | 0.566650 | -1.739417 | -0.039980 |
| H | 1.240850 | -1.921979 | 0.804433 |
| H | -0.251983 | -2.456375 | -0.005662 |
| H | 1.161900 | -1.910912 | -0.943458 |
| C | 1.219919 | 1.361599 | -0.080901 |
| H | 1.000723 | 1.996647 | -0.946481 |
| H | 1.111548 | 2.003978 | 0.799894 |
| H | 2.248190 | 1.009867 | -0.145380 |
| Si | -0.000159 | 0.000077 | 0.000764 |
|  |  |  |  |

# References

[1] A. Engelbrecht, F. Sladky, *Angew. Chem. Int. Ed.* **1964**, *3*, 383.

[2] a) E. Mayer, F. Sladky, *Inorg. Chem.* **1975**, *14*, 589; b) F. Sladky, H. Kropshofer, O. Leitzke, P. Peringer, *J. Inorg. Nucl. Chem.* **1976**, *28*, 69.

[3] R. K. Harris, E. D. Becker, S. M. Cabral de Menezes, P. Granger, R. E. Hoffman, K. W. Zilm, *Magn. Reson. Chem.* **2008**, *46*, 582.

[4] Adept Scientific, *gNMR V 5.0*, **2005**.

[5] G. M. Sheldrick, *Acta Cryst. (Acta Crystallographica)* **2008**, *A64*, 112.

[6] G. M. Sheldrick, *Acta Cryst. A* **2015**, *71*, 3.

[7] O. V. Dolomanov, L. J. Bourhis, R. J. Gildea, J. A. K. Howard, H. Puschmann, *J. Appl. Cryst.* **2009**, *42*, 339.

[8] TURBOMOLE GmbH, *TURBOMOLE V7.6*. *a development of University of Karlsruhe and Forschungszentrum Karlsruhe GmbH, 1989-2007, TURBOMOLE GmbH, since 2007. Available from: http://www.turbomole.com.*, **2021**.

[9] a) A. D. Becke, *Phys. Rev. A* **1988**, *38*, 3098; b) C. Lee, W. Yang, R. G. Parr, *Phys. Rev. B* **1988**, *37*, 785; c) S. H. Vosko, L. Wilk, M. Nusair, *Can. J. Phys.* **1980**, *58*, 1200.

[10] M. Sierka, A. Hogekamp, R. Ahlrichs, *J. Chem. Phys.* **2003**, *118*, 9136.

[11] F. Weigend, R. Ahlrichs, *Phys. Chem. Chem. Phys.* **2005**, *7*, 3297.

[12] P. Deglmann, F. Furche, R. Ahlrichs, *Chem. Phys. Lett.* **2002**, *362*, 511.

[13] M. A. Ellwanger, C. von Randow, S. Steinhauer, Y. Zhou, A. Wiesner, H. Beckers, T. Braun, S. Riedel, *Chem. Commun.* **2018**, *54*, 9301.
